# Concurrent remodelling of nucleolar 60S subunit precursors by the Rea1 ATPase and Spb4 RNA helicase

**DOI:** 10.7554/eLife.84877

**Published:** 2023-03-17

**Authors:** Valentin Mitterer, Matthias Thoms, Robert Buschauer, Otto Berninghausen, Ed Hurt, Roland Beckmann

**Affiliations:** 1 Biochemistry Center, University of Heidelberg Heidelberg Germany; 2 Gene Center, University of Munich Munich Germany; Columbia University United States; Columbia University United States

**Keywords:** ribosome biogenesis, RNA helicases, AAA-ATPases, RNA restructuring, Spb4, Rea1, *S. cerevisiae*

## Abstract

Biogenesis intermediates of nucleolar ribosomal 60S precursor particles undergo a number of structural maturation steps before they transit to the nucleoplasm and are finally exported into the cytoplasm. The AAA^+^-ATPase Rea1 participates in the nucleolar exit by releasing the Ytm1–Erb1 heterodimer from the evolving pre-60S particle. Here, we show that the DEAD-box RNA helicase Spb4 with its interacting partner Rrp17 is further integrated into this maturation event. Spb4 binds to a specific class of late nucleolar pre-60S intermediates, whose cryo-EM structure revealed how its helicase activity facilitates melting and restructuring of 25S rRNA helices H62 and H63/H63a prior to Ytm1–Erb1 release. In vitro maturation of such Spb4-enriched pre-60S particles, incubated with purified Rea1 and its associated pentameric Rix1-complex in the presence of ATP, combined with cryo-EM analysis depicted the details of the Rea1-dependent large-scale pre-ribosomal remodeling. Our structural insights unveil how the Rea1 ATPase and Spb4 helicase remodel late nucleolar pre-60S particles by rRNA restructuring and dismantling of a network of several ribosomal assembly factors.

## Introduction

Eukaryotic ribosomes consist of a small (40S) and a large (60S) subunit that are comprised of four ribosomal RNAs (rRNAs) and 79–80 ribosomal proteins. While the yeast 40S subunit contains the 18S rRNA and 33 ribosomal proteins, the 60S subunit contains the 5S, 5.8S, and 25S rRNA species and 46 ribosomal proteins. The two ribosomal subunits are assembled in a complex highly regulated and hierarchical biogenesis process that starts in the nucleolus by transcription of a common precursor RNA (the 35S pre-rRNA) and the co-transcriptional association of the first ribosomal proteins and assembly factors. Upon endonucleolytic cleavage within the internal RNA spacer element 1 (ITS1), the pre-40S and pre-60S subunits follow separate biogenesis routes in the nucleolus, nucleoplasm, and finally cytoplasm. For reviews on ribosome biogenesis see Baßler and Hurt, 2019; de la Cruz et al., 2015; Klinge and Woolford, 2019; Kressler et al., 2017.

The precursors of the large ribosomal subunit undergo a cascade of consecutive rRNA folding steps in which the initially flexible six intertwined 25S rRNA domains (named in the 5’ to 3’ direction as domains I to VI) get integrated into the developing 60S core. While in the earliest precursors the 27SA_2_ pre-rRNA is highly flexible, the proportion of stably folded rRNA domains is strongly increased in succeeding 27SB pre-rRNA containing precursor particles (Burlacu et al., 2017). In line with chemical probing data, cryo-EM structures revealed that shaping of the immature rRNA occurs in a hierarchical order, in which first the 25S rRNA domains I, II, and parts of domain VI, together with the 5.8S rRNA and internal transcribed spacer 2 (ITS2), adopt a folded conformation and form the solvent-exposed back side of the pre-60S particle (structural states 1/A and 2/B) (Kater et al., 2017; Sanghai et al., 2018; Zhou et al., 2019b). Subsequent compaction of large parts of domains III, IV, and V results in formation of the polypeptide exit tunnel (PET) in state C to E particles. Transition of the 60S precursors to the nucleoplasm goes along with a re-positioning of the L1 stalk (helices H75-H78 of 25S rRNA domain V) allowing restructuring and incorporation of large parts of domain V and formation of the central-protuberance (CP) (state F/Nog2 particles) (Kater et al., 2020; Wu et al., 2016). As part of the CP, the 5S rRNA, which is transcribed independently from the other rRNA species and flexibly assembled at an earlier maturation stage, becomes stably integrated and structurally visible at this stage (Leidig et al., 2014; Wu et al., 2016). Upon subsequent removal of ITS2 and rotational movement of the 5S rRNA into its nearly mature orientation, the pre-60S intermediates are exported to the cytoplasm (Gasse et al., 2015; Kater et al., 2020; Matsuo et al., 2014; Schuller et al., 2018; Thoms et al., 2015; Wu et al., 2016; Zhou et al., 2019a). Here, final structuring of the inter-subunit side (ISS) and the peptidyl-transferase centre (PTC) completes 60S maturation and permits entrance of the assembled subunit to the pool of actively translating ribosomes (Kargas et al., 2019; Ma et al., 2017; Malyutin et al., 2017; Zhou et al., 2019a).

The accurate and efficient rRNA maturation and ribosome assembly requires ca. 80 small nucleolar RNAs (snoRNAs) and at least 200 non-ribosomal assembly factors. Among these assembly factors are a variety of energy consuming enzymes such as AAA^+^-ATPases and RNA helicases that facilitate key RNA and protein remodelling events on nascent ribosomal intermediates (Kressler et al., 2010). The activity of the dynein-related AAA^+^-ATPase Rea1 is required for two separate ATP-hydrolysis-dependent pre-60S maturation steps. In the earlier step, Rea1 releases assembly factor Ytm1 and its binding partner Erb1 from late nucleolar pre-60S particles (Bassler et al., 2010; Thoms et al., 2016; Wegrecki et al., 2015). In a second maturation step, which goes along with CP formation in the nucleoplasm, Rea1 dislodges assembly factor Rsa4 and thereby primes the 60S precursors for their export to the cytoplasm (Barrio-Garcia et al., 2016; Kater et al., 2020; Matsuo et al., 2014; Ulbrich et al., 2009). The interaction of Rea1 with Ytm1 and Rsa4 is established between a metal-ion-dependent adhesion site (MIDAS) at the C-terminal tip of the Rea1 tail and the ubiquitin-like (UBL) domains of its assembly factor ligands (Ahmed et al., 2019; Bassler et al., 2010; Mickolajczyk et al., 2022; Ulbrich et al., 2009). We previously revealed the architecture of Rea1 and its associated pentameric Rix1-complex, which serves as docking platform for the ATPase on nucleoplasmic pre-60S particles (Barrio-Garcia et al., 2016; Kater et al., 2020). Upon pre-60S association, a highly conserved loop motif within the Rea1 MIDAS facilitates the attachment of the C-terminal MIDAS onto the N-terminal AAA^+^ ring domain (Ahmed et al., 2019; Chen et al., 2018; Kater et al., 2020; Sosnowski et al., 2018). This intramolecular MIDAS docking may enable a direct force-transmission upon ATP hydrolysis from the AAA^+^ domain onto the MIDAS to dislodge the MIDAS-bound Rsa4 ligand. While there is no direct evidence that the Rix1-complex recruits Rea1 also to nucleolar 60S precursors, inhibition of Ytm1 release resulted in a shift of otherwise nucleoplasmic Rix1 particles to a nucleolar stage (Bassler et al., 2010). This suggests a function of the Rix1-complex for mediating Rea1 remodeling of its nucleolar substrate particles as well.

Around 20 RNA helicases involved in ribosome biogenesis have been identified so far (Granneman et al., 2006; Mitterer and Pertschy, 2022; Rodríguez-Galán et al., 2013). While it is suggested that these ATP-dependent enzymes promote crucial ribosomal restructuring steps including rRNA folding/re-arrangement or disassembly of snoRNAs, a precise molecular function was described only for very few RNA helicases. The DEAD-box helicase Spb4 is essential for cell viability and 60S assembly (de la Cruz et al., 1998; García-Gómez et al., 2011). Spb4 exhibits RNA-stimulated ATPase activity in vitro, which is abolished by a point mutation within the catalytic DEAD (Walker B) helicase motif (Brüning et al., 2018). Upon its depletion and in *spb4* catalytic mutant backgrounds, processing of the 27SB pre-rRNA on pre-60S particles was blocked (Brüning et al., 2018; de la Cruz et al., 1998; García-Gómez et al., 2011). In line with this, depletion of Spb4 impaired the recruitment of the late B-factor Nog2, which is necessary for subsequent 27SB processing (Talkish et al., 2012). A low-resolution cryo-EM density and CRAC (UV-crosslinking and cDNA analysis) experiments suggested that Spb4 binds to pre-60S intermediates at a hinge region at the base of eukaryote-specific expansion segment 27 (ES27) within 25S rRNA domain IV (Brüning et al., 2018; Kater et al., 2017; Sanghai et al., 2018). However, despite the proposed pre-60S location, the function of Spb4 on these intermediates and its rRNA restructuring substrate remain unknown.

Here, we report an essential function of the RNA helicase Spb4 and its interaction partner Rrp17 for the maturation of late nucleolar pre-60S particles. Spb4 and Rrp17 bind to such intermediates directly prior to their transition to the nucleoplasm, which is mediated by the Rea1 AAA^+^-ATPase. Consistent with a most recently published study (Cruz et al., 2022), our cryo-EM structure of Spb4 bound to pre-60S particles suggests how the helicase facilitates restructuring of its substrate rRNA before it can later fold into its mature-like conformation. Furthermore, the association of Spb4 with 60S precursors is required to allow the Rea1-dependent release of the Ytm1–Erb1 subcomplex. By coupling an in vitro maturation assay with isolated pre-60S particles to cryo-EM analyses, we could recapitulate this major structural transition during removal and destabilization of a large set of assembly factors, which promotes the nucleolar exit of 60S precursors.

## Results

### Spb4 and Rrp17 are enriched on late nucleolar pre-60S particles

**Figure 1.**
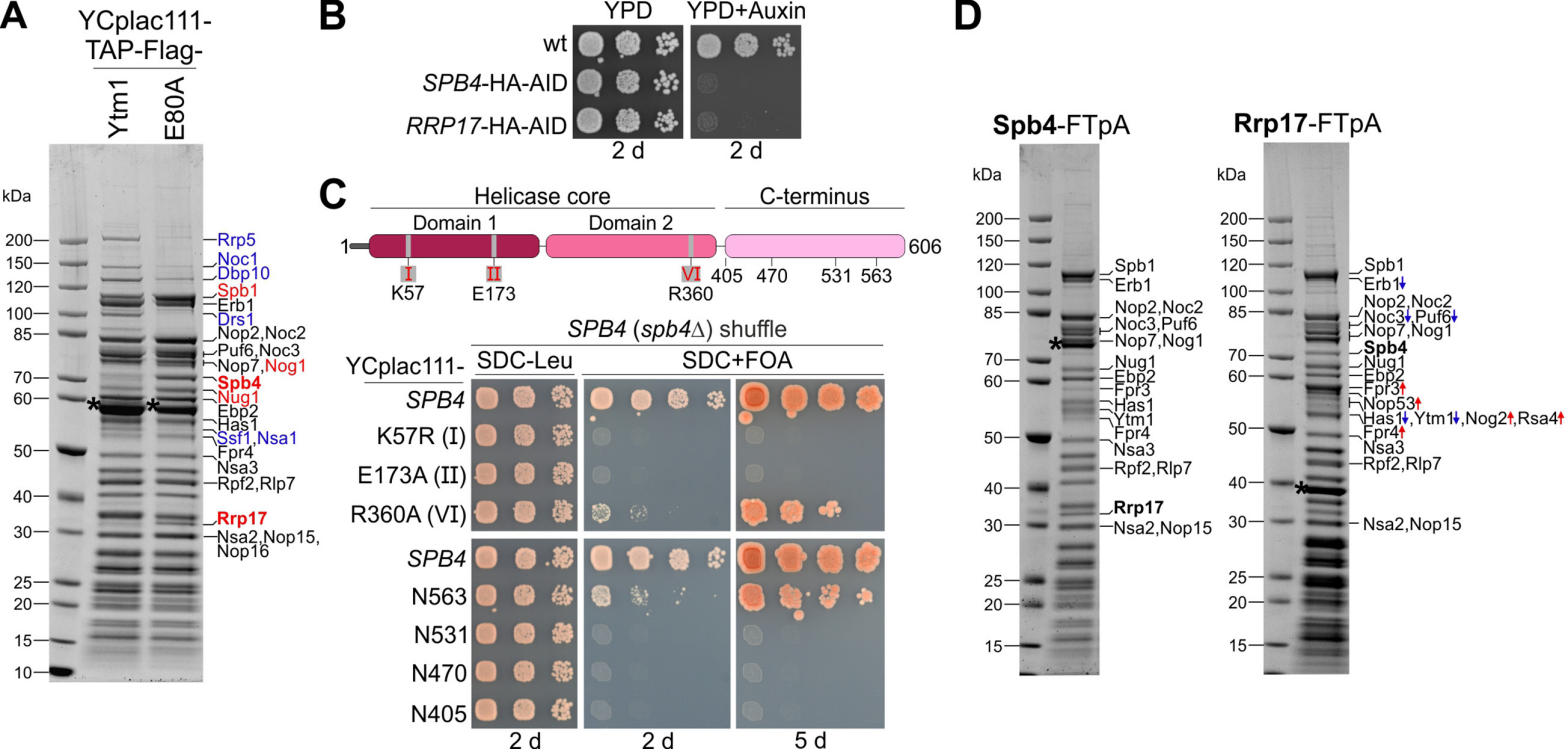
Spb4 and Rrp17 bind to late nucleolar 60S precursors. (**A**) Spb4 and Rrp17 are enriched on Ytm1 E80A particles. Plasmid-borne TAP-Flag tagged wild-type *YTM1* or the *ytm1* E80A mutant were affinity purified and final eluates analysed by SDS-PAGE and Coomassie staining. (**B**) *SPB4*-HA-AID and *RRP17*-HA-AID strains were spotted in tenfold serial dilutions on YPD plates without (YPD) or containing auxin (YPD + Auxin) and incubated for 2 d at 30°C. (**C**) Upper panel: Spb4 protein domain organization. Amino acids within the conserved helicase core signature motifs I (K57: ATP-binding), II (E173: ATP hydrolysis), and VI (R360: ATP-hydrolysis) that were mutated in this study are indicated. Lower panel: A *SPB4* (*spb4*Δ) shuffle strain was transformed with plasmids harbouring wild-type *SPB4* or indicated *spb4* mutant alleles. Transformants were spotted in tenfold serial dilutions on SDC-Leu or 5-FOA containing plates (SDC + FOA) and growth was monitored after incubation at 30°C for the indicated days. (**D**) Spb4-FTpA (left panel) and Rrp17-FTpA (right panel) affinity purifications enrich late nucleolar pre-60S particles. Pre-60S particles were isolated via chromosomally encoded Spb4- or Rrp17-FTpA. Final eluates were analysed by SDS-PAGE and Coomassie staining and indicated protein bands identified by mass spectrometry. Bands that show an increased or decreased relative intensity in the Rrp17 eluate compared to the Spb4 sample are depicted in red and blue, respectively. Note that the nucleoplasmic factors Nog2 and Rsa4 were detectable only in the Rrp17 sample. Bait proteins are marked with an asterisk. Figure 1—source data 1.Unedited and uncropped Coomassie-stained gels shown in Figure 1.Dashed boxes in the PDF indicate the respective areas shown in the figure.

During the transition from the nucleolus to the nucleoplasm, precursors of the 60S subunit undergo substantial changes regarding the rRNA folding status and concomitant association and dissociation of several assembly factors. Our previous cryo-EM analysis of a late nucleolar pre-60S particle (state E) purified via the dominant-lethal Ytm1 E80A mutant (this mutation impairs the Rea1-dependent release of the Ytm1–Erb1 subcomplex) revealed a low-resolution density for the DEAD-box RNA helicase Spb4 at the inter-subunit side, which was not present on earlier (states A–C) or later pre-60S maturation stages (states NE1, NE2, F) (Kater et al., 2020; Kater et al., 2017). Consistent with this observation, Spb4 was enriched on pre-60S particles isolated via Ytm1 E80A but not via wild-type Ytm1, whereas earlier nucleolar assembly factors (e.g. Noc1, Rrp5, Drs1) already dissociated at this stage (Figure 1A; Kater et al., 2017). Besides Spb4, another assembly factor, Rrp17, was accumulated on these Ytm1 E80A particles (Figure 1A), indicating that both, Spb4 and Rrp17, have a function during late steps of nucleolar pre-60S maturation.

**Figure 1—figure supplement 1.**
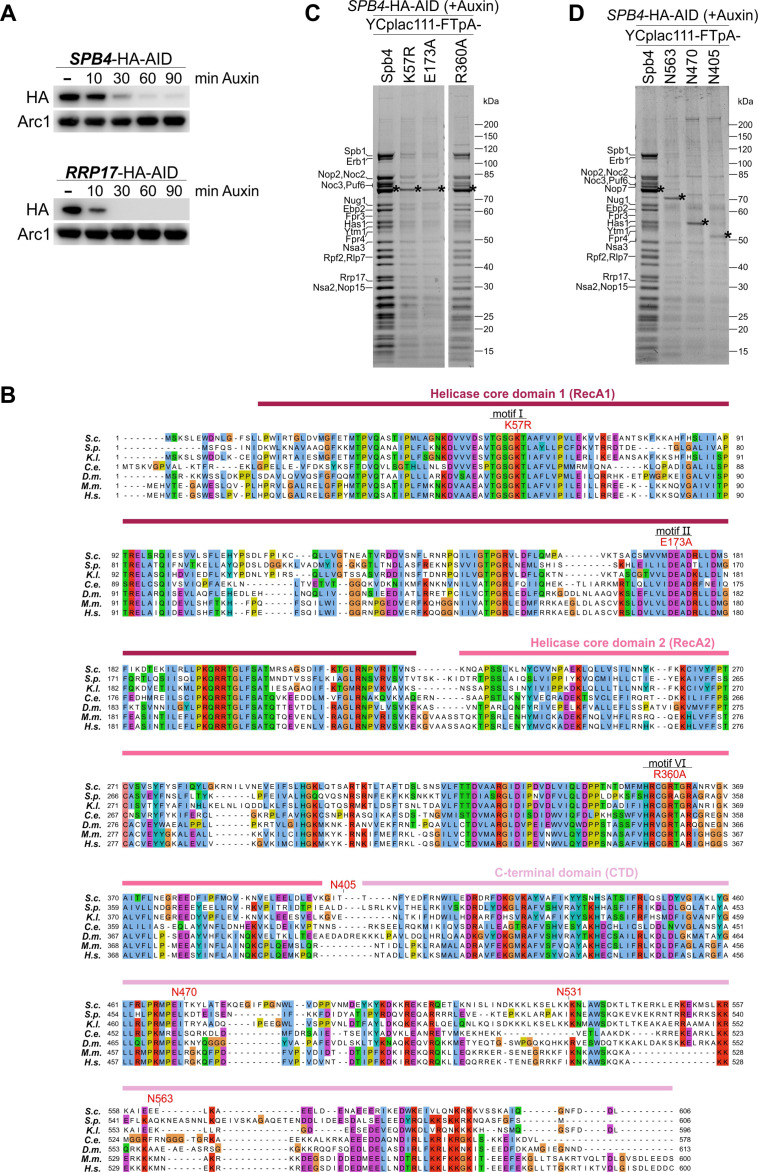
Affinity purifications using Spb4 mutants as bait protein. (**A**) Spb4-HA-AID and Rrp17-HA-AID are efficiently degraded in the presence of auxin. Logarithmically growing *SPB4*-HA-AID and *RRP17*-HA-AID cells were treated with 0.5 mM auxin and the level of Spb4-HA-AID and Rrp17-HA-AID protein degradation was assessed over 90 min by western blot analysis of whole-cell lysates using an anti-HA antibody. Equal sample loading was controlled by western blot analysis using an anti-Arc1 antibody. (**B**) Multiple sequence alignment of Spb4 orthologues. The Spb4 protein sequences from *Saccharomyces cerevisiae* (*S.c*.), *Schizosaccharomyces pombe* (*S.p*.), *Kluyveromyces lactis* (*K.l*.), *Caenorhabditis elegans* (*C.e*.), *Drosophila melanogaster* (*D.m*.), *Mus musculus* (*M.m*.), and *Homo sapiens* (*H.s*.) were aligned with Clustal omega and illustrated with Jalview. Point mutations introduced in catalytic helicase motifs and C-terminal truncation mutants are indicated. (**C, D**) Efficient pre-60S affinity purification via Spb4 is impaired in catalytic helicase mutants and C-terminal truncation mutants. Indicated plasmid-borne wild-type and mutant Spb4 variants fused to a C-terminal FTpA tag were affinity purified from cells upon auxin-induced depletion of chromosomal *SPB4*-HA-AID. Final eluates were analysed by SDS-PAGE and Coomassie staining. Figure 1—figure supplement 1—source data 1.Unedited and uncropped Coomassie-stained gels and western blots shown in Figure 1—figure supplement 1.Dashed boxes in the PDF indicate the respective areas shown in the figure.

As previous studies suggested a role of the *SPB4* and *RRP17* genes in ribosome biogenesis (Brüning et al., 2018; de la Cruz et al., 1998; García-Gómez et al., 2011; Oeffinger et al., 2009), we tested their essential functions by depletion of the Spb4-HA-AID and Rrp17-HA-AID proteins via an auxin-inducible degron (AID) approach, which as anticipated yielded non-viable yeast cells (Figure 1B, Figure 1—figure supplement 1A). Moreover, we analysed *spb4* point mutations of the catalytic helicase core domains (K57R: ATP-binding motif, E173A: ATP-hydrolysis motif, R360A: ATP-hydrolysis motif), and Spb4 C-terminal truncation mutants, which turned out to be lethal or exhibited a slow-growth phenotype (Figure 1C, Figure 1—figure supplement 1B). Subsequent affinity purifications of the helicase mutants and the C-terminal Spb4 truncations, all tagged with Flag-TEV-protein A (FTpA), showed that they still co-purify pre-ribosomal particles, however, somewhat less efficiently (Figure 1—figure supplement 1C and D). This indicates an essential pre-60S targeting function of the C-terminal domain (CTD), which was recently described also for DDX55, the human homologue of Spb4 (Choudhury et al., 2021), whereas another recent study on yeast Spb4 suggested that not the entire CTD is strictly required for pre-60S assembly (Cruz et al., 2022). Taken together, our results suggest that the Spb4 function on nascent 60S pre-ribosomes depends on both the catalytic and C-terminal domain.

In contrast to the tested *spb4* mutants, affinity purifications via wild-type Spb4 co-enriched a pattern of assembly factors highly similar to Ytm1 E80A particles (state E) and contained large amounts of Rrp17 (Figure 1D, left panel). Vice versa, Rrp17-FTpA affinity purifications co-enriched pre-60S particles with a comparable assembly factor composition and Spb4 but in contrast to Spb4 purifications also a few downstream nucleoplasmic assembly factors like Nog2, Rsa4, and the ITS2 foot-factor Nop53 (Figure 1D, right panel). Based on these affinity purifications, we conclude that both, Spb4 and Rrp17, join the maturation pathway around nucleolar state E. While Spb4 subsequently dissociates during or shortly after release of Ytm1–Erb1 by Rea1, Rrp17 may remain associated on downstream intermediates upon Nop53 association (Nop53 binds pre-60S particles at state NE1 upon Ytm1–Erb1 removal).

### Depletion of Spb4 and Rrp17 arrests pre-60S maturation at a stage directly before nucleolar exit

**Figure 2.**
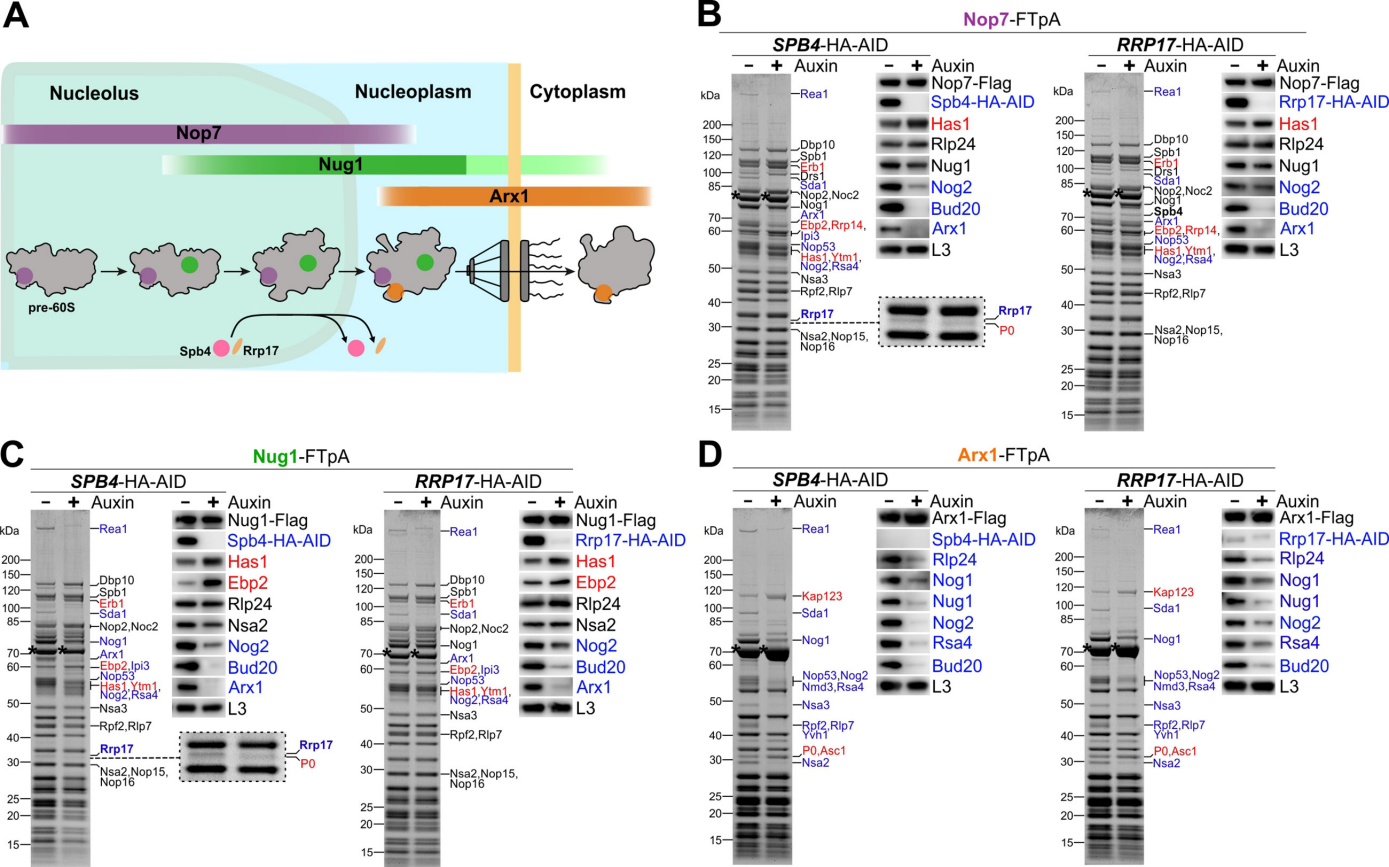
Spb4 and Rrp17 depletion arrests pre-60S maturation at a late nucleolar stage. (**A**) Colour-coded bait proteins selected for affinity purifications are associated with distinct maturation intermediates along the 60S biogenesis pathway in the nucleolus, nucleoplasm, and cytoplasm: Nop7 (violet), Nug1 (green), and Arx1 (orange). The proposed assembly/disassembly stage of Spb4 and Rrp17 is indicated. (**B–D**) Nop7-FTpA, Nug1-FTpA, and Arx1-FTpA were affinity purified from cells in the presence of Spb4-HA-AID or Rrp17-HA-AID (-Auxin) or upon their auxin-induced proteasomal degradation (+Auxin). Final eluates were analysed by SDS-PAGE and Coomassie staining (left panels) or western blotting using indicated antibodies (right panels). Depicted Coomassie-stained protein bands were identified by mass spectrometry. Bait proteins are marked with an asterisk. Protein bands with increased or decreased intensity in the depleted samples are indicated in red and blue, respectively. For a better visualization, the area of the Coomassie-stained gels of the Nop7 and Nug1 eluates to which Rrp17 migrates is enlarged (**B** and **C**, left panels; note that the additional protein band appearing in the Spb4-HA-AID-depleted samples was identified as ribosomal protein P0). Figure 2—source data 1.Unedited and uncropped Coomassie-stained gels and western blots shown in Figure 2B.Dashed boxes in the PDF indicate the respective areas shown in the figure. Figure 2—source data 2.Unedited and uncropped Coomassie-stained gels and western blots shown in Figure 2C.Dashed boxes in the PDF indicate the respective areas shown in the figure. Figure 2—source data 3.Unedited and uncropped Coomassie-stained gels and western blots shown in Figure 2D.Dashed boxes in the PDF indicate the respective areas shown in the figure.

To elucidate which particular pre-60S maturation step(s) require Spb4 and Rrp17, we assessed the impact of their cellular depletion on the assembly factor composition of purified pre-60S intermediates. Therefore, we generated chromosomal AID fusions of *SPB4* or *RRP17* in strains expressing FTpA-tagged Nop7, Nug1, or Arx1 and isolated these assembly factors from cells in presence of Spb4-HA-AID and Rrp17-HA-AID (-auxin) or after their auxin-induced degradation (+auxin) (Figure 2). In the absence of Spb4 or Rrp17, pre-60S maturation was effectively arrested at a late nucleolar stage, as apparent by the accumulation of assembly factors Ytm1–Erb1, Spb1, and Has1 on Nug1- and Nop7-derived particles, whereas the recruitment of Nop53 and downstream nucleoplasmic assembly factors such as Nog2 and Bud20 to these stalled maturation intermediates was impaired (Figure 2B and C). This suggests an important function of both Spb4 and Rrp17 for pre-60S maturation before Ytm1–Erb1 dissociation and transition of pre-60S particles to the nucleoplasm. Consistently, the export factor Arx1 that joins at a nucleoplasmic stage failed to efficiently co-purify pre-ribosomes upon Spb4 or Rrp17 depletion and was instead present mainly in a free form or associated with mature ribosomes or non-ribosomal factors (e.g. importins) (Figure 2D). Notably, while the association of Spb4 with Nug1- and Nop7-derived particles was a prerequisite for recruitment of Rrp17, the presence of Rrp17 was apparently not strictly required for Spb4-recruitment to these 60S precursors (Figure 2B, right panel; note that in Figure 2C Spb4 is co-migrating with the Nug1-Flag bait protein as revealed by mass spectrometry analyses).

Taken together, our data revealed an essential function of Spb4 and Rrp17 during final steps of nucleolar pre-60S maturation directly prior to release of the Erb1–Ytm1 subcomplex and subsequent assembly of Nop53. Thereby, Spb4 may associate with 60S precursors shortly before Rrp17 and help to mediate its incorporation.

### Cryo-EM structure of the Spb4 particle

**Figure 3.**
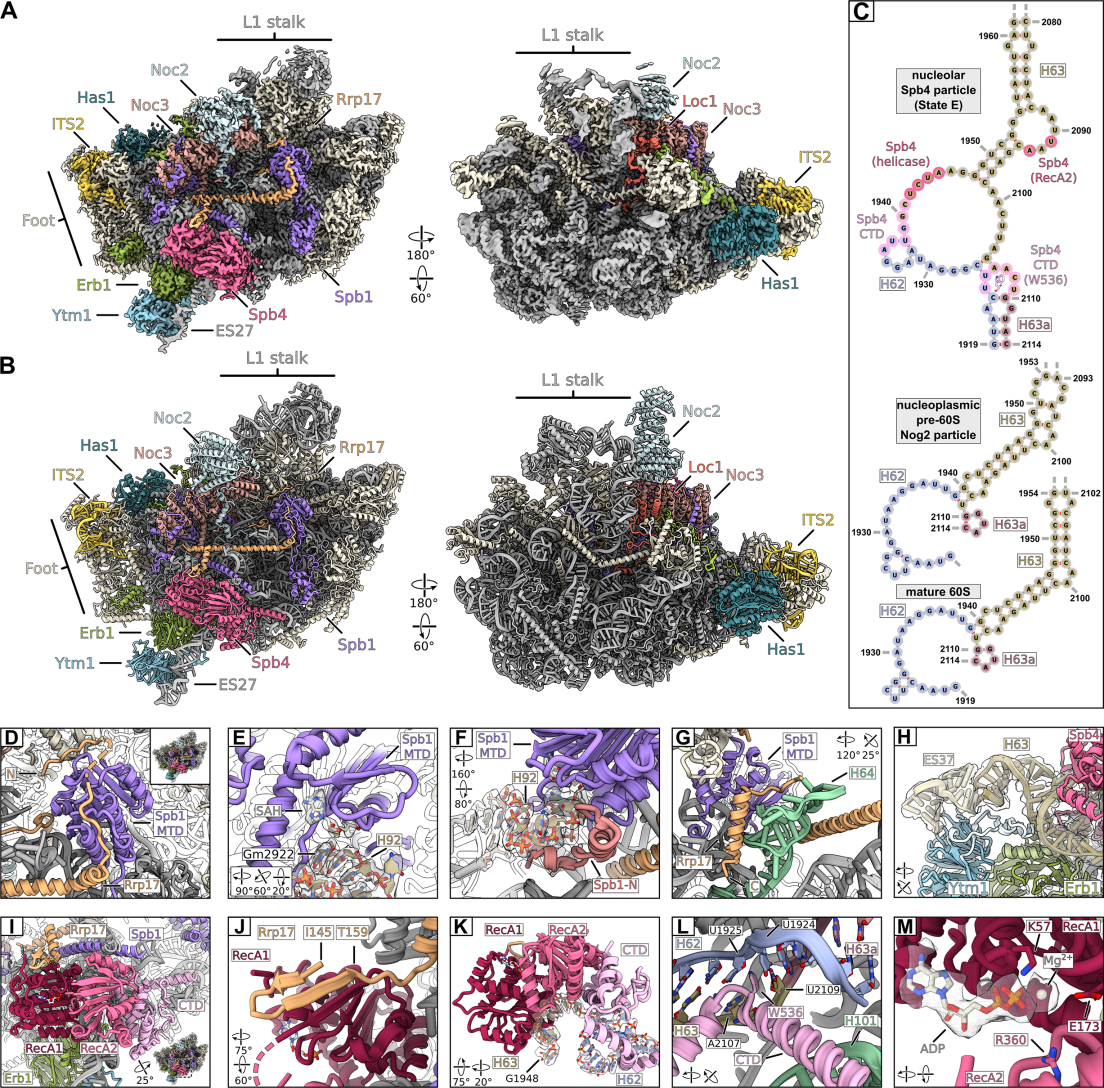
Cryo-EM structure of the nucleolar state E pre-60S subunit. (**A, B**) Cryo-EM density and model in two orientations, ribosome biogenesis factors relevant to the study are coloured and labelled. Ribosomal proteins and rRNA are shown in grey and additional biogenesis factors in beige. (**C**) Cryo-EM model-based secondary structure of H62, H63, and H63a of the rRNA in the nucleolar state E, the nucleoplasmic Nog2 particle (PDB-3jct) and mature ribosomes (PDB-6tb3) as comparison. Nucleotides interacting with Spb4 and the intercalating Trp536 of Spb4 are indicated. (**D–M**) Magnified regions focussing on Spb1, Spb4, and Rrp17. (**D**) The Spb1-MTD interacting with the N-terminus of Rrp17. (**E, F**) Spb1-MTD interacting with the A loop H92. The positioned Gm2922 and S-adenosyl homocysteine (SAH) are labelled and the segmented densities for H92 and SAH are shown. (**F**) The N-terminus (highlighted in red) of the Spb1-MTD clamps around H92 and helps positioning G2922. (**G**) Interaction of the Rrp17 C-terminus with H64 and the Spb1-MTD. (**H**) Ytm1 and Erb1 in contact with H63 and ES27. Insertions of Ytm1 (between ß16-ß17) and Erb1 (between ß8-ß9) are coloured in light blue and light green, respectively. The segmented density for ES27 and H63 is shown. (**I**) Overview of Spb4 bound to the state E pre-60S subunit. The RecA domains (RecA1, RecA2) and the C-terminal domain (CTD) are coloured and labelled. (**J–L**) Interaction of Spb4 with Rrp17 (**J**) and the immature H62/H63/H63a rRNA (**K, L**). (**M**) The Spb4 helicase core in complex with ADP. Residues mutated in this study are labelled and the segmented density for ADP is shown.

To obtain insight into the molecular function of Spb4, we sought to analyse the structure of the helicase bound to its pre-ribosomal 60S substrate particle and performed single-particle cryo-EM analysis with affinity-purified Spb4-FTpA intermediates. The major class of particles (40.85% of particles after 2D classification), which closely resembles the structural state E (Kater et al., 2017), was resolved at an overall resolution of 2.7 Å (Figure 3A and B, Figure 3—figure supplement 1A–C, and Figure 3—figure supplement 2A–E), whereas a minor class corresponded to state D at an overall resolution of 3.0 Å (Figure 3—figure supplement 1A–C and Figure 3—figure supplement 2F–I). The latter class contains in addition the Nsa1 module (consisting of assembly factors Nsa1, Rpf1, Mak16, and Rrp1) at the solvent-exposed pre-60S side but is otherwise identical to the better resolved state E (Figure 3—figure supplement 3A). The higher resolution of the state E compared to our previous study (Kater et al., 2017) allowed us to identify and build molecular models of assembly factors Spb4, Rrp17, Noc2, and Loc1, as well to resolve additional parts of Noc3, Spb1, Nsa3, and of the ITS2 RNA (Figure 3A and B, Figure 3—figure supplement 2A–E). At the inter-subunit side Spb4 is in contact with Rrp17 and located on top of the immature rRNA helices H62 and H63 in direct vicinity to the Ytm1–Erb1 complex (Figure 3A, B, H and I). The intertwined Loc1 is located at the solvent-exposed pre-60S side where it engages in multiple protein interactions with assembly factors Noc3, Spb1, Nip7, Erb1, Ebp2, Brx1, and ribosomal protein Rpl15 (eL15) (Figure 3A and B, Figure 3—figure supplement 3B). Next to Loc1, Noc3 interacts with the C-terminal part of its heat-repeat domain with its binding partner Noc2 (Figure 3B, Figure 3—figure supplement 3C). While the middle part of Noc2 (aa 86–176) is not resolved, the C-terminal Noc2 heat-repeat domain and the N-terminal part of the protein are visible, and both contribute to establish the interaction with Noc3. Besides its interaction with Noc3, Noc2 is anchored to the particle by its elongated N-terminus that protrudes deeply into the 60S core. There, the Noc2 N-terminus contacts the immature H35 rRNA, which likely requires Noc2 dissociation to adopt its mature conformation (Figure 3—figure supplement 3C). We were also able to resolve additional parts of the ITS2 spacer RNA (nucleotides 60–65 and 213–226) and the C-terminus of Nsa3 (aa 324–353) at the prominent pre-60S ‘foot’ structure (Figure 3—figure supplement 3D). This unveiled internal base-pairing of ITS2 bases 62–65 with 213–216, which is in line with a proposed ‘ring-pin’ model for the ITS2 structure based on chemical probing data (Burlacu et al., 2017). Furthermore, it revealed how the ITS2 RNA and Nsa3 are twined around and enclose assembly factor Nop15 (Figure 3—figure supplement 3D), which therefore can presumably dissociate only upon ITS2 processing and subsequent release of the foot factors (Gasse et al., 2015; Schuller et al., 2018).

**Figure 3—figure supplement 1.**
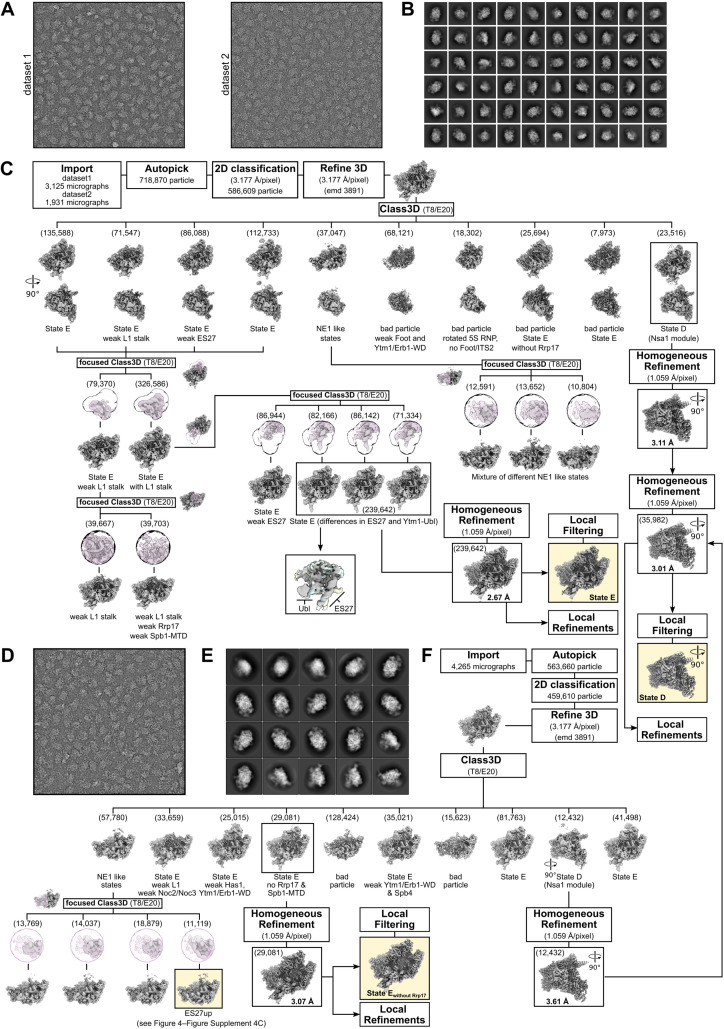
Cryo-electron microscopy data processing scheme. (**A, B**) Representative micrographs and 2D class averages of the two datasets used for processing of the Spb4-FTpA sample and determination of the state E structure. (**C**) Sorting scheme of the Spb4 sample. Final classes are indicated. (**D–F**) Representative micrograph (**D**), 2D classes (**E**), and processing scheme (**F**) of the sample used for determination of the state E-like structure without Rrp17/Spb1-MTD. Particles for state D from (**C**) and (**F**) were combined to increase the resolution of the final state D map. Particles of the NE1-like state with the ES27 rRNA in an upward conformation (**F**) were combined with an identical class described in Figure 4—figure supplement 4C. Final cryo-EM volumes are highlighted and shown with their respective resolution.

**Figure 3—figure supplement 2.**
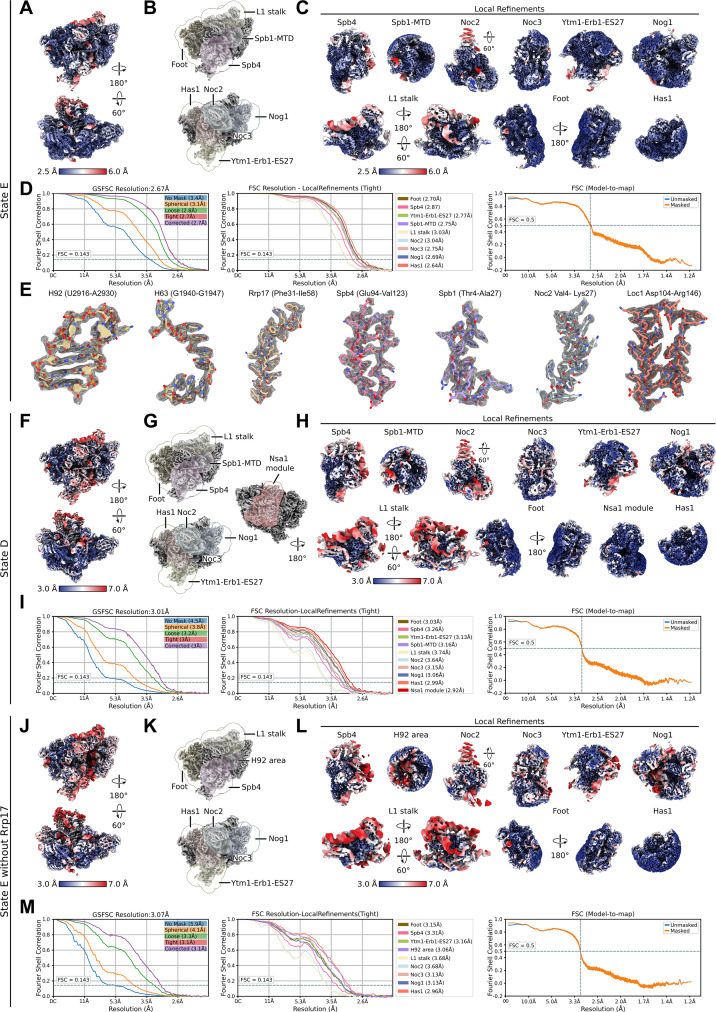
Local resolution distribution and Fourier shell correlation (FSC) curves for state E, state D, and state E lacking Rrp17. Shown are data for state E (**A–E**), state D (**F–I**), and the state E without Rrp17 (**J–M**). (**A, F, J**) Consensus cryo-EM map of state E, state D, and the state E lacking Rrp17 coloured according to local resolution. (**B, G, K**) The different consensus cryo-EM maps and masks used for the local refinements. (**C, H, L**) Local refinements coloured according to local resolution. (**D, I, M**) FSC of the consensus map (left), local refined maps (middle), and the model-to-map-FSC curves (right). (**E**) Segmented densities shown as mesh and corresponding models of individual parts of the nucleolar state E particle.

**Figure 3—figure supplement 3.**
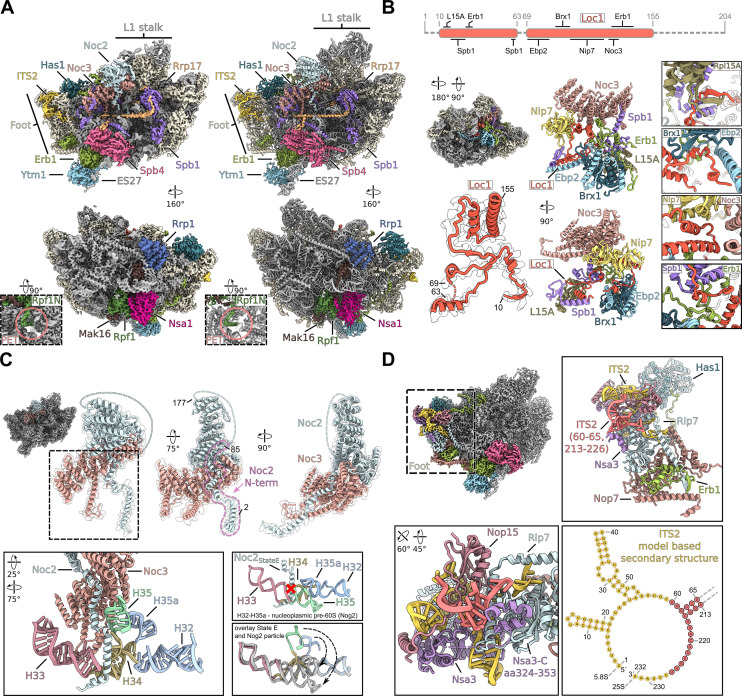
Structural details of nucleolar pre-60S states D/E. (**A**) Cryo-EM density (left panels) and model (right panels) of the nucleolar pre-60S state D containing the Nsa1 module (Nsa1–Rpf1–Mak16–Rrp1). Close-up views show the N-terminus of Rpf1 entering the polypeptide exit tunnel (PET). (**B**) The Loc1 interaction network. Schematic representation of the Loc1 interactions, unmodelled regions of Loc1 are shown as dashed lines (upper panel). Cryo-EM density of the state E class with the highlighted Loc1 interaction network and the molecular model and segmented density of Loc1 (left panels). Interaction network of the intrinsic Loc1 in two orientations. Only interacting regions and domains of the interacting factors are shown for simplicity (middle panels). Magnified regions of selected interactions between Loc1 and interacting proteins (right panels). (**C**) The Noc2–Noc3 complex shown in three orientations (upper panels). The Noc2 N-terminus (aa 2–85) is highlighted and the unmodelled middle part (aa 86–176) is indicated as dashed line. Interaction of the Noc2 N-terminus with rRNA H35, H32-H35 (nt 802–941) within state E is shown in different colours in the lower left panel. The lower right panels compare the orientation of H32-H35 in the nucleoplasmic Nog2 particle and the nucleolar state E. The sterical clash between the nucleoplasmic rRNA (Nog2 particle) and the Noc2 N-terminus (state E) is indicated showing that Noc2 release is required for H35 and H35a maturation. The H32-H35a rRNA of the Nog2 state (grey) is superimposed with the H32-H35a rRNA within state E (coloured). The rearrangements of H35 and H35a are indicated by arrows. (**D**) Cryo-EM map of the state E focussing on the ITS2-foot area. Magnifications of the foot-ITS2 region in different orientation are shown, and the new modelled region of the ITS2 (nt 60–65 and nt 213–226) is indicated in red. Secondary structure of the ITS2 based on the state E model (lower right panel).

**Figure 3—figure supplement 4.**
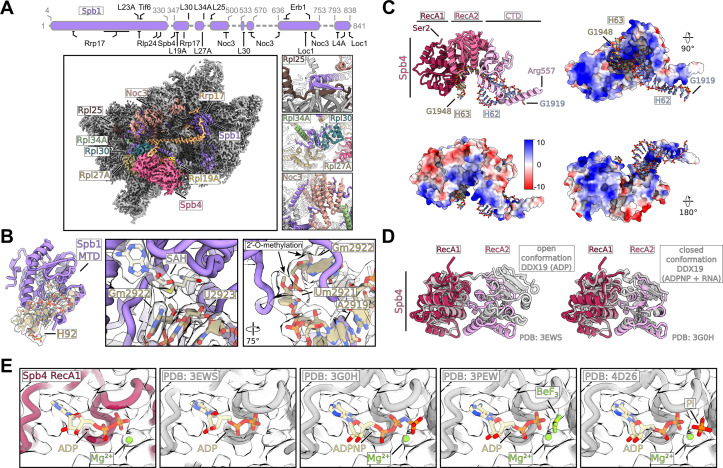
Structural details of Spb1 and Spb4. (**A**) Interaction network of Spb1 with biogenesis factors and ribosomal proteins identified in this study. A schematic overview of Spb1 with its various interaction partners is shown. Unmodelled parts are shown as dashed line (upper panel). Spb1 is shown as cartoon representation, and the state E subunit is shown as cryo-EM density. Interacting proteins identified in this study are coloured and labelled (lower left panel). Magnified regions showing selected interactions of Spb1 with Rpl25, Rpl30, Rpl34A, and Noc3 are shown (lower right). (**B**) The Spb1-MTD interacting with the A loop H92 (left panel). Close-ups of SAH and the methylated target Gm2922 in two orientations. Surrounding nucleotides including Um2921 are labelled (middle and right panel). The segmented densities for SAH and H92 are shown and the 2′-O-methylations of Gm2922 and Um2921 are highlighted. (**C**) Model of Spb4 interacting with the immature rRNA H62 and H63 (upper left panel) and surface views of Spb4 coloured by its electrostatic potential and in complex with H62-H63 are shown in different orientations. (**D**) The RecA domains of Spb4 are in a closed conformation in state D/E. Comparison of the Spb4 helicase core model with superimposed models of DDX19 in an open conformation bound to ADP (PDB: 3EWS) and in the closed conformation bound to ATP-analogue (ADPNP) and target RNA (PDB: 3G0H). The DDX19 models were fitted to the RecA1 domain of Spb4. The CTD of Spb4, the N-terminus of DDX19 as well as nucleotides and target RNAs are omitted for clarity. (**E**) Close-up of the nucleotide binding pocket within the Spb4 RecA1 domain. The model and the transparent cryo-EM density are shown and the models of ADP and Mg^2+^ are labelled (left panel). Models of different RNA helicases were rigid body fitted into the Spb4 (state E) density and are shown in grey. Different bound nucleotides and Mg^2+^ are labelled. The models of the human DDX19 in complex with ADP (PDB: 3EWS) and ADPNP (PDB: 3G0H), the *S. cerevisiae* Dbp5 L327V bound to ADP BeF_3_ and the Vasa helicase E339Q mutant from *B. mori* bound to ADP and Pi were used for comparison.

**Figure 3—figure supplement 5.**
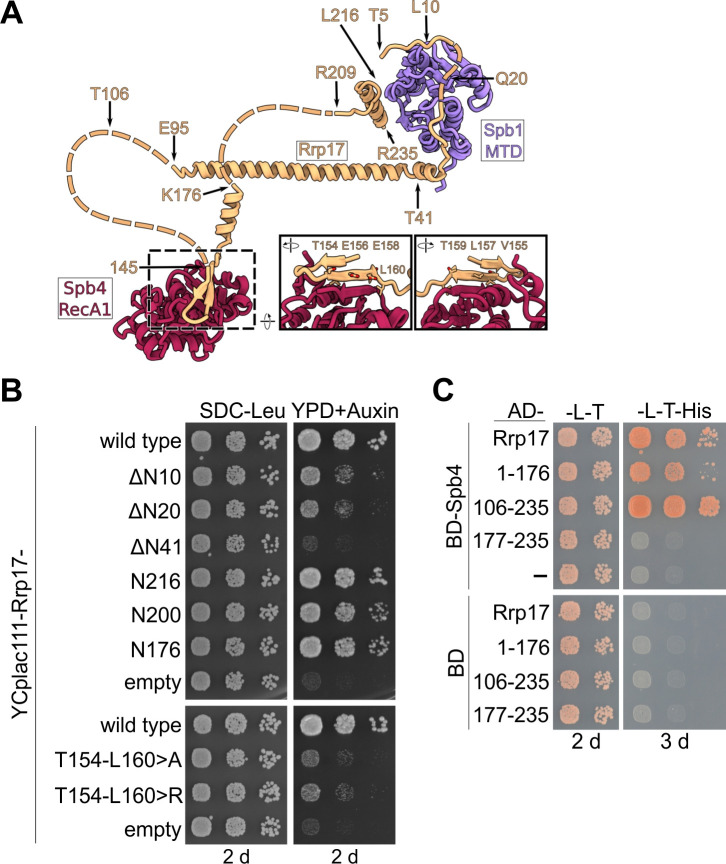
Importance of the Spb4–Rrp17 β-sheet augmentation for cell growth and protein–protein interaction. (**A**) Rrp17 in contact with the Spb1-MTD and Spb4-RecA1 domains in state E pre-60S subunits. Truncations and mutations of Rrp17 used for growth complementation and Y2H studies (**B **and **C**) are indicated. (**B**) An *RRP17*-HA-AID strain was transformed with plasmids harbouring wild-type *RRP17* or the indicated *rrp17* mutant alleles. Transformants were spotted in tenfold serial dilutions on SDC-Leu plates or YPD plates containing 2 mM auxin (YPD + Auxin) and growth monitored after incubation for 2 d at 30°C. (**C**) Spb4 fused to the Gal4 DNA-binding domain (BD) and indicated Rrp17 constructs fused to the Gal4 activation domain (AD) were transformed into the PJ69-4A yeast two-hybrid strain. Cells were spotted in ten-fold serial dilutions on SDC-Leu-Trp (-L-T) and SDC-Leu-Trp-His (-L-T-His; growth on this medium indicates a yeast two-hybrid interaction) plates and growth was monitored after incubation at 30°C for 2 and 3 d, respectively.

**Figure 3—figure supplement 6.**
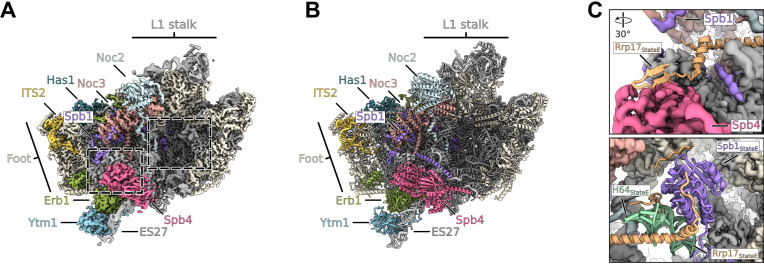
Cryo-EM structure of the state preceding state E lacking Rrp17 and the Spb1-MTD. (**A, B**) Coloured map (**A**) and model (**B**) of the state E like state lacking Rrp17 and the N-terminal MTD domain of Spb1. Relevant factors are coloured and labelled. (**C**) Coloured cryo-EM density of the state lacking Rrp17 focussing on the Spb4 RecA1 region (upper panel) and the region where the Spb1-MTD domain binds in state E (lower panel). The models of Rrp17, the Spb1-MTD, and rRNA H64 of the state E were overlaid.

### The α-helical Rrp17 connects Spb4 and the methyltransferase Spb1

While the methyltransferase Spb1 was already visualized in previous cryo-EM structures (Kater et al., 2020; Kater et al., 2017), we were able to model additional parts (between aa 347–570) of the so far unresolved widely meandering central region of the protein revealing interactions with Noc3, Rpl25 (uL23), Rpl30 (eL30), and Rpl34 (eL34) (Figure 3A and B, Figure 3—figure supplement 4A). Notably, we identified a ligand within the binding pocket of Spb1’s methyltransferase domain (MTD), which is most probably S-adenosyl homocysteine (SAH) (Figure 3E, Figure 3—figure supplement 4B), suggesting that methylation of its 2'-O-ribose target in nucleotide G2922 of the A-site loop (Lapeyre and Purushothaman, 2004) has occurred at this stage. The G2922 methylation was most recently shown to be monitored by the GTPase Nog2 establishing a crucial checkpoint for pre-60S maturation (Yelland et al., 2023). To facilitate this modification, the very N-terminal part of Spb1 (aa 4–27) may play an important role for target recognition as it clamps 25S rRNA H92 that harbours nucleotide G2922 and thereby suitably positions it for methylation (Figure 3E and F). In agreement with 2'-O-ribose methylation of G2922 at the analysed stage, we observed Gm2922 in the corresponding RNA density as well as adjacent Um2921 (Figure 3E, Figure 3—figure supplement 4B), which is in a redundant function methylated by Spb1 or the snR52 C/D box snoRNP at an earlier pre-60S biogenesis step (Bonnerot et al., 2003; Lapeyre and Purushothaman, 2004).

The α-helical Rrp17 is present at the inter-subunit side clamped between the MTD of Spb1 and the helicase core of Spb4 (Figure 3A and B, Figure 3—figure supplement 5A). While the essential unstructured Rrp17 N-terminus is meandering across the MTD of Spb1 (Figure 3D), a more central part of the protein (aa 145–159) is reaching towards Spb4 forming an anti-parallel β-sheet with the N-terminal RecA-like domain of the helicase (Figure 3I and J, Figure 3—figure supplement 5A). Prompted by this β-sheet augmentation, we analysed the growth of yeast cells upon mutation of the corresponding Rrp17 amino acids (154–160). As observed for N-terminal (Spb1 interaction) but not C-terminal Rrp17 truncation mutants, disruption of the β-sheet augmentation in *rrp17* 154–160>A and 154–160>R mutant cells yielded slow-growth phenotypes, revealing the importance of this Rrp17–Spb4 contact (Figure 3—figure supplement 5B). Moreover, a yeast two-hybrid interaction between Spb4 and Rrp17 was observed, mediated by the central part of Rrp17 (aa 106–177) that includes amino acids 154–160 (Figure 3—figure supplement 5C). The C-terminal part of Rrp17 (aa 209–235) is reaching back and contacts again the Spb1 MTD in proximity of its N-terminus, as well as 25S rRNA H64 (Figure 3G).

### Spb4-containing pre-60S intermediates up- and downstream of state E

In the major class of the obtained state E-like Spb4 particles, Rrp17 and Spb1 including its N-terminal MTD were already incorporated as described above. In addition, the Spb4 purifications contained a mixture of distinct NE1-like states downstream of Ytm1–Erb1 removal (Kater et al., 2020) as well as state E-like particles with only weak densities for the L1 stalk, Rrp17, and the Spb1 MTD, potentially representing intermediates upstream of state E (Figure 3—figure supplement 1C). Moreover, analysis of another independent dataset of purified Spb4 particles, which underwent an only partially efficient maturation reaction, revealed an additional E-like state (resolved at 3.1 Å) lacking the corresponding Rrp17 and Spb1 MTD densities but show stable incorporation of the L1 stalk (Figure 3—figure supplement 1D–F, Figure 3—figure supplement 2J–M, Figure 3—figure supplement 6). We conclude that this state may indeed represent a novel maturation intermediate directly preceding state E. Taken together, our cryo-EM analyses suggest that Spb4 is assembled around state D/E shortly prior to final Spb1 MTD and Rrp17 incorporation, which agrees with the biochemical protein data of purified pre-60S particles upon Spb4 and Rrp17 depletion (Figure 2).

### Spb4 accommodates ADP and promotes H62/H63/H63a restructuring

Our cryo-EM structure gave several insights into the molecular interactions of the Spb4 catalytic and C-terminal domains on the pre-60S particle (Figure 3A–C , and H–L). Most importantly, we observe a single-stranded rRNA (nucleotides U1942 to A1945 of 25S rRNA H63) within the helicase core active site (Figure 3K, Figure 3—figure supplement 4C). The accommodation of the rRNA substrate between the two RecA-like domains induces bending and strand separation of the rRNA around the base of ES27, resulting in an alternate base-pairing of helices H62/H63/H63a compared to nucleoplasmic maturation intermediates and mature 60S subunits (Figure 3C and K). This may explain why the rRNA area at the base of 25S domain IV initially appears to form stable duplexes, while it becomes more flexible and accessible for chemical modification in presence of Spb4, suggesting that the helicase disrupts this region upon its association (Brüning et al., 2018; Burlacu et al., 2017). In addition to the catalytic domain, Spb4’s essential CTD appears significantly involved in inducing substrate RNA strand disruption and establishing this alternate conformation. In the obtained substrate-bound state, the first half of the CTD (aa 406–499) is tightly docked onto the C-terminal RecA-like domain (RecA2) and binds H62/H63 nucleotides A1936 to C1941, thereby maintaining separation of the rRNA strands (Figure 3C, K and L, Figure 3—figure supplement 4C). Furthermore, a conserved tryptophan (W536) within the flexible C-terminal tail of the CTD (aa 500–606) intercalates between nucleotides of the immature H62/H63/H63a rRNA, which later adopts its mature-like fold in nucleoplasmic pre-60S particles (Figure 3C and L).

Two main conformations for the catalytic RNA helicase core domains have been described, which depend on the nucleotide and substrate binding state. Cooperative ATP and RNA substrate binding bring the two RecA domains in a closed conformation, while subsequent ATP hydrolysis usually leads to substrate release and re-opening of the RecA domains (Andersen et al., 2006; Bono et al., 2006; Mallam et al., 2012; Sengoku et al., 2006; Theissen et al., 2008). Examination of the catalytic Spb4 core in our structure revealed that, while the two RecA domains are in a clear closed conformation and tightly bound to the substrate RNA, the helicase surprisingly accommodates in its binding pocket a nucleotide which we could unambiguously identify as ADP (Figure 3M, Figure 3—figure supplement 4D and E). Thus, Spb4 has already hydrolysed ATP at a previous step but remains associated with pre-ribosomes in a post-catalytic and still ADP-bound closed state and preserves its re-arranged rRNA substrate in the observed immature alternate conformation as also observed in a recent structure (Cruz et al., 2022). This unusual behaviour can be explained by the requirement of ATP binding and hydrolysis to provide the energy for binding and local strand melting of the rRNA which is then followed by a subsequent stabilization of this state through the observed interactions of Sbp4 with several assembly factors such as Rrp17. As suggested by Cruz et al., 2022, it would also allow for a concerted release of Spb4 together with Ytm1 and other factors upon Rea1 activity, thereby facilitating the timely re-annealing of the H62-H63 rRNA module in the mature conformation as observed in the subsequent NE states. Thus, the interaction and activity of Sbp4 appears to set the stage for the correct formation of the H61-H62-H63-H64 rRNA region as an important prerequisite for the further maturation of rRNA domain IV.

### Rea1-dependent nucleolar pre-60S maturation can be induced on isolated Spb4 particles in vitro

**Figure 4.**
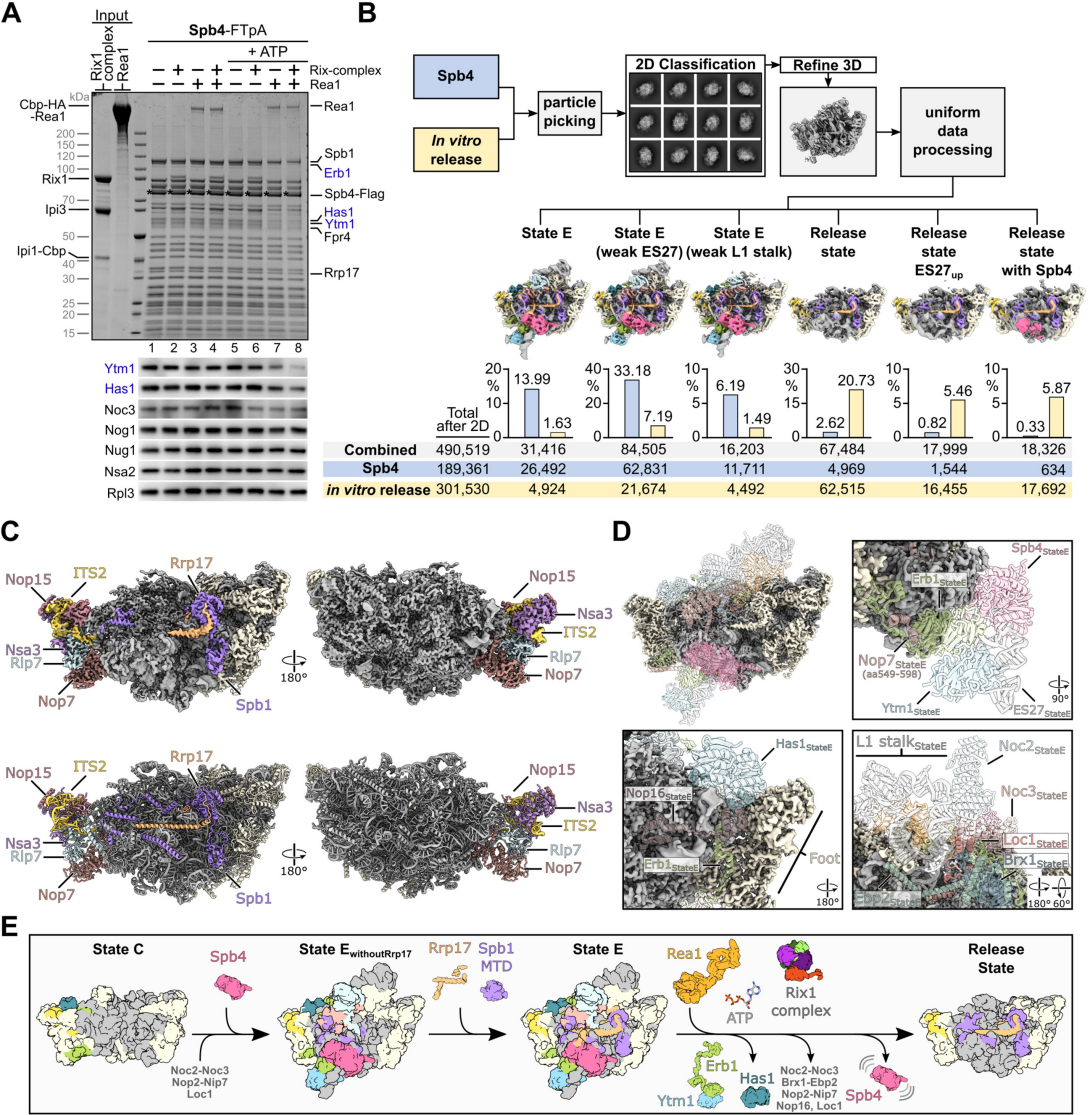
Rea1-dependent remodelling induced on isolated Spb4 particles in vitro. (**A**) Rea1 releases the Ytm1–Erb1 subcomplex together with RNA helicase Has1 from pre-60S particles. TEV-eluates of pre-60S particles isolated via Spb4-FTpA were as indicated incubated with purified Rea1, Rix1-complex (Rix1_2_–Ipi3_2_–Ipi1) and/or 3 mM ATP for 45 min at room temperature. Still bound material was re-isolated via Spb4-Flag by incubation with anti-Flag agarose beads and eluted with Flag peptide. Final eluates were analysed by SDS-PAGE and Coomassie staining (upper panel) or western blotting using the indicated antibodies (lower panel). The Spb4-Flag bait protein is marked with an asterisk and the released assembly factors Ytm1, Erb1, and Has1 are indicated in blue (**B**) Single-particle cryo-EM analyses of Spb4 control and in vitro matured pre-60S samples. Micrographs of the untreated Spb4 control and the in vitro release samples (corresponding to lanes 1 and 8 in (**A**), respectively) were combined for data processing. After reference free particle picking and 2D classification, the combined dataset was processed and six final classes are shown (maps are 3 x binned and coloured according to the colour scheme in Figure 3). The diagrams indicate the percentage of particles from the Spb4 and in vitro release dataset corresponding to the individual cryo-EM reconstruction. Combined particle numbers of each class as well as the particle numbers of the individual datasets (Spb4 control and in vitro release) are shown underneath. (**C**) Cryo-EM map (upper panels) and model (lower panel) of the main release state. Spb1, Rrp17 and the foot factors are coloured and labelled and additional biogenesis factors are highlighted in beige. (**D**) Overview and magnified regions of the release state cryo-EM volume with ribosomal proteins and rRNA coloured in grey and biogenesis factors and ITS2 RNA coloured in beige. Superposition of transparent molecular models from the preceding state E indicating factors and rRNA which are destabilised/released in the release state class. (**E**) Model summarising the results of this study. Spb4 is assembled at a late nucleolar stage and remains attached in a helicase-core closed conformation upon ATP hydrolysis. Rrp17 is incorporated at state E, at which also the Spb1-MTD gets positioned for methylation of G2922. Rea1-dependent release of Ytm1–Erb1 facilitates large-scale remodelling of the particle and transition to the NE1-like release state. Figure 4—source data 1.Unedited and uncropped Coomassie-stained gels and western blots shown in Figure 4A.Dashed boxes in the PDF indicate the respective areas shown in the figure.

Next, we wanted to analyse in further detail how the Rea1 ATPase extracts Ytm1–Erb1 from nucleolar pre-60S particles. Therefore, we aimed to induce Rea1-dependent maturation of isolated TAP-Ytm1 pre-60S intermediates in vitro. We incubated Ytm1-derived particles with affinity purified Rea1, the pentameric Rix-complex (i.e. Rix1_2_–Ipi3_2_–Ipi1; this complex is suggested to guide Rea1 to its pre-ribosomal substrates), and ATP. However, upon subsequent re-isolation of pre-60S particles via Rpl3-Flag, the Ytm1–Erb1 complex remained fully associated with the pre-ribosomes (Figure 4—figure supplement 1A). Strikingly, performing this maturation assay with Spb4-purified pre-60S particles exhibited an efficient release of the Ytm1–Erb1 complex and, remarkably, the DEAD-box helicase Has1 (Figure 4A, lane 8). The release of these assembly factors was more efficient in presence of the Rix1-complex, however, the addition of Rea1 and ATP was in principle sufficient to perform the restructuring step, at least in this in vitro setup with excess of purified Rea1 (Figure 4A, lane 7). The in vitro maturation also occurred when the slow growing catalytic *spb4* R360A mutant was used (Figure 1C, Figure 4—figure supplement 1B and D), which indicates that ATP-hydrolysis by Spb4 is not required for this maturation step. However, since in vitro maturation was inducible on Spb4- but not on Ytm1-derived pre-60S particles that co-purify only small amounts of the helicase Spb4 (Figure 1A), we conclude that pre-60S recruitment of Spb4 and potentially also Rrp17 is a prerequisite for this remodelling step. Indeed, also incubation of Rrp17 particles with Rea1, Rix1-complex, and ATP resulted in limited but clearly detectable release of the sub-stoichiometrically associated Ytm1–Erb1 complex (Figure 4—figure supplement 1C).

**Figure 4—figure supplement 1.**
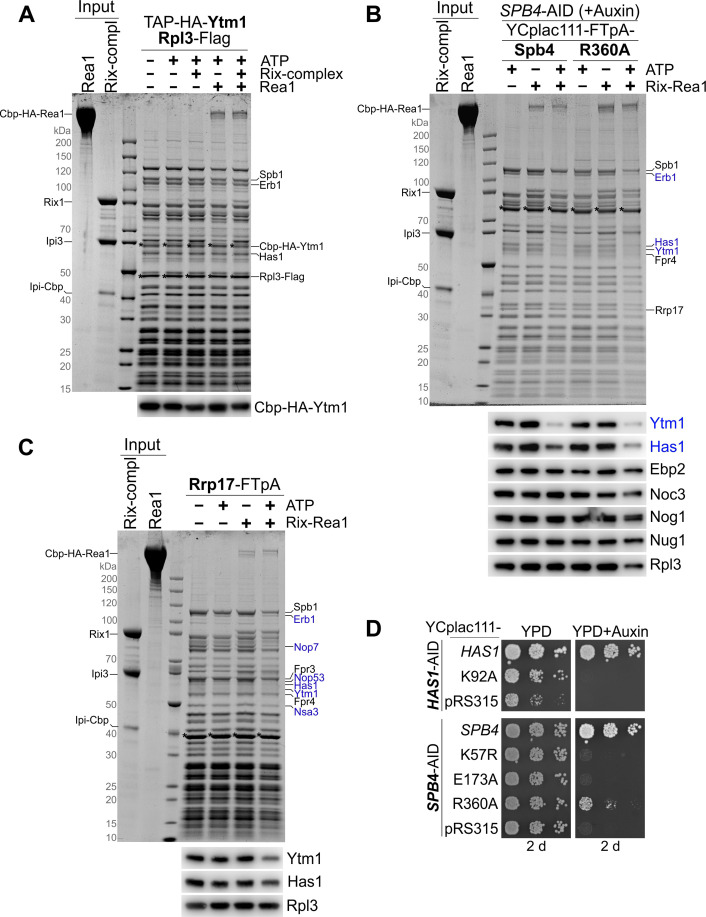
In vitro maturation assays utilizing distinct substrate particles. (**A**) Rea1-dependent remodelling cannot be induced on Ytm1-derived pre-60S particles. TEV-eluates of pre-60S particles isolated via TAP-HA-Ytm1 were as indicated incubated with purified Rea1, Rix1-complex (Rix1_2_–Ipi3_2_–Ipi1) and/or 3 mM ATP for 45 min at room temperature. Still bound material was re-isolated via Rpl3-Flag by incubation with anti-Flag agarose beads and eluted with Flag peptide. Final eluates were analysed by SDS-PAGE and Coomassie staining (upper panel) or western blotting using an anti-HA antibody to detect Ytm1 (lower panel). (**B**) Ytm1–Erb1 and Has1 are released from Spb4 R360A-derived mutant particles. Plasmid-borne FTpA tagged wild-type and R360A mutant Spb4 proteins were isolated from cells upon auxin-induced depletion of chromosomal *SPB4*-HA-AID. TEV eluates were incubated with Rea1, Rix1-complex and/or 3 mM ATP as described above and re-isolated via Spb4-Flag. Final eluates were analysed by SDS-PAGE and Coomassie staining (upper panel) or western blotting with the indicated antibodies (lower panel). (**C**) TEV-eluates of pre-60S particles isolated via Rrp17-FTpA were incubated with Rea1, Rix1-complex and/or 3 mM ATP as described above and re-isolated via Rrp17-Flag. Final eluates were analysed by SDS-PAGE and Coomassie staining (upper panel) or western blotting with the indicated antibodies (lower panel). Released proteins are indicated in blue. The bait proteins are marked with an asterisk. (**D**) A *HAS1*-HA-AID strain was transformed with plasmids harbouring wild-type *HAS1* or the *has1* K92A allele (upper panel) and a *SPB4*-HA-AID strain was transformed with plasmids harbouring indicated *spb4* alleles (lower panel). Transformants were spotted in tenfold serial dilution on YPD plates with or without auxin and growth assessed after incubation at 30°C for 2 d. Figure 4—figure supplement 1—source data 1.Unedited and uncropped Coomassie-stained gels and western blots shown in Figure 4—figure supplement 1.Dashed boxes in the PDF indicate the respective areas shown in the figure.

**Figure 4—figure supplement 2.**
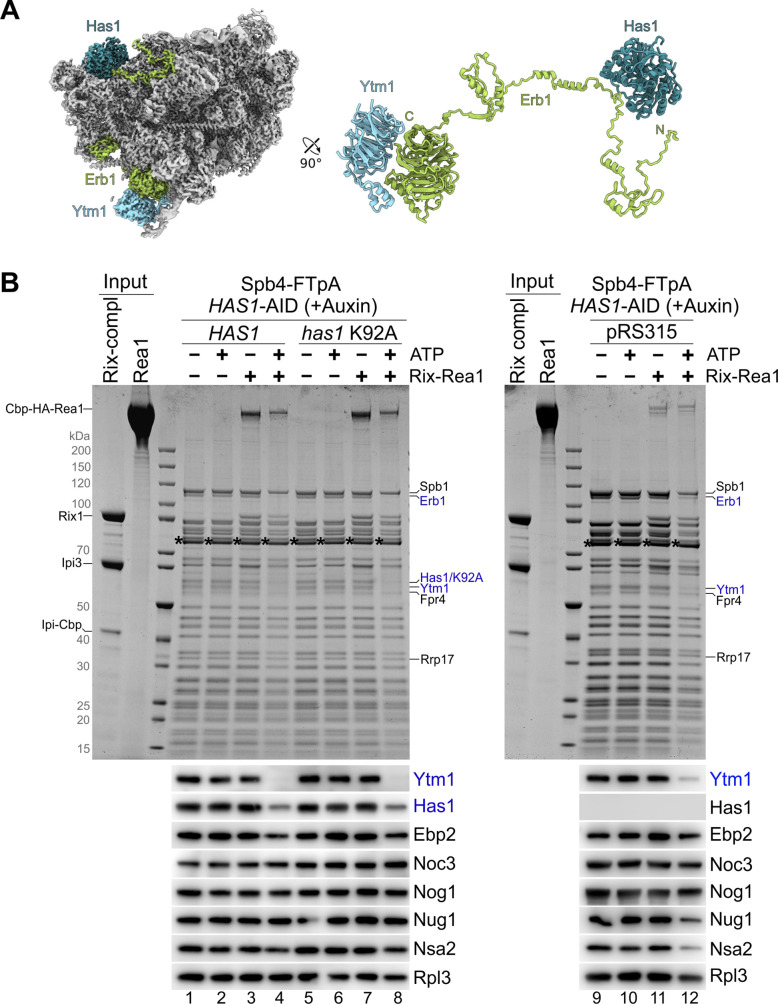
Catalytically inactive Has1 K92A protein is dislodged from Spb4 particles upon Ytm1–Erb1 release. (**A**) Cryo-EM reconstruction of the nucleolar state E pre-60S particle showing the contact between Has1 and the long unstructured Erb1 N-terminal tail. Ytm1 (light blue), Erb1 (green), and Has1 (blue) are displayed bound to the pre-60S particle (grey) as coloured cryo-EM map (left panel) or enlarged as cartoon representation (right panel). (**B**) Spb4-FTpA particles were isolated upon *HAS1*-HA-AID depletion from cells expressing wild-type *HAS1* or a *has1* K92A Walker A mutant (left panel) or in absence of plasmid-borne *HAS1* (right panel). Spb4 TEV eluates were as indicated incubated with purified Rea1, Rix1-complex (Rix1_2_–Ipi3_2_–Ipi1) and/or 3 mM ATP for 45 min at room temperature. Still bound material was re-isolated via Spb4-Flag by incubation with anti-Flag agarose beads and eluted with Flag peptide. Final eluates were analysed by SDS-PAGE and Coomassie staining (upper panels) or western blotting using the indicated antibodies (lower panels). The released proteins are indicated in blue. Figure 4—figure supplement 2—source data 1.Unedited and uncropped Coomassie-stained gels and western blots shown in Figure 4—figure supplement 2.Dashed boxes in the PDF indicate the respective areas shown in the figure.

**Figure 4—figure supplement 3.**
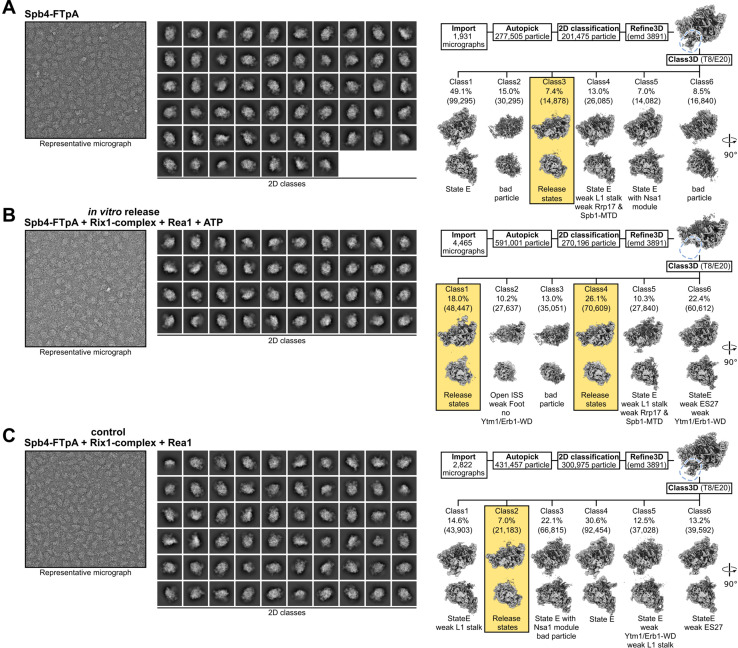
Comparison of Spb4, in vitro release, and control samples. Processing schemes for the (**A**) Spb4-FTpA sample, (**B**) the in vitro release sample (Spb4-FTpA+Rix1 complex + Rea1 and ATP), and (**C**) the control sample (Spb4-FTpA+Rix1 complex + Rea1 but lacking ATP). The left and middle panels show representative micrographs and 2D class averages, respectively. The right panels show the initial processing schemes. After the initial refinement (Refine3D), density for the Ytm1-WD and Erb1-WD complex is clearly visible in the Spb4 and control samples but absent in the in vitro release sample (indicated with blue dashed lines). Subsequent 3D classification for each dataset is shown and the percentage and particle numbers (in brackets) are shown. The release state classes of each dataset are highlighted.

**Figure 4—figure supplement 4.**
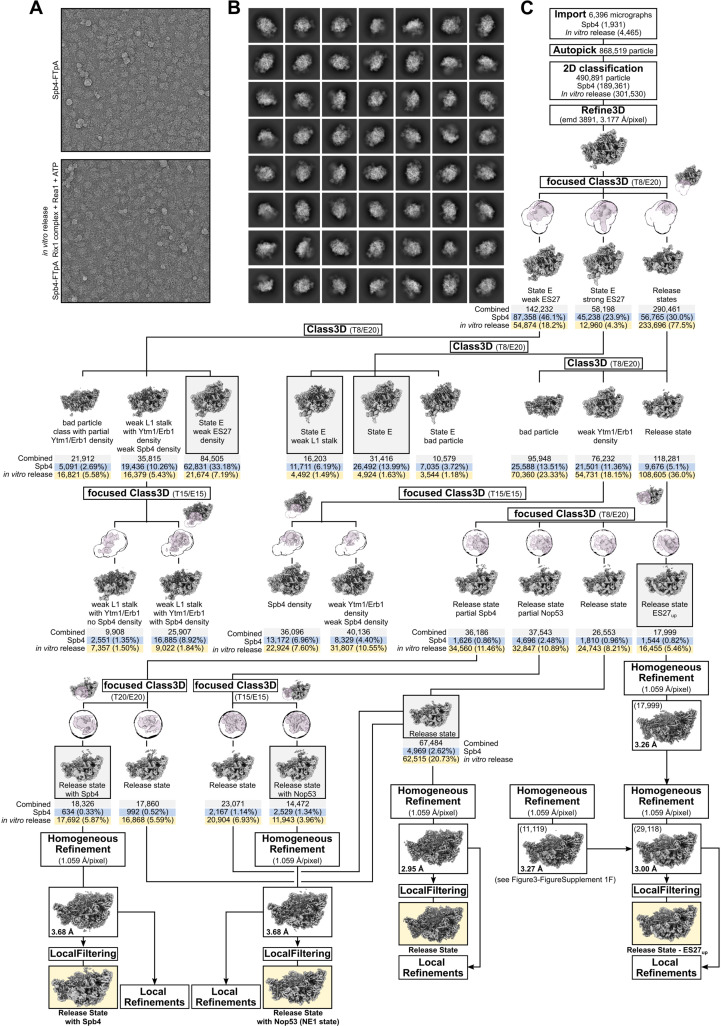
Sorting scheme of the combined Spb4 and in vitro release datasets. Representative micrographs of each dataset (**A**) and combined particle averages after 2D classification (**B**). (**C**) Sorting scheme of the combined dataset. Particle numbers of the combined and the individual dataset of each class are shown. Final volumes are highlighted and shown with their respective resolution. In case of the release state ES27up volume, particles from an identical class (see Figure 3—figure supplement 1F) were added to increase the resolution.

**Figure 4—figure supplement 5.**
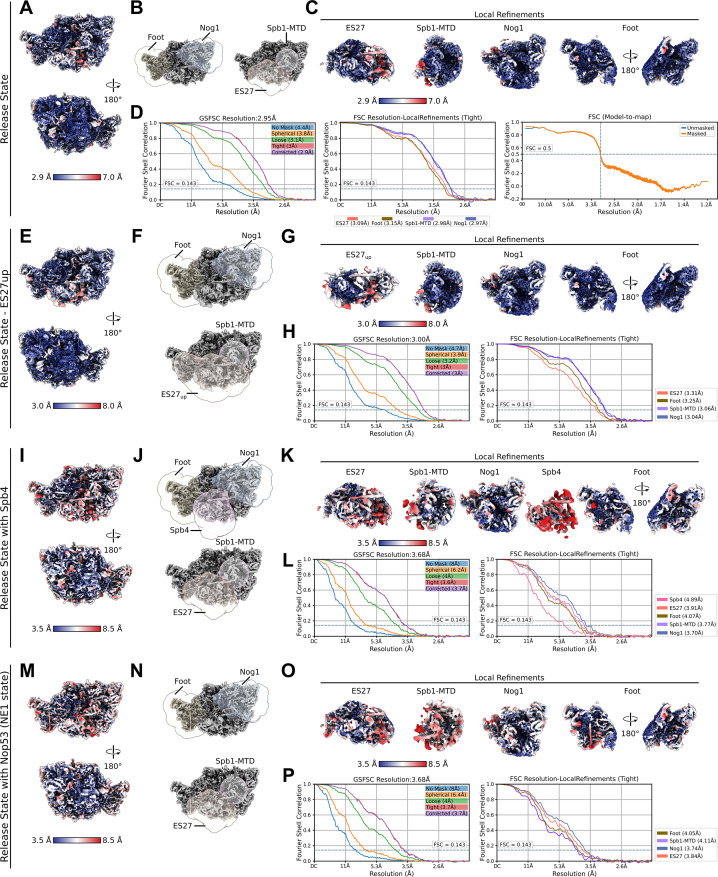
Local resolution and Fourier shell correlation (FSC) curves of the observed release states. Data for the release state (**A–D**), the release state – ES27up (**E–H**), the release state with Spb4 (**I–L**) and the NE1 state containing Nop53 (**M–P**) are depicted. Consensus cryo-EM maps coloured according to the local resolution (**A, E, I, M**), the different consensus cryo-EM maps superimposed with masks used for the local refinements (**B, F, J, N**) and the different local refinements coloured according to the local resolution (**C, G, K, O**) are displayed. The FSC curves for the consensus map (left) and the local refined maps (**D, H, L, P**) as well as the model-to-map-FSC curve for the release state (**D**) are shown.

**Figure 4—figure supplement 6.**
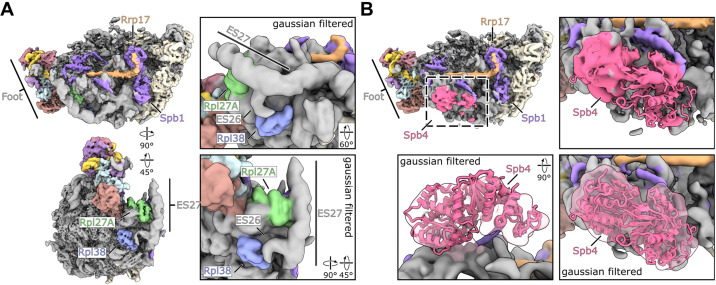
Cryo-EM maps of the release state with ES27up and the release state containing Spb4. (**A**) Coloured cryo-EM map of the release state with ES27 in an upward conformation shown in two orientations (left panels). Gaussian filtered map focussing on contact sites between Rpl27A-ES27 and Rpl38-ES26 (right panels). (**B**) Cryo-EM map overview of the Spb4 containing release state and close-up views focussing on the Spb4 density. Gaussian filtered maps are indicated. The model of the Spb4 helicase core (aa 2–398) was rigid body fitted into the density.

### Ytm1–Erb1 release promotes the dissociation of the RNA helicase Has1

While Rea1’s direct substrate Ytm1 is recruited to the pre-60S subunit via tight interactions with the C-terminal β-propeller domain of Erb1 near the bottom of the foot-structure (Kater et al., 2017; Thoms et al., 2016; Wegrecki et al., 2015), the RNA helicase Has1 is bound at a distant site over H16 at the top of the foot and is contacted by the meandering unstructured Erb1 N-terminal tail (Figure 4—figure supplement 2A; Kater et al., 2017; Zhou et al., 2019b). The nucleotide binding pocket of Has1 was found empty in our and a previously published pre-60S cryo-EM structure (Zhou et al., 2019b). To address whether upon addition of ATP the catalytic activity of Has1 might be activated during the observed pre-60S release, we performed the in vitro maturation assay with Spb4 particles derived from *HAS1*-HA-AID-depleted cells in presence of a catalytically inactive, non-viable *has1* K92A mutant impaired in nucleotide-binding (Figure 4—figure supplements 1D and 2B, left panel; Rocak et al., 2005). Upon incubation with Rea1, the Rix1-complex, and ATP, wild-type and mutant Has1 proteins were released with similar efficiency together with the Ytm1–Erb1 complex, demonstrating that the removal of all three factors occurs independently from Has1’s catalytic activity (Figure 4—figure supplement 2B, left panel; compare lanes 4 and 8). Furthermore, the Ytm1–Erb1 release could be induced also from particles in absence of any Has1 protein (Figure 4—figure supplement 2B, right panel; lane 12). Thus, the Rea1-dependent pre-60S release of the Ytm1–Erb1 complex occurs before and independently of Has1, and in turn results in displacement of the helicase, which is potentially pulled off by its interaction with the intertwined Erb1 tail.

### Cryo-EM analysis of in vitro matured Spb4-derived pre-60S ribosomal particles

To follow the conformational re-arrangements induced by Rea1/Rix1-complex on Spb4-derived pre-60S particles, we analysed the in vitro matured particles by single-particle cryo-EM. Initial classification of the untreated Spb4 sample, the in vitro control sample (Spb4 particles, Rea1/Rix1-complex, no ATP), and the in vitro release sample (Spb4 particles, Rea1/Rix1-complex, plus ATP) indicated a significant formation of in vitro matured pre-60S particles (44.1% of all particles), resembling structural state NE1 particles (Kater et al., 2020; Figure 4—figure supplement 3). In contrast, only 7.4 and 7.0% of such NE1-like particles, respectively, were seen in the untreated Spb4 and Spb4 in vitro control samples. To better compare the ratios and classes within the untreated Spb4 control and the in vitro release samples, and to avoid any sorting bias of the individual datasets, we decided to join the micrographs of both samples and perform uniform data processing (Figure 4—figure supplements 4 and 5). After extensive sorting, we identified six major classes, three state E-like states containing Ytm1–Erb1 and three released states similar to the described NE1 state (Figure 4B, Figure 4—figure supplements 4–6). The Ytm1–Erb1-containing state E-like states were mainly present in the untreated Spb4-FTpA sample (53.36%; only 10.31% in the in vitro release sample), whereas the release states strongly increased in the case of the in vitro release sample (32.06%; only 3.77% in the Spb4 control sample). We conclude that Rea1/Rix1-complex/ATP-induced remodelling of Spb4 particles in vitro resulted in maturation from E-like to distinct NE1-like states. All three release states have strong resemblance to the previously described NE1 state (obtained from Nop53 affinity purifications) but lack the foot-factor Nop53. However, we also find a minor particle class containing Nop53, which indicates that the attachment of Spb4 and Nop53 to pre-60S intermediates might not be strictly mutually exclusive (Figure 4—figure supplement 4).

Notably, all obtained release state particles still contain Rrp17 bound to the Spb1 MTD (Figure 4B and C), which is in line with Rrp17-FTpA purifications suggesting that the protein is still present on intermediates downstream of Spb4 (Figure 1D). In contrast, the Ytm1–Erb1 complex, the helicase Has1 and also Spb4 were no longer visible in the in vitro released states. Since Spb4 was our bait for specimen purification, it appears that Spb4 remained attached to the in vitro matured pre-60S particles in a flexible way and hence was not detectible by cryo-EM. Indeed, one of the released states showed a fuzzy density in the area where the Spb4 helicase core is located in the preceding state E, and rigid body fitting of the Spb4 helicase core clearly suggests that the poorly resolved density corresponds to Spb4 (Figure 4—figure supplement 6B). Moreover, the densities for several other assembly factors such as Loc1, Brx1, Ebp2, Noc2–Noc3, Nip7, Nop2 at the inter-subunit side, and Nop16 at the solvent-exposed side were not visible in the released NE1-like particles, revealing large-scale remodelling around the area that in state E is accessed and connected by the long N-terminal Erb1 tail (Figure 4C and D). Thus, Erb1 removal could result in destabilization of these factors, which may become invisible either by complete removal or flexible attachment to the in vitro matured pre-60S particles.

## Discussion

In this study, we provided functional insights into key restructuring events occurring on late nucleolar 60S subunit precursors preceding their transition to the nucleoplasmic compartment (Figure 4E). By combining biochemical and structural cryo-EM analyses, we unveiled how induction of the ATP-dependent release of the Ytm1–Erb1 heterodimer by the AAA^+^-ATPase Rea1 results in large-scale remodelling of pre-60S particles isolated via the RNA helicase Spb4. Such in vitro matured particles, from which a network of assembly factors appeared dismantled, with subtle variations, resembled structural state NE1, while largely lacking assembly factor Nop53. NE1 is the earliest state obtained upon purification of native pre-60S particles isolated via Nop53, which can only join upon release of Erb1 due to overlapping binding sites at the pre-60S foot structure (Kater et al., 2020). While our biochemical data show that the removal of RNA helicase Has1 occurs together with Ytm1–Erb1, they do not suggest the complete release of further assembly factors coupled to this step. In contrast, our single-particle cryo-EM analyses clearly revealed a strong enrichment of NE1-like particle classes in the in vitro release sample, which implicates the synchronized disassembly or at least destabilization of numerous additional assembly factors. The initial release of Ytm1–Erb1 and Has1 may therefore result in pre-rRNA re-arrangement and assembly factor destabilization, which would in consequence be too flexible to be resolved by cryo-EM or become unstably attached and potentially dissociate during cryo-EM sample preparation. Indeed, this is plausible considering the intertwined nature of the Erb1 N-terminus, which, by forming various rRNA and protein contacts, may until its release stabilize nucleolar pre-60S particles. However, it remains open whether in vivo further factors and intermediary maturation steps are required to facilitate assembly factor disassembly and formation of NE1 particles upon Ytm1–Erb1 and Has1 release.

The ATP-binding pocket of Has1 was found empty in our and also a previous pre-60S cryo-EM structure (Zhou et al., 2019b). As furthermore a Has1 Walker A mutant protein was efficiently released from the particle in our maturation assay, we conclude that Has1 dissociates in an ATP-independent manner from 60S precursors. This is in agreement with previous studies suggesting that the Has1 function on 90S pre-ribosomes requires its catalytic activity (Gnanasundram et al., 2019; Liang and Fournier, 2006), while the role of Has1 in pre-60S maturation could be ATP-independent and may depend only on its physical presence (Gnanasundram et al., 2019). Alternatively, since Has1 binds already to very early nucleolar pre-60S maturation intermediates, its catalytic activity may be required during its initial pre-60S recruitment. On such early nucleolar precursors, Has1 was implicated to play a role for contact formation between the 25S 5’ end and the 5.8S rRNA (Dembowski et al., 2013).

Our previous cryo-EM structures of nucleoplasmic pre-60S particles revealed how the pentameric Rix1-complex recruits the AAA^+^-ATPase Rea1 to these intermediates before nuclear export (Barrio-Garcia et al., 2016; Kater et al., 2020). The Rix1-complex is thereby essential to accurately position Rea1 for interaction and the subsequent release of its direct substrate Rsa4. Previous results (Bassler et al., 2010) and the increased efficiency in Ytm1–Erb1 release contributed by the Rix1-complex in our in vitro maturation assays suggest that the Rix1-complex recruits Rea1 to its earlier pre-60S substrate as well. Nevertheless, since structural insights into the more transient interaction of Rea1 with these nucleolar 60S precursors are missing, it remains unclear how the ATPase extracts Ytm1–Erb1 and how the Rix1-complex mediates this remodelling step.

The DEAD-box helicase Spb4 is one of around 20 RNA helicases involved in ribosome biogenesis that have been identified so far (Mitterer and Pertschy, 2022). While the approximate ribosomal maturation stages at which these RNA helicases are required have been determined, their molecular functions and precise substrates in most cases remain elusive. In addition, most RNA helicases could not be resolved on the so far existing cryo-EM structures of ribosomal precursors, likely due to high flexibility, unstable association or fast dissociation. Two exceptions in which ribosome-bound helicase structures could be determined are the DEAH helicase Dhr1 and the Ski2-like helicase Mtr4, which unwind the U3 snoRNA and RNA spacer substrates, respectively (Cheng et al., 2020; Du et al., 2020; Lau et al., 2021; Schuller et al., 2018; Singh et al., 2021). In contrast to such processive helicases, DEAD-box helicases are proposed to mediate RNA folding by non-processive local strand melting and annealing. In agreement with a most recently published work (Cruz et al., 2022), our cryo-EM structure of substrate-bound Spb4 suggests how such RNA strand displacement and re-arrangement by a DEAD-box helicase attached to its pre-ribosomal substrate is orchestrated. The existing general model on the function of DEAD-box helicases suggests that substrate RNA and ATP-binding together induce the closed active conformation of the catalytic RecA domains, whereas ATP-hydrolysis in the active site instantly triggers a re-opening of the catalytic domains going along with the release of the restructured RNA substrate.(Andersen et al., 2006; Bono et al., 2006; Mallam et al., 2012; Sengoku et al., 2006). Intriguingly, in contrast to this proposed mechanism, we found ADP instead of ATP in the nucleotide binding pocket of substrate RNA-bound Spb4 with its catalytic RecA domains in a closed conformation. Thus, while Spb4 likely has hydrolysed ATP at a previous stage, ATP-hydrolysis did not facilitate re-opening of the RecA-like domains and the helicase remained bound to ADP and its H62/H63 restructuring target. A reason for the unexpected Spb4 conformation might be the interactions of the first RecA domain with Rrp17, as well as the tight interaction of the CTD with the rRNA, which could potentially lock the rigid Spb4 core in its closed state. We therefore assume that current models on the function of DEAD-box helicases need to be extended since on complex substrates such as pre-ribosomes several additional factors might influence the state of the catalytic domains. In line with this, the DEAD-box helicase Dbp7 was also suggested to rely on an extensive interaction network for its function in rRNA folding (Aquino et al., 2021; Jaafar et al., 2021) and specific DEAD-box cofactors were shown to modulate the kinetics of the helicase core conformational cycle (Harms et al., 2014). Upon Spb4 dissociation, the H62/H63/H63a rRNA is then adopting a new stably folded and mature-like state as seen in cryo-EM structures of downstream pre-60S intermediates (Kater et al., 2020; Wu et al., 2016). What eventually triggers substrate release and pre-60S dissociation of Spb4 remains, however, unclear and open for future studies. As we could not recapitulate the H62/H63/H63a folding to a more mature state in our in vitro assays, the release or association of further assembly factors and ribosomal proteins may be required to facilitate this transition. While our manuscript was in revision, another study providing a highly similar cryo-EM structure of pre-60S bound Spb4 was published (Cruz et al., 2022). The authors report similar results on the function of Spb4 and its rRNA restructuring target and observe Spb4 in a post-catalytic state. However, they observed that the ATP-binding pocket of Spb4 is empty, whereas we clearly identified ADP in the active site. This difference may be a result of slightly different preparation conditions and indicates that after ATP hydrolysis the remaining ADP binds with low affinity.

Two recent studies reported that the human Spb4 homolog DDX55 is strongly upregulated in various lung cancers and hepatocellular carcinoma (HCC) tissues promoting HCC cell proliferation and migration (Cui et al., 2021; Yu et al., 2022). Thus, the obtained insights into the Spb4 function for ribosome biogenesis could be of relevance for medical studies and treatment of such cancer types. Furthermore, our study demonstrated the great potential of structural cryo-EM analyses of in vitro matured pre-ribosomal intermediates, which will be a promising approach to unveil further restructuring and maturation events on evolving ribosomal precursors.

## Materials and methods

### Yeast strains and plasmids

*Saccharomyces cerevisiae* strains used in this study are listed in Supplementary file 1 and are derived from the W303 background (Thomas and Rothstein, 1989). Strains were constructed using established gene disruption and genomic tagging methods (Janke et al., 2004; Longtine et al., 1998). For yeast two-hybrid analyses, the reporter strain PJ69-4A was used (James et al., 1996). Plasmids used in this study are listed in Supplementary file 2 and were constructed using standard DNA cloning techniques and verified by sequencing.

### Growth analyses of yeast mutant strains

To investigate growth phenotypes of plasmid-derived *spb4* mutant alleles, *LEU2* plasmids carrying the respective constructs were transformed into a *SPB4* shuffle (*spb4*Δ YCplac33-P*_SPB4_-SPB4*) strain. Growth phenotypes of the mutant alleles were analysed on plates containing 1 g/l 5-fluoroorotic acid (5-FOA) (Apollo Scientific, Cat# PC4054) to select for cells that have lost the wild-type *SPB4*-containing *URA3* plasmid. To analyse growth phenotypes of plasmid-derived *rrp17* mutant alleles, a *RRP17*-HA-AID strain was transformed with plasmids carrying respective constructs and growth assessed upon Rrp17-HA-AID depletion on plates containing 2 mM auxin (3-indoleacetic acid) (Sigma-Aldrich, Cat# I2886). Genetic experiments were performed at least in two biological replicates.

### Yeast two-hybrid analyses

Plasmids expressing the Spb4 bait protein, fused to the *GAL4* DNA-binding domain (GAL4-BD), and the Rrp17 prey protein constructs, fused to the *GAL4* activation domain (GAL4-AD), were co-transformed into the reporter strain PJ69-4A (James et al., 1996). Yeast two-hybrid interactions were documented by spotting representative transformants in tenfold serial dilution steps on SDC-Leu-Trp and SDC-Leu-Trp-His (*HIS3* reporter gene) plates.

### Over-expression and affinity purification of yeast proteins

An N-terminal protein A-TEV-Cbp-HA (TAP-HA)-*REA1* fusion was over-expressed from a centromeric plasmid under the control of the galactose-inducible *GAL1-10* promoter. Cells were grown in synthetic galactose complete medium lacking leucine (SGC-Leu) to an OD_600_ value of around 1.5. The medium was supplemented with 1.2% galactose, 1.2% bacto peptone, and 0.6% yeast extract (final concentrations) and cells further grown to an OD_600_ value of ~6. Cells were harvested and flash-frozen in liquid nitrogen and stored at –20°C. Cells were lysed in lysis buffer containing 25  mM HEPES (pH 7.5), 250  mM NaCl, 10  mM MgCl_2_, 10  mM KCl, 5% glycerol, 0.01% NP-40, 1  mM DTT, 0.5  mM phenylmethylsulfonyl fluoride (PMSF), and 1× SIGMAFAST protease inhibitor (Sigma-Aldrich, Cat# S8830), by shaking in a bead beater (Fritsch) in the presence of glass beads. The lysate was cleared by consecutive centrifugation at 5000 and 18,000  rpm and incubated with IgG Sepharose 6 Fast Flow beads (GE Healthcare; Cat# 17096902) on a rotating wheel for 120  min at 4°C. Beads were extensively washed with lysis buffer (without protease inhibitors) and subsequently eluted through TEV cleavage at 16°C for 150  min. A final concentration of 2 mM CaCl_2_ was added to the protein eluate prior to incubation with Calmodulin agarose beads (G-Biosciences; Cat# 786–282) for 90 min at 4°C. After extensive washing with lysis buffer containing 2 mM CaCl_2_, the protein was eluted by three consecutive incubations with elution buffer containing 50 mM TRIS (pH 8.0), 100 mM NaCl, 5 mM KCl, 2 mM MgCl_2_, 8 mM EGTA, 0,01% NP-40 for 20 min at 4°C. EGTA-eluates were pooled and concentrated to a final concentration of ~2  mg/ml using an Amicon Ultra-4 cellulose centrifugal filter unit (Merck Millipore, Cat# UFC810024).

The pentameric Rix1_(2)_–Ipi3_(2)_–Ip1 complex, was over-expressed from 2 micron plasmids harbouring *RIX1*-TEV-proteinA, *IPI1*-Cbp, and untagged *IPI3* under control of the inducible *GAL1-10* promoter. Cells were grown in synthetic raffinose complete medium lacking leucine and tryptophane (SRC-Leu-Trp) to an OD_600_ value of around 1.5. Protein co-expression was induced by addition of an equal volume of double concentrated YPG medium for 6–7 hr. Cell harvesting and protein purification until elution with EGTA (in buffer without NP-40) was performed as described above. EGTA-eluates were concentrated and further purified by size exclusion chromatography (SEC) on a Superose 6 increase 10/300 GL column (GE Healthcare) equilibrated with lysis buffer. The respective fractions were pooled and concentrated to a final concentration of ~2  mg/ml using Amicon Ultra-4 cellulose filter units.

### Affinity purification of pre-ribosomal 60S particles

For two-step affinity purifications from yeast, respective Flag-TEV-proteinA (FTpA) tagged bait proteins were expressed under control of their native promoter. Yeast strains were grown in YPD (for endogenous bait proteins) or in synthetic dextrose complete (SDC) medium lacking leucine (for plasmid-derived bait proteins or maintenance of plasmid-derived mutants) at 30°C. Cells were harvested in the logarithmic growth phase, flash frozen in liquid nitrogen, and stored at –20°C. For depletion of auxin-inducible degron (AID) tagged proteins (as indicated in the figures), cultures were incubated in the presence of 0.5  mM auxin (3-indoleacetic acid, Sigma-Aldrich, Cat# I2886) for 120  min prior to harvesting the cells. Cell pellets were resuspended in lysis buffer containing 50  mM Tris-HCl (pH 7.5), 100  mM NaCl, 5  mM MgCl_2_, 0.05% NP-40, 1  mM DTT, supplemented with 1  mM PMSF, 1 × SIGMAFAST protease inhibitor (Sigma-Aldrich), and cells were ruptured by shaking in a bead beater (Fritsch) in the presence of glass beads. Lysates were cleared by two subsequent centrifugation steps at 4°C for 10 and 30  min at 5000 and 15,000  rpm, respectively. Supernatants were incubated with immunoglobulin G (IgG) Sepharose 6 Fast Flow beads (GE Healthcare) on a rotating wheel at 4°C for 90  min. Beads were transferred into Mobicol columns (MoBiTec) and, after washing with 15  ml of lysis buffer (per 2 l culture volume), cleavage with tobacco etch virus (TEV) protease was performed at 16°C for 120  min. In a second purification step, TEV eluates were incubated with Flag agarose beads (ANTI-FlagM2 Affinity Gel, Sigma-Aldrich, Cat# A2220) for 75  min at 4°C. After washing with 5  ml of lysis buffer, bound proteins were eluted with lysis buffer containing 300  µg/ml Flag peptide (Sigma-Aldrich, Cat# F3290) at 4°C for 45  min. Buffer lacking NP-40 was used for the last purification step in samples used for cryo-EM. Flag eluates were analysed by SDS-PAGE on 4–12% polyacrylamide gels (NuPAGE, Invitrogen, Cat# NP0322BOX) with colloidal Coomassie staining (Roti-blue, Carl Roth, Cat# A152.1) or by western blotting with antibodies, as indicated in the respective figures. Pre-60S affinity purifications were performed at least in two technical replicates.

### In vitro maturation assays with purified pre-ribosomal 60S particles

Yeast strains expressing chromosomal *SPB4*-FTpA (or plasmid derived YCplac111-*SPB4*-FTpA and YCplac111-*spb4_*R360A-FTpA for experiments in Figure 4—figure supplement 1B), *RRP17*-FTpA, or TAP-HA-*YTM1 RPL3*-Flag fusions under control of the respective endogenous promoters were grown in YPD or SDC-Leu medium and as indicated in the figures incubated in the presence of 0.5  mM auxin (3-indoleacetic acid, Sigma-Aldrich) for 120  min prior to harvesting the cells. Pre-ribosomal particles were purified via immunoglobulin G (IgG) Sepharose 6 Fast Flow beads (GE Healthcare) as described above. TEV-eluates were adjusted to a final volume of 0.5 ml and, as indicated in the figures, incubated with ATP (3 mM final concentration) (Sigma-Aldrich, Cat# A2383), purified Rea1 protein, and/or purified Rix1-complex on a rotating wheel for 45 min at room temperature. Subsequently, samples were chilled on ice and still bound material was re-isolated by incubation with anti-Flag agarose beads (ANTI-FlagM2 Affinity Gel, Sigma-Aldrich) for 60  min at 4°C and eluted and analysed as described above. The release assays with Spb4 particles were performed at least in three technical replicates.

### Western blotting

Western blot analysis was performed using the following antibodies: anti-Nog1 antibody (1:5000), anti-Nog2 antibody (1:20,000), anti-Arx1 antibody (1:2,000), anti-Nsa2 antibody (1:10,000), anti-Rlp24 antibody (1:2,000), provided by Micheline Fromont-Racine, anti-Nug1 antibody (1:10,000), anti-Bud20 antibody (1:5000), provided by Vikram Panse, anti-Ytm1 antibody (1:100), provided by John Woolford, anti-Has1 antibody (1:10,000) provided by Patrick Linder, anti-Ebp2 antibody (1:10,000), provided by Keiko Mizuta, anti-Noc3 antibody (1:1000), provided by Herbert Tschochner, anti-Rpl3 antibody (1:5,000), provided by Jonathan Warner, anti-Rsa4 antibody (1:10,000), provided by Miguel Remacha, anti-Arc1 antibody (1:5000, raised in the Hurt lab), horseradish-peroxidase-conjugated anti-Flag antibody (1:10,000; Sigma-Aldrich, Cat# A8592, RRID:AB_439702), horseradish-peroxidase-conjugated anti-HA antibody (1:5000; Roche, Cat# 12013819001; RRID:AB_390917), secondary horseradish-peroxidase-conjugated goat anti-rabbit antibody (1:2,000; Bio-Rad, Cat# 166-2408EDU, RRID:AB_11125345), and secondary horseradish-peroxidase-conjugated goat anti-mouse antibody (1:2,000; Bio-Rad, Cat# STAR105P, RRID:AB_323002).

### Cryo-EM sample preparation, analysis, and image processing

All purified samples were supplemented with 0.05% octaethylene glycol monododecyl ether. The samples (3.5 µl) were applied onto Quantifoil R3/3 Holey grids with 2 nm continuous carbon support, which were glow discharged with a Harrick PDC-32G plasma cleaner at 2.1 × 10^–1^ torr for 20 s. After a 45 s incubation time, the grids were blotted for 3 s and plunge frozen in liquid ethane using a Vitrobot Mark IV (FEI Company) operated at 4°C and 90% humidity.

Cryo-EM data were collected on a Titan Krios operated at 300 kV, equipped with a Gatan K2 direct electron detector. Images were acquired with a nominal pixel size of 1.059 Å under low-dose conditions, with a total dose of 42.4–46.8 e^-^/Å^2^ over 40 frames using EPU (Thermo Fisher). Gain corrected frames were dose-weighted, aligned, summed, and motion corrected with MotionCor2 (Zheng et al., 2017) and the contrast-transfer-function (CTF) parameters were estimated with CTFFIND4 and Gctf (Rohou and Grigorieff, 2015; Zhang, 2016).

The micrographs were manually inspected and low-quality micrographs were discarded. Particles were auto-picked without reference in Relion 3.1 (Zivanov et al., 2020; Zivanov et al., 2018) using the Laplacian-of-Gaussian blob detection. The particles were extracted and imported into CryoSPARC (Punjani et al., 2017) for iterative 2D classification and good 2D classes were selected and used for homogeneous refinement with the EMD-3891 map (lowpass filtered to 60 Å) as an initial reference (Kater et al., 2017). Particles were reimported into Relion 3.1 using the pyem (csparc2star.py) tool and 3× binned particles were extracted with 3.177 Å/pixel. All subsequent 3D refinements and 3D classifications steps were performed with Relion 3.1. For details see sorting schemes in Figure 3—figure supplement 1, Figure 4—figure supplement 3, and Figure 4—figure supplement 3. The particles of the final classes were extracted with a pixel size of 1.059 Å/pixel, and homogeneous refinements, local refinements, and local filtering were performed with CryoSPARC. Composite maps were generated with ChimeraX using the vop max command (Goddard et al., 2018; Pettersen et al., 2021).

For the uniform data processing and comparison of the untreated Spb4 control sample and the in vitro release sample, the micrographs of both dataset were joined in Relion 3.1 and processed as described above. Particle numbers of the Spb4 control dataset and the in vitro release dataset of each class were obtained through the unique optic groups identifiers of the datasets.

### Model building and refinement

The model PDB-6ELZ (Kater et al., 2017) was fitted into the density map of the State E particle and was used as an initial model. The overall better resolution of our cryo-EM map compared to the original reconstruction allowed us to improve the state E model (PDB-6ELZ). In particular, molecular models of Rrp17, Loc1, Noc2, and parts of Spb1 were built de novo in Coot (Emsley et al., 2010; Emsley and Cowtan, 2004). For the Spb4 model, swiss-model (Waterhouse et al., 2018) and alphaFold models (Jumper et al., 2021) were used as initial references. The RecA domains were rigid body fitted separately into the density and the C-terminus was built de novo. For the state D, we used our molecular model of the state E, the PDB entries 6C0F (Sanghai et al., 2018) and 6EM5 (Kater et al., 2017) as initial references. The model of state E without Rrp17 was generated with our model of state E as initial reference. The model was rigid body fitted into the cryo-EM map and badly resolved and/or missing parts including Rrp17 and the Spb1-MTD were deleted. For the release state particle, the state E model and the NE1 state model PDB-6YLX (Kater et al., 2020) were used as reference. All models were manually adjusted in Coot (Emsley et al., 2010; Emsley and Cowtan, 2004) and the final models were real-space refined using Phenix (Adams et al., 2010; Liebschner et al., 2019). Data collection and refinement statistics are summarized in Supplementary file 3.

ChimeraX was used to visualize molecular models and cryo-EM densities (Goddard et al., 2018; Pettersen et al., 2021). The model-based secondary structure predictions of rRNA H62/H63/H63a and the ITS2 were generated with Forna (Kerpedjiev et al., 2015).

## Data Availability

Atomic models reported in this study have been deposited in the Protein Data Bank (PDB) and can be retrieved using the following accession codes: 8BVN, 8BVU, 8BVV, 8BVY. Cryo-EM density maps have been deposited in the Electron Microscopy Data Bank (EMDB) and can be retrieved using the following accession codes: 16267, 16272, 16273, 16275, 16276, 16277, 16278. Yeast strains and plasmids are available from the corresponding authors upon request. The following datasets were generated: ThomsM MittererV BuschauerR BerninghausenO HurtE BeckmannR 2022State E pre-60S subunitRCSB Protein Data Bank8BVN ThomsM MittererV BuschauerR BerninghausenO HurtE BeckmannR 2023State D pre-60S subunitRCSB Protein Data Bank8BVU ThomsM MittererV BuschauerR BerninghausenO HurtE BeckmannR 2023StateE without Rrp17, pre-60S subunitRCSB Protein Data Bank8BVV ThomsM MittererV BuschauerR BerninghausenO HurtE BeckmannR 2022Release state pre-60S subunitRCSB Protein Data Bank8BVY ThomsM MittererV BuschauerR BerninghausenO HurtE BeckmannR 2022State E pre-60SElectron Microscopy Data BankEMD-16267 ThomsM MittererV BuschauerR BerninghausenO HurtE BeckmannR 2022State D pre-60SElectron Microscopy Data BankEMD-16272 ThomsM MittererV BuschauerR BerninghausenO HurtE BeckmannR 2022State E without Rrp17Electron Microscopy Data BankEMD-16273 ThomsM MittererV BuschauerR BerninghausenO HurtE BeckmannR 2022Release stateElectron Microscopy Data BankEMD-16275 ThomsM MittererV BuschauerR BerninghausenO HurtE BeckmannR 2022Release state ES27upElectron Microscopy Data BankEMD-16276 ThomsM MittererV BuschauerR BerninghausenO HurtE BeckmannR 2022Release state with Spb4Electron Microscopy Data BankEMD-16277 ThomsM MittererV BuschauerR BerninghausenO HurtE BeckmannR 2022Release state without Nop53Electron Microscopy Data BankEMD-16278

## Additional files

Supplementary file 1. *Saccharomyces cerevisiae* strains used in this study.

Supplementary file 2. Plasmids used in this study.

Supplementary file 3. Data collection and refinement statistics.

MDAR checklist

## References

[bib1] Adams PD, Afonine PV, Bunkóczi G, Chen VB, Davis IW, Echols N, Headd JJ, Hung LW, Kapral GJ, Grosse-Kunstleve RW, McCoy AJ, Moriarty NW, Oeffner R, Read RJ, Richardson DC, Richardson JS, Terwilliger TC, Zwart PH (2010). PHENIX: A comprehensive python-based system for macromolecular structure solution. Acta Crystallographica. Section D, Biological Crystallography.

[bib2] Ahmed YL, Thoms M, Mitterer V, Sinning I, Hurt E (2019). Crystal structures of rea1-MIDAS bound to its ribosome assembly factor ligands resembling integrin-ligand-type complexes. Nature Communications.

[bib3] Andersen CBF, Ballut L, Johansen JS, Chamieh H, Nielsen KH, Oliveira CLP, Pedersen JS, Séraphin B, Le Hir H, Andersen GR (2006). Structure of the exon junction core complex with a trapped DEAD-box atpase bound to RNA. Science.

[bib4] Aquino GRR, Hackert P, Krogh N, Pan KT, Jaafar M, Henras AK, Nielsen H, Urlaub H, Bohnsack KE, Bohnsack MT (2021). The RNA helicase dbp7 promotes domain V/VI compaction and stabilization of inter-domain interactions during early 60S assembly. Nature Communications.

[bib5] Barrio-Garcia C, Thoms M, Flemming D, Kater L, Berninghausen O, Baßler J, Beckmann R, Hurt E (2016). Architecture of the rix1-rea1 checkpoint machinery during pre-60S-ribosome remodeling. Nature Structural & Molecular Biology.

[bib6] Bassler J, Kallas M, Pertschy B, Ulbrich C, Thoms M, Hurt E (2010). The AAA-atpase rea1 drives removal of biogenesis factors during multiple stages of 60S ribosome assembly. Molecular Cell.

[bib7] Baßler J, Hurt E (2019). Eukaryotic ribosome assembly. Annual Review of Biochemistry.

[bib8] Bonnerot C, Pintard L, Lutfalla G (2003). Functional redundancy of spb1p and a snr52-dependent mechanism for the 2’-O-ribose methylation of a conserved rrna position in yeast. Molecular Cell.

[bib9] Bono F, Ebert J, Lorentzen E, Conti E (2006). The crystal structure of the exon junction complex reveals how it maintains a stable grip on mrna. Cell.

[bib10] Brüning L, Hackert P, Martin R, Davila Gallesio J, Aquino GRR, Urlaub H, Sloan KE, Bohnsack MT (2018). RNA helicases mediate structural transitions and compositional changes in pre-ribosomal complexes. Nature Communications.

[bib11] Burlacu E, Lackmann F, Aguilar L-C, Belikov S, van Nues R, Trahan C, Hector RD, Dominelli-Whiteley N, Cockroft SL, Wieslander L, Oeffinger M, Granneman S (2017). High-throughput RNA structure probing reveals critical folding events during early 60S ribosome assembly in yeast. Nature Communications.

[bib12] Chen Z, Suzuki H, Kobayashi Y, Wang AC, DiMaio F, Kawashima SA, Walz T, Kapoor TM (2018). Structural insights into mdn1, an essential AAA protein required for ribosome biogenesis. Cell.

[bib13] Cheng J, Lau B, La Venuta G, Ameismeier M, Berninghausen O, Hurt E, Beckmann R (2020). 90S pre-ribosome transformation into the primordial 40S subunit. Science.

[bib14] Choudhury P, Kretschmer J, Hackert P, Bohnsack KE, Bohnsack MT (2021). The dexd box ATPase DDX55 is recruited to domain IV of the 28S ribosomal RNA by its C-terminal region. RNA Biology.

[bib15] Cruz VE, Sekulski K, Peddada N, Sailer C, Balasubramanian S, Weirich CS, Stengel F, Erzberger JP (2022). Sequence-Specific remodeling of a topologically complex RNP substrate by spb4. Nature Structural & Molecular Biology.

[bib16] Cui Y, Hunt A, Li Z, Birkin E, Lane J, Ruge F, Jiang WG (2021). Lead DEAD/H box helicase biomarkers with the therapeutic potential identified by integrated bioinformatic approaches in lung cancer. Computational and Structural Biotechnology Journal.

[bib17] de la Cruz J, Kressler D, Rojo M, Tollervey D, Linder P (1998). Spb4p, an essential putative RNA helicase, is required for a late step in the assembly of 60S ribosomal subunits in *Saccharomyces cerevisiae*. RNA.

[bib18] de la Cruz J, Karbstein K, Woolford JL (2015). Functions of ribosomal proteins in assembly of eukaryotic ribosomes in vivo. Annual Review of Biochemistry.

[bib19] Dembowski JA, Kuo B, Woolford JL (2013). Has1 regulates consecutive maturation and processing steps for assembly of 60S ribosomal subunits. Nucleic Acids Research.

[bib20] Du Y, An W, Zhu X, Sun Q, Qi J, Ye K (2020). Cryo-EM structure of 90*s* small ribosomal subunit precursors in transition states. Science.

[bib21] Emsley P, Cowtan K (2004). Coot: model-building tools for molecular graphics. Acta Crystallographica. Section D, Biological Crystallography.

[bib22] Emsley P, Lohkamp B, Scott WG, Cowtan K (2010). Features and development of coot. Acta Crystallographica. Section D, Biological Crystallography.

[bib23] García-Gómez JJ, Lebaron S, Froment C, Monsarrat B, Henry Y, de la Cruz J (2011). Dynamics of the putative RNA helicase spb4 during ribosome assembly in *Saccharomyces cerevisiae*. Molecular and Cellular Biology.

[bib24] Gasse L, Flemming D, Hurt E (2015). Coordinated ribosomal ITS2 RNA processing by the Las1 complex integrating endonuclease, polynucleotide kinase, and exonuclease activities. Molecular Cell.

[bib25] Gnanasundram SV, Kos-Braun IC, Koš M (2019). At least two molecules of the RNA helicase HAS1 are simultaneously present in pre-ribosomes during ribosome biogenesis. Nucleic Acids Research.

[bib26] Goddard TD, Huang CC, Meng EC, Pettersen EF, Couch GS, Morris JH, Ferrin TE (2018). UCSF chimerax: meeting modern challenges in visualization and analysis. Protein Science.

[bib27] Granneman S, Bernstein KA, Bleichert F, Baserga SJ (2006). Comprehensive mutational analysis of yeast DEXD/H box RNA helicases required for small ribosomal subunit synthesis. Molecular and Cellular Biology.

[bib28] Harms U, Andreou AZ, Gubaev A, Klostermeier D (2014). EIF4B, eif4g and RNA regulate eif4a activity in translation initiation by modulating the eif4a conformational cycle. Nucleic Acids Research.

[bib29] Jaafar M, Contreras J, Dominique C, Martín-Villanueva S, Capeyrou R, Vitali P, Rodríguez-Galán O, Velasco C, Humbert O, Watkins NJ, Villalobo E, Bohnsack KE, Bohnsack MT, Henry Y, Merhi RA, de la Cruz J, Henras AK (2021). Association of snr190 snorna chaperone with early pre-60S particles is regulated by the RNA helicase dbp7 in yeast. Nature Communications.

[bib30] James P, Halladay J, Craig EA (1996). Genomic libraries and a host strain designed for highly efficient two-hybrid selection in yeast. Genetics.

[bib31] Janke C, Magiera MM, Rathfelder N, Taxis C, Reber S, Maekawa H, Moreno-Borchart A, Doenges G, Schwob E, Schiebel E, Knop M (2004). A versatile toolbox for PCR-based tagging of yeast genes: New fluorescent proteins, more markers and promoter substitution cassettes. Yeast.

[bib32] Jumper J, Evans R, Pritzel A, Green T, Figurnov M, Ronneberger O, Tunyasuvunakool K, Bates R, Žídek A, Potapenko A, Bridgland A, Meyer C, Kohl SAA, Ballard AJ, Cowie A, Romera-Paredes B, Nikolov S, Jain R, Adler J, Back T, Petersen S, Reiman D, Clancy E, Zielinski M, Steinegger M, Pacholska M, Berghammer T, Bodenstein S, Silver D, Vinyals O, Senior AW, Kavukcuoglu K, Kohli P, Hassabis D (2021). Highly accurate protein structure prediction with alphafold. Nature.

[bib33] Kargas V, Castro-Hartmann P, Escudero-Urquijo N, Dent K, Hilcenko C, Sailer C, Zisser G, Marques-Carvalho MJ, Pellegrino S, Wawiórka L, Freund SM, Wagstaff JL, Andreeva A, Faille A, Chen E, Stengel F, Bergler H, Warren AJ (2019). Mechanism of completion of peptidyltransferase centre assembly in eukaryotes. eLife.

[bib34] Kater L, Thoms M, Barrio-Garcia C, Cheng J, Ismail S, Ahmed YL, Bange G, Kressler D, Berninghausen O, Sinning I, Hurt E, Beckmann R (2017). Visualizing the assembly pathway of nucleolar pre-60S ribosomes. Cell.

[bib35] Kater L, Mitterer V, Thoms M, Cheng J, Berninghausen O, Beckmann R, Hurt E (2020). Construction of the central protuberance and L1 stalk during 60S subunit biogenesis. Molecular Cell.

[bib36] Kerpedjiev P, Hammer S, Hofacker IL (2015). Forna (force-directed RNA): Simple and effective online RNA secondary structure diagrams. Bioinformatics.

[bib37] Klinge S, Woolford JL (2019). Ribosome assembly coming into focus. Nature Reviews. Molecular Cell Biology.

[bib38] Kressler D, Hurt E, Bassler J (2010). Driving ribosome assembly. Biochimica et Biophysica Acta.

[bib39] Kressler D, Hurt E, Baßler J (2017). A puzzle of life: crafting ribosomal subunits. Trends in Biochemical Sciences.

[bib40] Lapeyre B, Purushothaman SK (2004). Spb1p-directed formation of gm2922 in the ribosome catalytic center occurs at a late processing stage. Molecular Cell.

[bib41] Lau B, Cheng J, Flemming D, La Venuta G, Berninghausen O, Beckmann R, Hurt E (2021). Structure of the maturing 90S pre-ribosome in association with the RNA exosome. Molecular Cell.

[bib42] Leidig C, Thoms M, Holdermann I, Bradatsch B, Berninghausen O, Bange G, Sinning I, Hurt E, Beckmann R (2014). 60S ribosome biogenesis requires rotation of the 5S ribonucleoprotein particle. Nature Communications.

[bib43] Liang XH, Fournier MJ (2006). The helicase Has1p is required for snoRNA release from pre-rRNA. Molecular and Cellular Biology.

[bib44] Liebschner D, Afonine PV, Baker ML, Bunkóczi G, Chen VB, Croll TI, Hintze B, Hung LW, Jain S, McCoy AJ, Moriarty NW, Oeffner RD, Poon BK, Prisant MG, Read RJ, Richardson JS, Richardson DC, Sammito MD, Sobolev OV, Stockwell DH, Terwilliger TC, Urzhumtsev AG, Videau LL, Williams CJ, Adams PD (2019). Macromolecular structure determination using x-rays, neutrons and electrons: recent developments in phenix. Acta Crystallographica. Section D, Structural Biology.

[bib45] Longtine MS, McKenzie A, Demarini DJ, Shah NG, Wach A, Brachat A, Philippsen P, Pringle JR (1998). Additional modules for versatile and economical PCR-based gene deletion and modification in *Saccharomyces cerevisiae*. Yeast.

[bib46] Ma C, Wu S, Li N, Chen Y, Yan K, Li Z, Zheng L, Lei J, Woolford JL, Gao N (2017). Structural snapshot of cytoplasmic pre-60S ribosomal particles bound by NMD3, lsg1, TIF6 and Reh1. Nature Structural & Molecular Biology.

[bib47] Mallam AL, Del Campo M, Gilman B, Sidote DJ, Lambowitz AM (2012). Structural basis for RNA-duplex recognition and unwinding by the DEAD-box helicase Mss116p. Nature.

[bib48] Malyutin AG, Musalgaonkar S, Patchett S, Frank J, Johnson AW (2017). Nmd3 is a structural mimic of eIF5A, and activates the cpgtpase lsg1 during 60S ribosome biogenesis. The EMBO Journal.

[bib49] Matsuo Y, Granneman S, Thoms M, Manikas RG, Tollervey D, Hurt E (2014). Coupled GTPase and remodelling ATPase activities form a checkpoint for ribosome export. Nature.

[bib50] Mickolajczyk KJ, Olinares PDB, Chait BT, Liu S, Kapoor TM (2022). The Midas domain of AAA mechanoenzyme mdn1 forms catch bonds with two different substrates. eLife.

[bib51] Mitterer V, Pertschy B (2022). Rna folding and functions of RNA helicases in ribosome biogenesis. RNA Biology.

[bib52] Oeffinger M, Zenklusen D, Ferguson A, Wei KE, El Hage A, Tollervey D, Chait BT, Singer RH, Rout MP (2009). Rrp17p is a eukaryotic exonuclease required for 5’ end processing of pre-60S ribosomal RNA. Molecular Cell.

[bib53] Pettersen EF, Goddard TD, Huang CC, Meng EC, Couch GS, Croll TI, Morris JH, Ferrin TE (2021). UCSF chimerax: structure visualization for researchers, educators, and developers. Protein Science.

[bib54] Punjani A, Rubinstein JL, Fleet DJ, Brubaker MA (2017). CryoSPARC: Algorithms for rapid unsupervised cryo-EM structure determination. Nature Methods.

[bib55] Rocak S, Emery B, Tanner NK, Linder P (2005). Characterization of the ATPase and unwinding activities of the yeast DEAD-box protein Has1p and the analysis of the roles of the conserved motifs. Nucleic Acids Research.

[bib56] Rodríguez-Galán O, García-Gómez JJ, de la Cruz J (2013). Yeast and human RNA helicases involved in ribosome biogenesis: current status and perspectives. Biochimica et Biophysica Acta.

[bib57] Rohou A, Grigorieff N (2015). CTFFIND4: Fast and accurate defocus estimation from electron micrographs. Journal of Structural Biology.

[bib58] Sanghai ZA, Miller L, Molloy KR, Barandun J, Hunziker M, Chaker-Margot M, Wang J, Chait BT, Klinge S (2018). Modular assembly of the nucleolar pre-60S ribosomal subunit. Nature.

[bib59] Schuller JM, Falk S, Fromm L, Hurt E, Conti E (2018). Structure of the nuclear exosome captured on a maturing preribosome. Science.

[bib60] Sengoku T, Nureki O, Nakamura A, Kobayashi S, Yokoyama S (2006). Structural basis for RNA unwinding by the DEAD-box protein *Drosophila* vasa. Cell.

[bib61] Singh S, Vanden Broeck A, Miller L, Chaker-Margot M, Klinge S (2021). Nucleolar maturation of the human small subunit processome. Science.

[bib62] Sosnowski P, Urnavicius L, Boland A, Fagiewicz R, Busselez J, Papai G, Schmidt H (2018). The cryoEM structure of the *Saccharomyces cerevisiae* ribosome maturation factor rea1. eLife.

[bib63] Talkish J, Zhang J, Jakovljevic J, Horsey EW, Woolford JL (2012). Hierarchical recruitment into nascent ribosomes of assembly factors required for 27SB pre-rRNA processing in *Saccharomyces cerevisiae*. Nucleic Acids Research.

[bib64] Theissen B, Karow AR, Köhler J, Gubaev A, Klostermeier D (2008). Cooperative binding of ATP and RNA induces a closed conformation in a DEAD box RNA helicase. PNAS.

[bib65] Thomas BJ, Rothstein R (1989). Elevated recombination rates in transcriptionally active DNA. Cell.

[bib66] Thoms M, Thomson E, Baßler J, Gnädig M, Griesel S, Hurt E (2015). The exosome is recruited to RNA substrates through specific adaptor proteins. Cell.

[bib67] Thoms M, Ahmed YL, Maddi K, Hurt E, Sinning I (2016). Concerted removal of the erb1-ytm1 complex in ribosome biogenesis relies on an elaborate interface. Nucleic Acids Research.

[bib68] Ulbrich C, Diepholz M, Bassler J, Kressler D, Pertschy B, Galani K, Böttcher B, Hurt E (2009). Mechanochemical removal of ribosome biogenesis factors from nascent 60S ribosomal subunits. Cell.

[bib69] Waterhouse A, Bertoni M, Bienert S, Studer G, Tauriello G, Gumienny R, Heer FT, de Beer TAP, Rempfer C, Bordoli L, Lepore R, Schwede T (2018). SWISS-MODEL: Homology modelling of protein structures and complexes. Nucleic Acids Research.

[bib70] Wegrecki M, Rodríguez-Galán O, de la Cruz J, Bravo J (2015). The structure of erb1-ytm1 complex reveals the functional importance of a high-affinity binding between two β-propellers during the assembly of large ribosomal subunits in eukaryotes. Nucleic Acids Research.

[bib71] Wu S, Tutuncuoglu B, Yan K, Brown H, Zhang Y, Tan D, Gamalinda M, Yuan Y, Li Z, Jakovljevic J, Ma C, Lei J, Dong MQ, Woolford JL, Gao N (2016). Diverse roles of assembly factors revealed by structures of late nuclear pre-60S ribosomes. Nature.

[bib72] Yelland JN, Bravo JPK, Black JJ, Taylor DW, Johnson AW (2023). A single 2’-O-methylation of ribosomal RNA gates assembly of a functional ribosome. Nature Structural & Molecular Biology.

[bib73] Yu B, Zhou S, Long D, Ning Y, Yao H, Zhou E, Wang Y (2022). DDX55 promotes hepatocellular carcinoma progression by interacting with BRD4 and participating in exosome-mediated cell-cell communication. Cancer Science.

[bib74] Zhang K (2016). Gctf: real-time CTF determination and correction. Journal of Structural Biology.

[bib75] Zheng SQ, Palovcak E, Armache JP, Verba KA, Cheng Y, Agard DA (2017). MotionCor2: anisotropic correction of beam-induced motion for improved cryo-electron microscopy. Nature Methods.

[bib76] Zhou Y, Musalgaonkar S, Johnson AW, Taylor DW (2019a). Tightly-orchestrated rearrangements govern catalytic center assembly of the ribosome. Nature Communications.

[bib77] Zhou D, Zhu X, Zheng S, Tan D, Dong MQ, Ye K (2019b). Cryo-em structure of an early precursor of large ribosomal subunit reveals a half-assembled intermediate. Protein & Cell.

[bib78] Zivanov J, Nakane T, Forsberg BO, Kimanius D, Hagen WJ, Lindahl E, Scheres SH (2018). New tools for automated high-resolution cryo-EM structure determination in RELION-3. eLife.

[bib79] Zivanov J, Nakane T, Scheres SHW (2020). Estimation of high-order aberrations and anisotropic magnification from cryo-EM data sets in RELION-3.1. IUCrJ.

