# Supplementary figures and images for: Concurrent remodelling of nucleolar 60S subunit precursors by the Rea1 ATPase and Spb4 RNA helicase
Source: eLife. 2023 Mar 17;12:e84877. doi: 10.7554/eLife.84877 (PMC10154028; doi:10.7554/eLife.84877)

Figure 1A

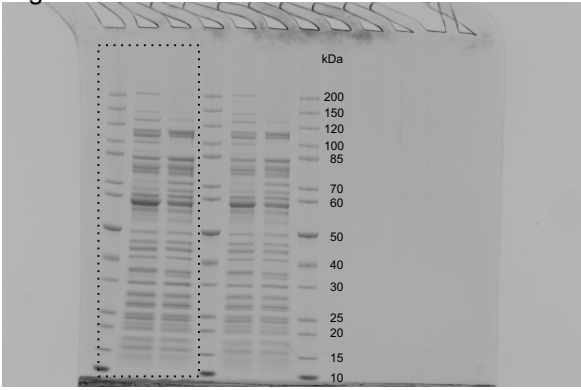

Figure 1D, left panel

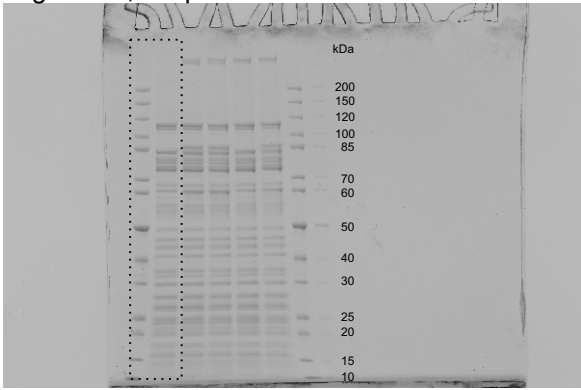

Figure 1D, right panel

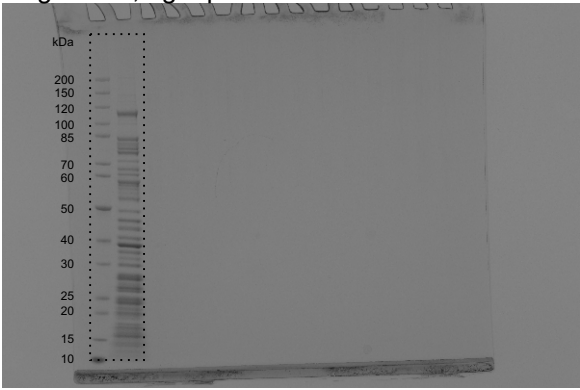

Supplement: Figure 1—source data 1. — Dashed boxes in the PDF indicate the respective areas shown in the figure. [file elife-84877-fig1-data1.zip › Figure1_Source_data/Figure1_Source_data_1.pdf]

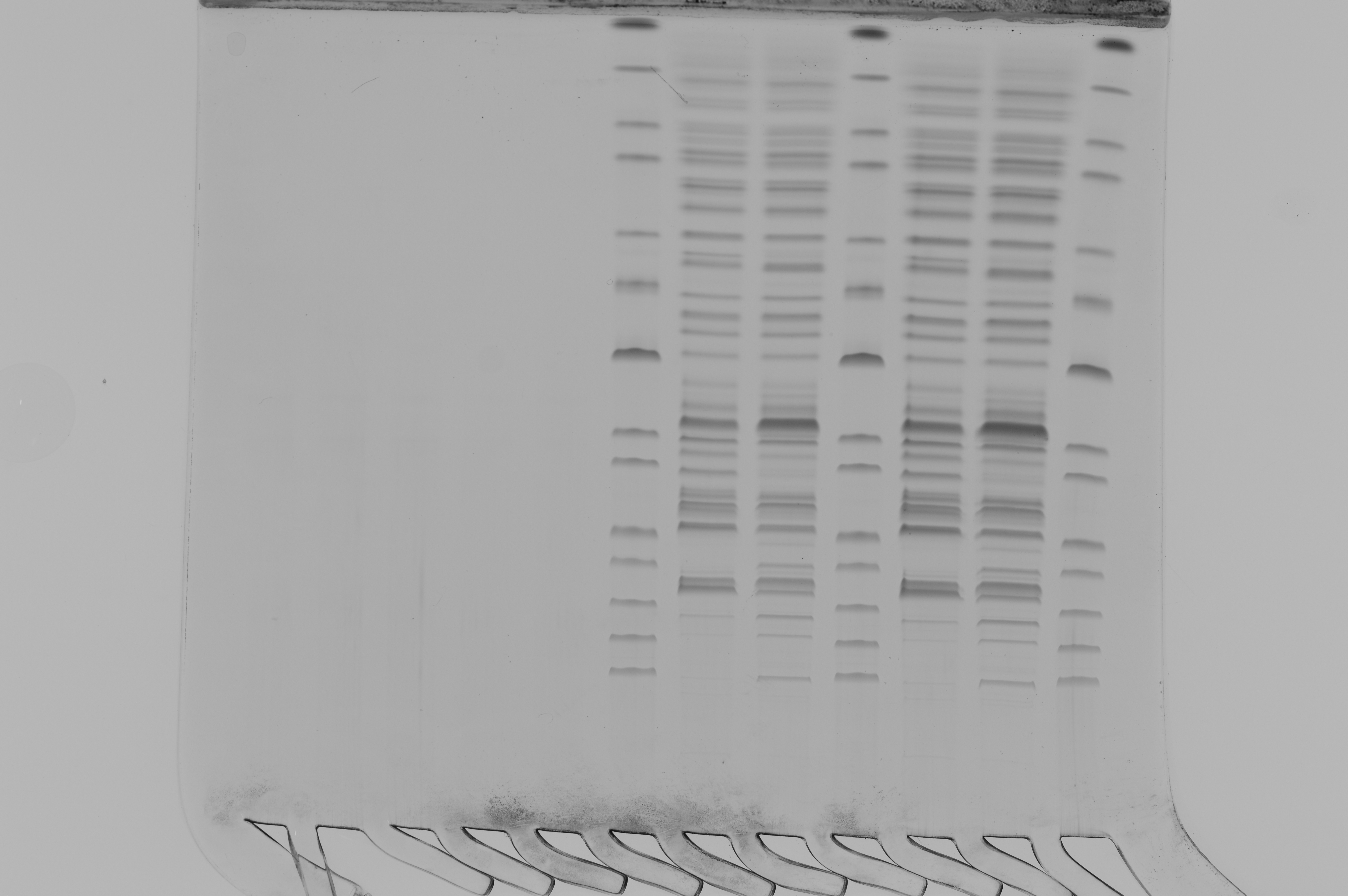

Supplement: Figure 1—source data 1. — Dashed boxes in the PDF indicate the respective areas shown in the figure. [file elife-84877-fig1-data1.zip › Figure1_Source_data/Figure1A_Coomassie.JPG]

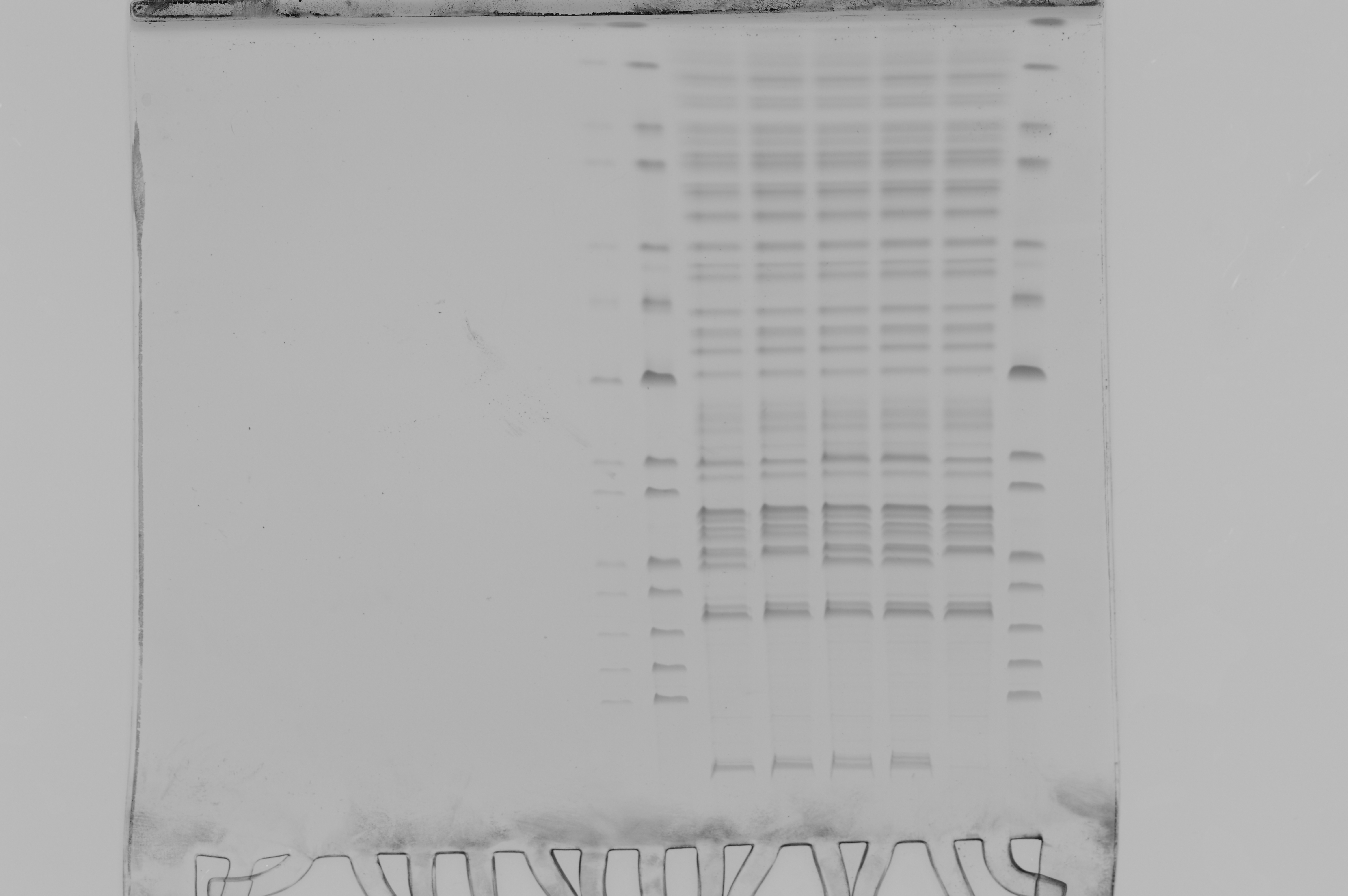

Supplement: Figure 1—source data 1. — Dashed boxes in the PDF indicate the respective areas shown in the figure. [file elife-84877-fig1-data1.zip › Figure1_Source_data/Figure1D_left_panel_Coomassie.JPG]

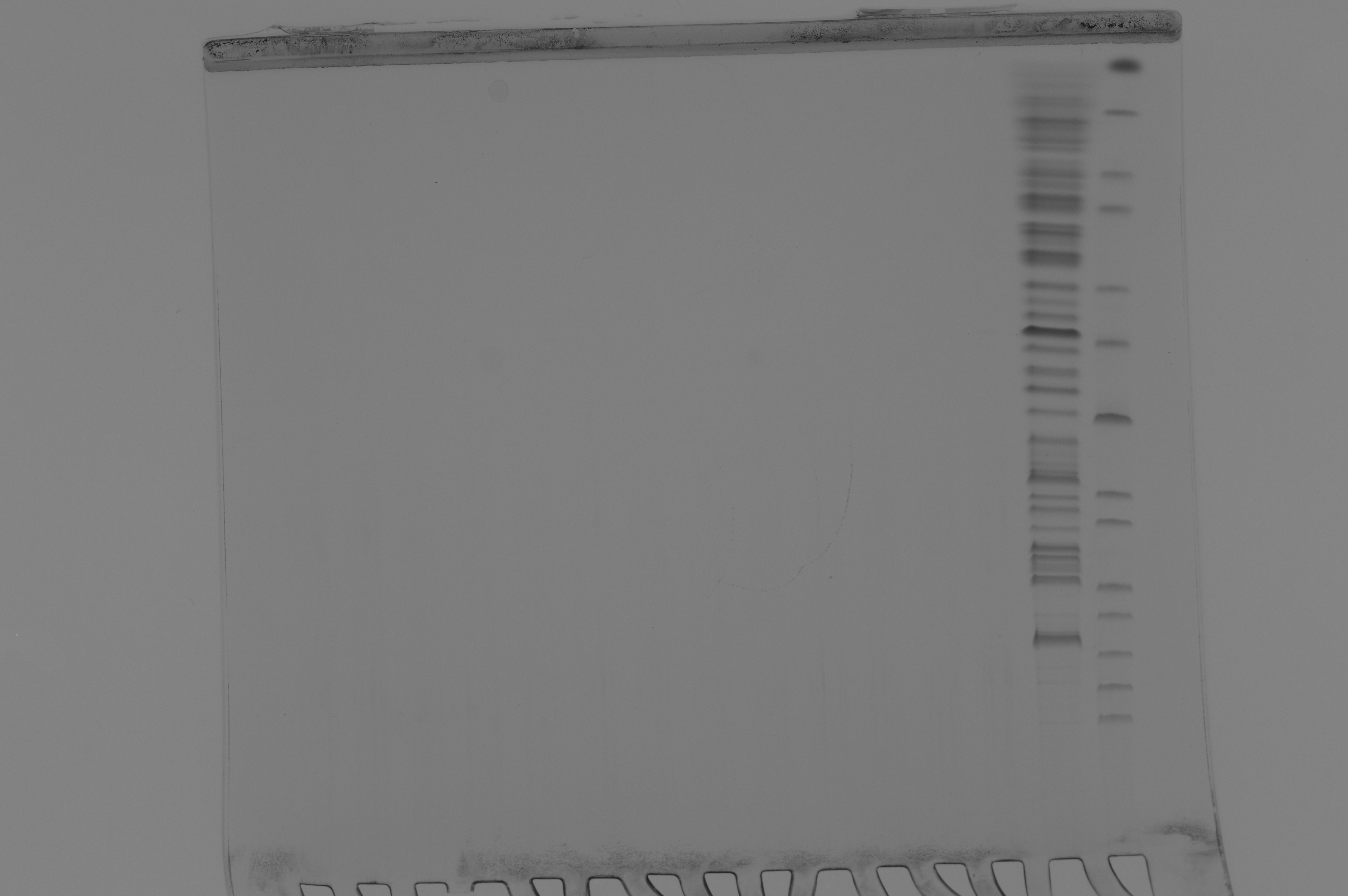

Supplement: Figure 1—source data 1. — Dashed boxes in the PDF indicate the respective areas shown in the figure. [file elife-84877-fig1-data1.zip › Figure1_Source_data/Figure1D_right_panel_Coomassie.JPG]

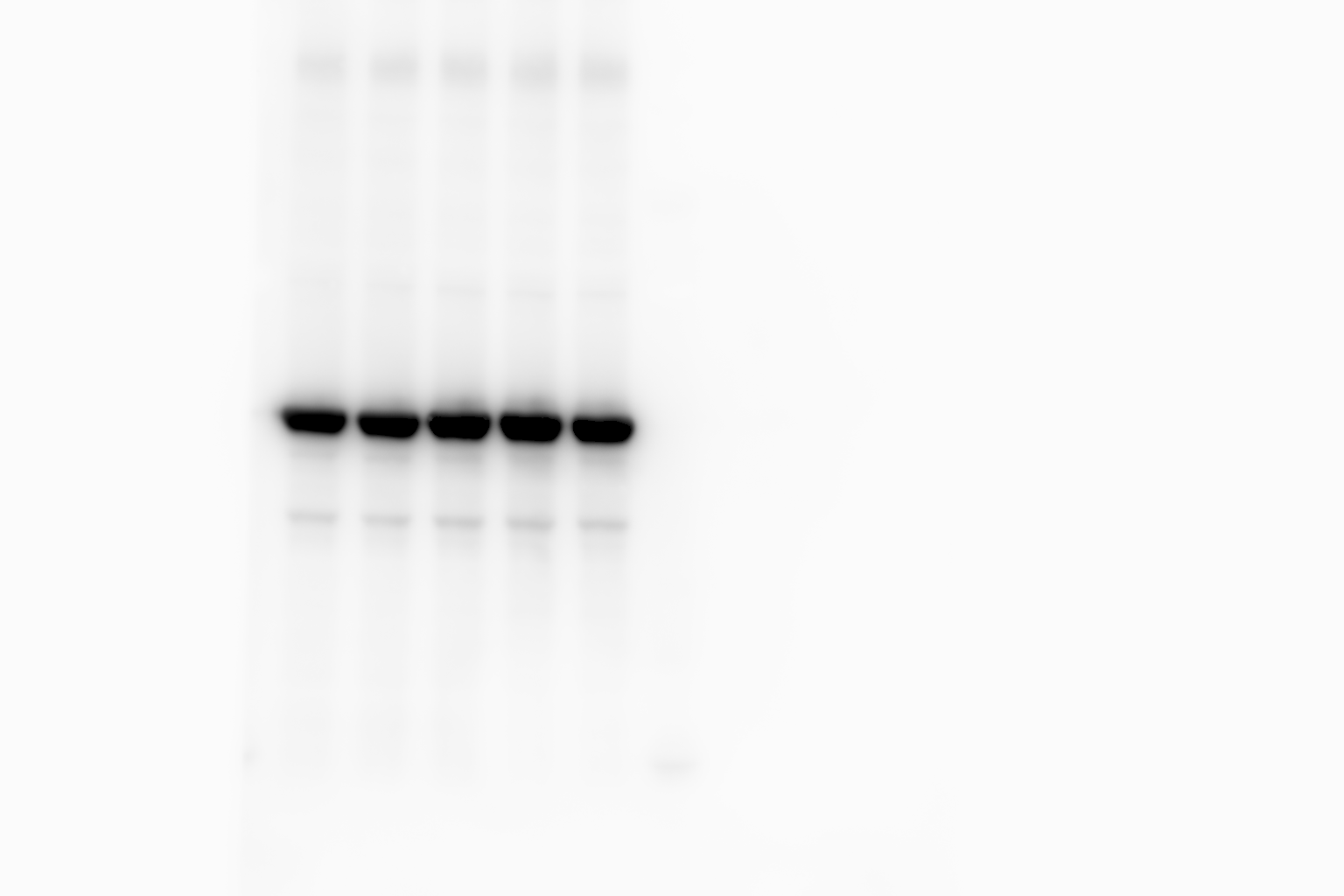

Supplement: Figure 1—figure supplement 1—source data 1. — Dashed boxes in the PDF indicate the respective areas shown in the figure. [file elife-84877-fig1-figsupp1-data1.zip › Figure1_Figure_Supplement1_Sourca_data1/Figure1_Figure_Supplement1A_lower_panel_Arc1.tif]

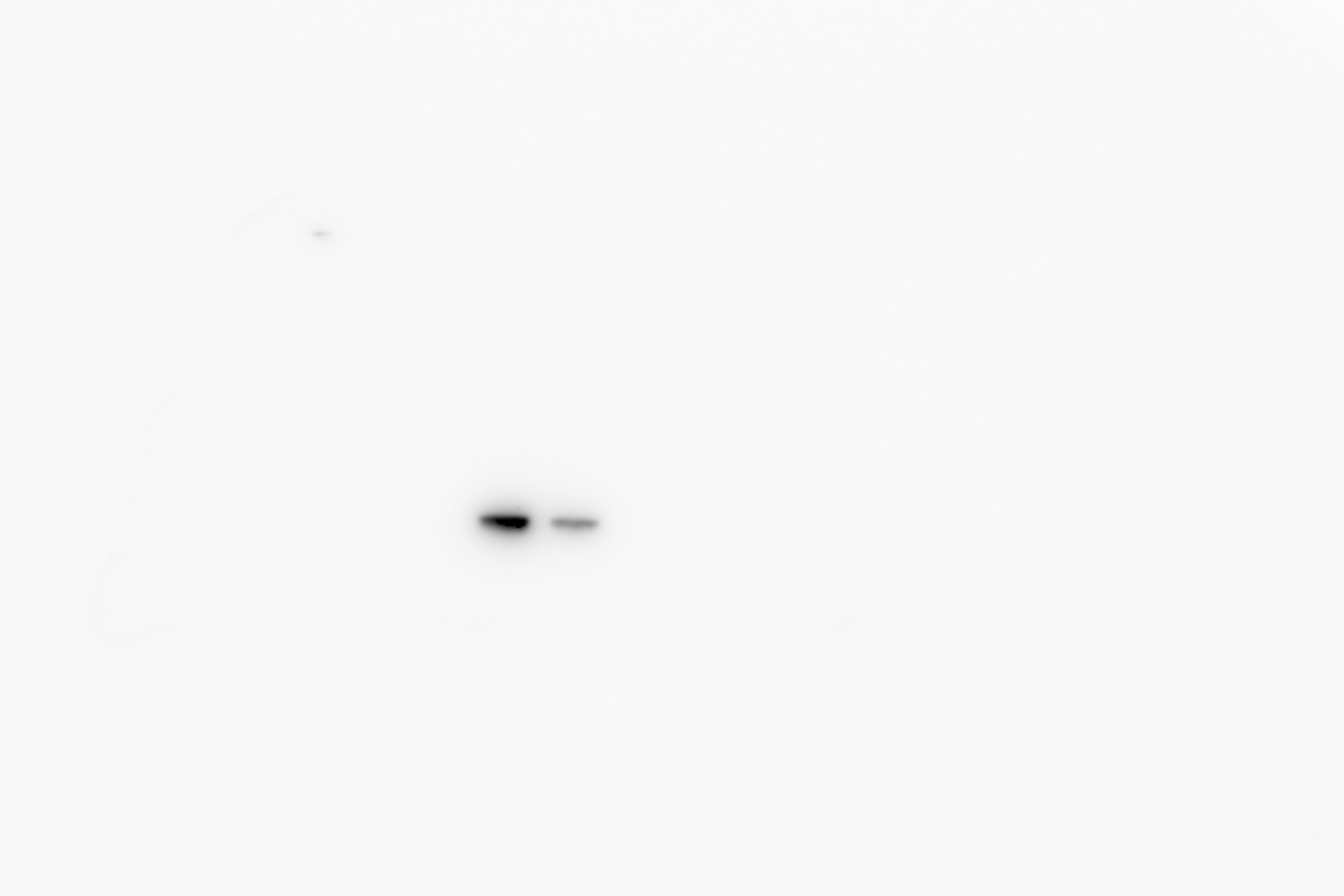

Supplement: Figure 1—figure supplement 1—source data 1. — Dashed boxes in the PDF indicate the respective areas shown in the figure. [file elife-84877-fig1-figsupp1-data1.zip › Figure1_Figure_Supplement1_Sourca_data1/Figure1_Figure_Supplement1A_lower_panel_HA.tif]

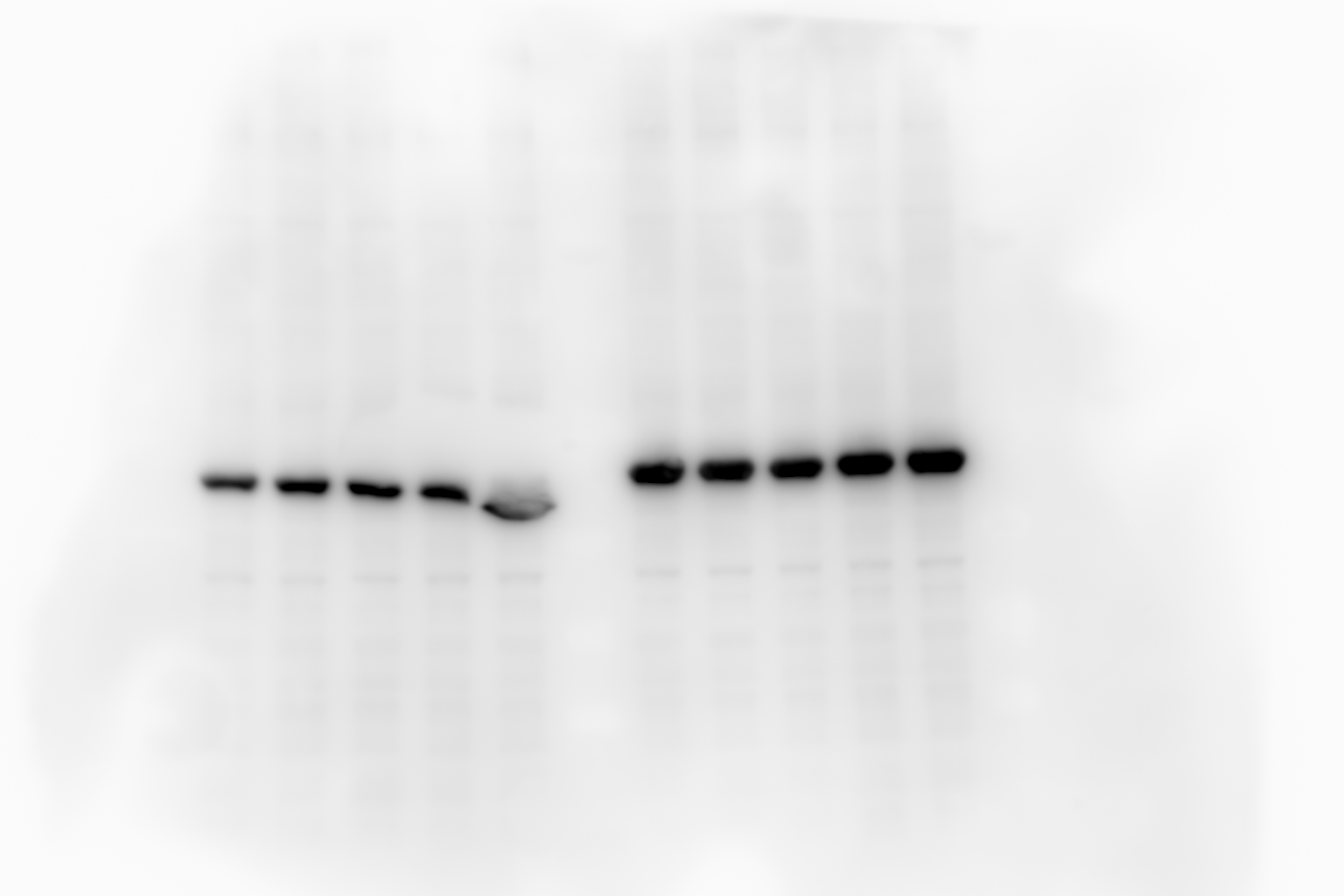

Supplement: Figure 1—figure supplement 1—source data 1. — Dashed boxes in the PDF indicate the respective areas shown in the figure. [file elife-84877-fig1-figsupp1-data1.zip › Figure1_Figure_Supplement1_Sourca_data1/Figure1_Figure_Supplement1A_upper_panel_Arc1.tif]

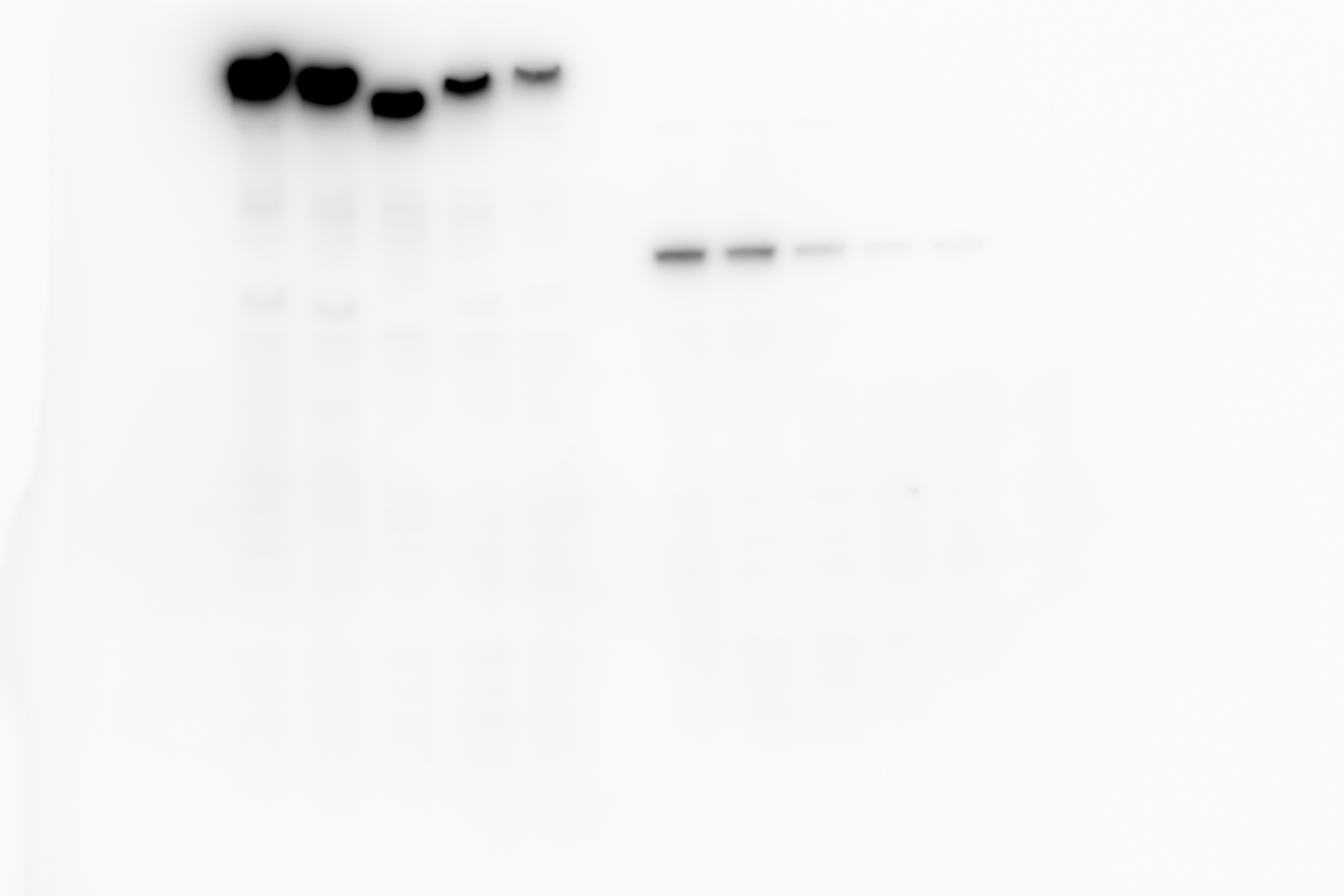

Supplement: Figure 1—figure supplement 1—source data 1. — Dashed boxes in the PDF indicate the respective areas shown in the figure. [file elife-84877-fig1-figsupp1-data1.zip › Figure1_Figure_Supplement1_Sourca_data1/Figure1_Figure_Supplement1A_upper_panel_HA.tif]

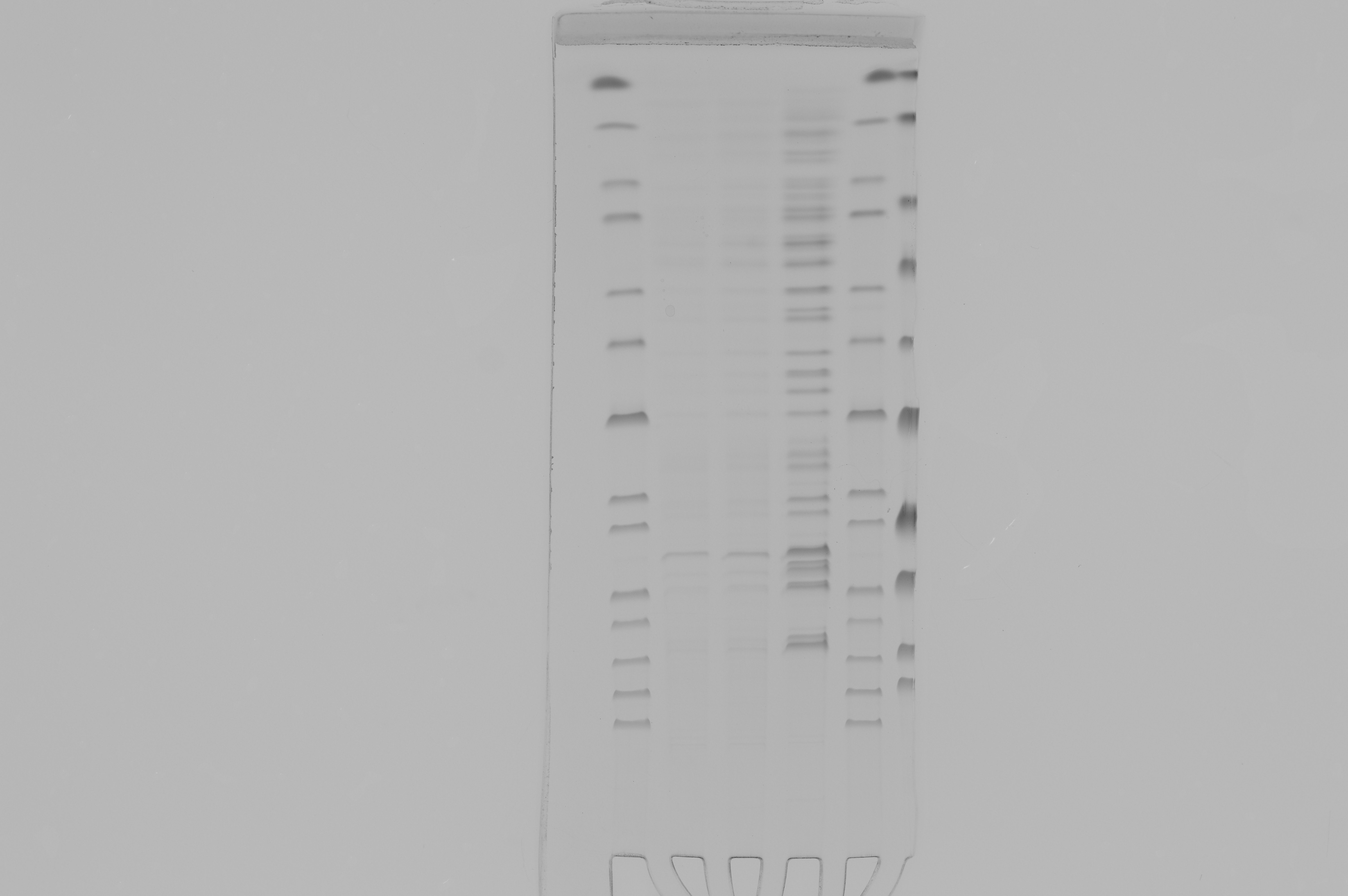

Supplement: Figure 1—figure supplement 1—source data 1. — Dashed boxes in the PDF indicate the respective areas shown in the figure. [file elife-84877-fig1-figsupp1-data1.zip › Figure1_Figure_Supplement1_Sourca_data1/Figure1_Figure_Supplement1C_Coomassie_left_panel.JPG]

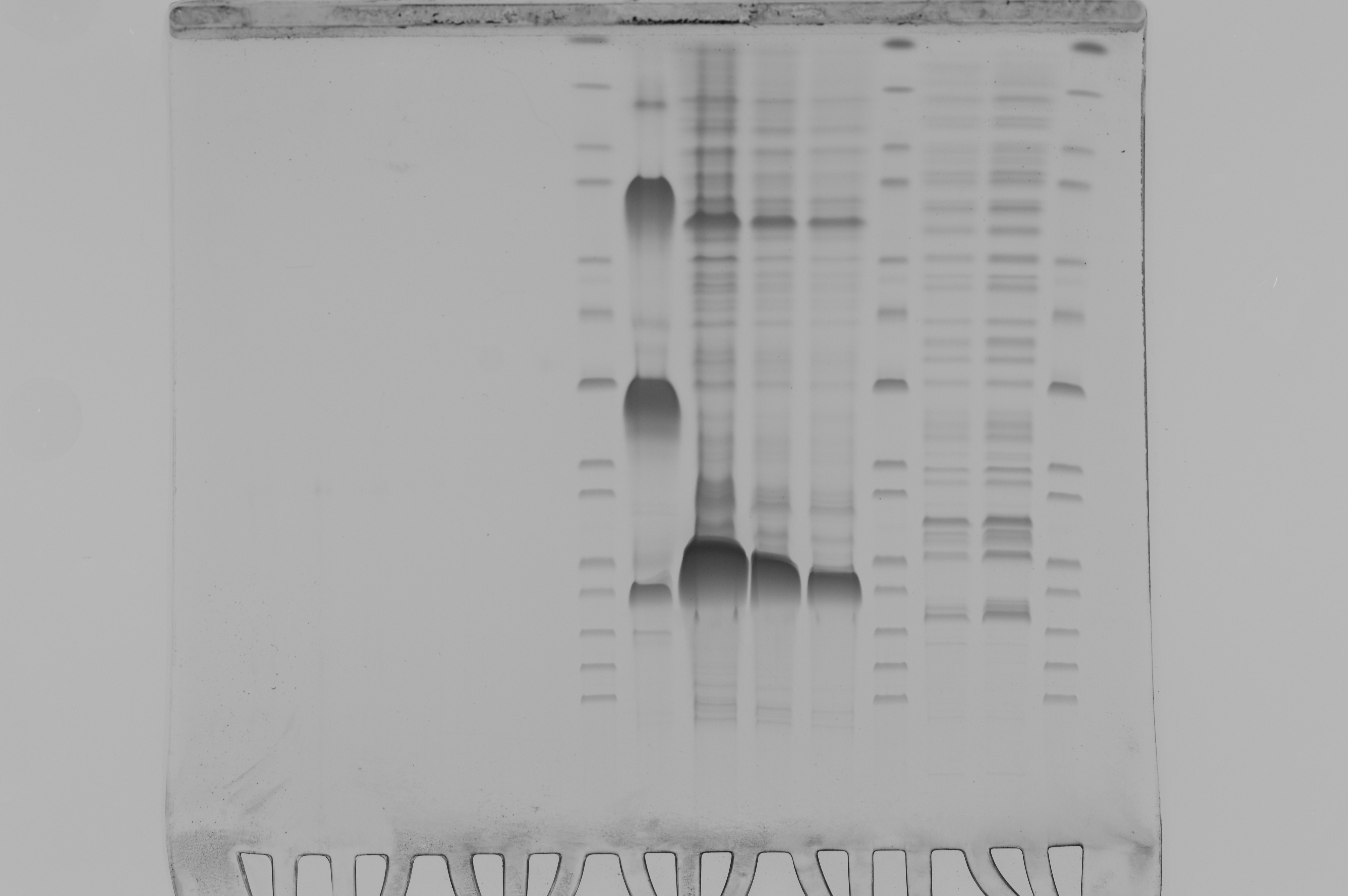

Supplement: Figure 1—figure supplement 1—source data 1. — Dashed boxes in the PDF indicate the respective areas shown in the figure. [file elife-84877-fig1-figsupp1-data1.zip › Figure1_Figure_Supplement1_Sourca_data1/Figure1_Figure_Supplement1C_Coomassie_right_panel.JPG]

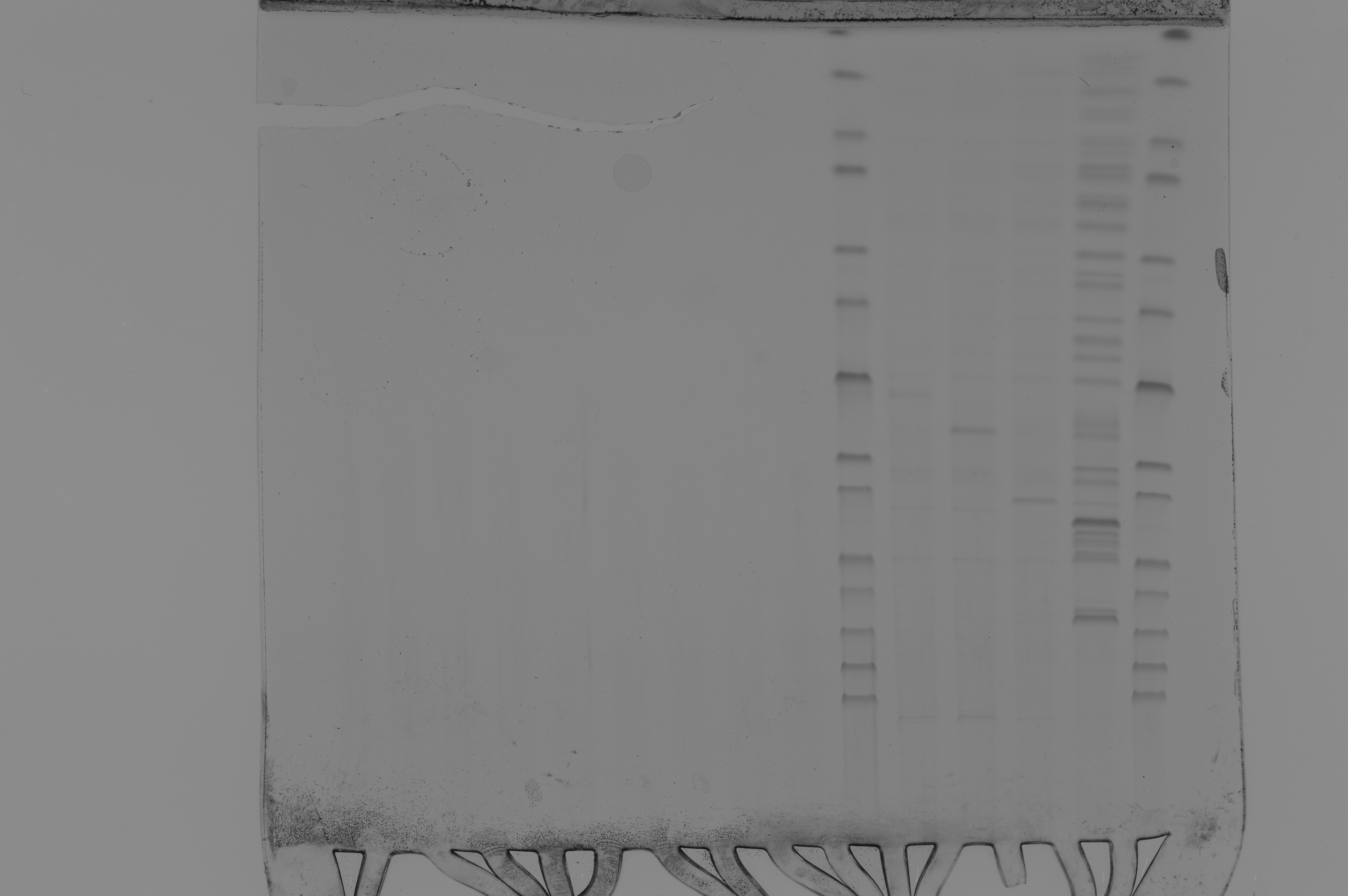

Supplement: Figure 1—figure supplement 1—source data 1. — Dashed boxes in the PDF indicate the respective areas shown in the figure. [file elife-84877-fig1-figsupp1-data1.zip › Figure1_Figure_Supplement1_Sourca_data1/Figure1_Figure_Supplement1D_Coomassie.JPG]

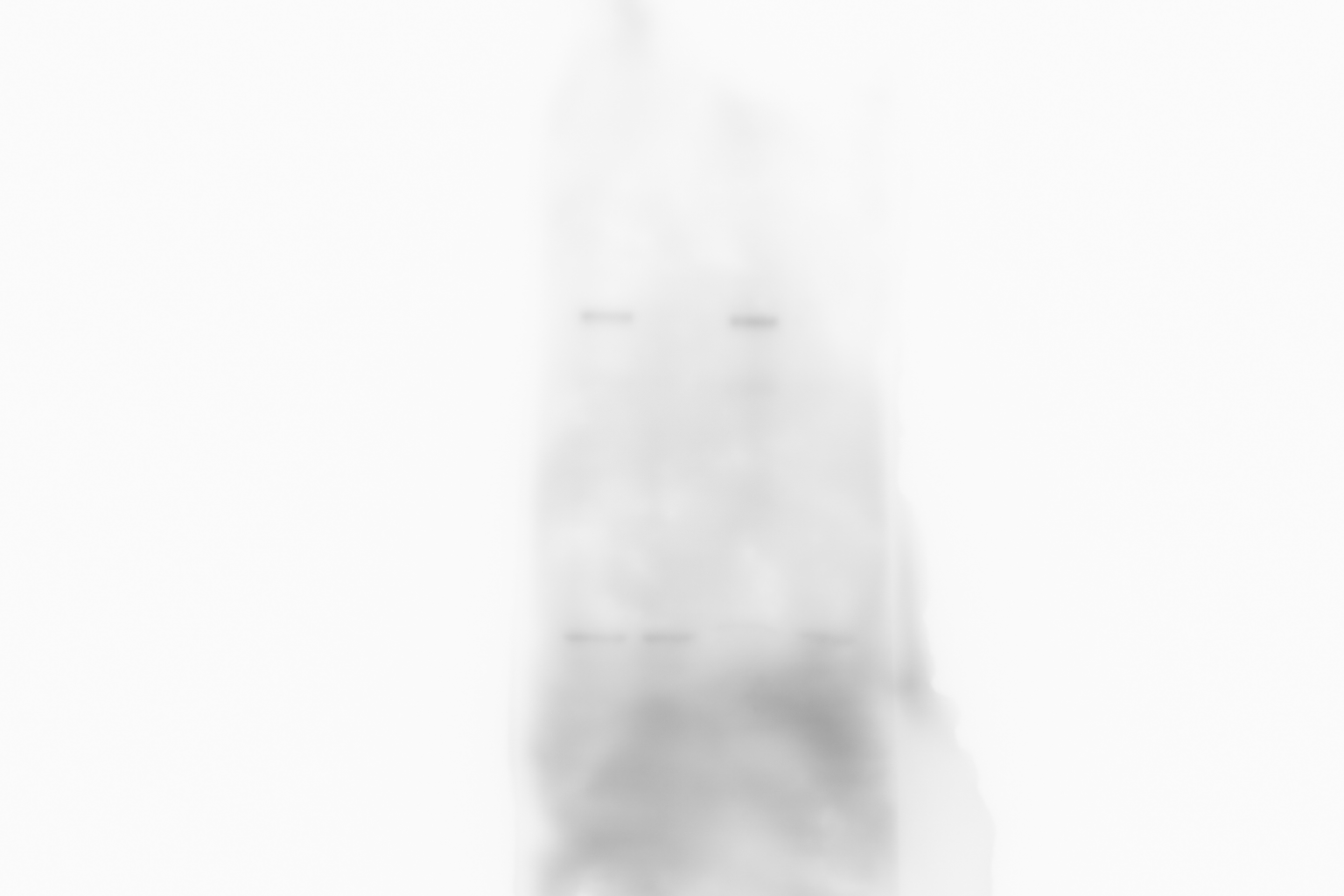

Supplement: Figure 2—source data 1. — Dashed boxes in the PDF indicate the respective areas shown in the figure. [file elife-84877-fig2-data1.zip › Figure2_Source_data_1/Figure2B_left_panel_Arx1.tif]

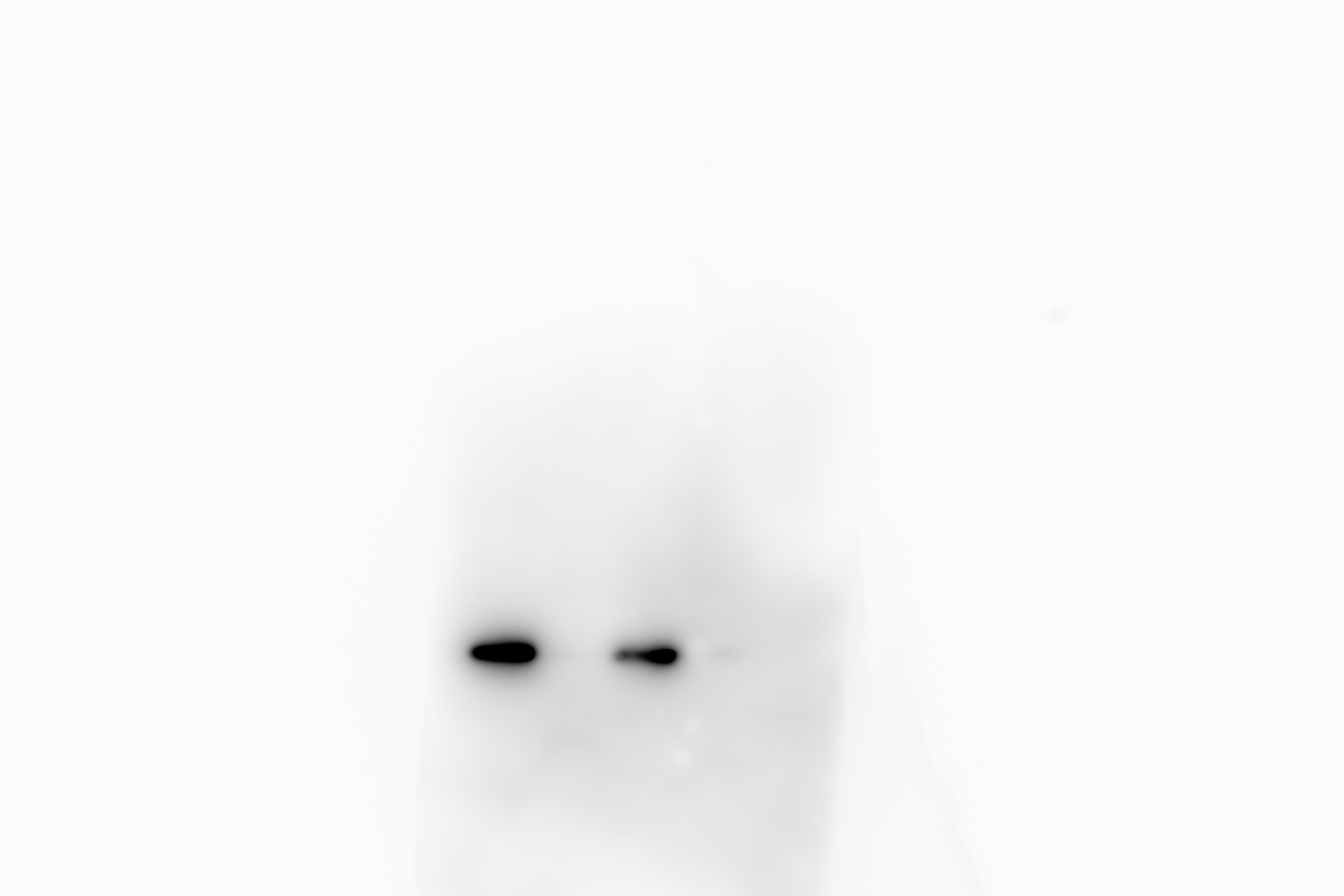

Supplement: Figure 2—source data 1. — Dashed boxes in the PDF indicate the respective areas shown in the figure. [file elife-84877-fig2-data1.zip › Figure2_Source_data_1/Figure2B_left_panel_Bud20.tif]

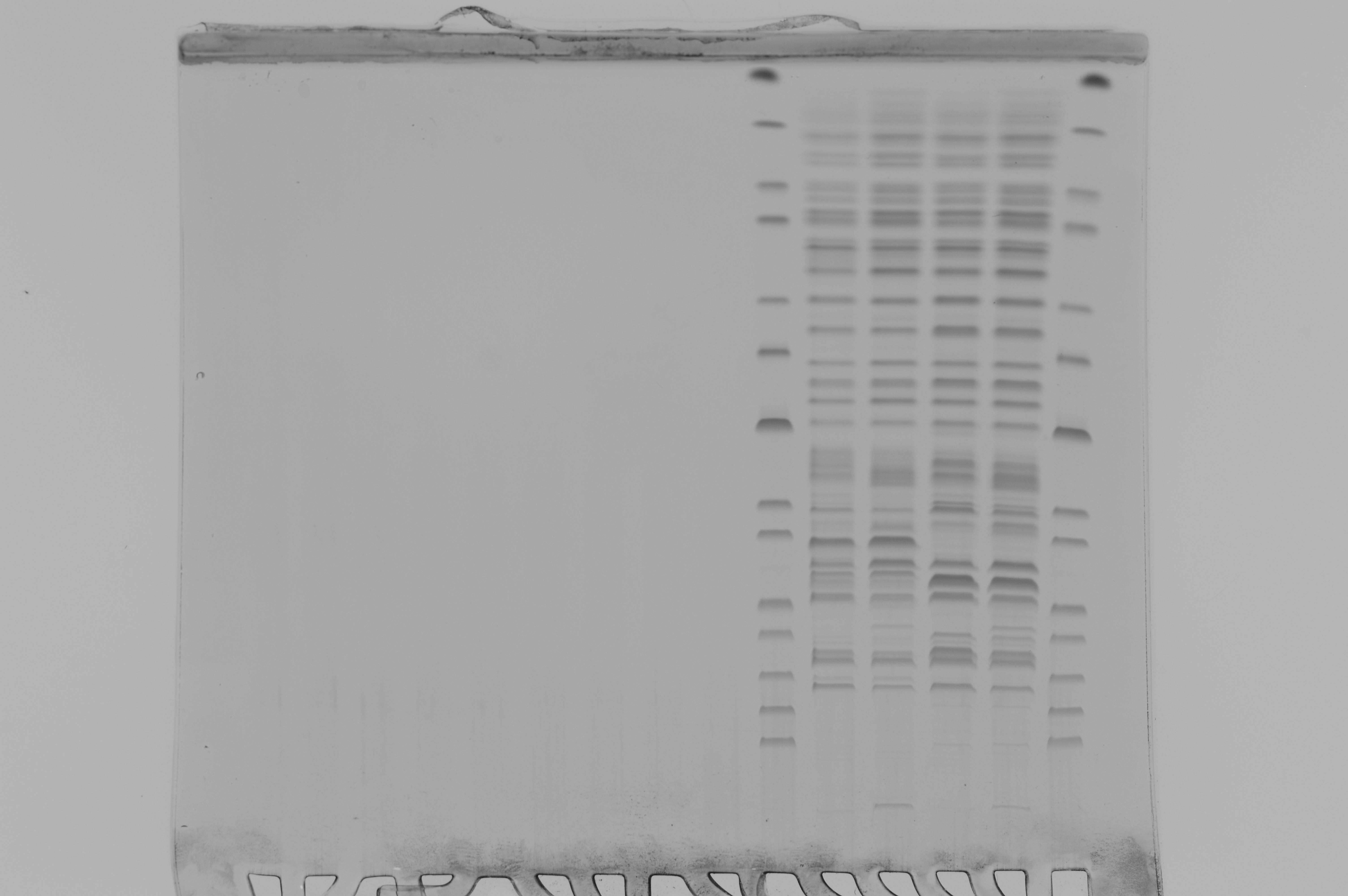

Supplement: Figure 2—source data 1. — Dashed boxes in the PDF indicate the respective areas shown in the figure. [file elife-84877-fig2-data1.zip › Figure2_Source_data_1/Figure2B_left_panel_Coomassie.JPG]

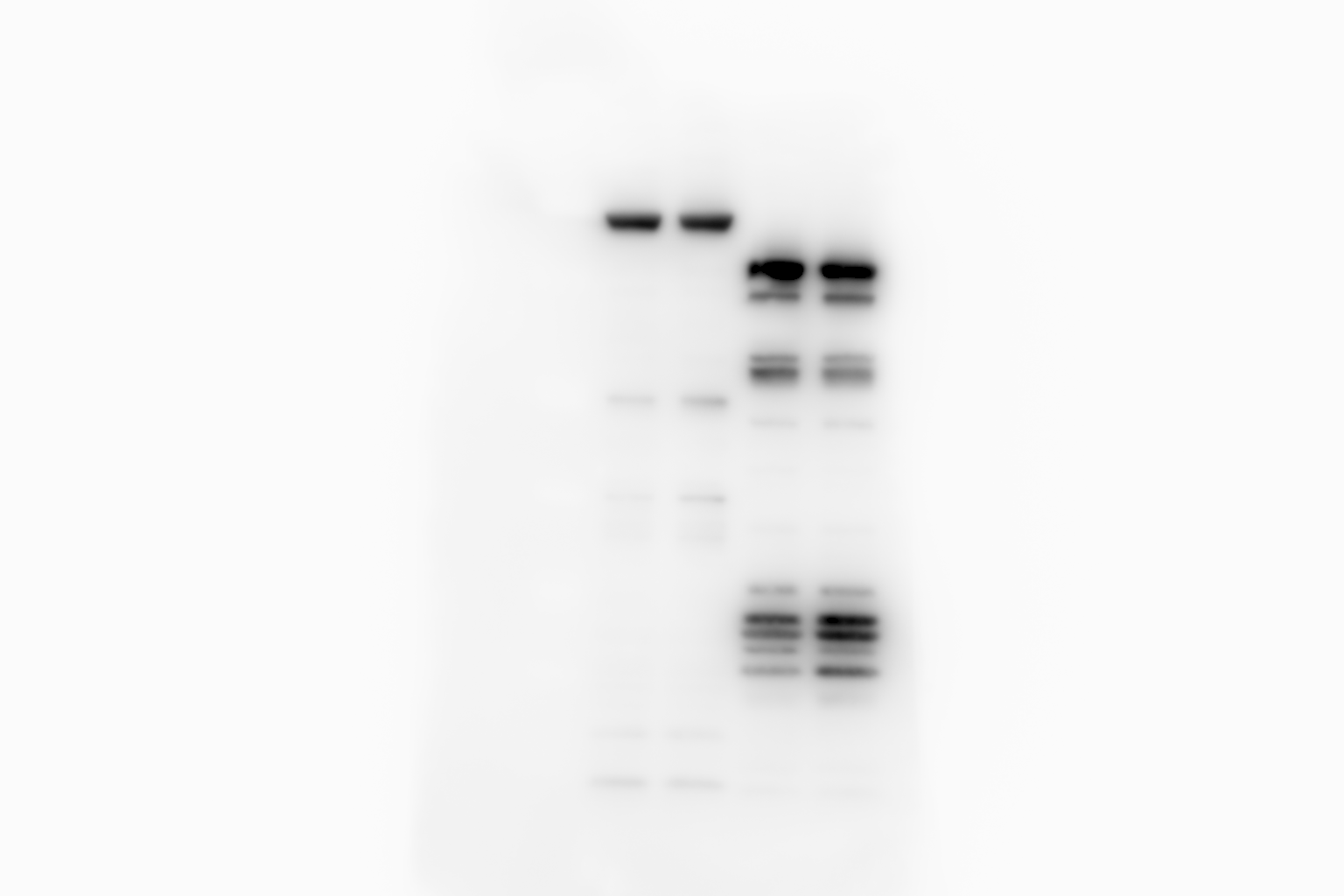

Supplement: Figure 2—source data 1. — Dashed boxes in the PDF indicate the respective areas shown in the figure. [file elife-84877-fig2-data1.zip › Figure2_Source_data_1/Figure2B_left_panel_Flag.tif]

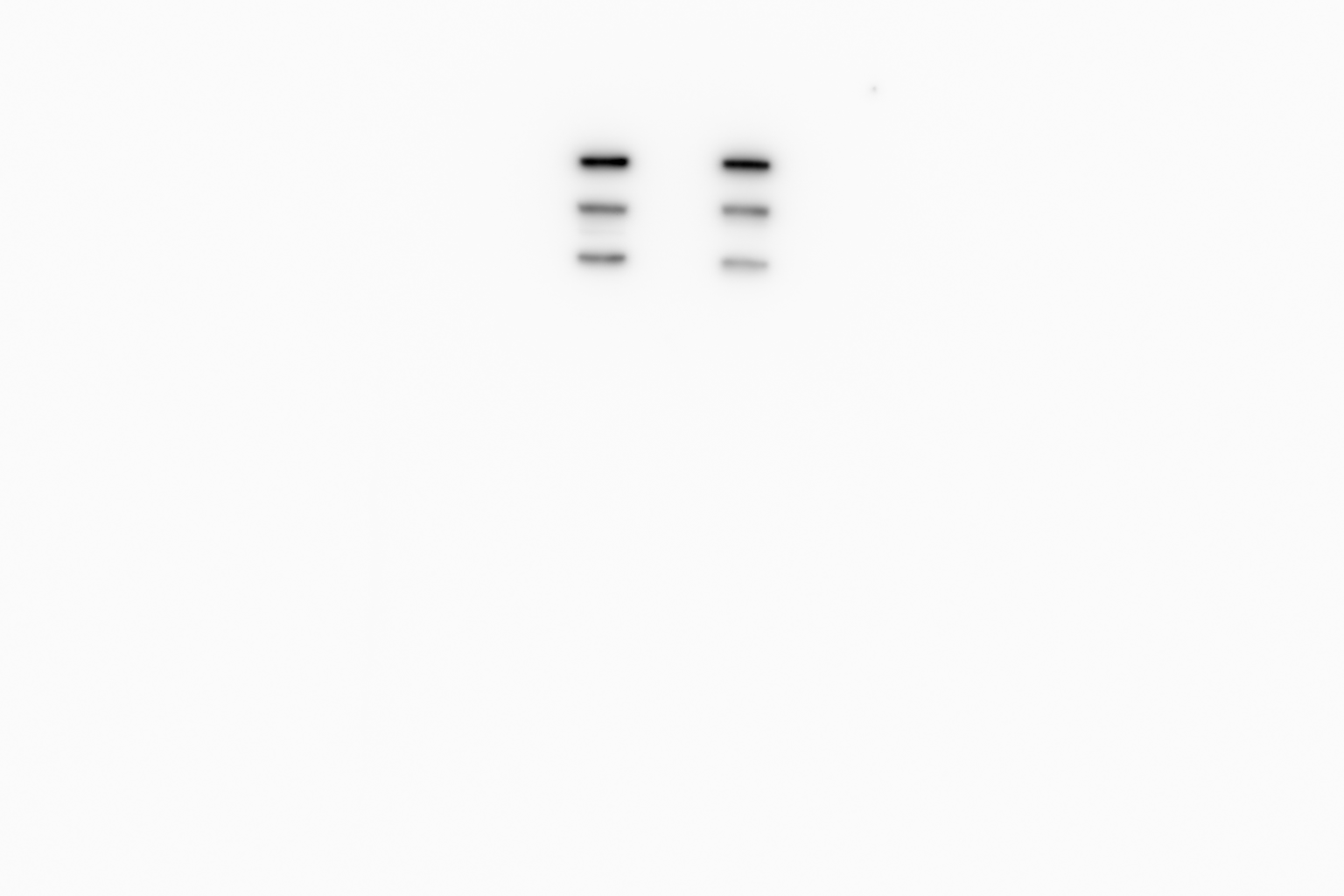

Supplement: Figure 2—source data 1. — Dashed boxes in the PDF indicate the respective areas shown in the figure. [file elife-84877-fig2-data1.zip › Figure2_Source_data_1/Figure2B_left_panel_HA.tif]

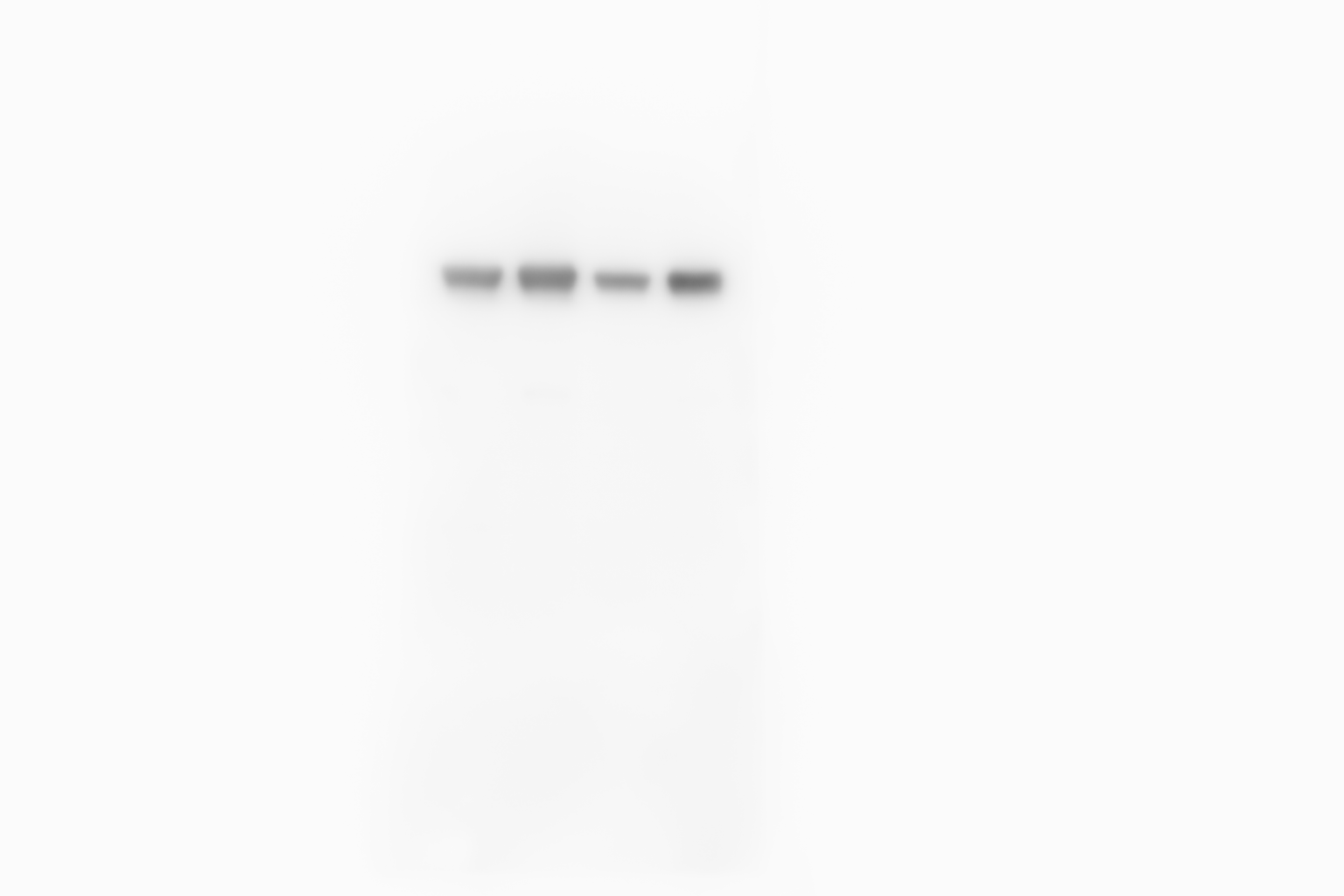

Supplement: Figure 2—source data 1. — Dashed boxes in the PDF indicate the respective areas shown in the figure. [file elife-84877-fig2-data1.zip › Figure2_Source_data_1/Figure2B_left_panel_Has1.tif]

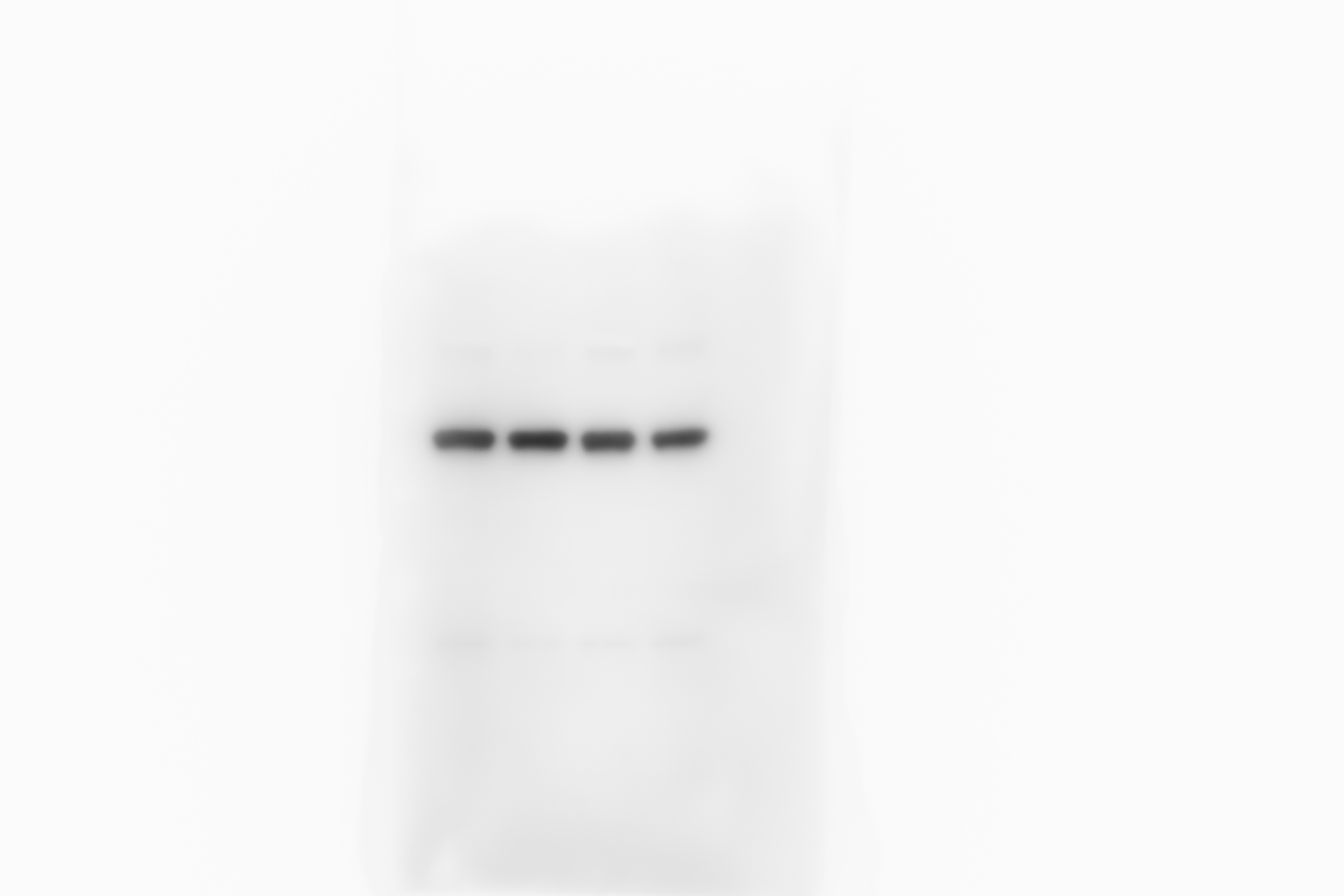

Supplement: Figure 2—source data 1. — Dashed boxes in the PDF indicate the respective areas shown in the figure. [file elife-84877-fig2-data1.zip › Figure2_Source_data_1/Figure2B_left_panel_L3.tif]

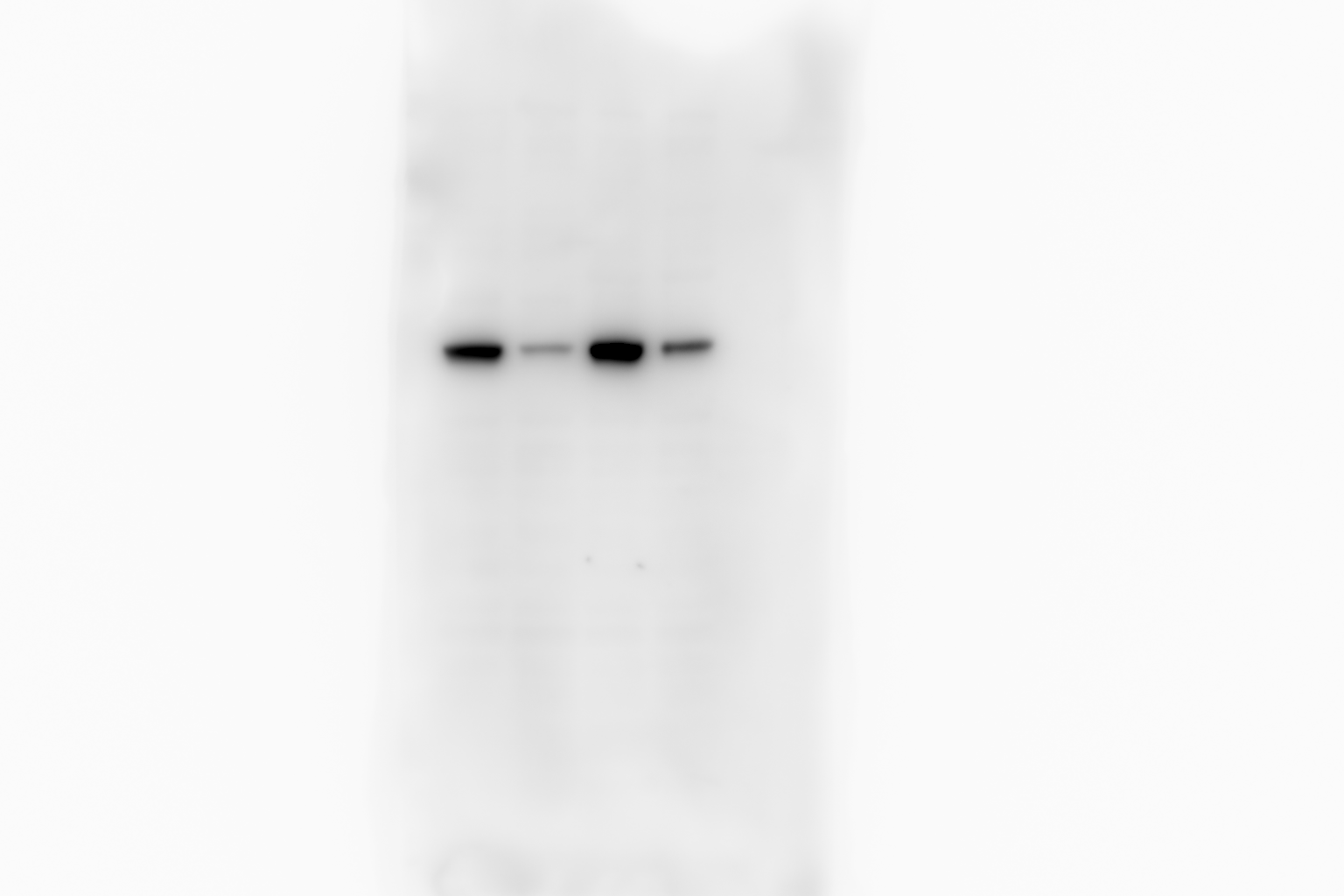

Supplement: Figure 2—source data 1. — Dashed boxes in the PDF indicate the respective areas shown in the figure. [file elife-84877-fig2-data1.zip › Figure2_Source_data_1/Figure2B_left_panel_Nog2.tif]

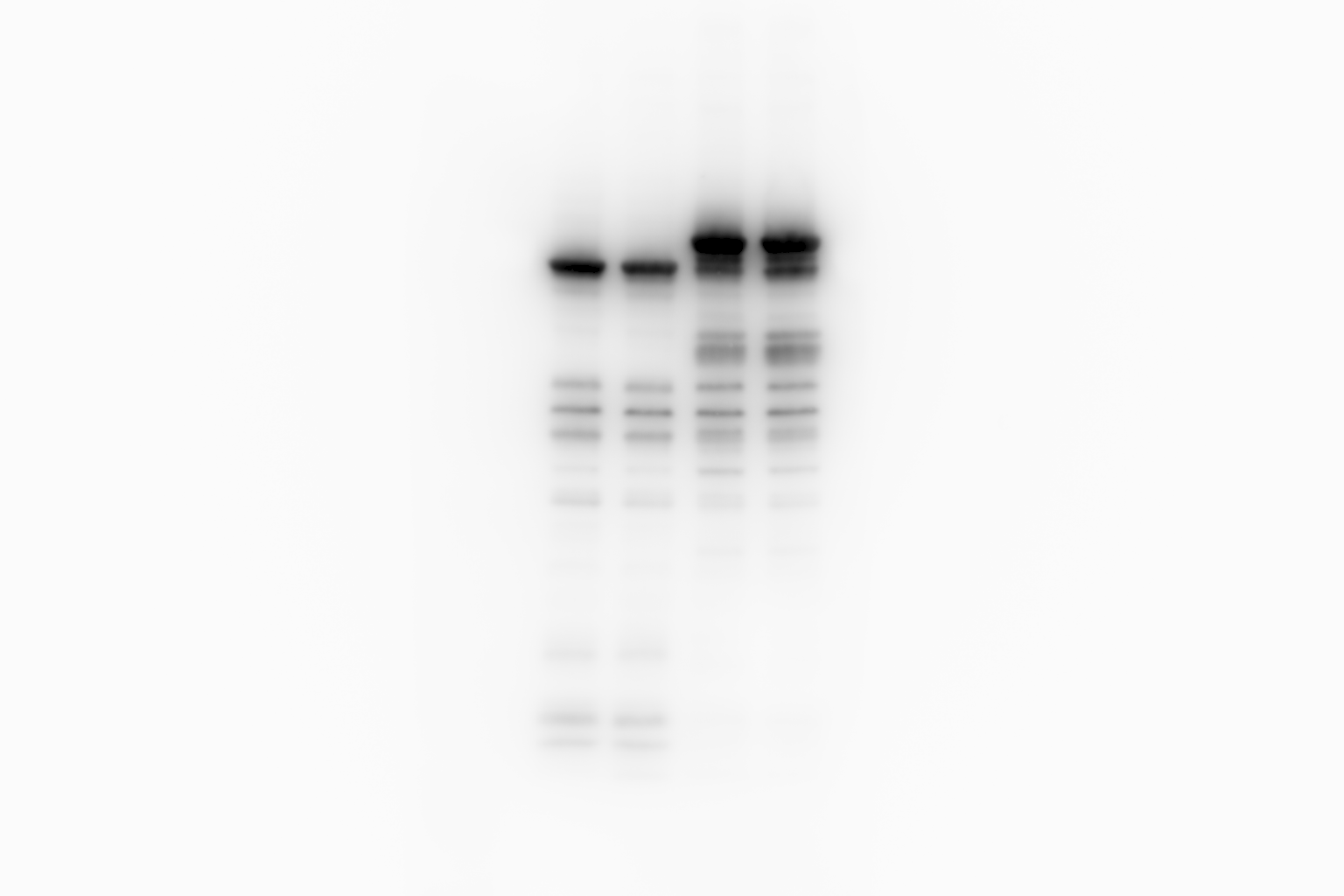

Supplement: Figure 2—source data 1. — Dashed boxes in the PDF indicate the respective areas shown in the figure. [file elife-84877-fig2-data1.zip › Figure2_Source_data_1/Figure2B_left_panel_Nug1.tif]

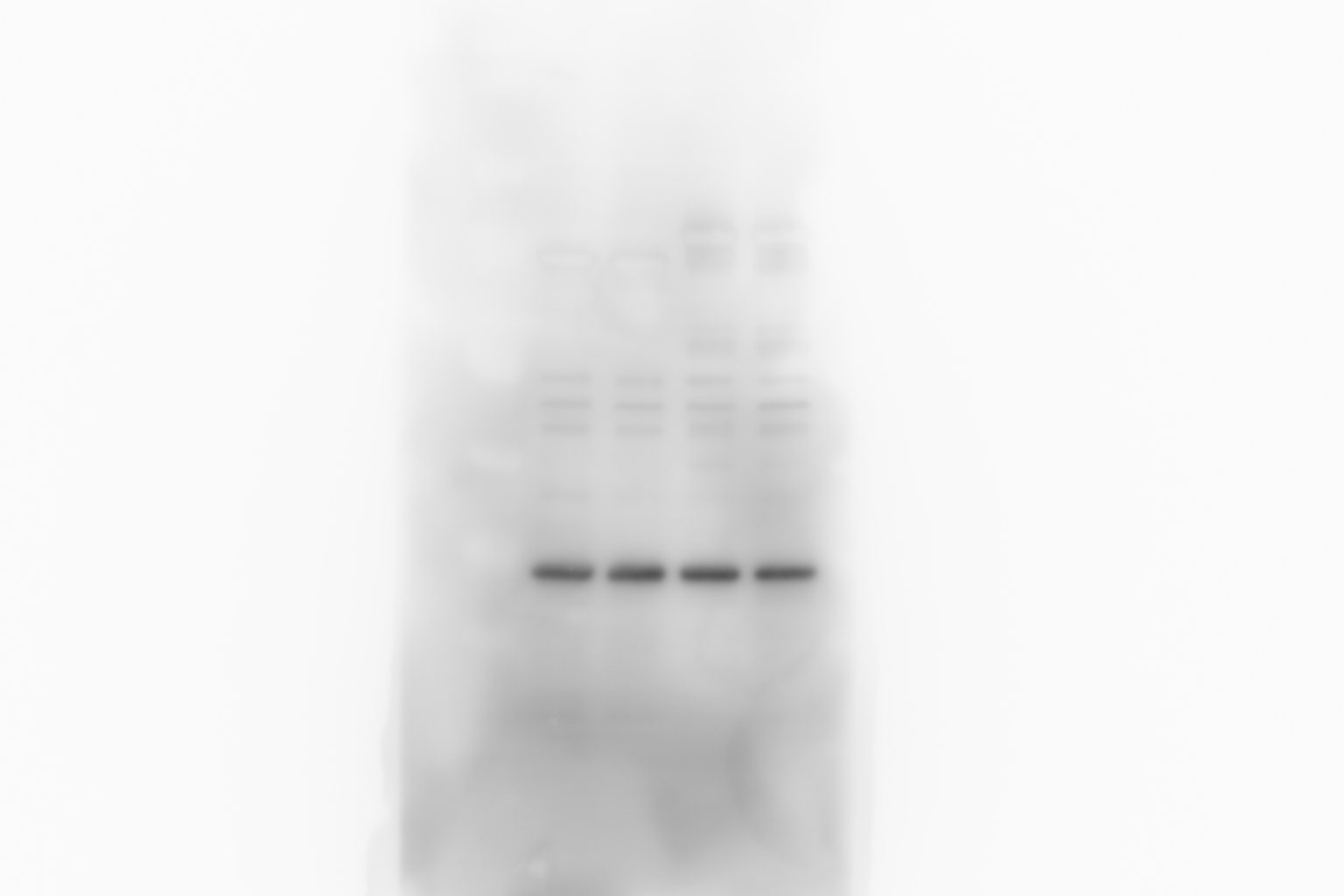

Supplement: Figure 2—source data 1. — Dashed boxes in the PDF indicate the respective areas shown in the figure. [file elife-84877-fig2-data1.zip › Figure2_Source_data_1/Figure2B_left_panel_Rlp24.tif]

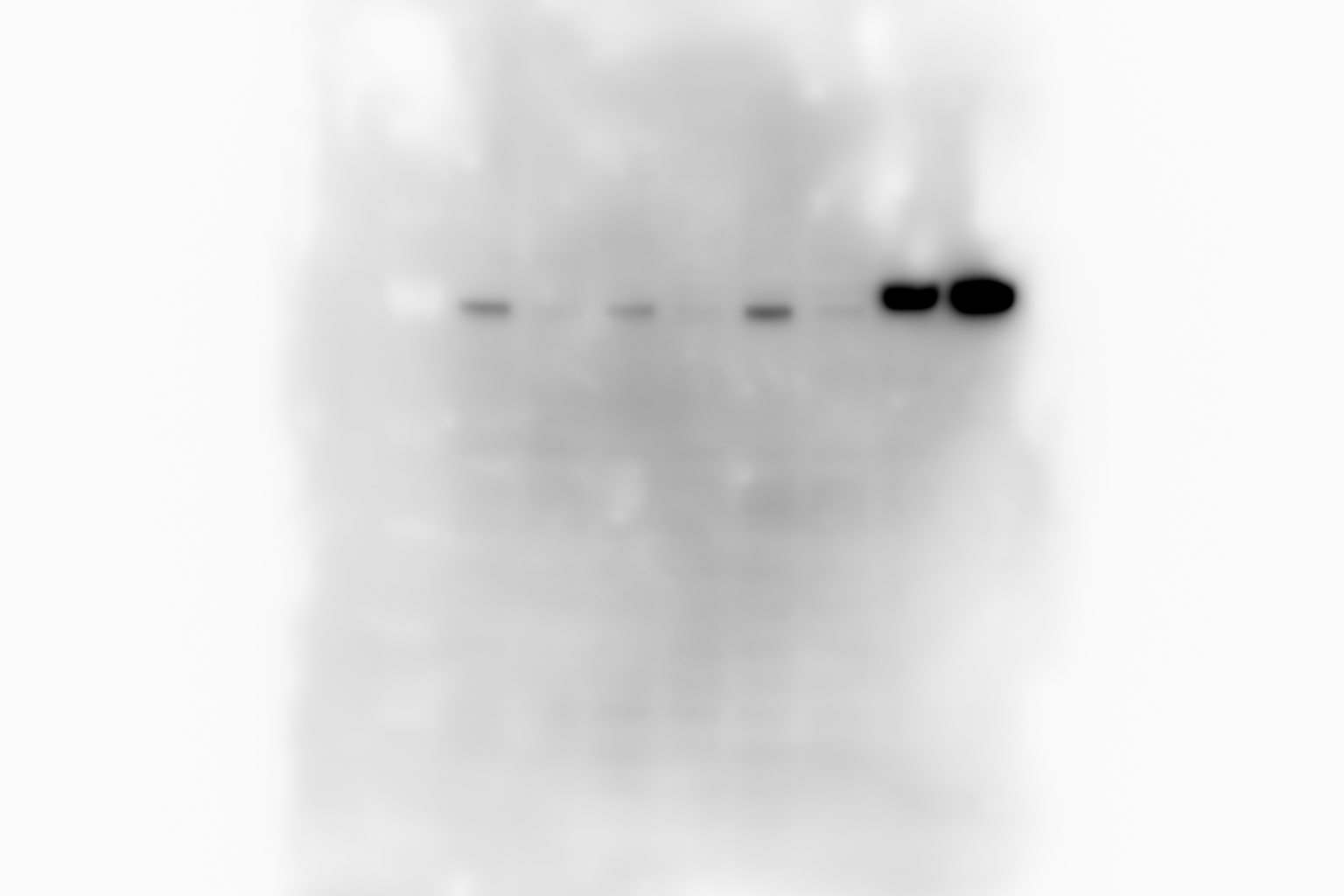

Supplement: Figure 2—source data 1. — Dashed boxes in the PDF indicate the respective areas shown in the figure. [file elife-84877-fig2-data1.zip › Figure2_Source_data_1/Figure2B_right_panel_Arx1.tif]

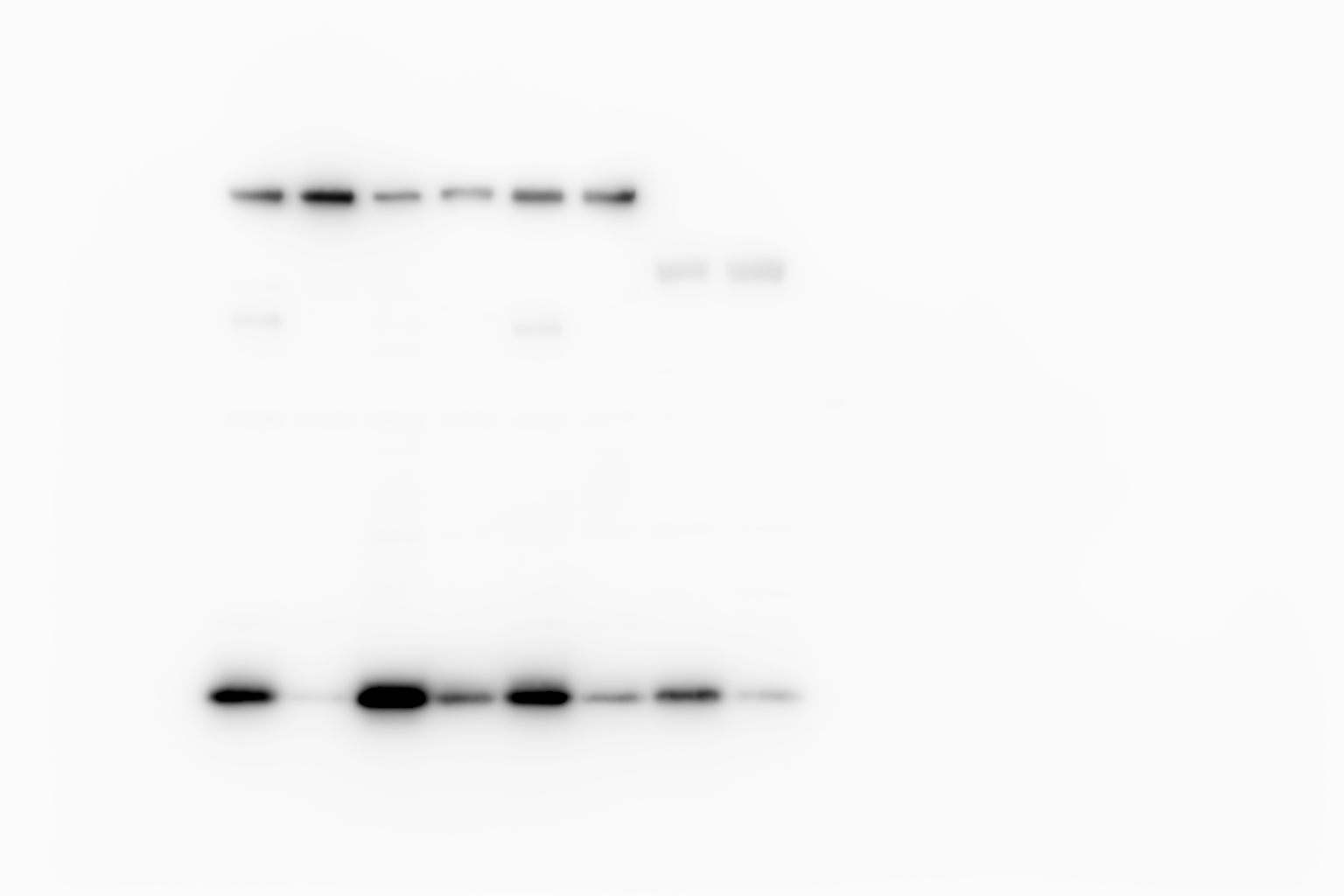

Supplement: Figure 2—source data 1. — Dashed boxes in the PDF indicate the respective areas shown in the figure. [file elife-84877-fig2-data1.zip › Figure2_Source_data_1/Figure2B_right_panel_Bud20.tif]

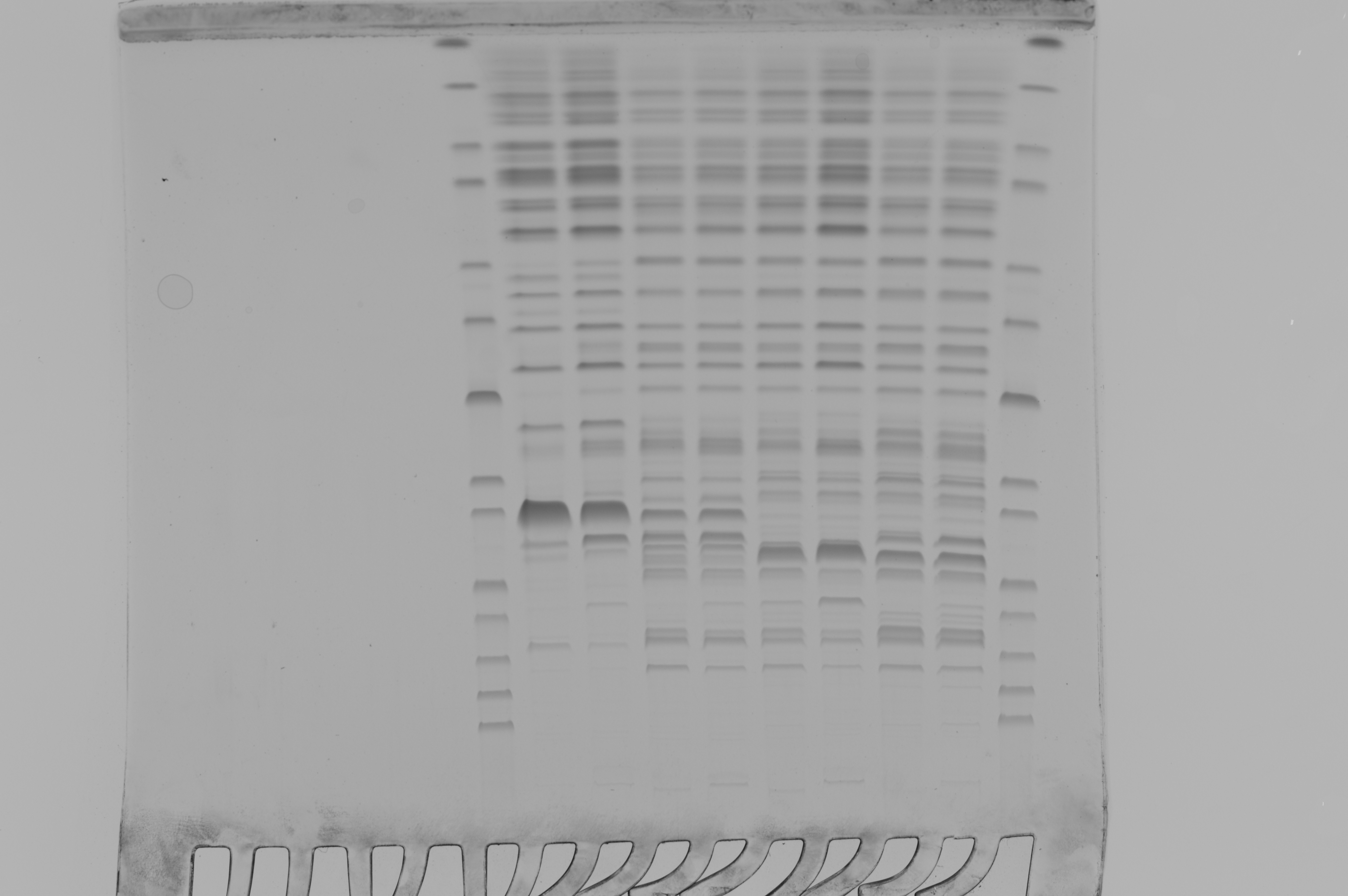

Supplement: Figure 2—source data 1. — Dashed boxes in the PDF indicate the respective areas shown in the figure. [file elife-84877-fig2-data1.zip › Figure2_Source_data_1/Figure2B_right_panel_Coomassie.JPG]

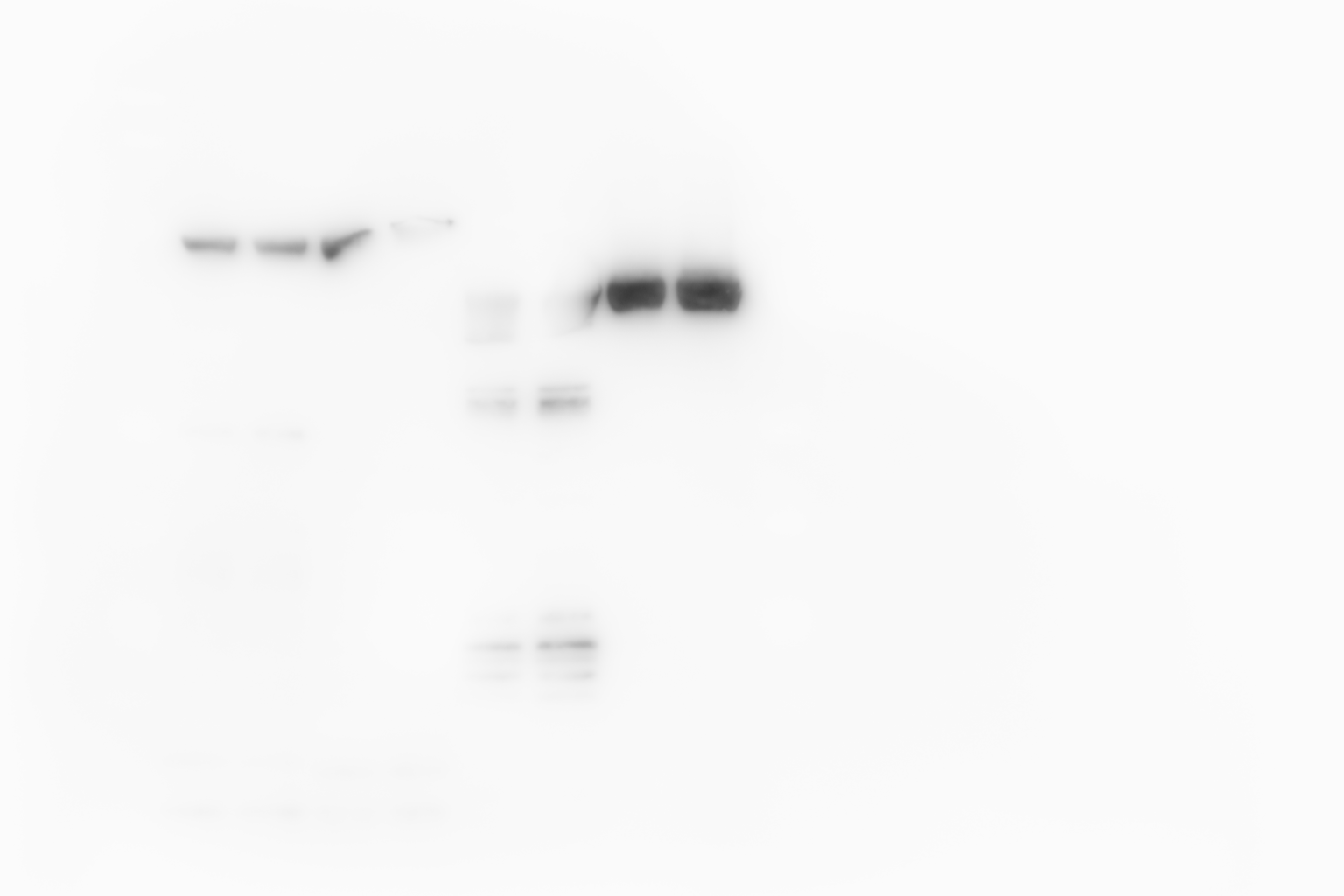

Supplement: Figure 2—source data 1. — Dashed boxes in the PDF indicate the respective areas shown in the figure. [file elife-84877-fig2-data1.zip › Figure2_Source_data_1/Figure2B_right_panel_Flag.tif]

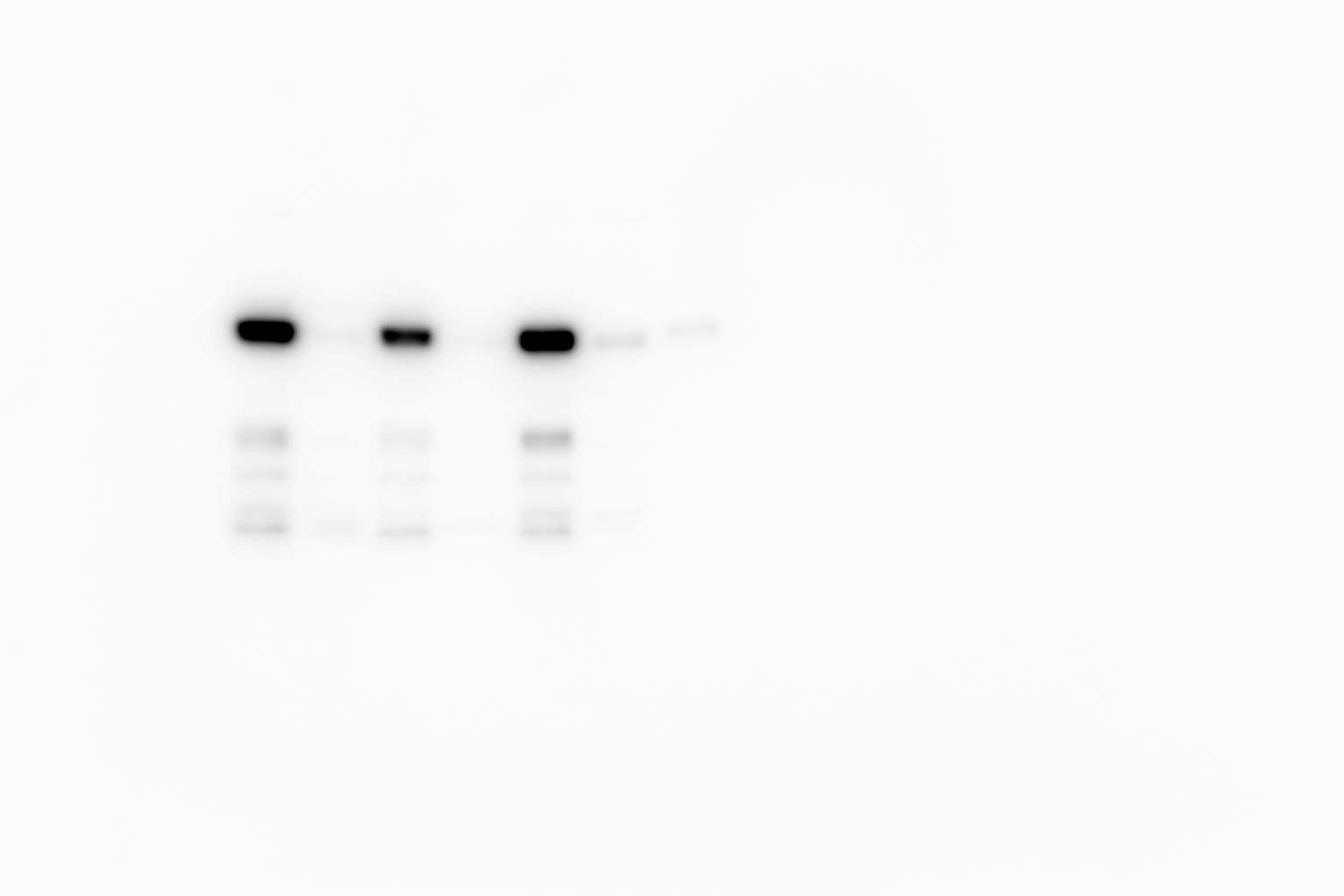

Supplement: Figure 2—source data 1. — Dashed boxes in the PDF indicate the respective areas shown in the figure. [file elife-84877-fig2-data1.zip › Figure2_Source_data_1/Figure2B_right_panel_HA.tif]

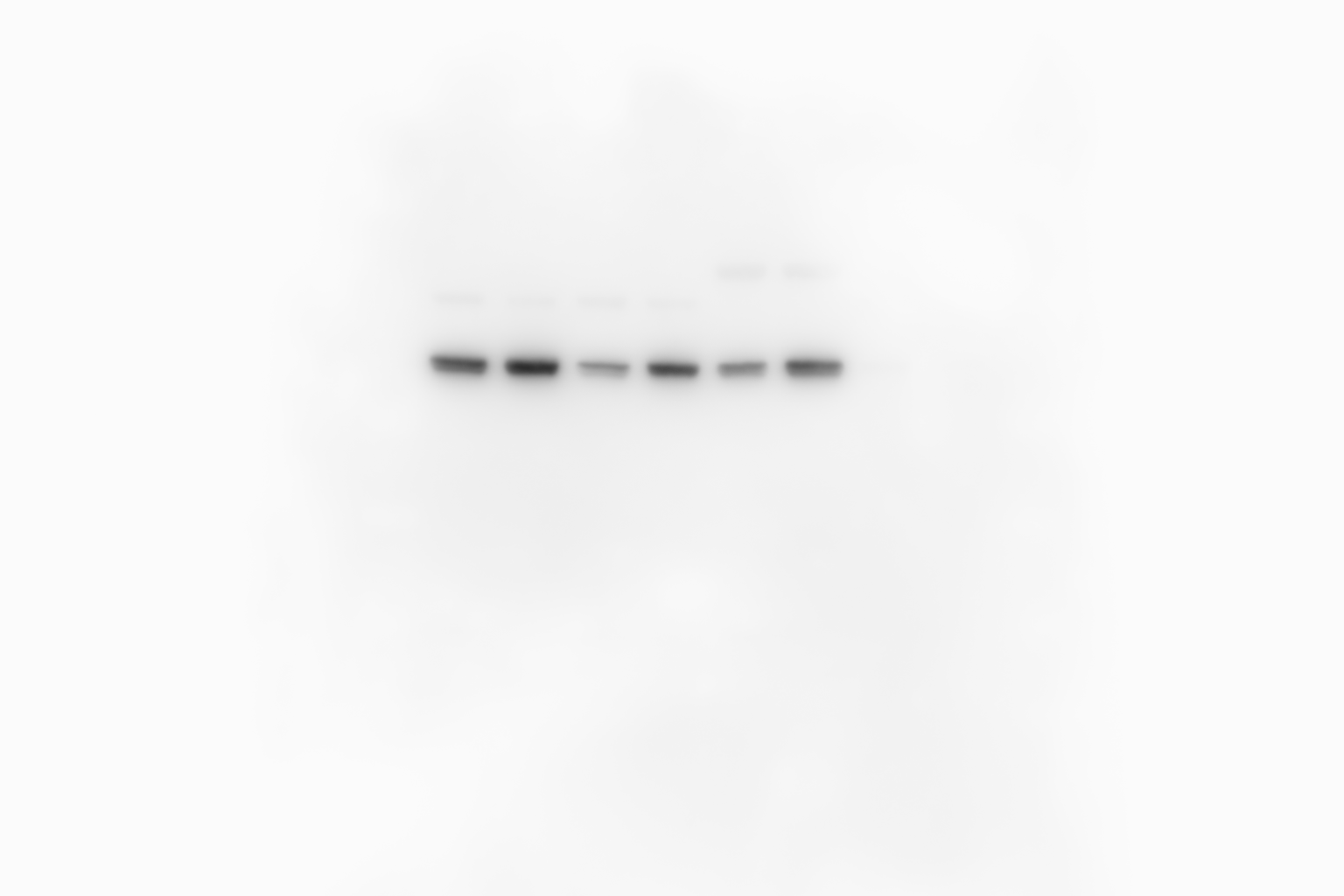

Supplement: Figure 2—source data 1. — Dashed boxes in the PDF indicate the respective areas shown in the figure. [file elife-84877-fig2-data1.zip › Figure2_Source_data_1/Figure2B_right_panel_Has1.tif]

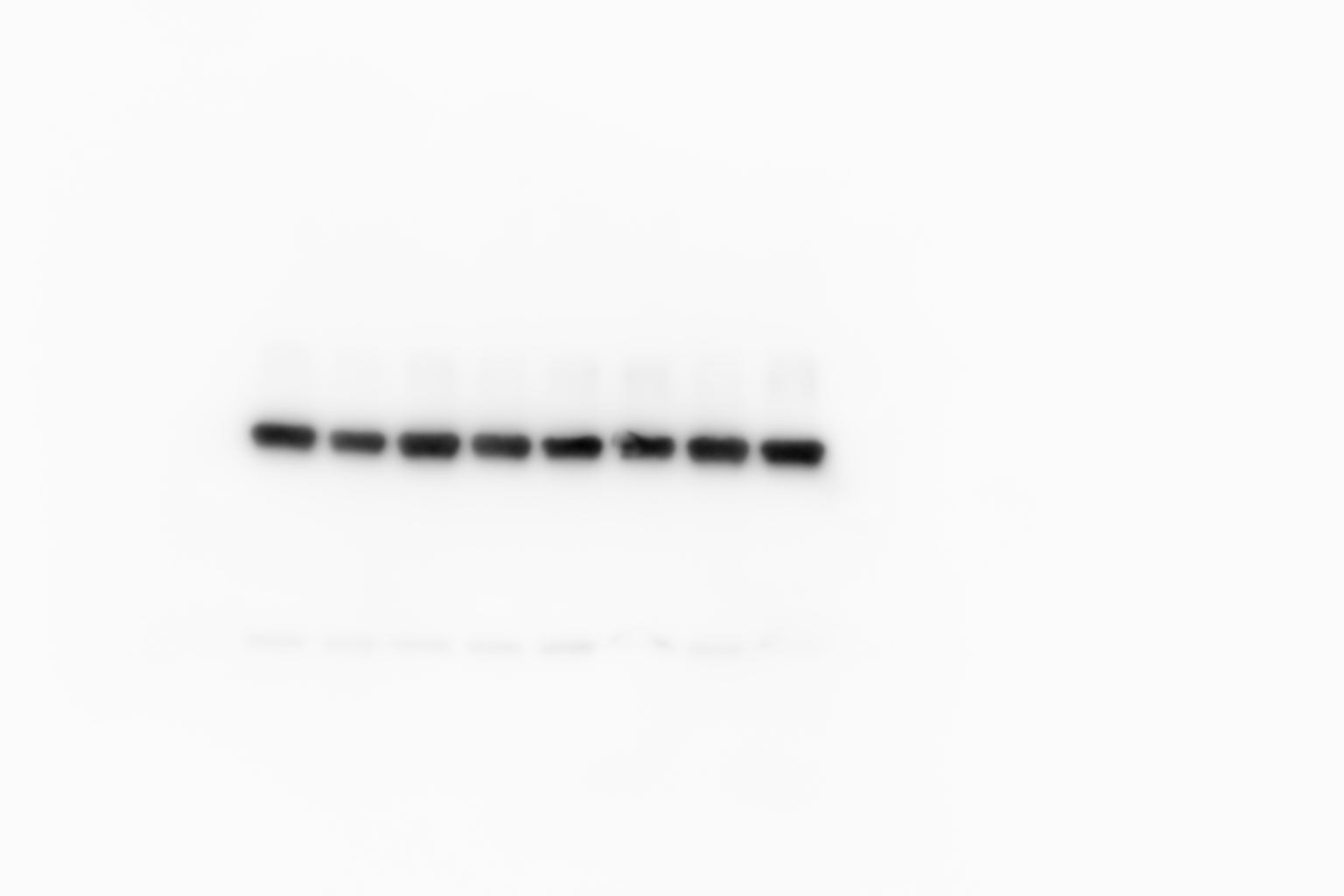

Supplement: Figure 2—source data 1. — Dashed boxes in the PDF indicate the respective areas shown in the figure. [file elife-84877-fig2-data1.zip › Figure2_Source_data_1/Figure2B_right_panel_L3.tif]

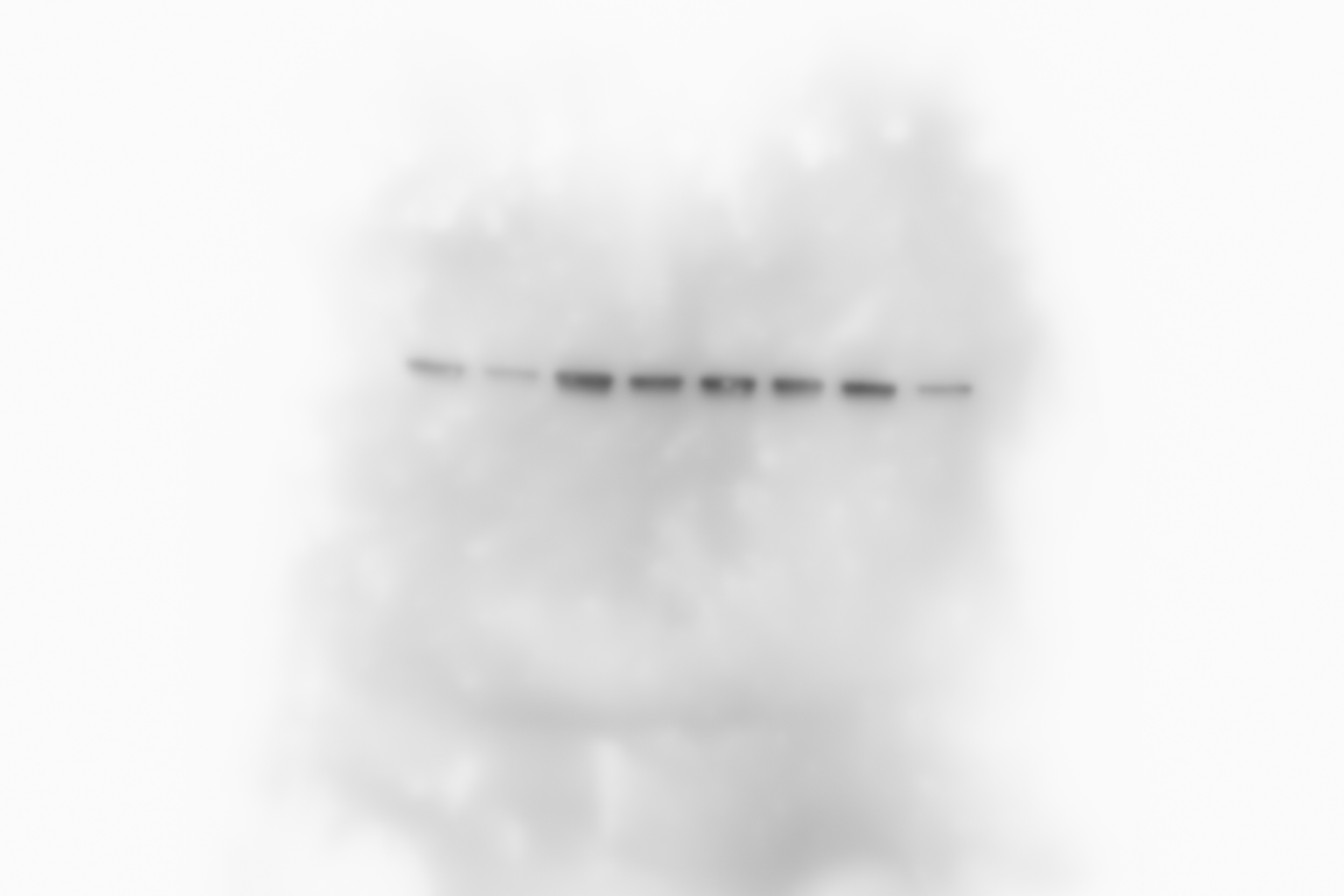

Supplement: Figure 2—source data 1. — Dashed boxes in the PDF indicate the respective areas shown in the figure. [file elife-84877-fig2-data1.zip › Figure2_Source_data_1/Figure2B_right_panel_Nog2.tif]

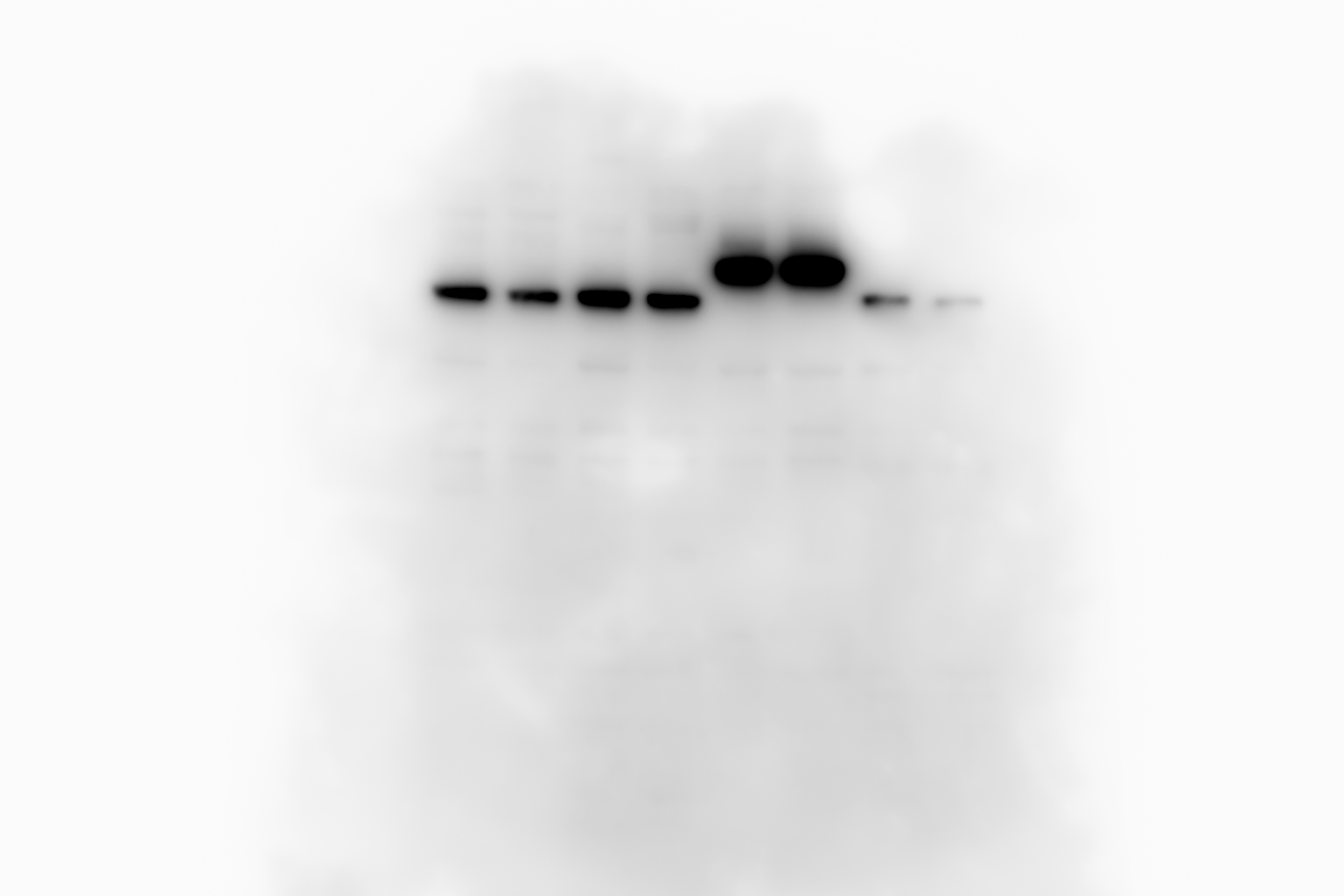

Supplement: Figure 2—source data 1. — Dashed boxes in the PDF indicate the respective areas shown in the figure. [file elife-84877-fig2-data1.zip › Figure2_Source_data_1/Figure2B_right_panel_Nug1.tif]

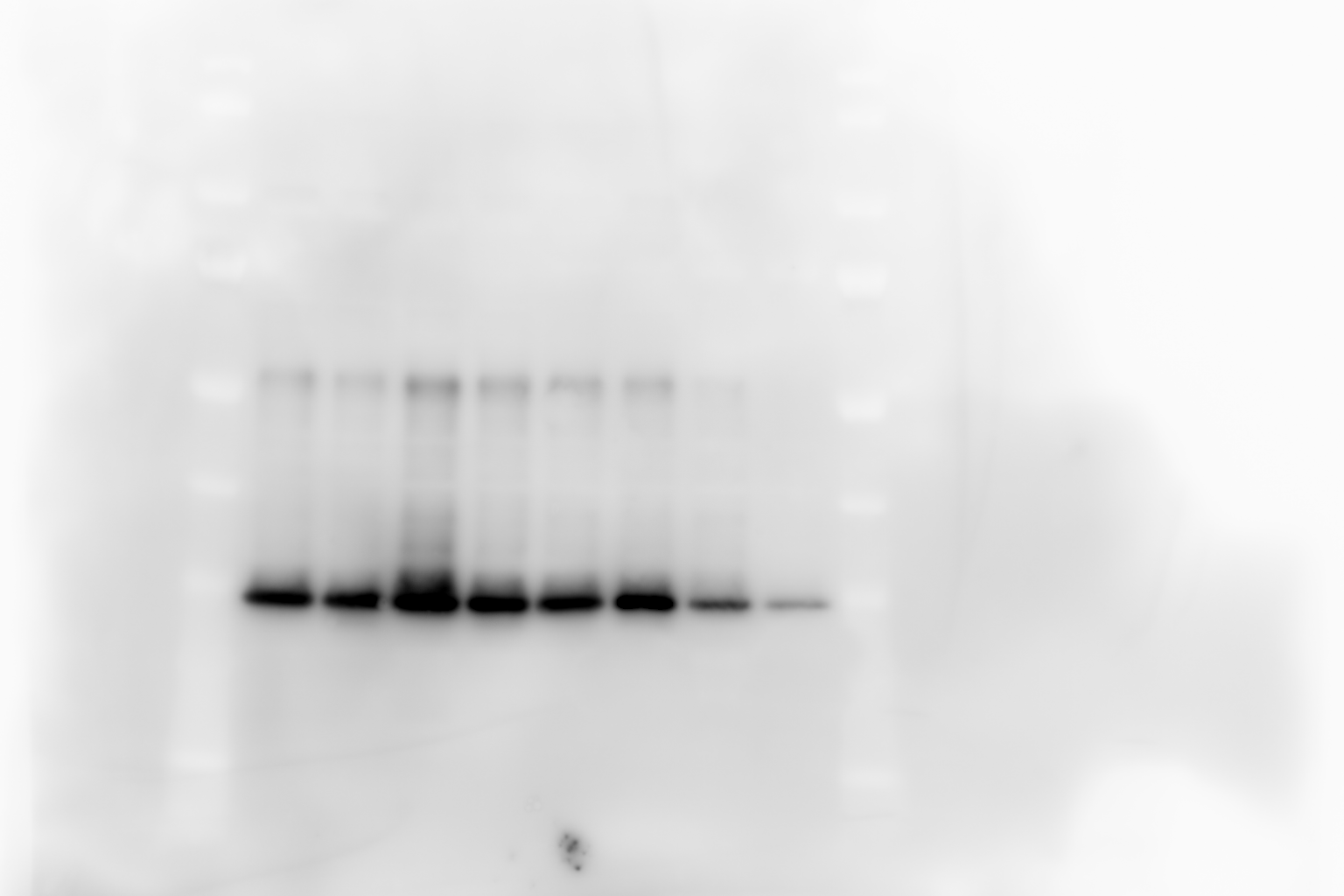

Supplement: Figure 2—source data 1. — Dashed boxes in the PDF indicate the respective areas shown in the figure. [file elife-84877-fig2-data1.zip › Figure2_Source_data_1/Figure2B_right_panel_Rlp24.tif]

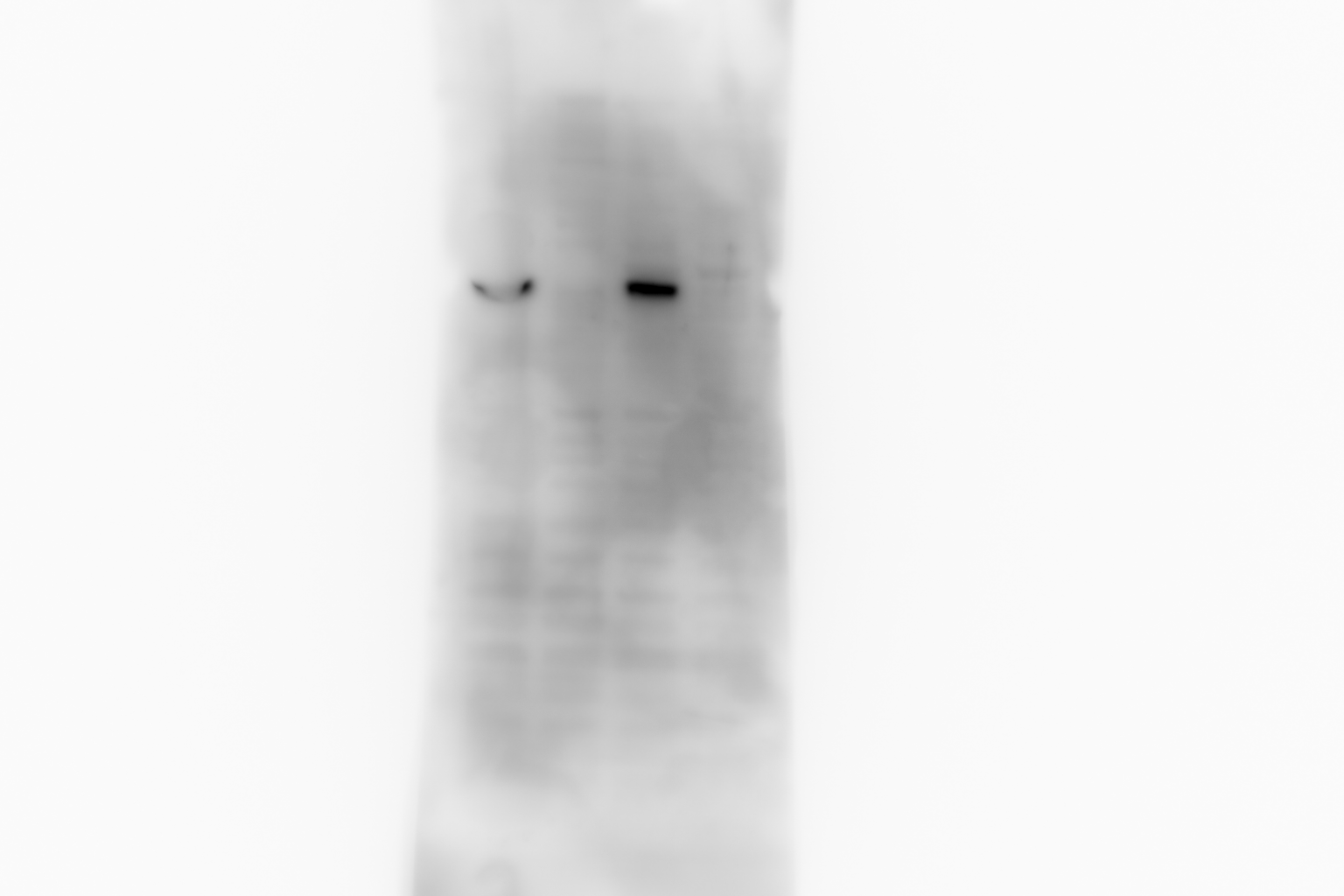

Supplement: Figure 2—source data 2. — Dashed boxes in the PDF indicate the respective areas shown in the figure. [file elife-84877-fig2-data2.zip › Figure2_Source_data_2/Figure2C_left_panel_Arx1.tif]

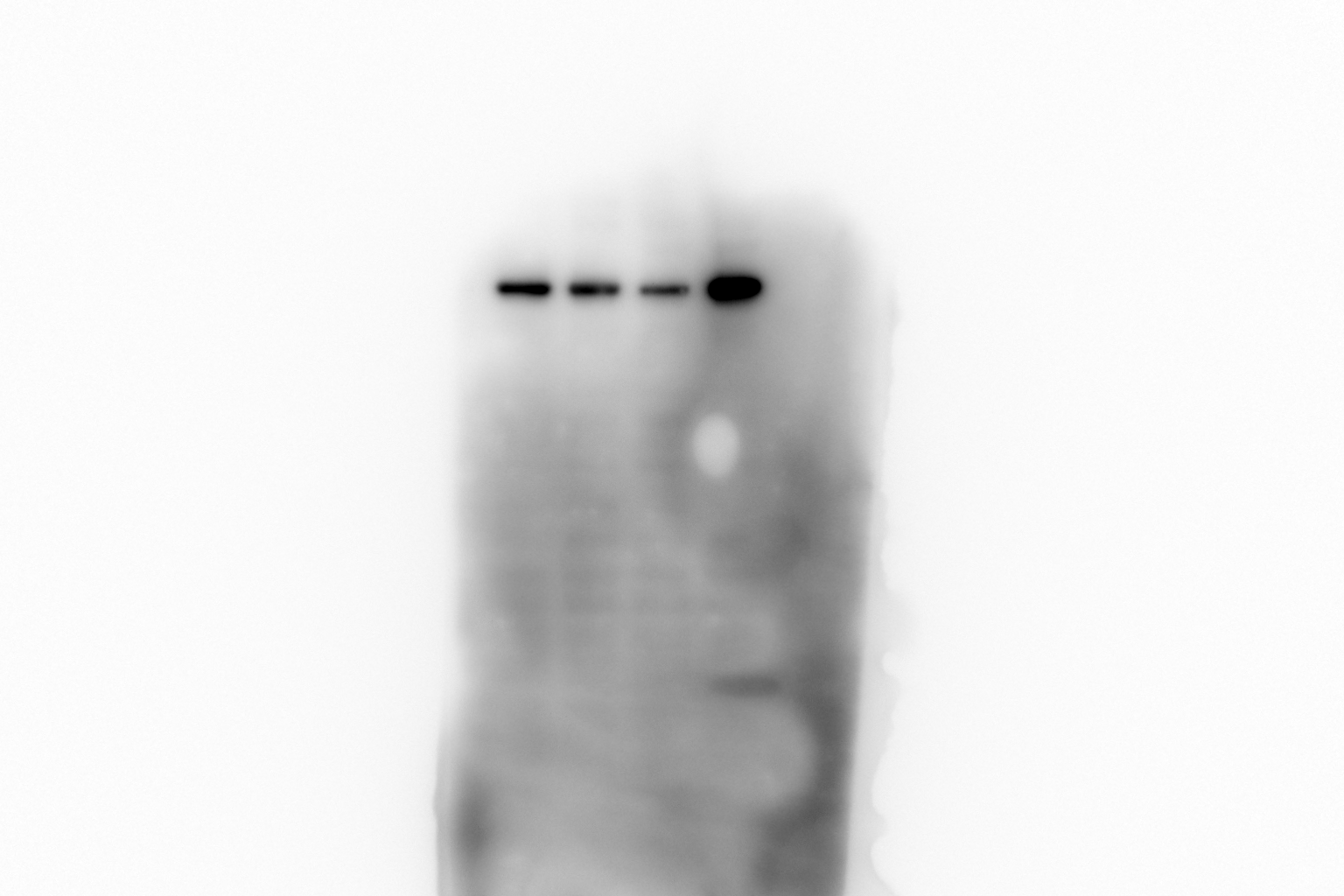

Supplement: Figure 2—source data 2. — Dashed boxes in the PDF indicate the respective areas shown in the figure. [file elife-84877-fig2-data2.zip › Figure2_Source_data_2/Figure2C_left_panel_Ebp2.tif]

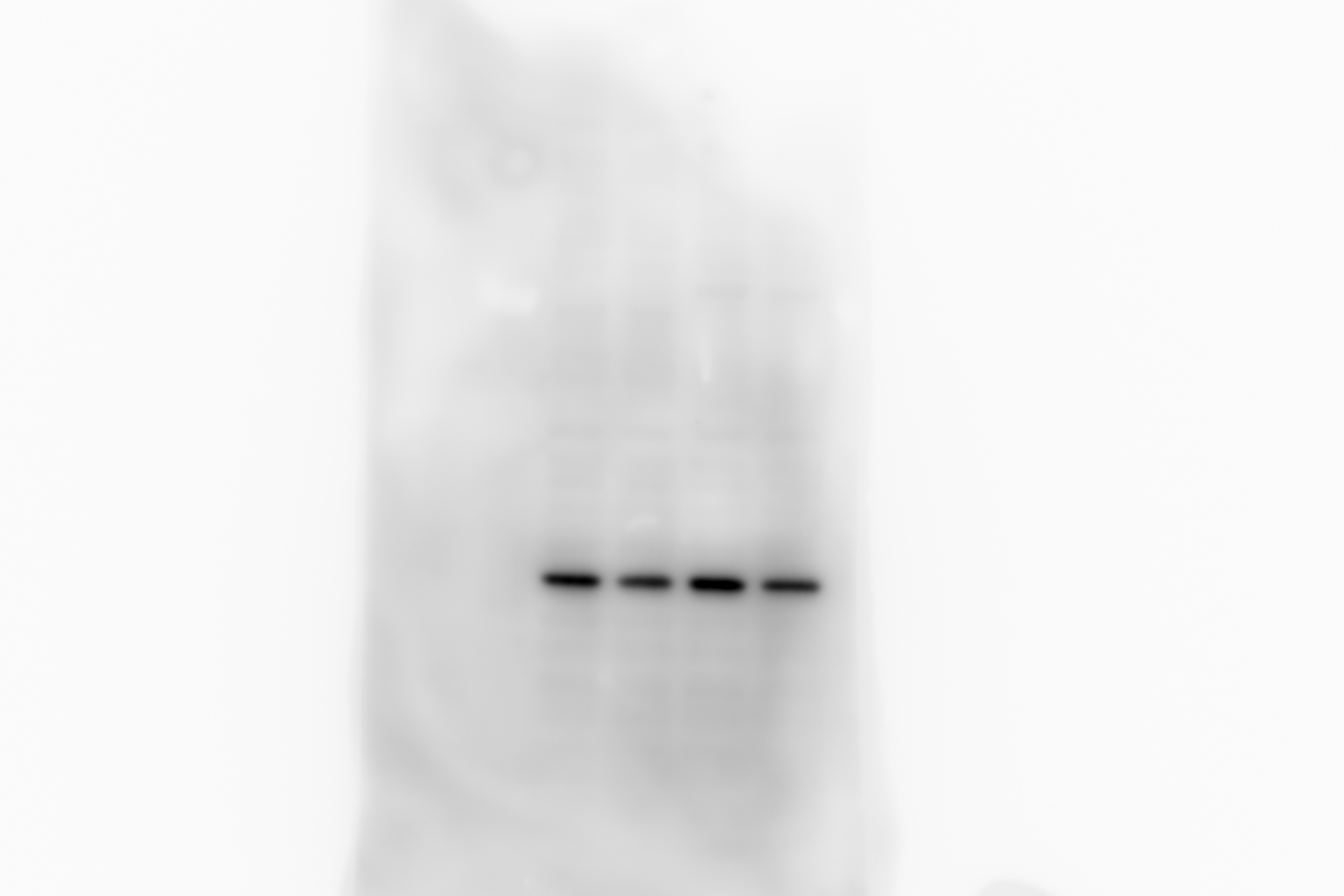

Supplement: Figure 2—source data 2. — Dashed boxes in the PDF indicate the respective areas shown in the figure. [file elife-84877-fig2-data2.zip › Figure2_Source_data_2/Figure2C_left_panel_Nsa2.tif]

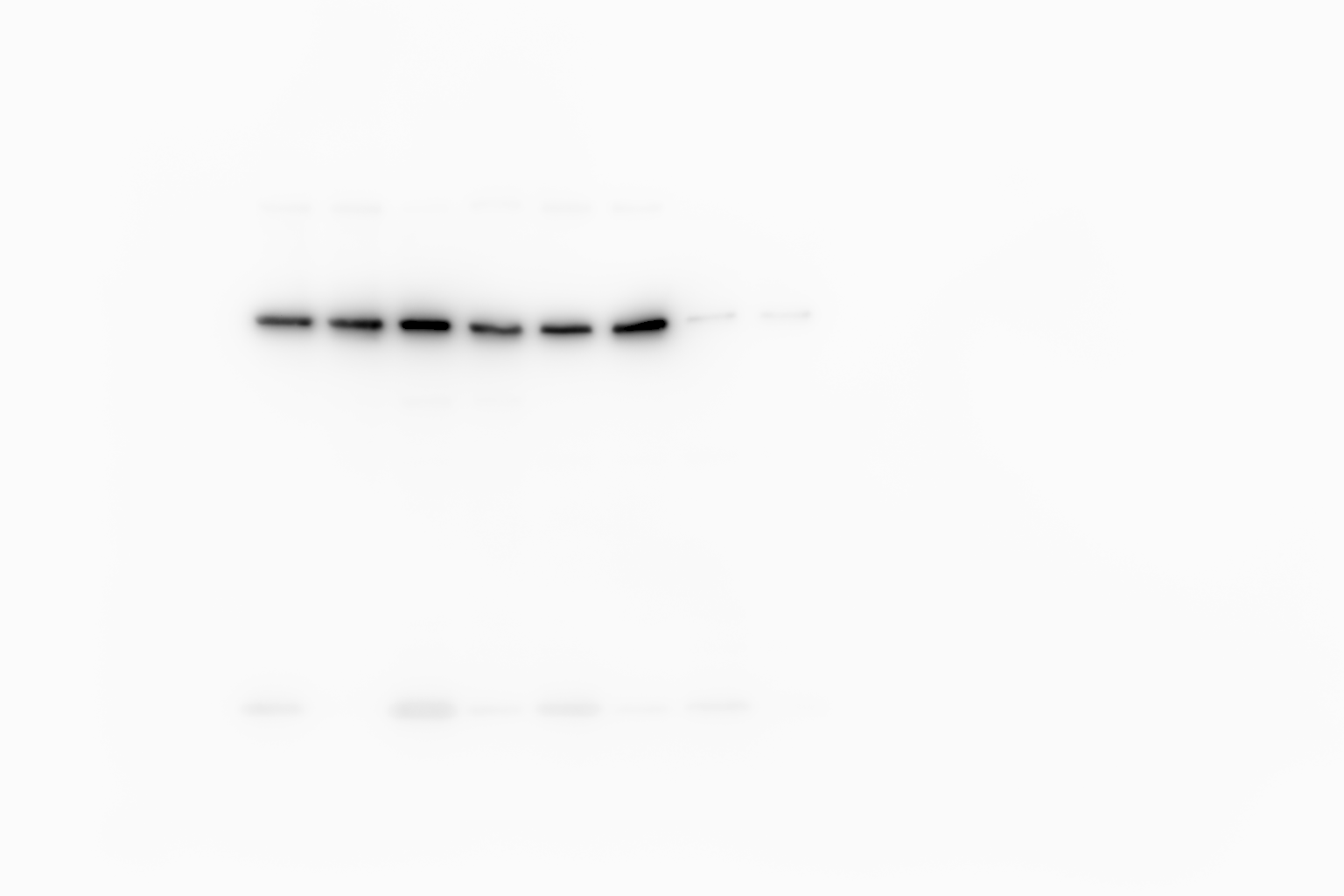

Supplement: Figure 2—source data 2. — Dashed boxes in the PDF indicate the respective areas shown in the figure. [file elife-84877-fig2-data2.zip › Figure2_Source_data_2/Figure2C_right_panel_Ebp2.tif]

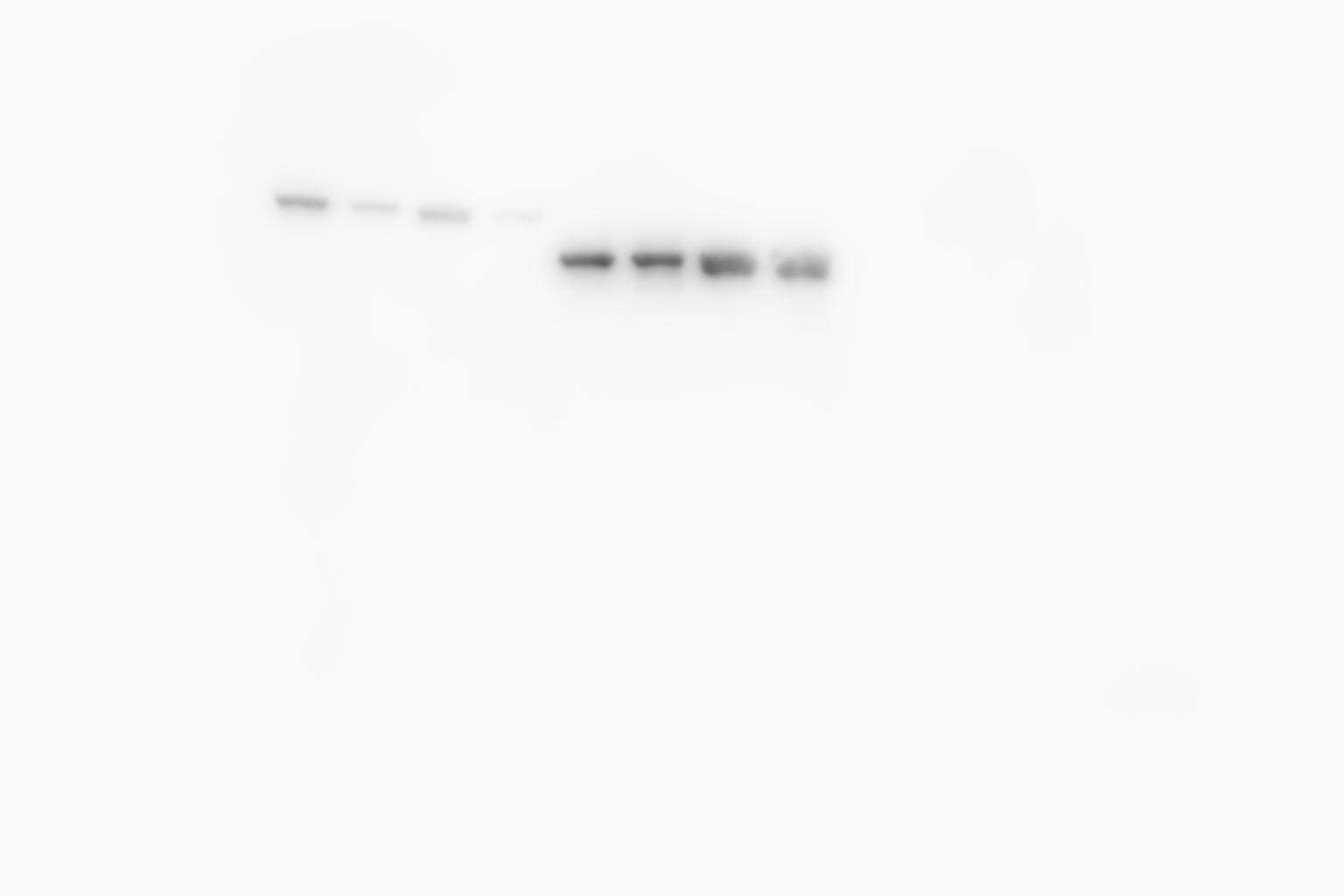

Supplement: Figure 2—source data 2. — Dashed boxes in the PDF indicate the respective areas shown in the figure. [file elife-84877-fig2-data2.zip › Figure2_Source_data_2/Figure2C_right_panel_Flag.tif]

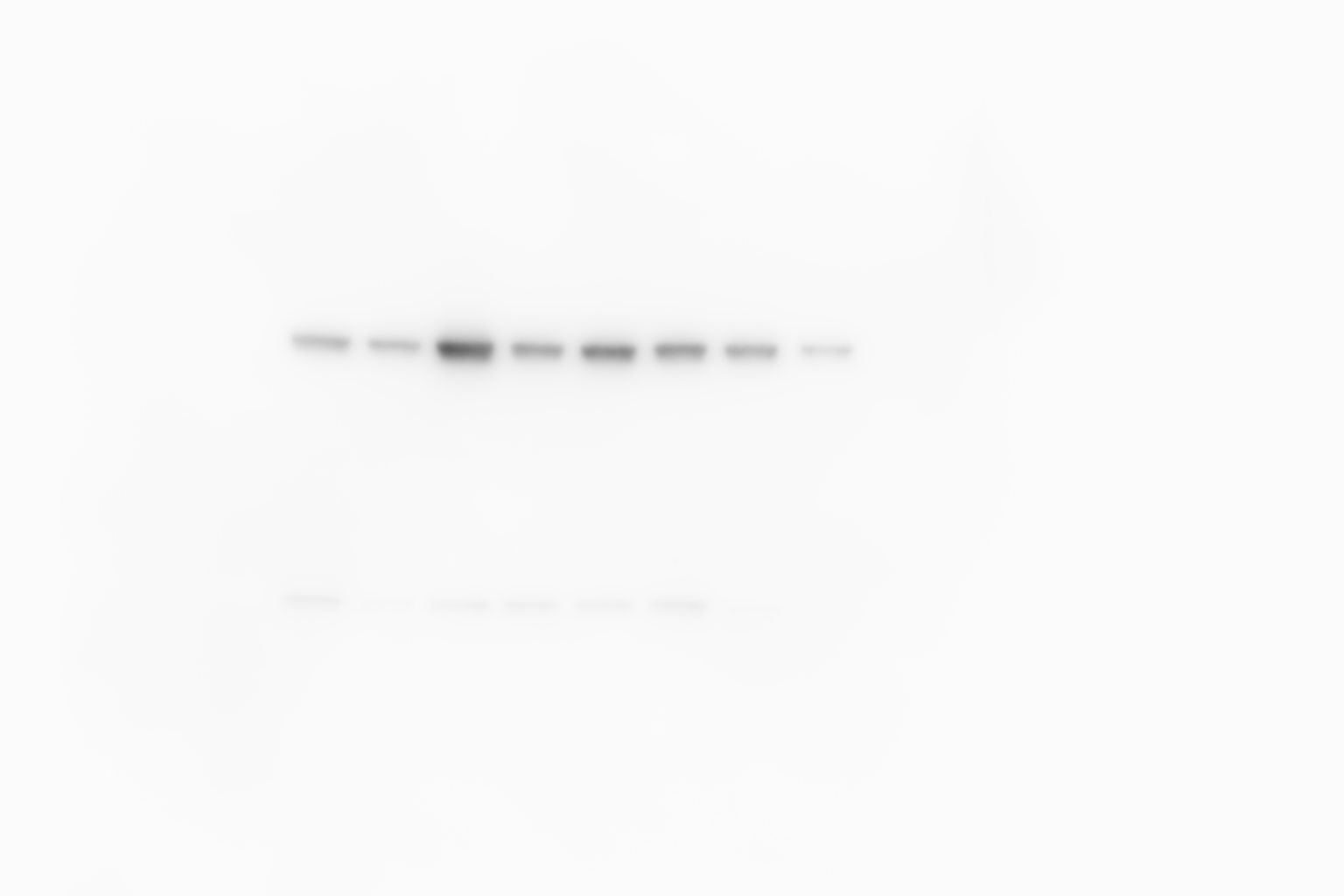

Supplement: Figure 2—source data 2. — Dashed boxes in the PDF indicate the respective areas shown in the figure. [file elife-84877-fig2-data2.zip › Figure2_Source_data_2/Figure2C_right_panel_Nog2.tif]

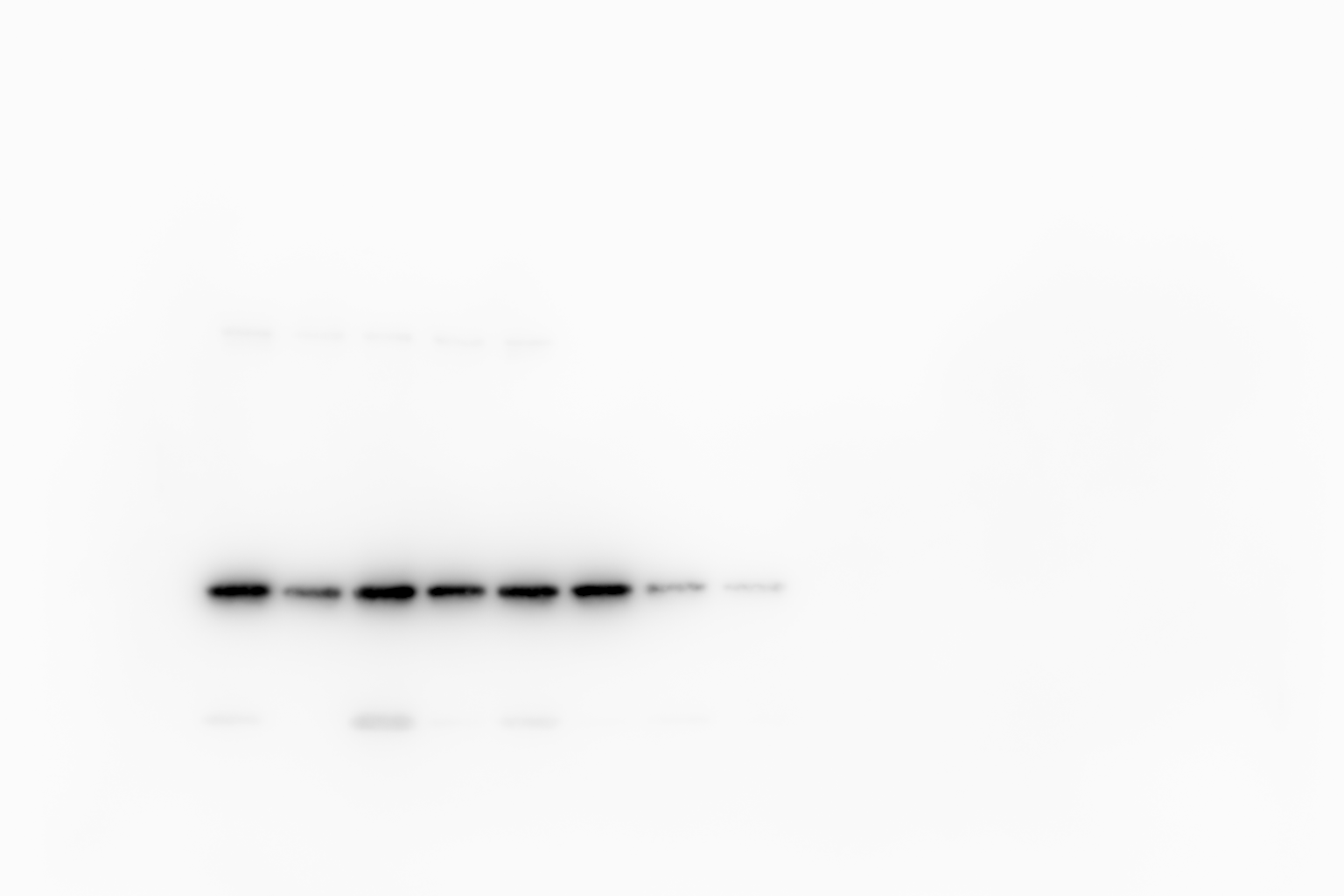

Supplement: Figure 2—source data 2. — Dashed boxes in the PDF indicate the respective areas shown in the figure. [file elife-84877-fig2-data2.zip › Figure2_Source_data_2/Figure2C_right_panel_Nsa2.tif]

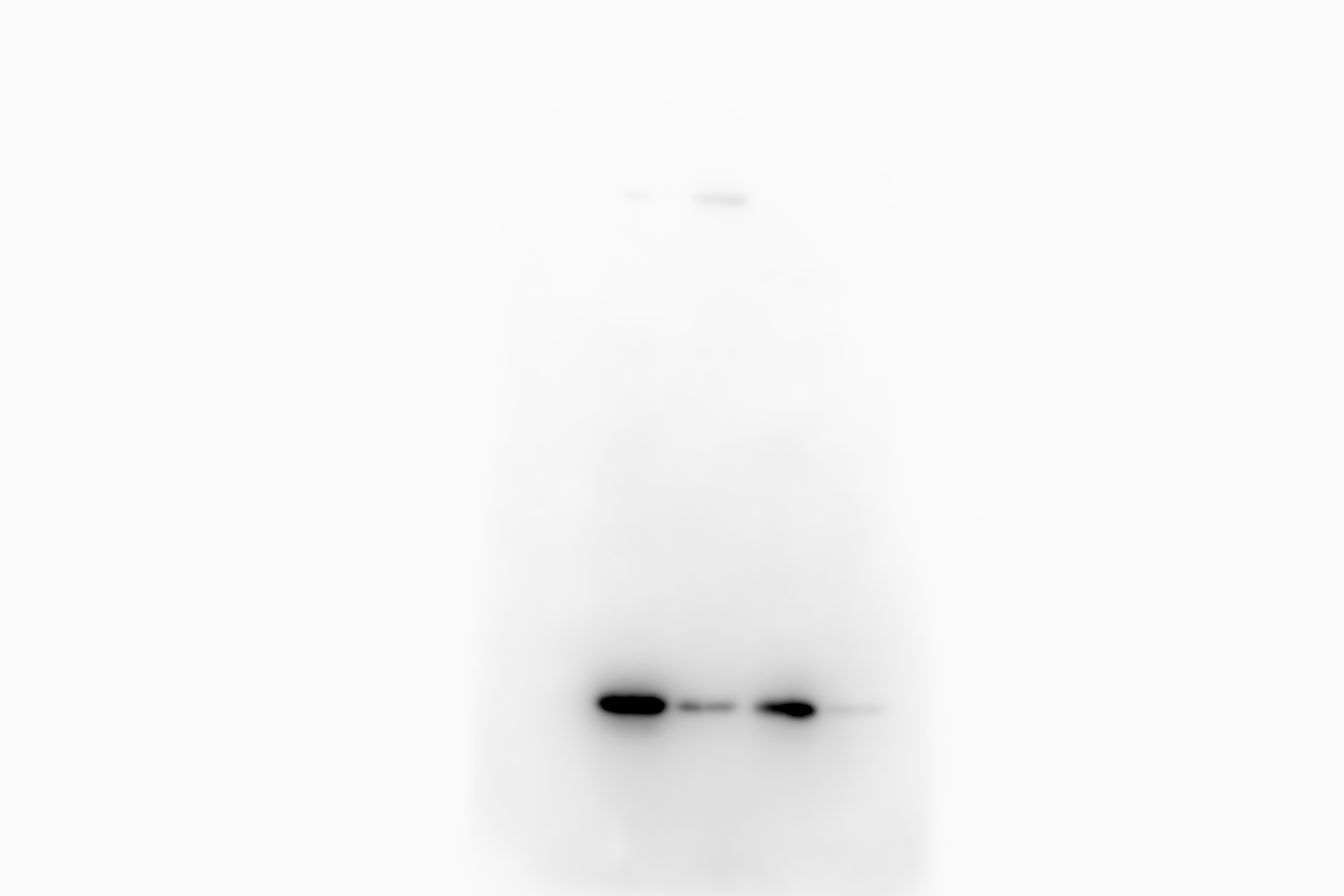

Supplement: Figure 2—source data 3. — Dashed boxes in the PDF indicate the respective areas shown in the figure. [file elife-84877-fig2-data3.zip › Figure2_Source_data_3/Figure2D_left_panel_Bud20.tif]

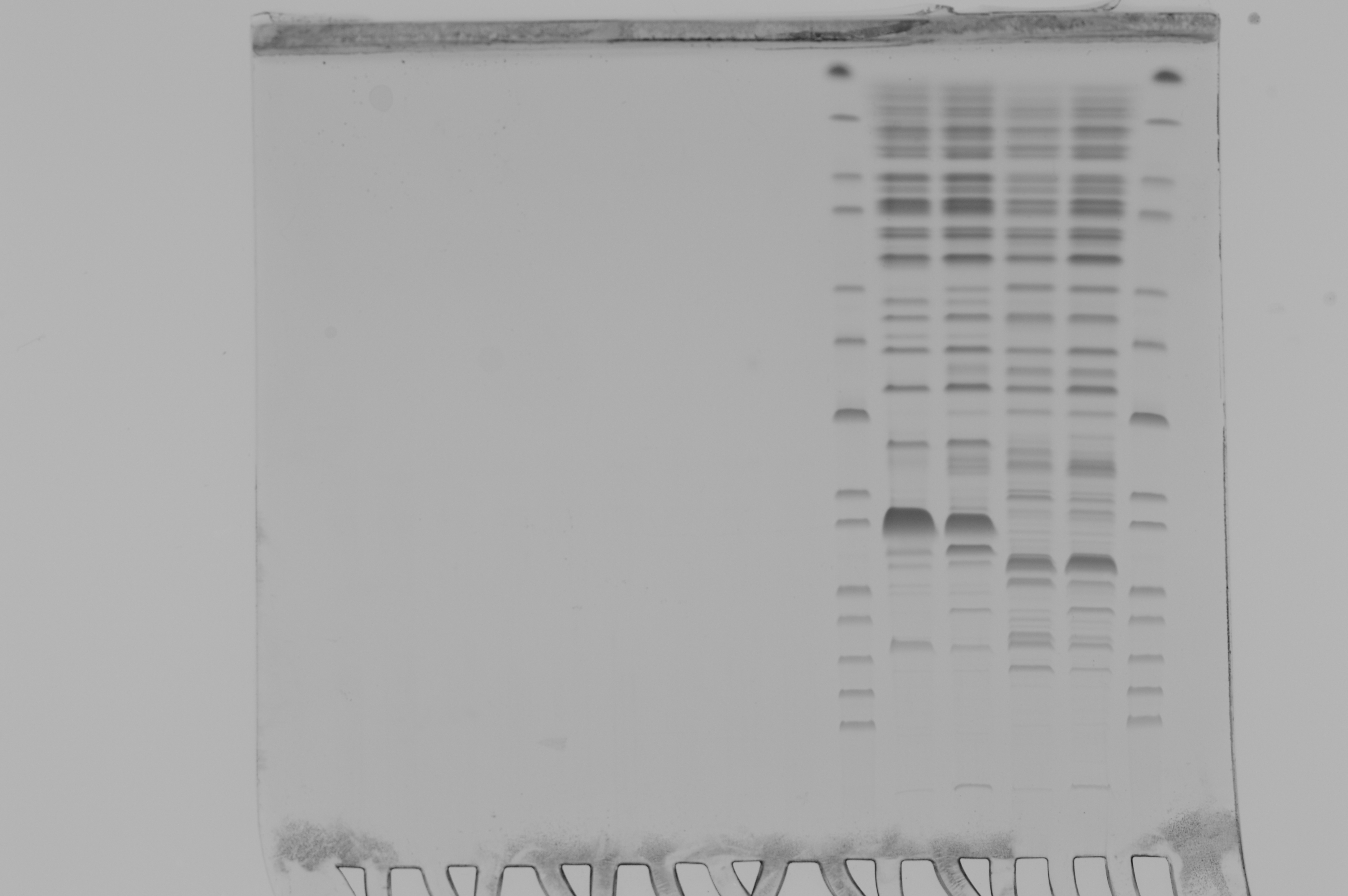

Supplement: Figure 2—source data 3. — Dashed boxes in the PDF indicate the respective areas shown in the figure. [file elife-84877-fig2-data3.zip › Figure2_Source_data_3/Figure2D_left_panel_Coomassie.JPG]

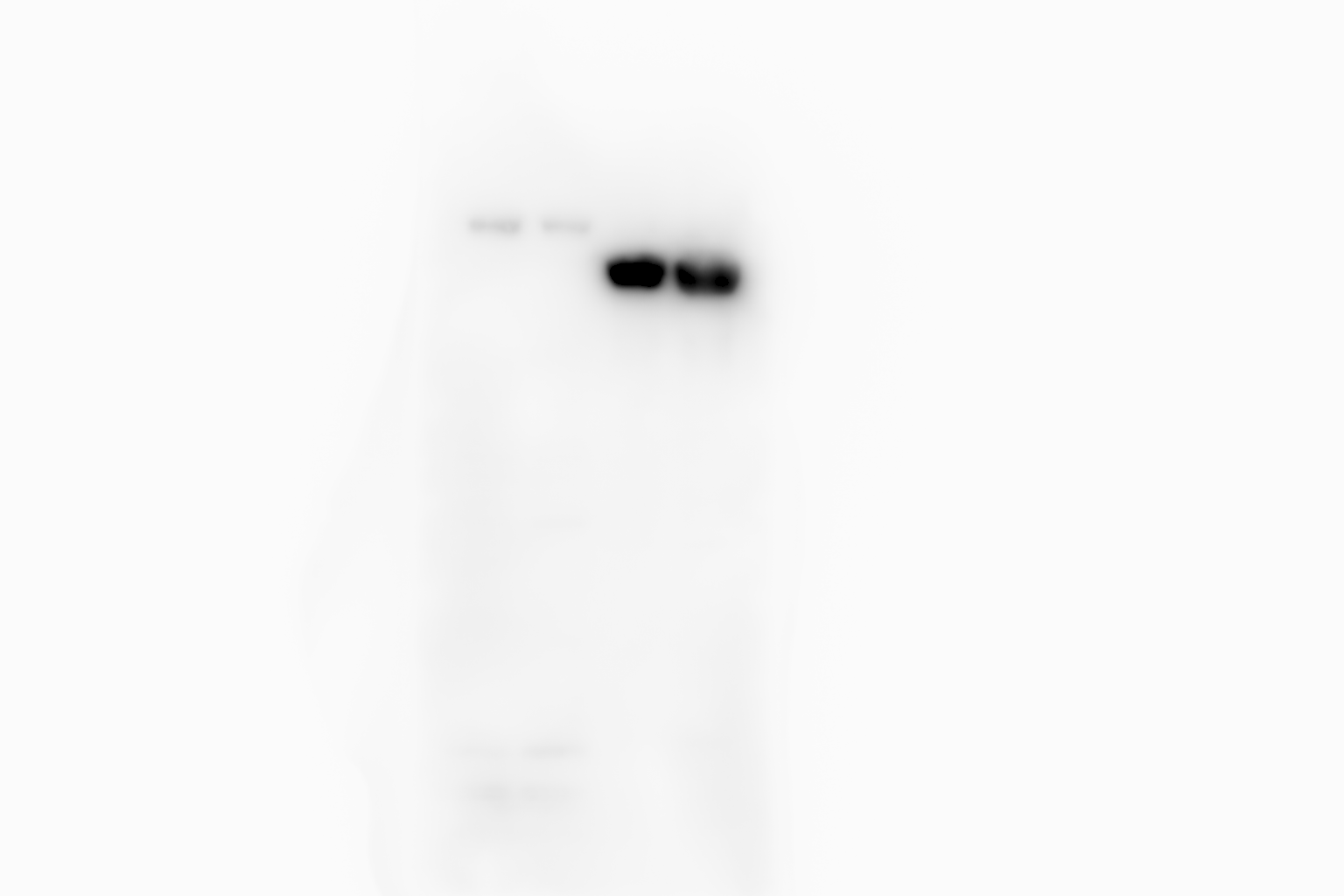

Supplement: Figure 2—source data 3. — Dashed boxes in the PDF indicate the respective areas shown in the figure. [file elife-84877-fig2-data3.zip › Figure2_Source_data_3/Figure2D_left_panel_Flag.tif]

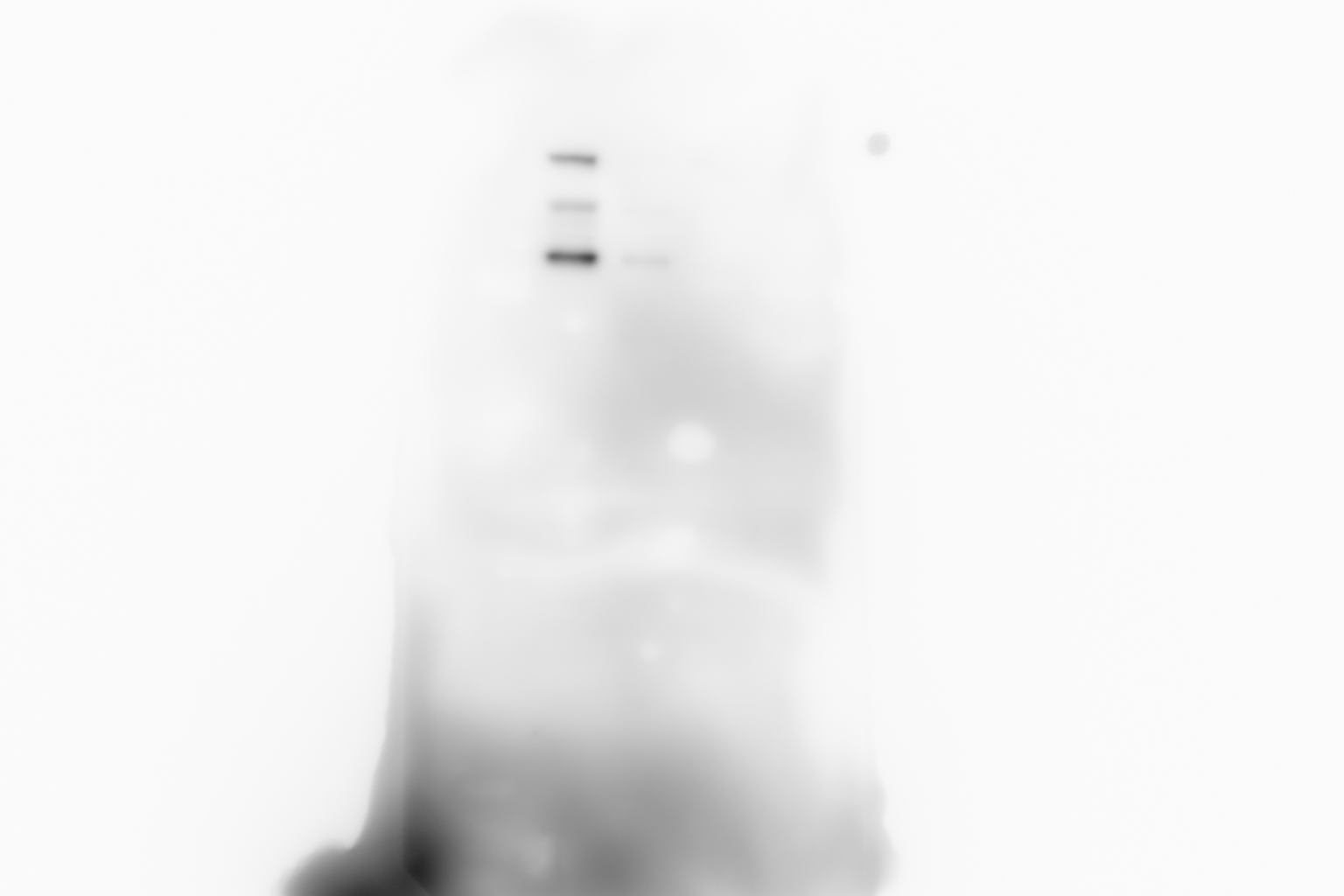

Supplement: Figure 2—source data 3. — Dashed boxes in the PDF indicate the respective areas shown in the figure. [file elife-84877-fig2-data3.zip › Figure2_Source_data_3/Figure2D_left_panel_HA.tif]

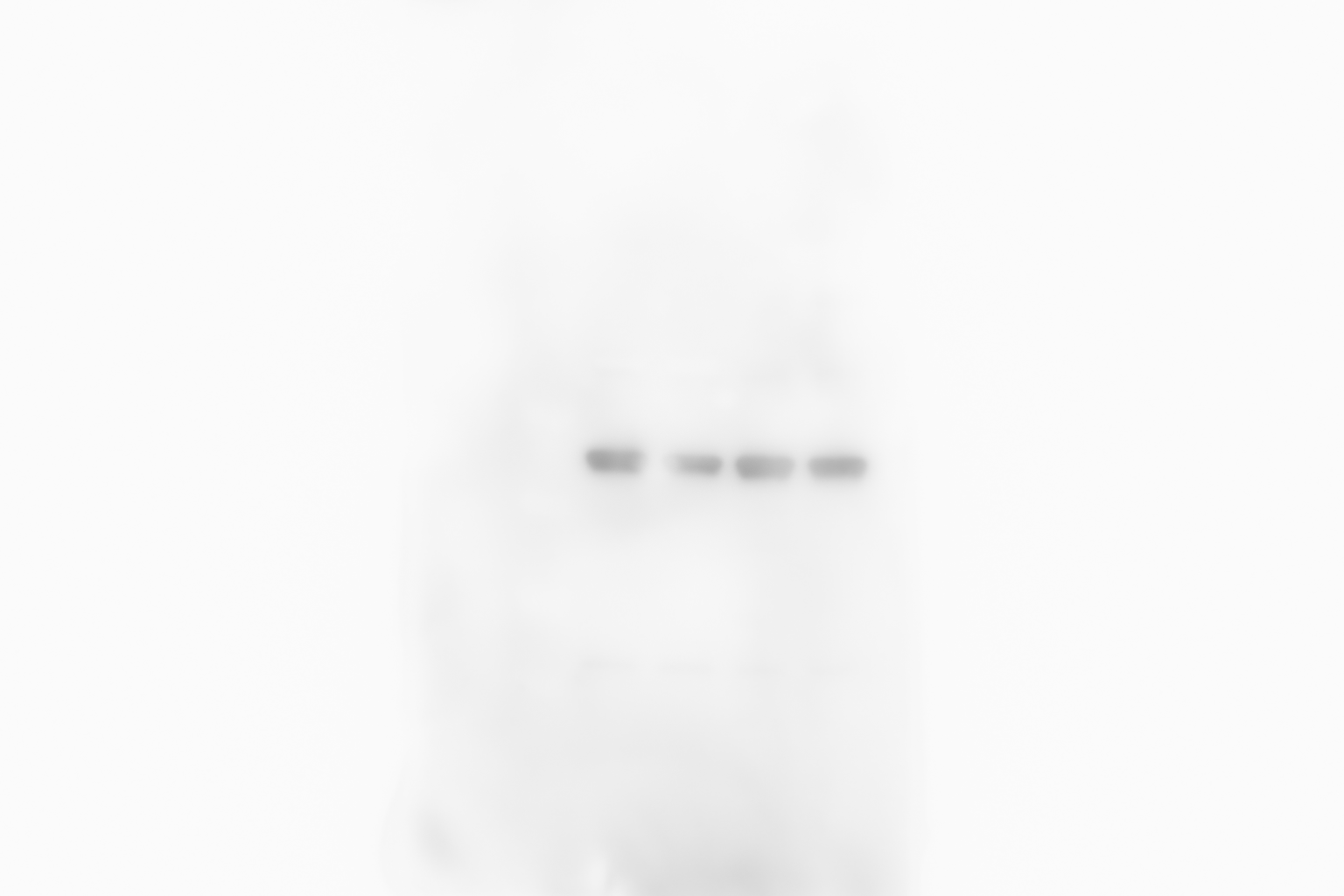

Supplement: Figure 2—source data 3. — Dashed boxes in the PDF indicate the respective areas shown in the figure. [file elife-84877-fig2-data3.zip › Figure2_Source_data_3/Figure2D_left_panel_L3.tif]

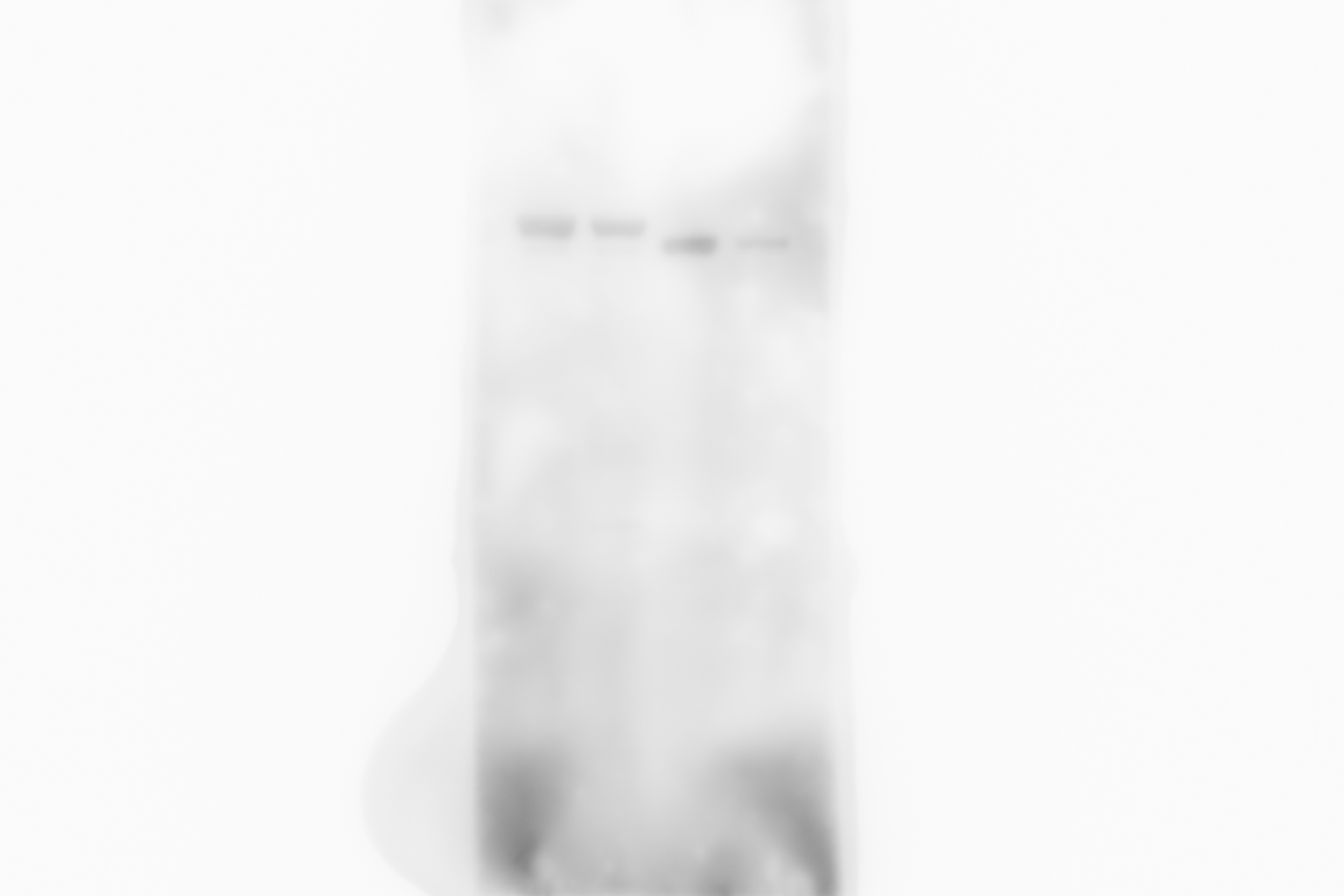

Supplement: Figure 2—source data 3. — Dashed boxes in the PDF indicate the respective areas shown in the figure. [file elife-84877-fig2-data3.zip › Figure2_Source_data_3/Figure2D_left_panel_Nog1.tif]

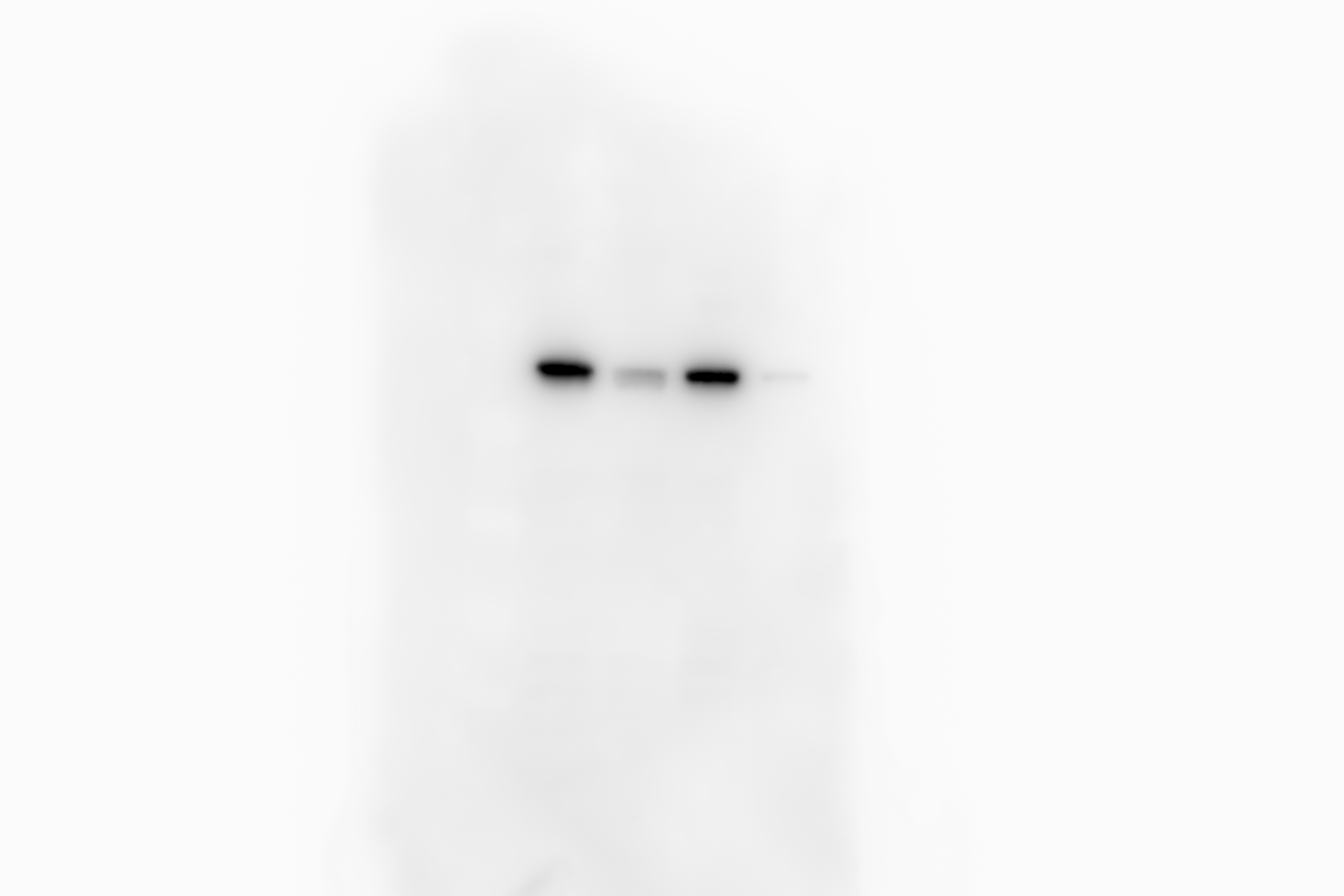

Supplement: Figure 2—source data 3. — Dashed boxes in the PDF indicate the respective areas shown in the figure. [file elife-84877-fig2-data3.zip › Figure2_Source_data_3/Figure2D_left_panel_Nog2.tif]

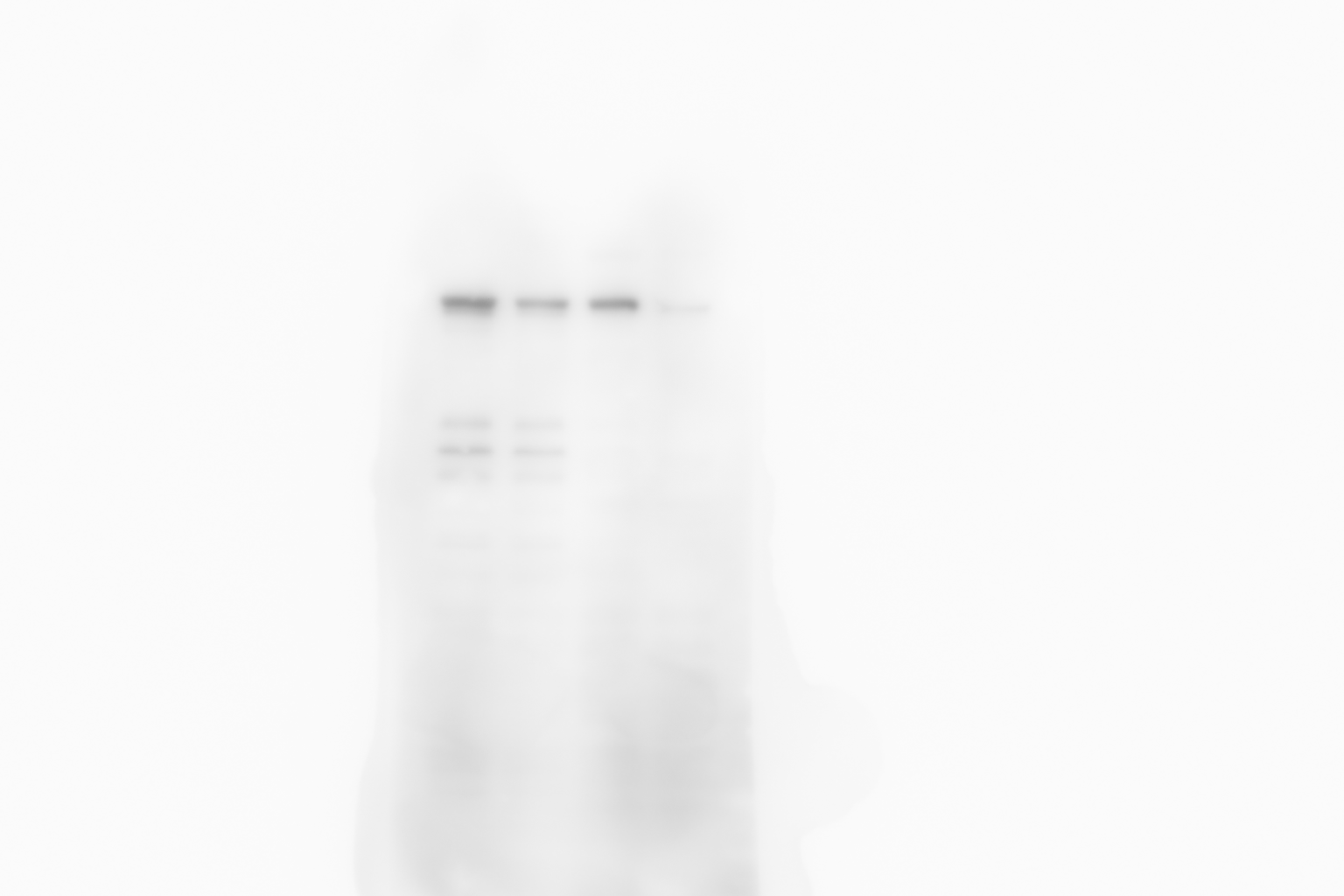

Supplement: Figure 2—source data 3. — Dashed boxes in the PDF indicate the respective areas shown in the figure. [file elife-84877-fig2-data3.zip › Figure2_Source_data_3/Figure2D_left_panel_Nug1.tif]

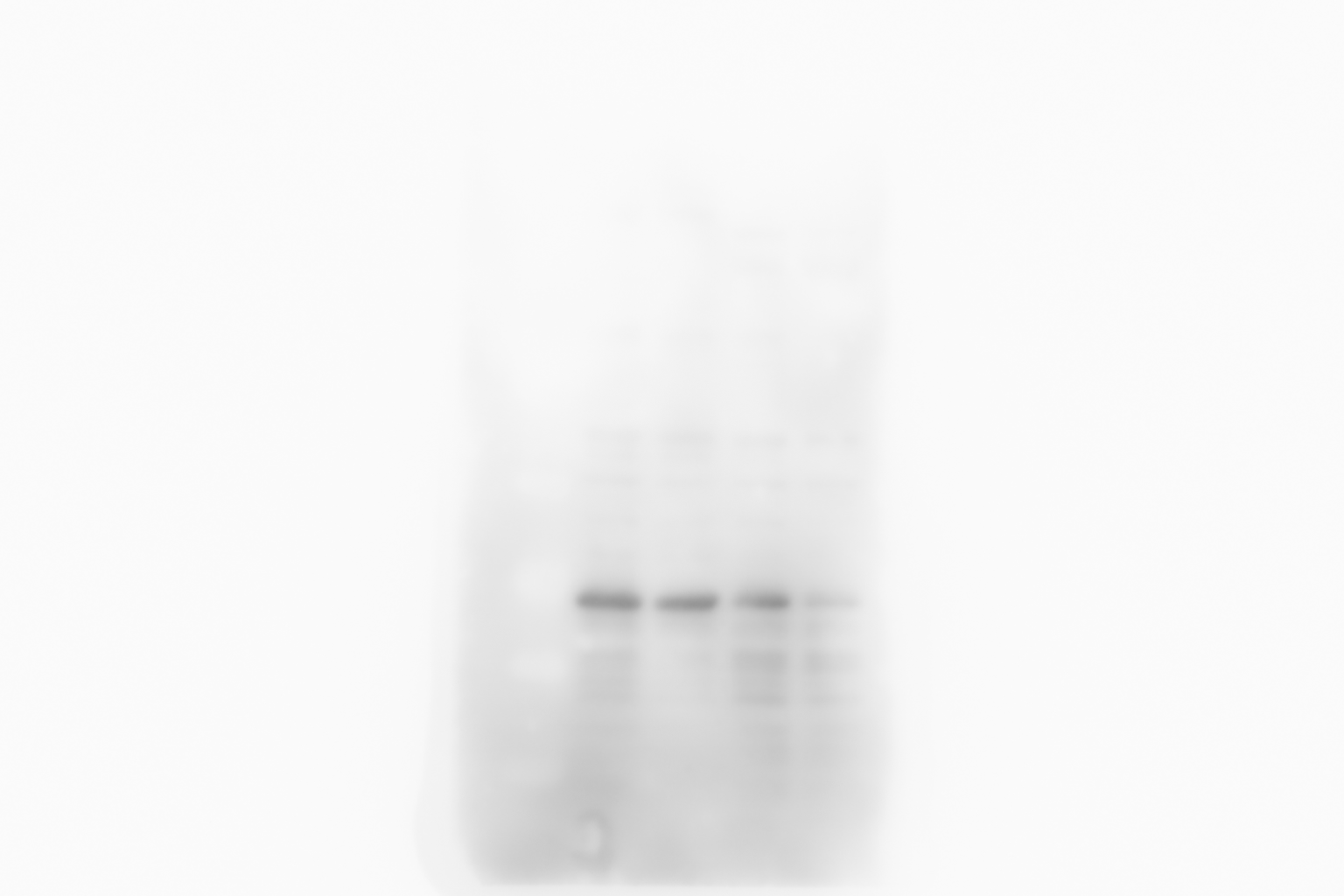

Supplement: Figure 2—source data 3. — Dashed boxes in the PDF indicate the respective areas shown in the figure. [file elife-84877-fig2-data3.zip › Figure2_Source_data_3/Figure2D_left_panel_Rlp24.tif]

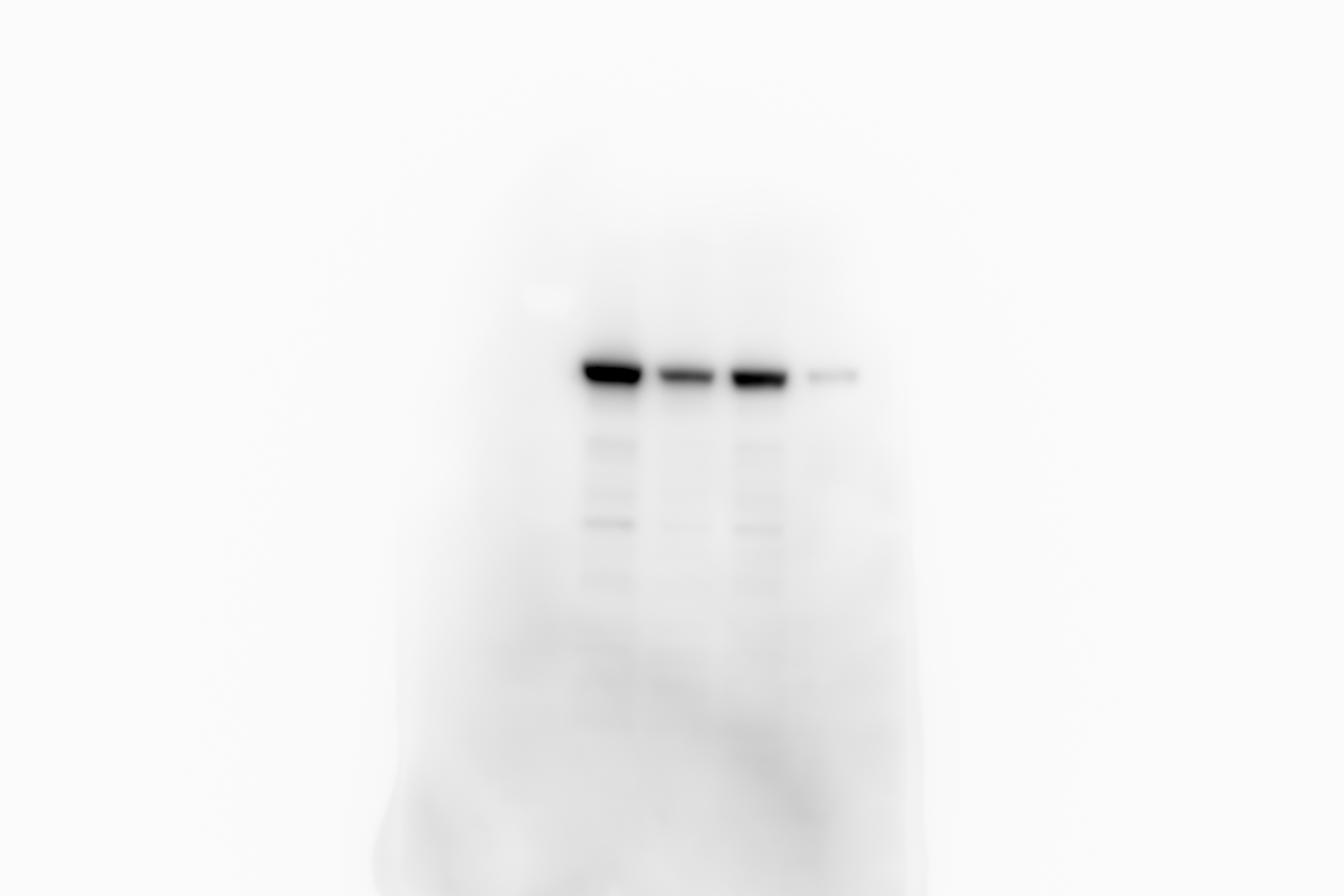

Supplement: Figure 2—source data 3. — Dashed boxes in the PDF indicate the respective areas shown in the figure. [file elife-84877-fig2-data3.zip › Figure2_Source_data_3/Figure2D_left_panel_Rsa4.tif]

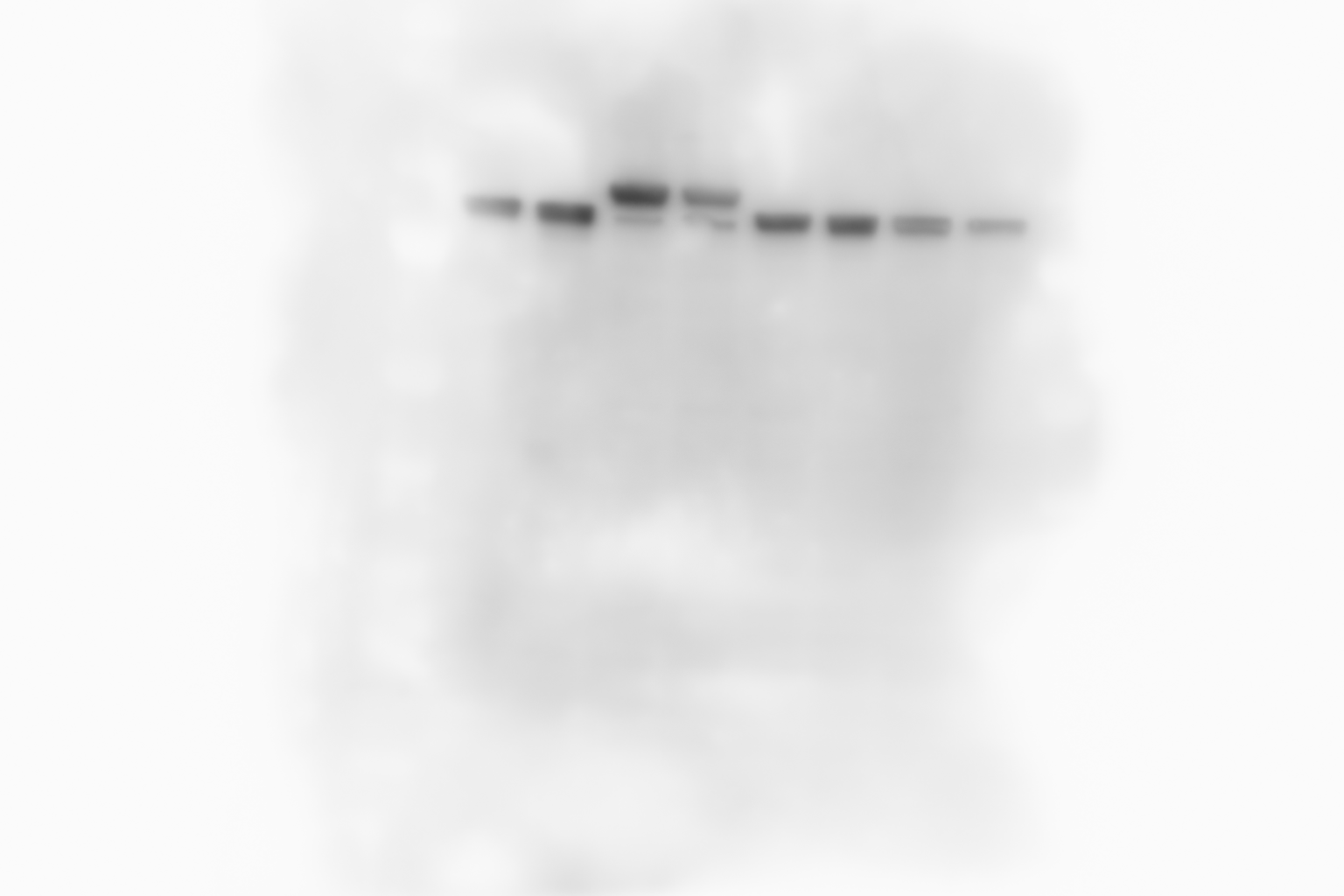

Supplement: Figure 2—source data 3. — Dashed boxes in the PDF indicate the respective areas shown in the figure. [file elife-84877-fig2-data3.zip › Figure2_Source_data_3/Figure2D_right_panel_Nog1.tif]

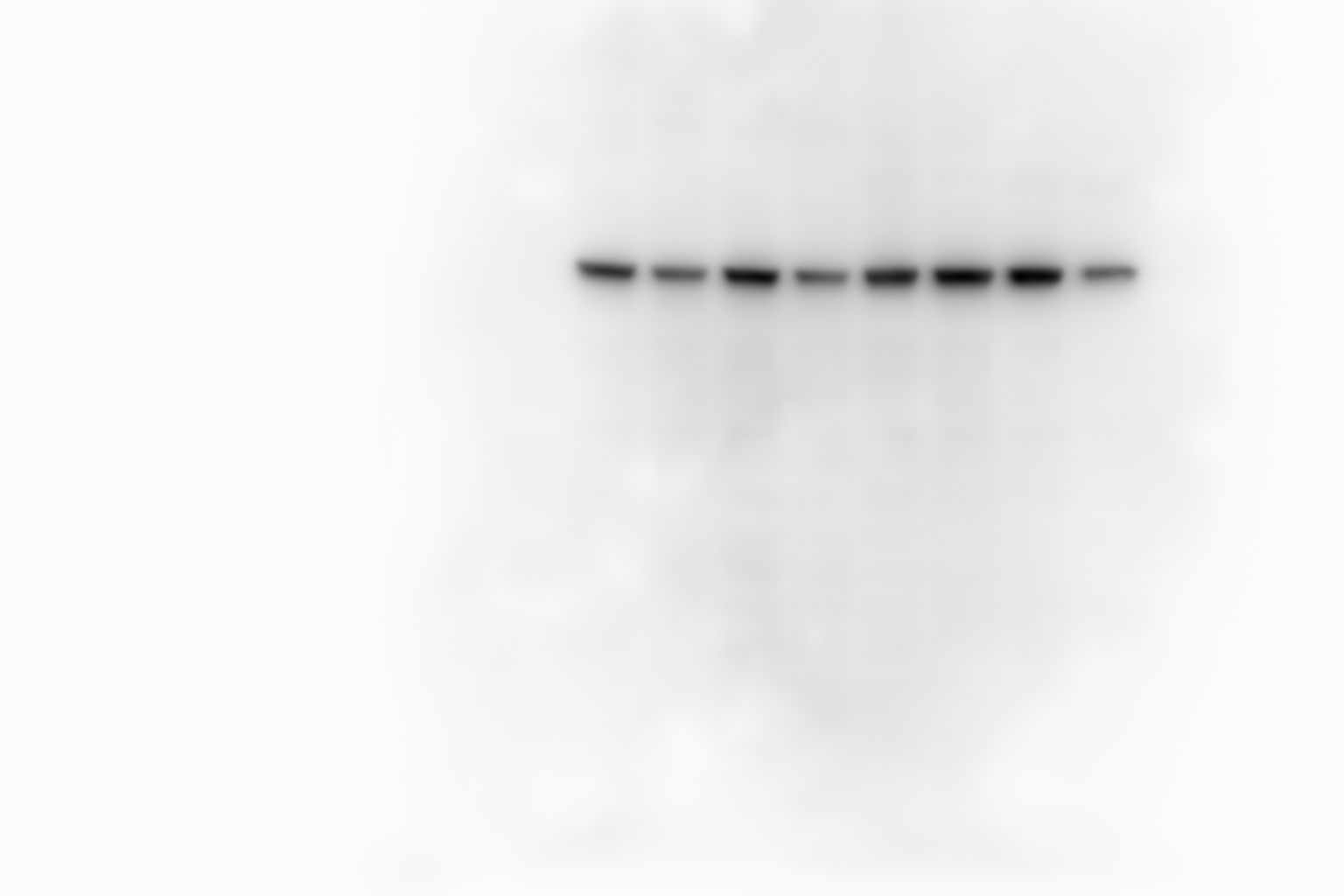

Supplement: Figure 2—source data 3. — Dashed boxes in the PDF indicate the respective areas shown in the figure. [file elife-84877-fig2-data3.zip › Figure2_Source_data_3/Figure2D_right_panel_Rsa4.tif]

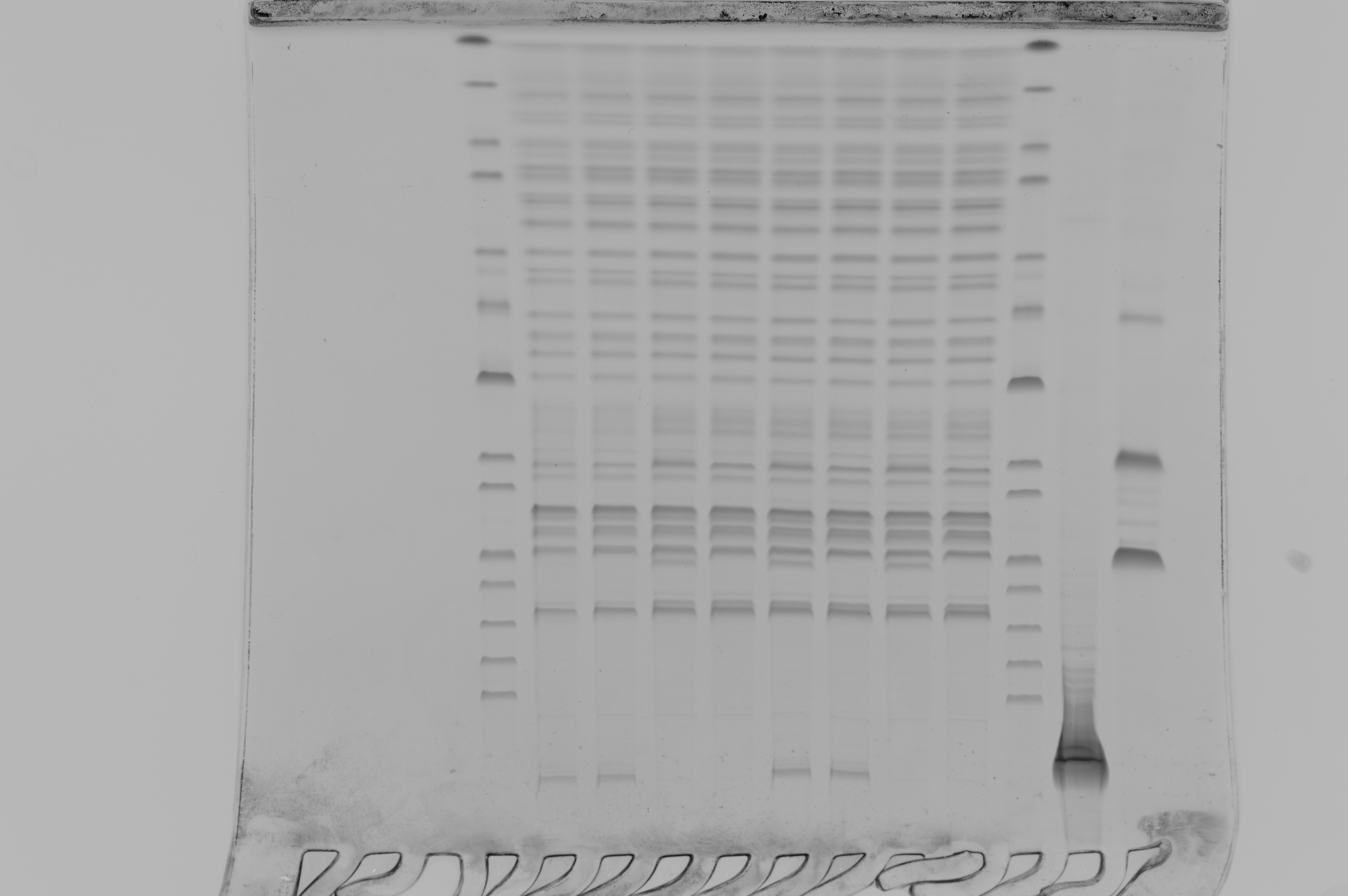

Supplement: Figure 4—source data 1. — Dashed boxes in the PDF indicate the respective areas shown in the figure. [file elife-84877-fig4-data1.zip › Figure4_Source_data/Figure4A_Coomassie.JPG]

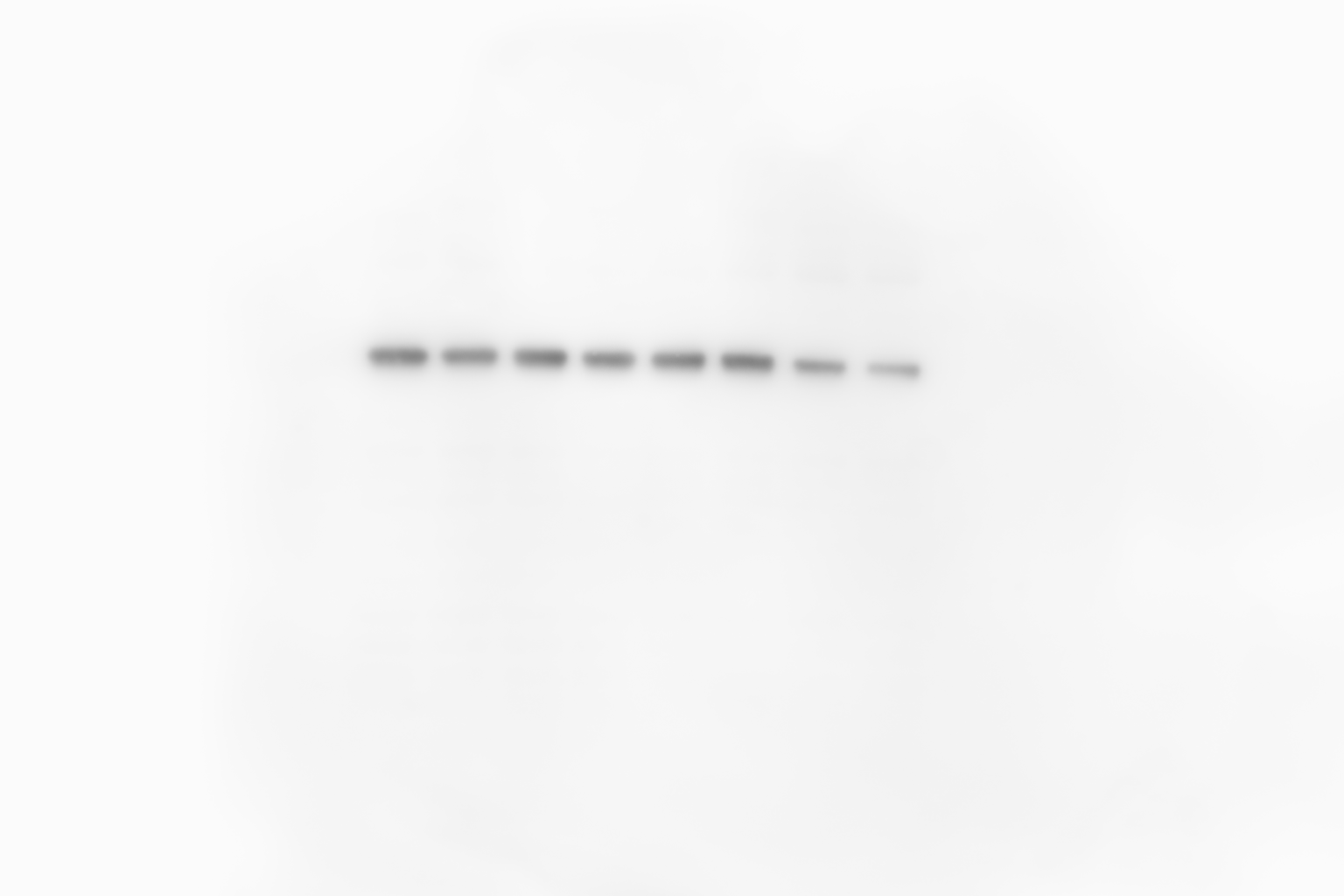

Supplement: Figure 4—source data 1. — Dashed boxes in the PDF indicate the respective areas shown in the figure. [file elife-84877-fig4-data1.zip › Figure4_Source_data/Figure4A_Has1.tif]

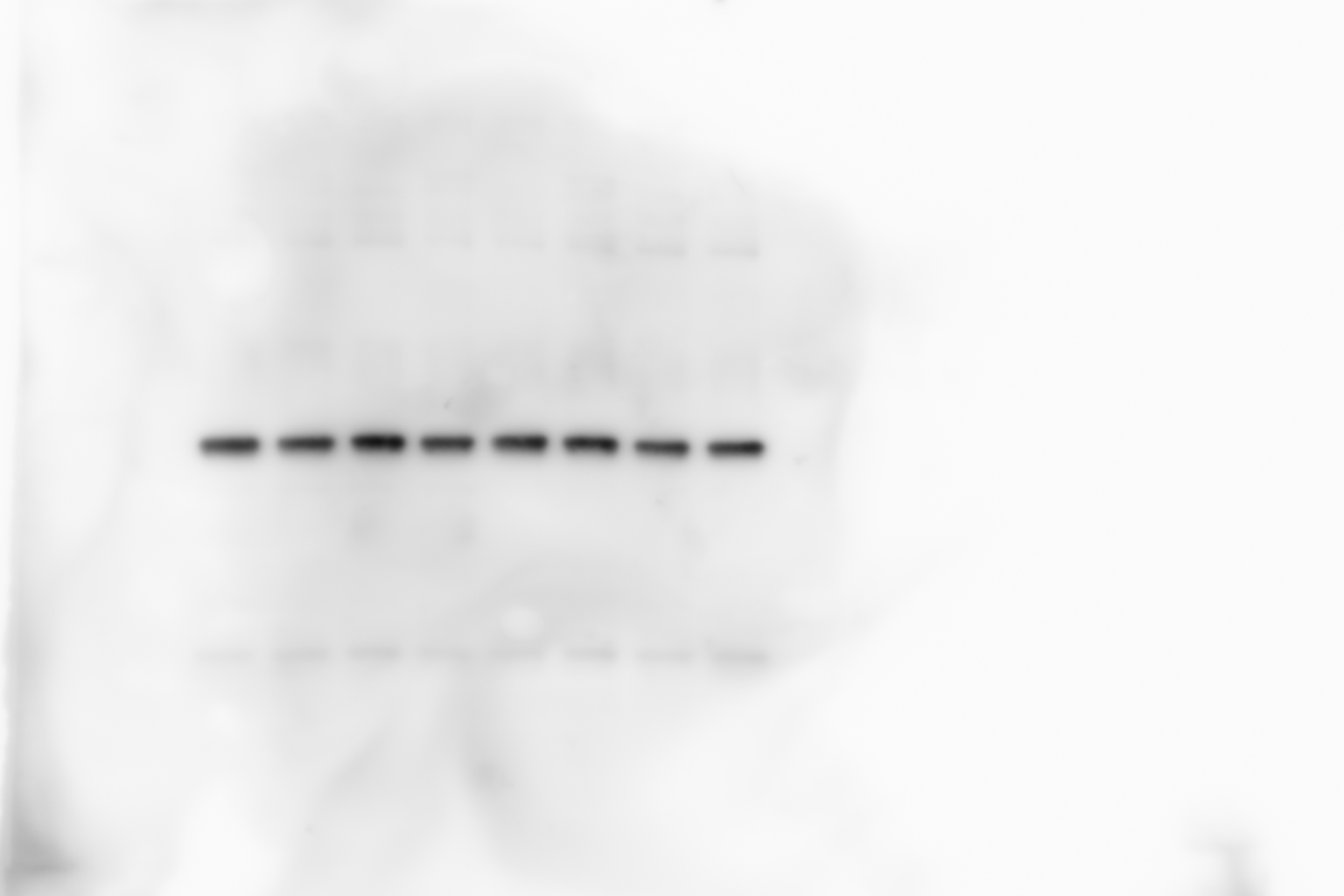

Supplement: Figure 4—source data 1. — Dashed boxes in the PDF indicate the respective areas shown in the figure. [file elife-84877-fig4-data1.zip › Figure4_Source_data/Figure4A_L3.tif]

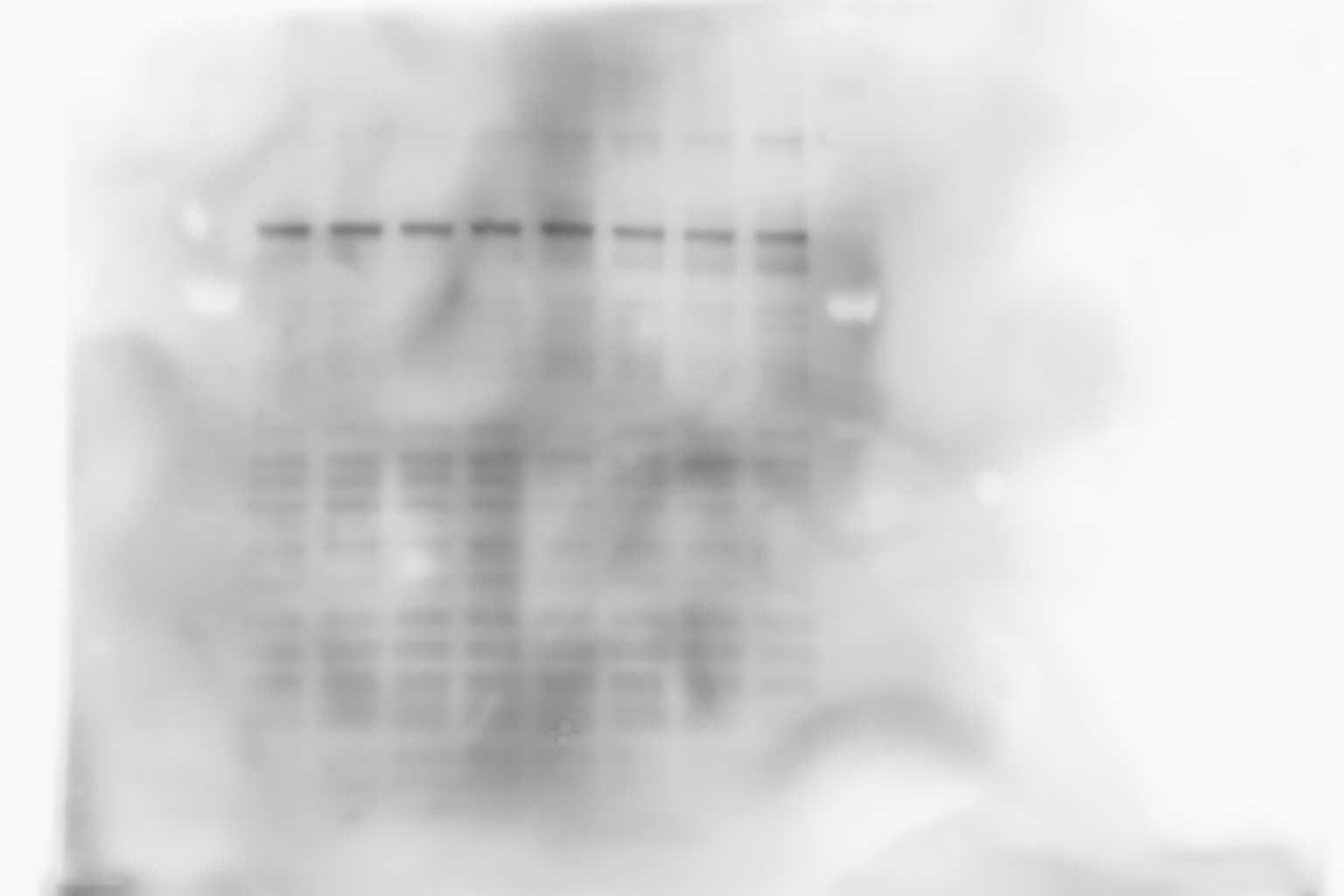

Supplement: Figure 4—source data 1. — Dashed boxes in the PDF indicate the respective areas shown in the figure. [file elife-84877-fig4-data1.zip › Figure4_Source_data/Figure4A_Noc3.tif]

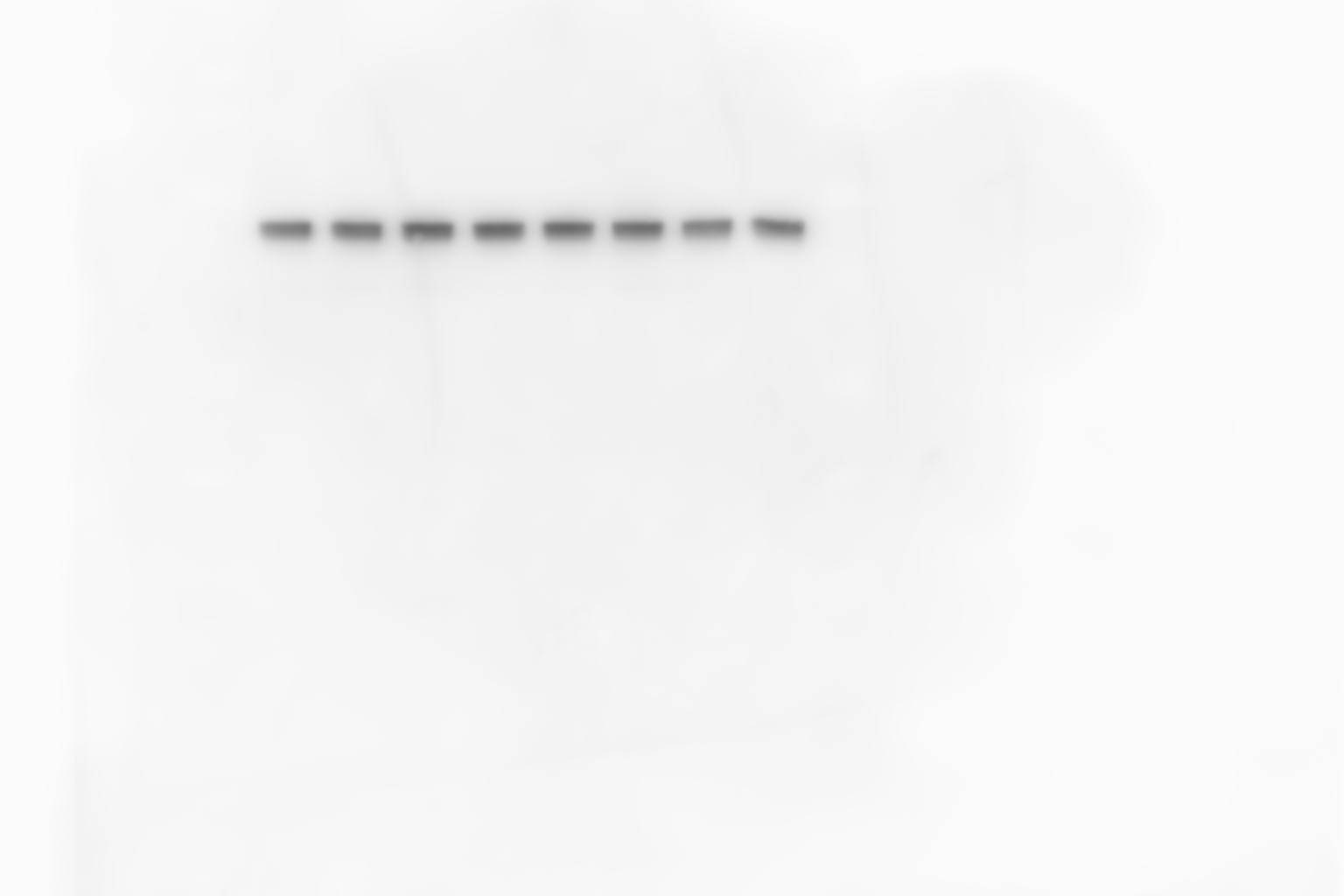

Supplement: Figure 4—source data 1. — Dashed boxes in the PDF indicate the respective areas shown in the figure. [file elife-84877-fig4-data1.zip › Figure4_Source_data/Figure4A_Nog1.tif]

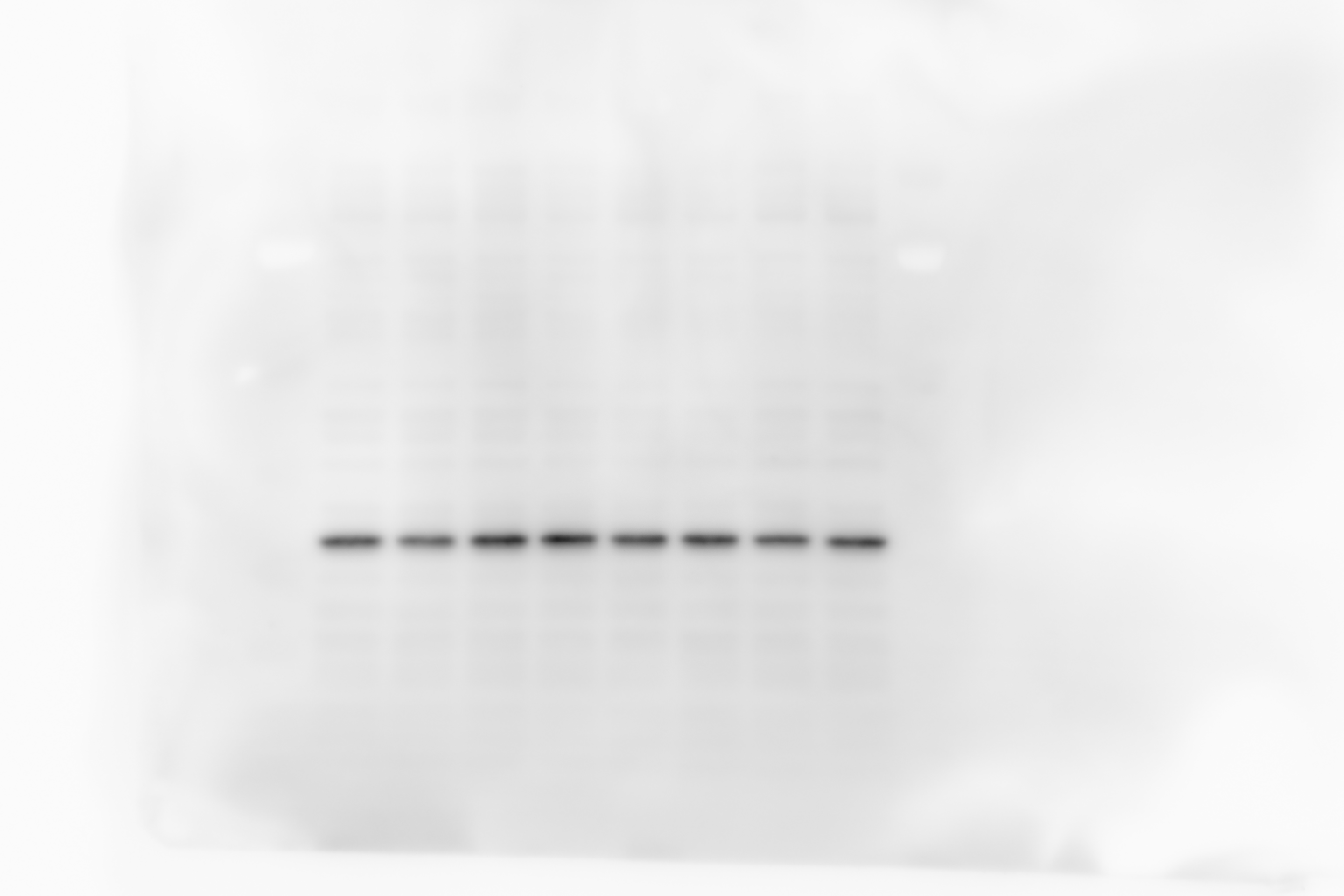

Supplement: Figure 4—source data 1. — Dashed boxes in the PDF indicate the respective areas shown in the figure. [file elife-84877-fig4-data1.zip › Figure4_Source_data/Figure4A_Nsa2.tif]

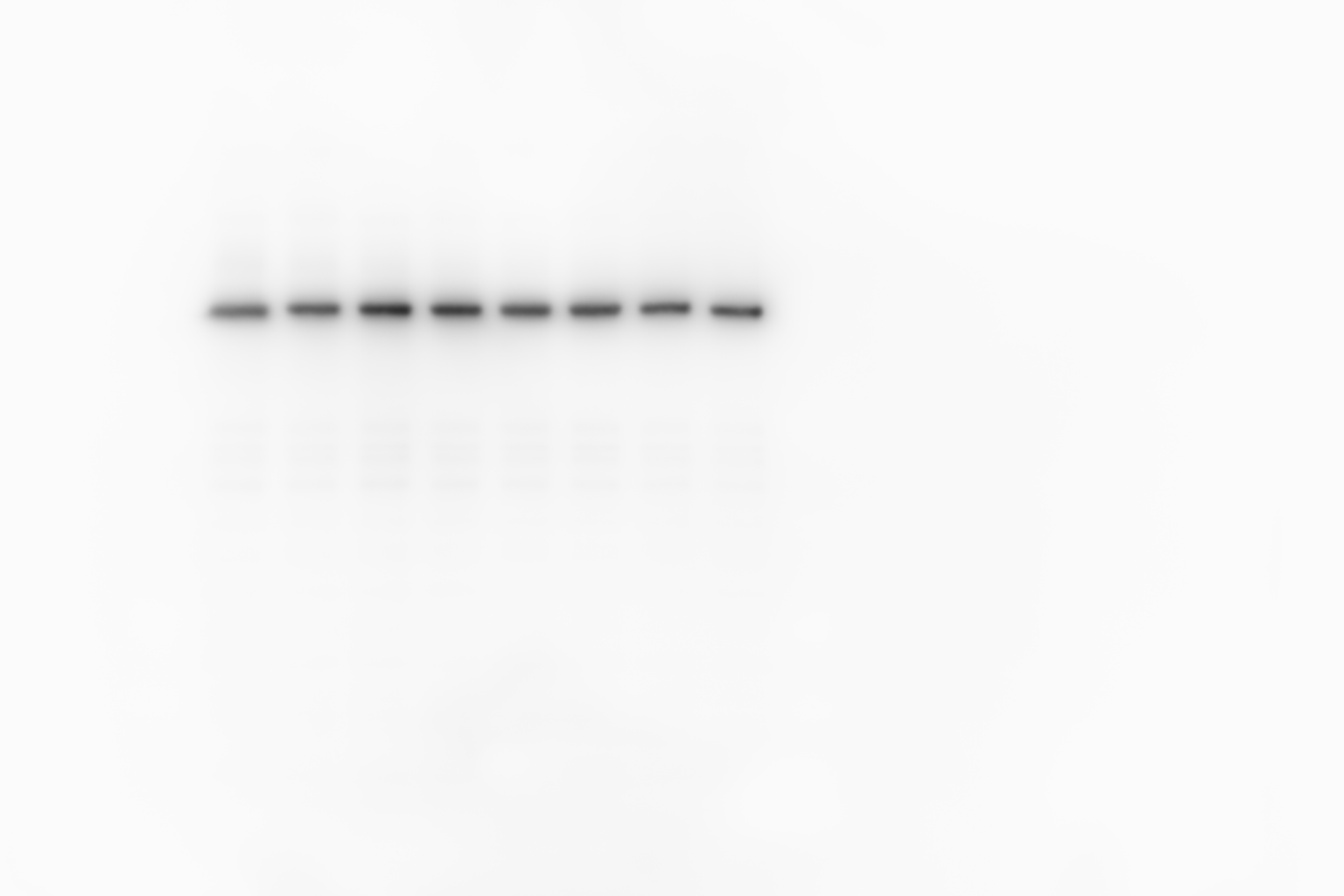

Supplement: Figure 4—source data 1. — Dashed boxes in the PDF indicate the respective areas shown in the figure. [file elife-84877-fig4-data1.zip › Figure4_Source_data/Figure4A_Nug1.tif]

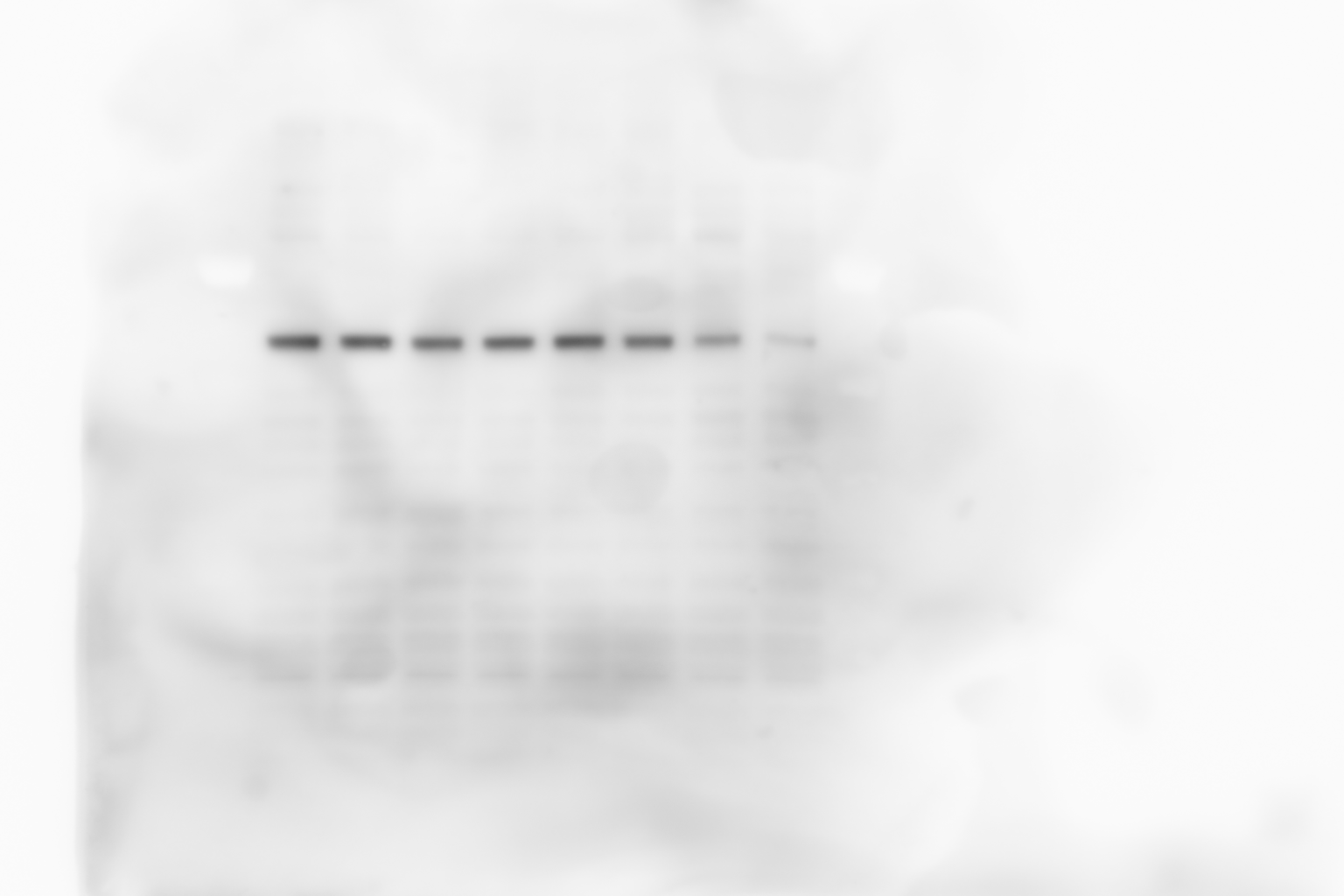

Supplement: Figure 4—source data 1. — Dashed boxes in the PDF indicate the respective areas shown in the figure. [file elife-84877-fig4-data1.zip › Figure4_Source_data/Figure4A_Ytm1.tif]

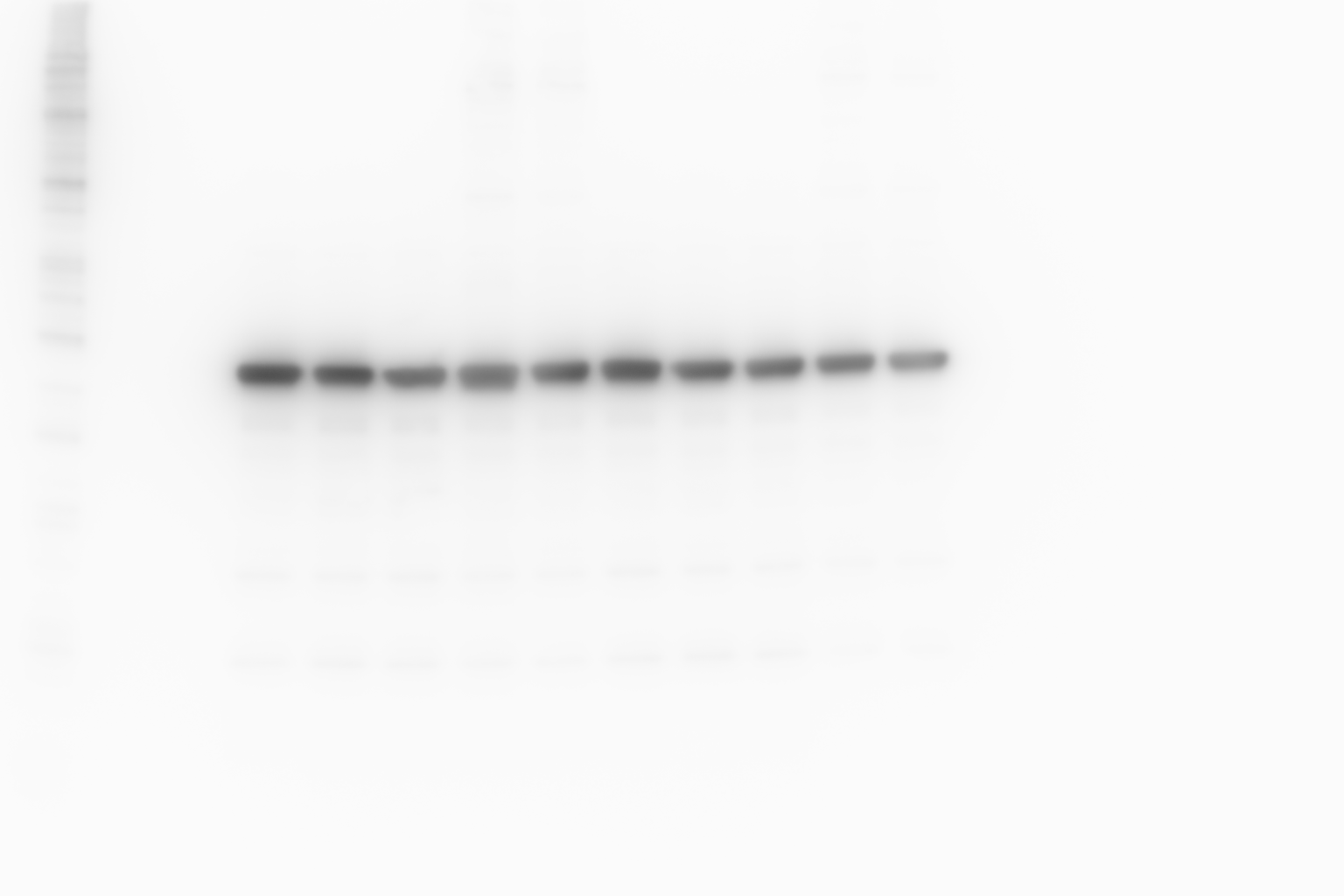

Supplement: Figure 4—figure supplement 1—source data 1. — Dashed boxes in the PDF indicate the respective areas shown in the figure. [file elife-84877-fig4-figsupp1-data1.zip › Figure4_Figure_Supplement1_Source_data1/Figure4_Figure_Supplement1A_anti-HA.tif]

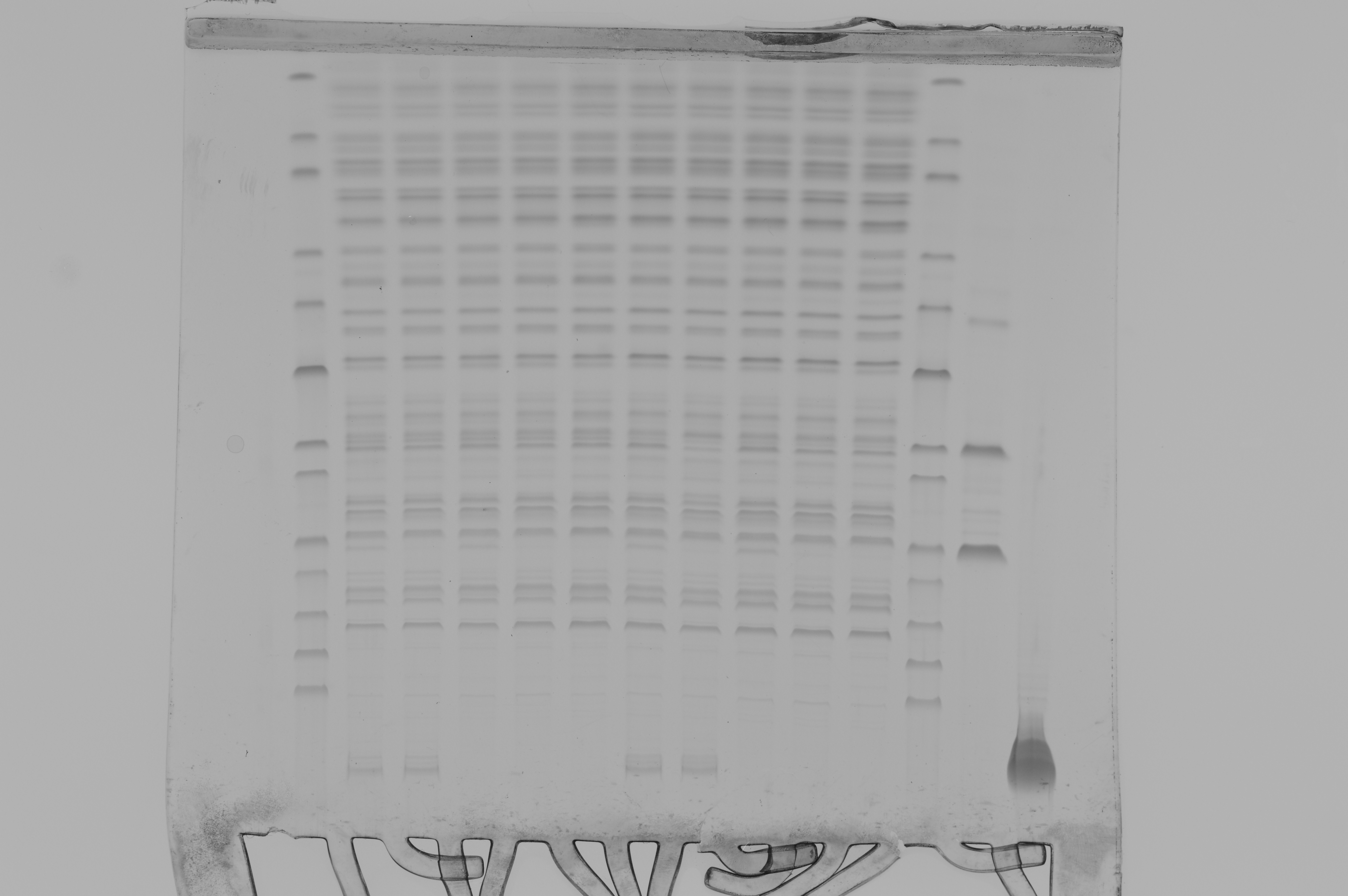

Supplement: Figure 4—figure supplement 1—source data 1. — Dashed boxes in the PDF indicate the respective areas shown in the figure. [file elife-84877-fig4-figsupp1-data1.zip › Figure4_Figure_Supplement1_Source_data1/Figure4_Figure_Supplement1A_Coomassie.JPG]

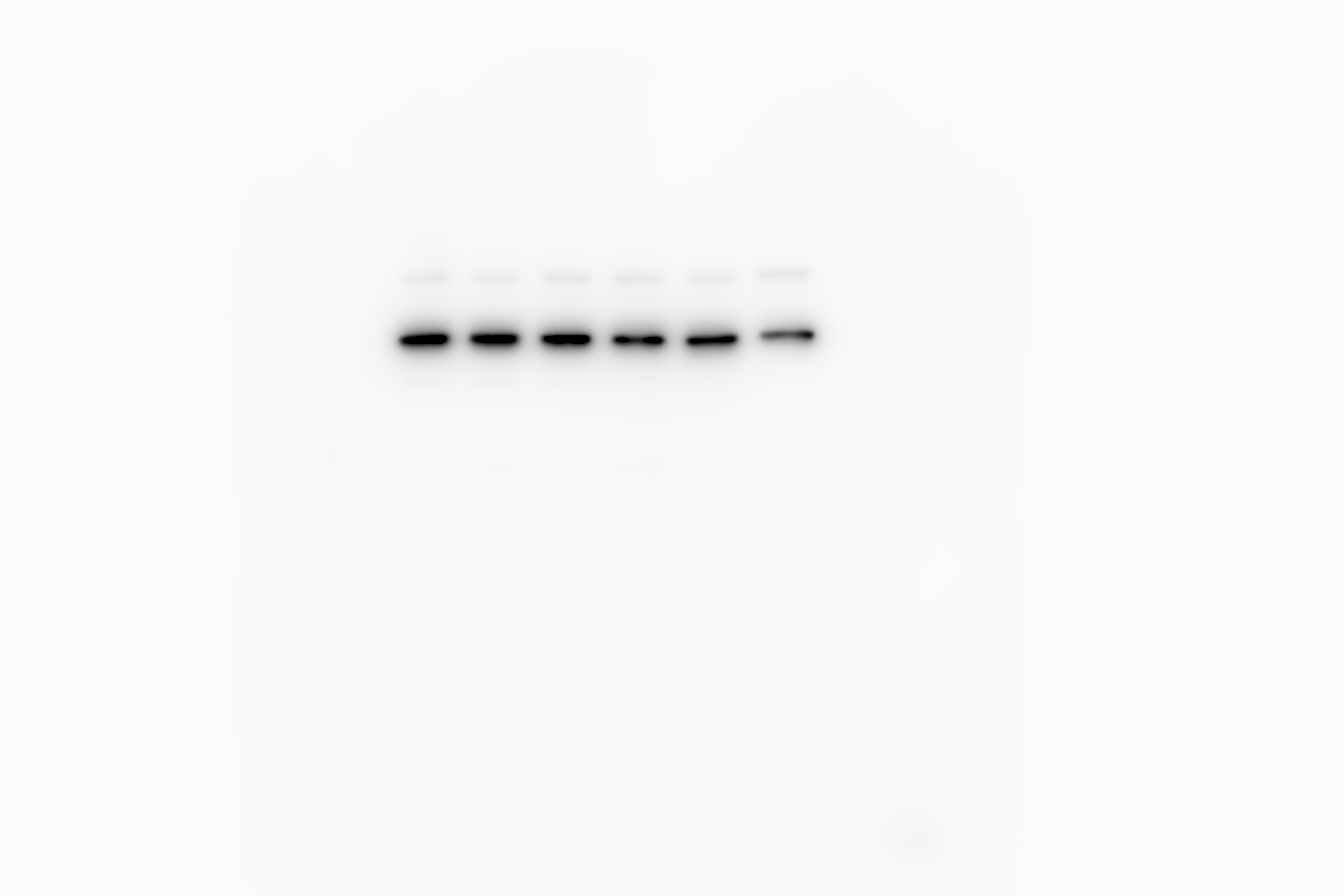

Supplement: Figure 4—figure supplement 1—source data 1. — Dashed boxes in the PDF indicate the respective areas shown in the figure. [file elife-84877-fig4-figsupp1-data1.zip › Figure4_Figure_Supplement1_Source_data1/Figure4_Figure_Supplement1B_anti-Ebp2.tif]

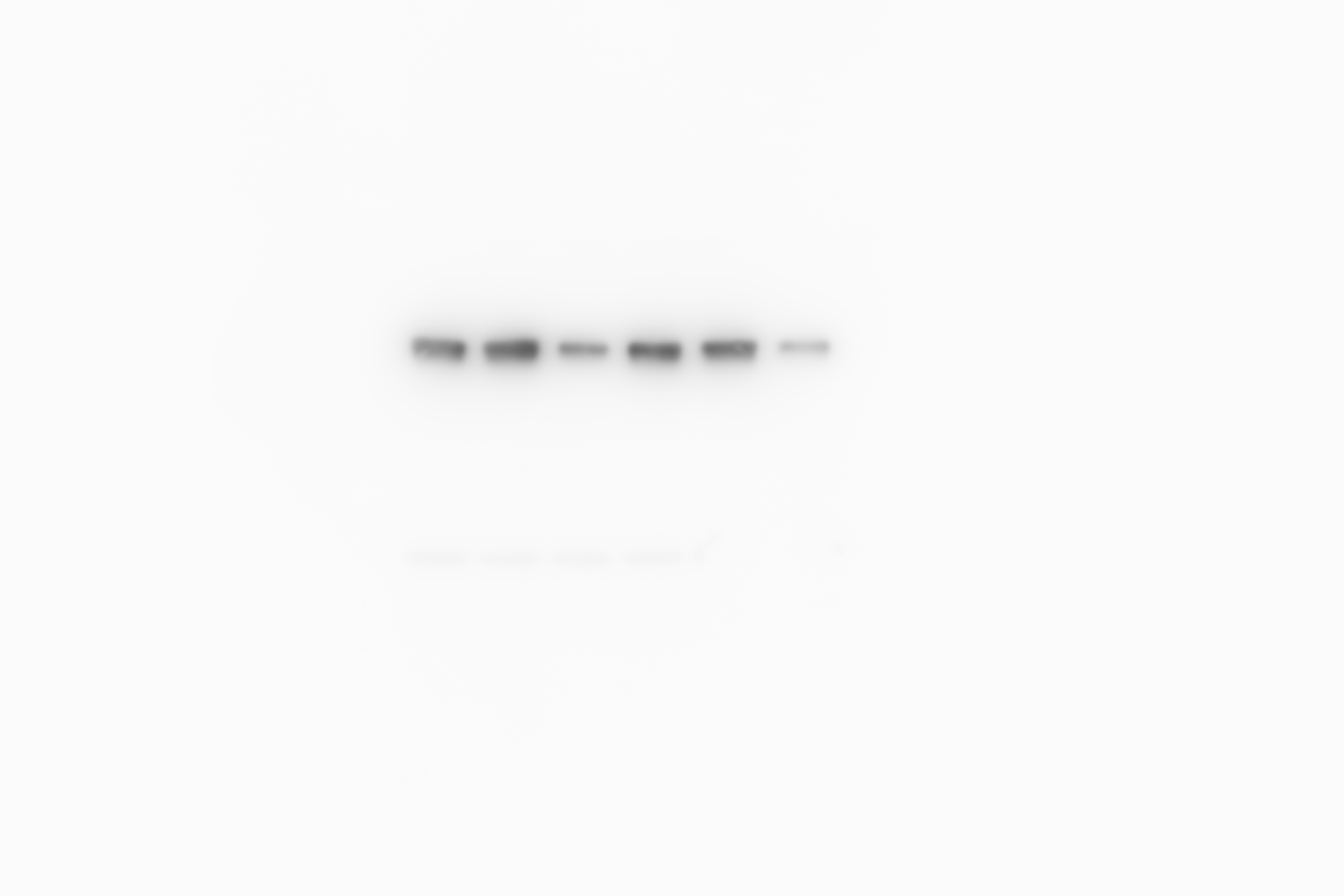

Supplement: Figure 4—figure supplement 1—source data 1. — Dashed boxes in the PDF indicate the respective areas shown in the figure. [file elife-84877-fig4-figsupp1-data1.zip › Figure4_Figure_Supplement1_Source_data1/Figure4_Figure_Supplement1B_anti-Has1.tif]

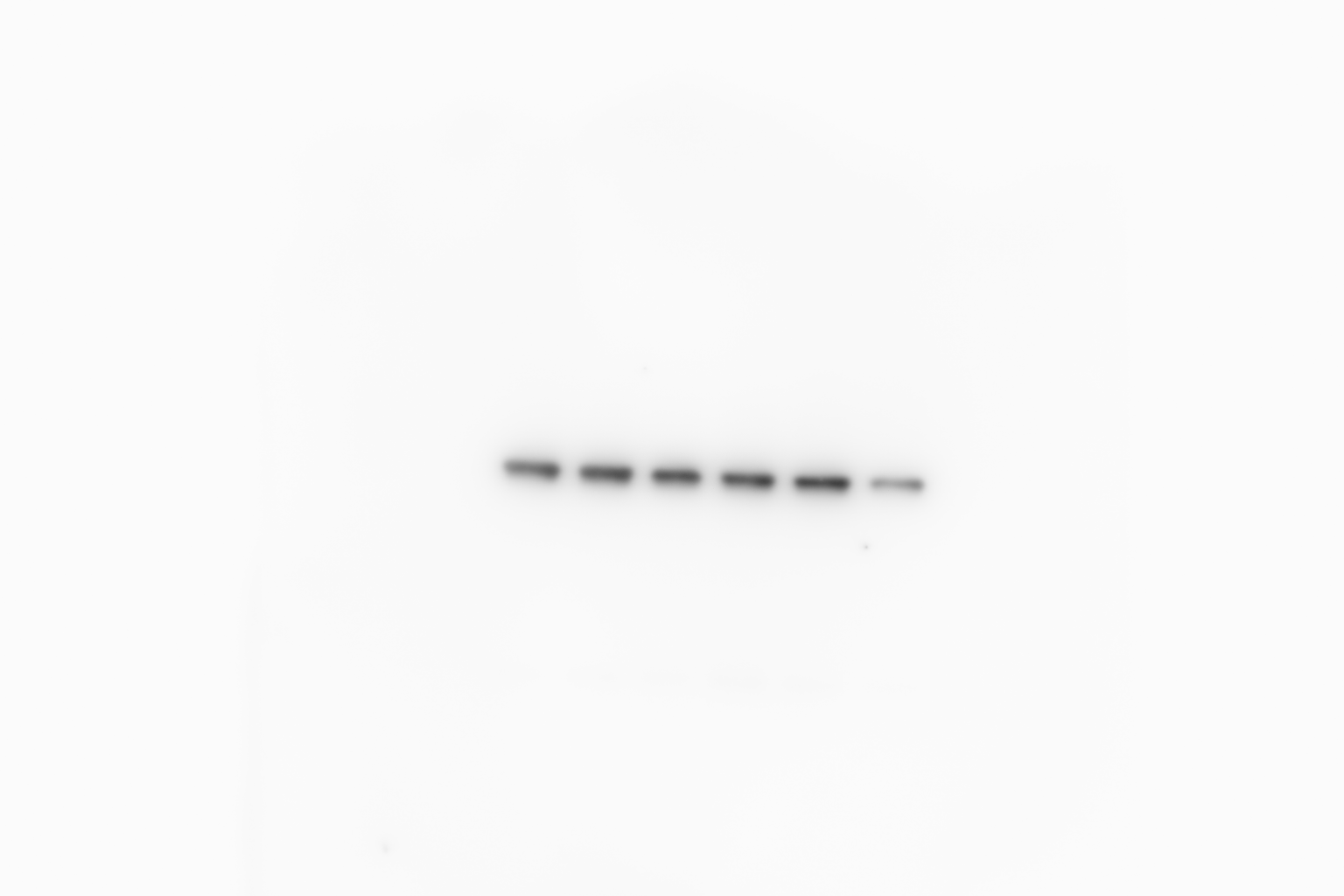

Supplement: Figure 4—figure supplement 1—source data 1. — Dashed boxes in the PDF indicate the respective areas shown in the figure. [file elife-84877-fig4-figsupp1-data1.zip › Figure4_Figure_Supplement1_Source_data1/Figure4_Figure_Supplement1B_anti-L3.tif]

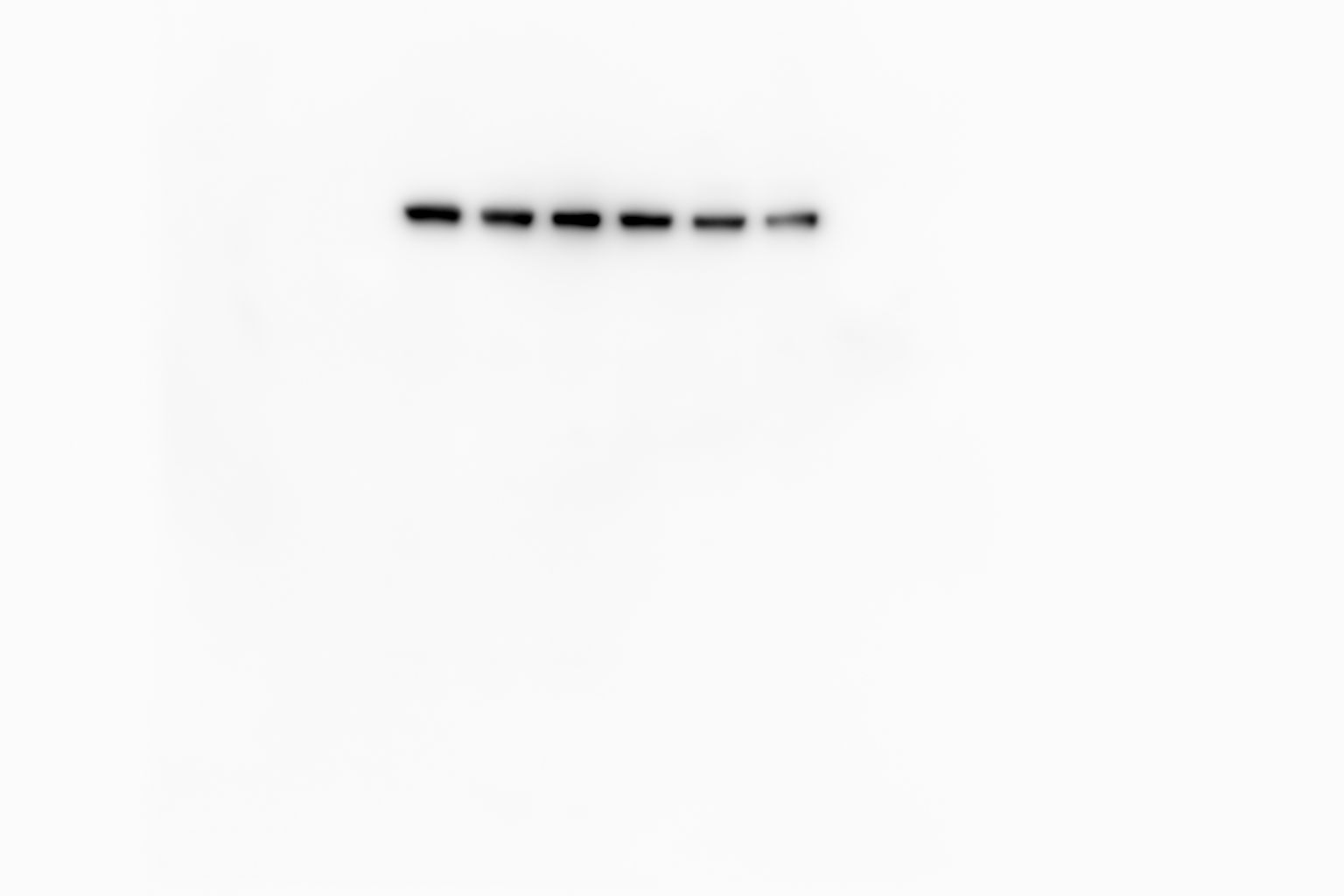

Supplement: Figure 4—figure supplement 1—source data 1. — Dashed boxes in the PDF indicate the respective areas shown in the figure. [file elife-84877-fig4-figsupp1-data1.zip › Figure4_Figure_Supplement1_Source_data1/Figure4_Figure_Supplement1B_anti-Noc3.tif]

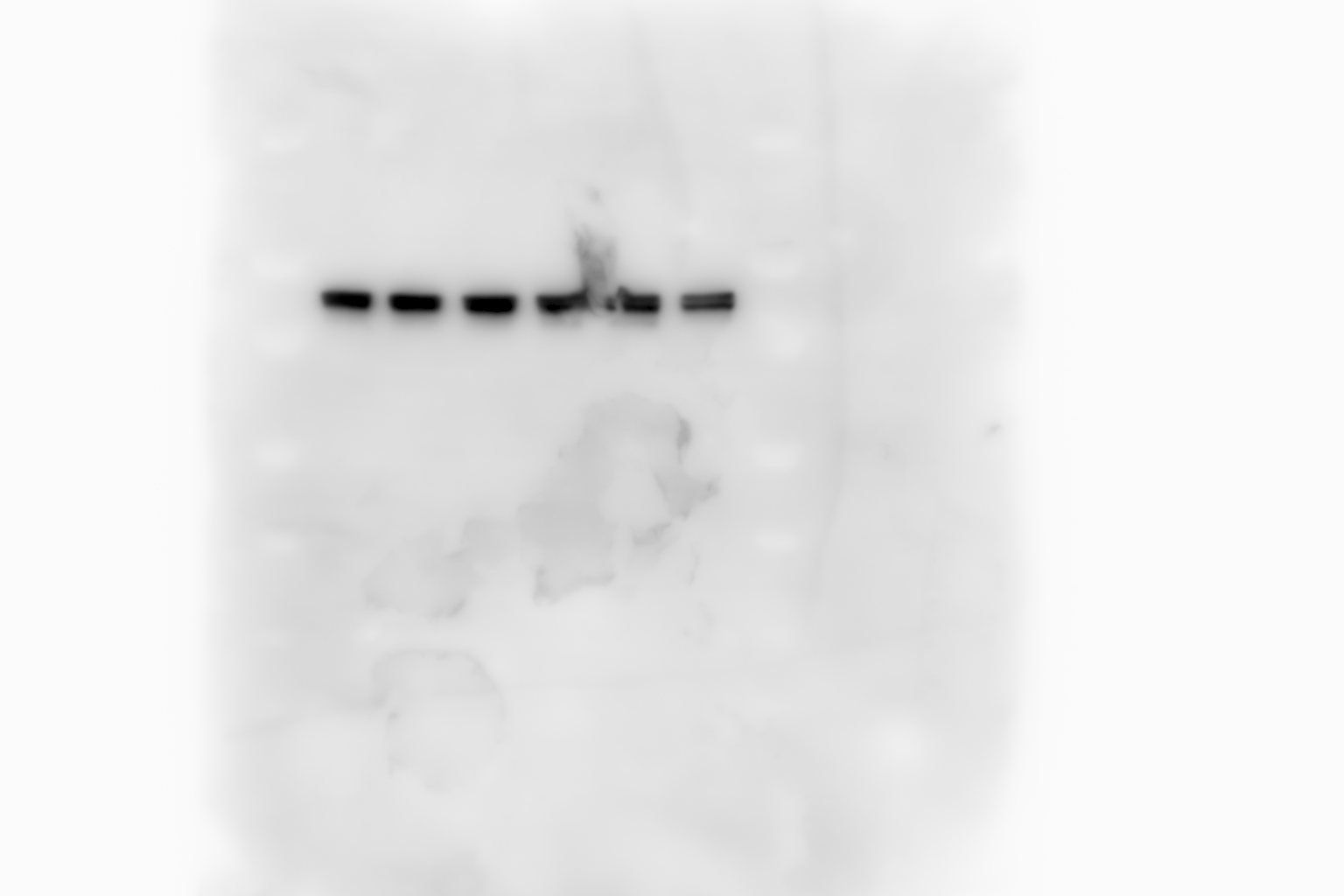

Supplement: Figure 4—figure supplement 1—source data 1. — Dashed boxes in the PDF indicate the respective areas shown in the figure. [file elife-84877-fig4-figsupp1-data1.zip › Figure4_Figure_Supplement1_Source_data1/Figure4_Figure_Supplement1B_anti-Nog1.tif]

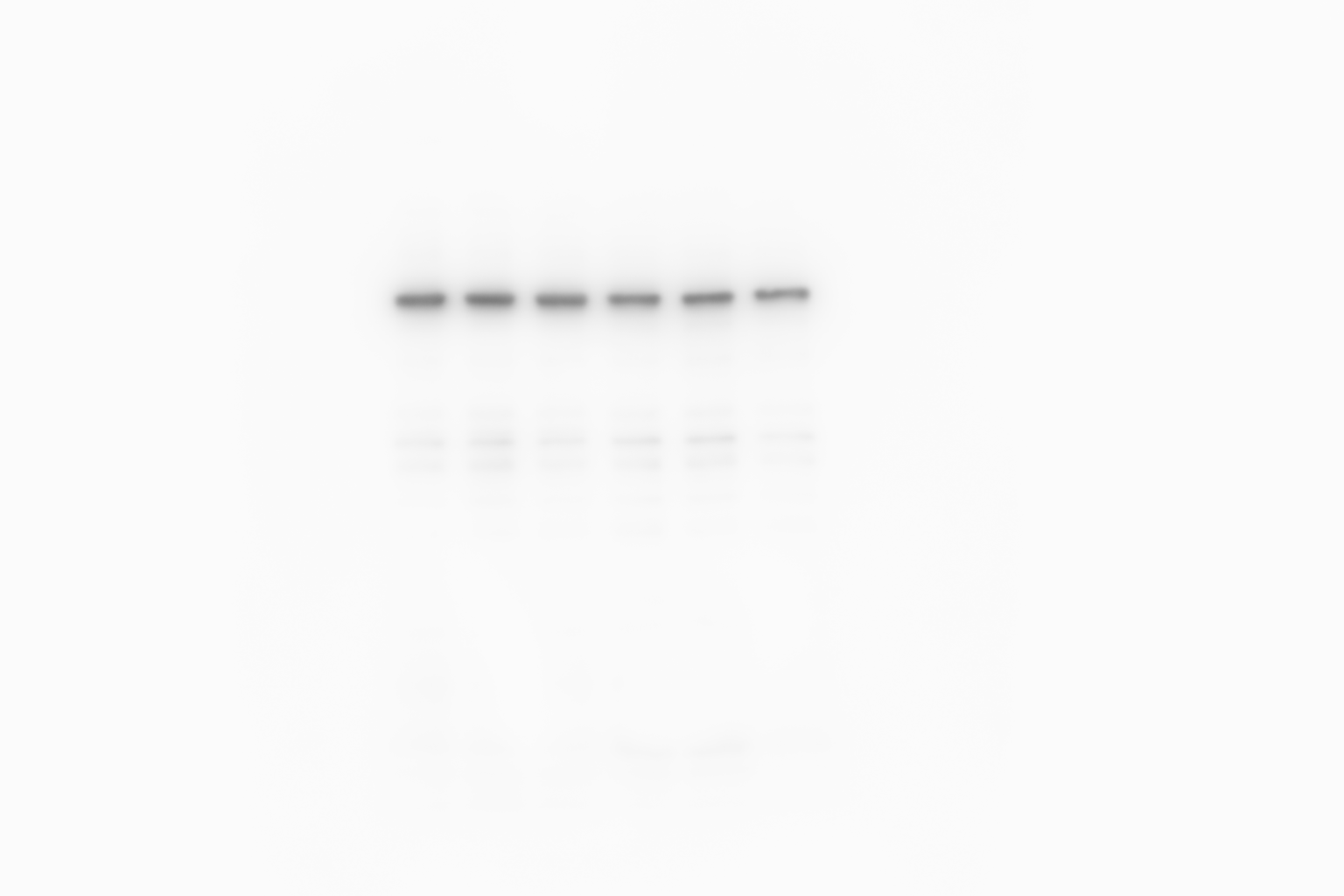

Supplement: Figure 4—figure supplement 1—source data 1. — Dashed boxes in the PDF indicate the respective areas shown in the figure. [file elife-84877-fig4-figsupp1-data1.zip › Figure4_Figure_Supplement1_Source_data1/Figure4_Figure_Supplement1B_anti-Nug1.tif]

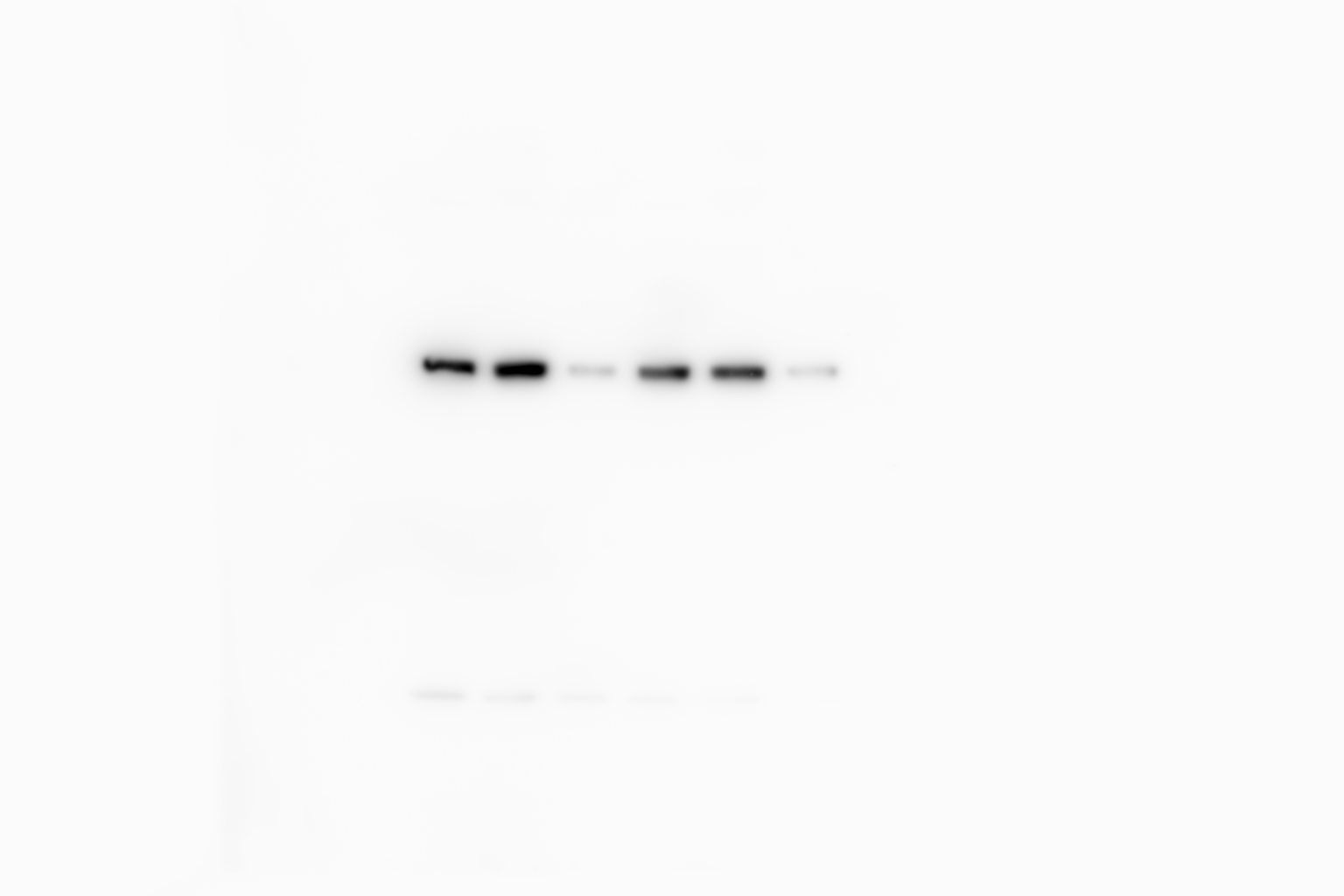

Supplement: Figure 4—figure supplement 1—source data 1. — Dashed boxes in the PDF indicate the respective areas shown in the figure. [file elife-84877-fig4-figsupp1-data1.zip › Figure4_Figure_Supplement1_Source_data1/Figure4_Figure_Supplement1B_anti-Ytm1.tif]

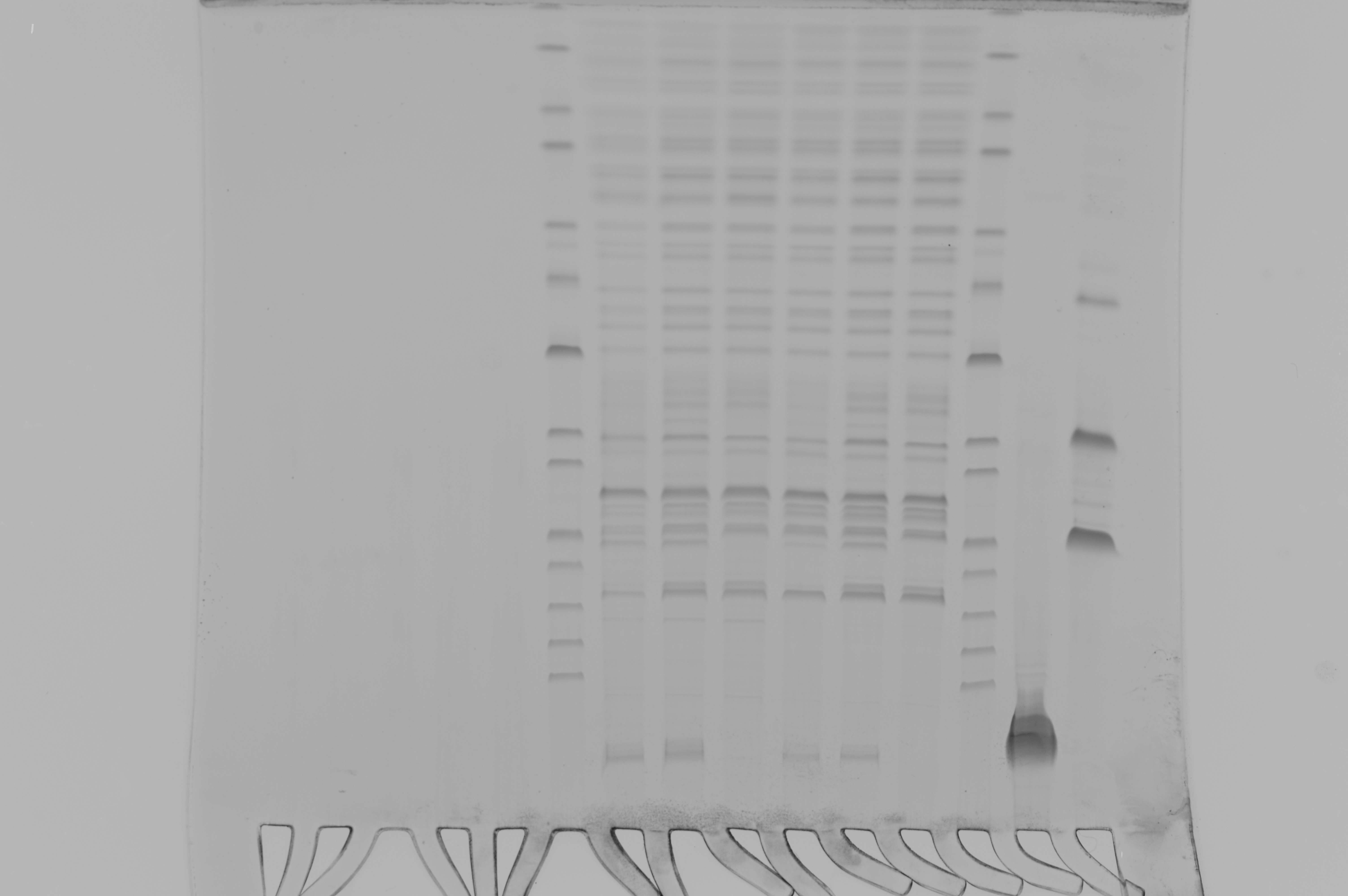

Supplement: Figure 4—figure supplement 1—source data 1. — Dashed boxes in the PDF indicate the respective areas shown in the figure. [file elife-84877-fig4-figsupp1-data1.zip › Figure4_Figure_Supplement1_Source_data1/Figure4_Figure_Supplement1B_Coomassie.JPG]

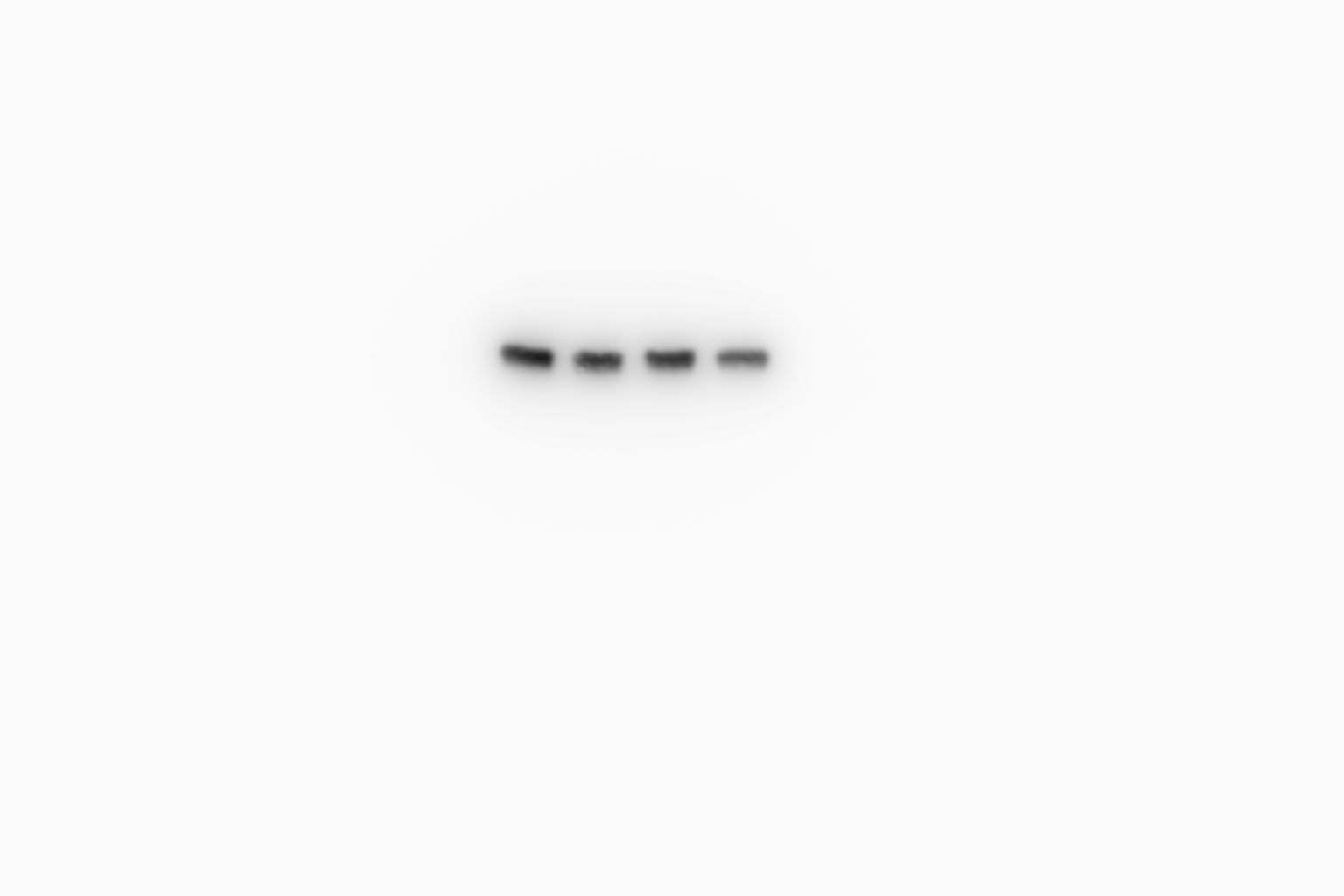

Supplement: Figure 4—figure supplement 1—source data 1. — Dashed boxes in the PDF indicate the respective areas shown in the figure. [file elife-84877-fig4-figsupp1-data1.zip › Figure4_Figure_Supplement1_Source_data1/Figure4_Figure_Supplement1C_anti-Has1.tif]

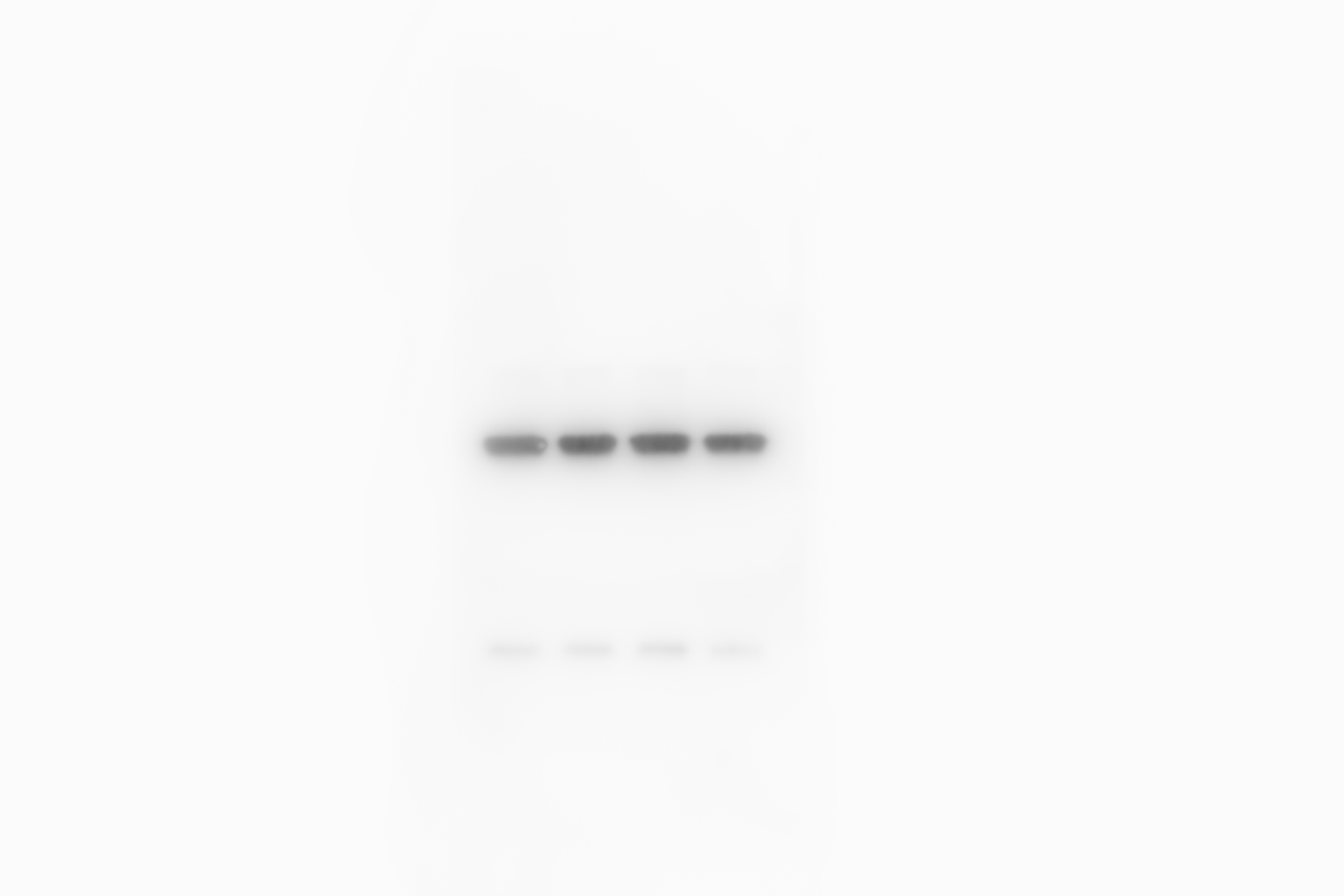

Supplement: Figure 4—figure supplement 1—source data 1. — Dashed boxes in the PDF indicate the respective areas shown in the figure. [file elife-84877-fig4-figsupp1-data1.zip › Figure4_Figure_Supplement1_Source_data1/Figure4_Figure_Supplement1C_anti-L3.tif]

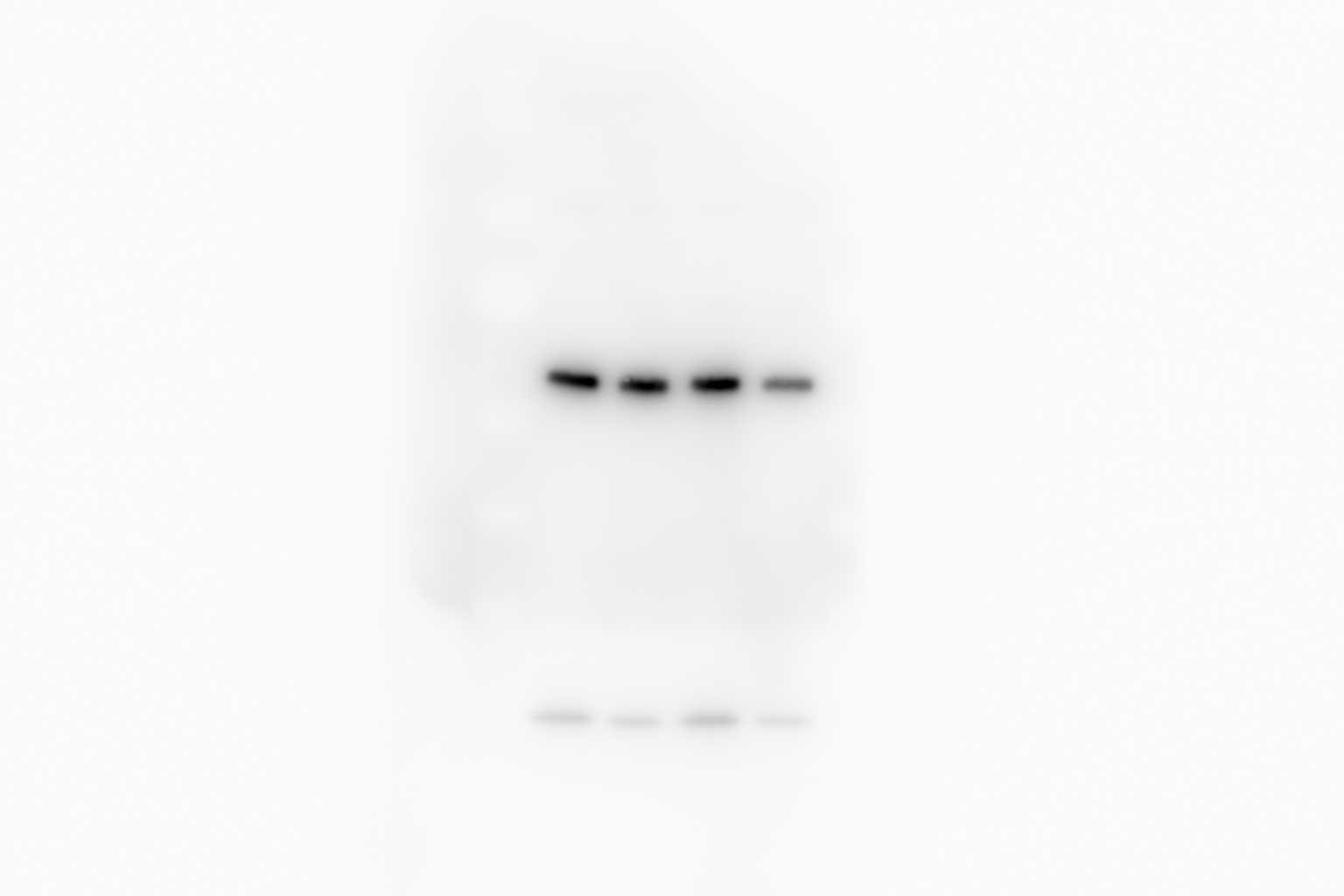

Supplement: Figure 4—figure supplement 1—source data 1. — Dashed boxes in the PDF indicate the respective areas shown in the figure. [file elife-84877-fig4-figsupp1-data1.zip › Figure4_Figure_Supplement1_Source_data1/Figure4_Figure_Supplement1C_anti-Ytm1.tif]

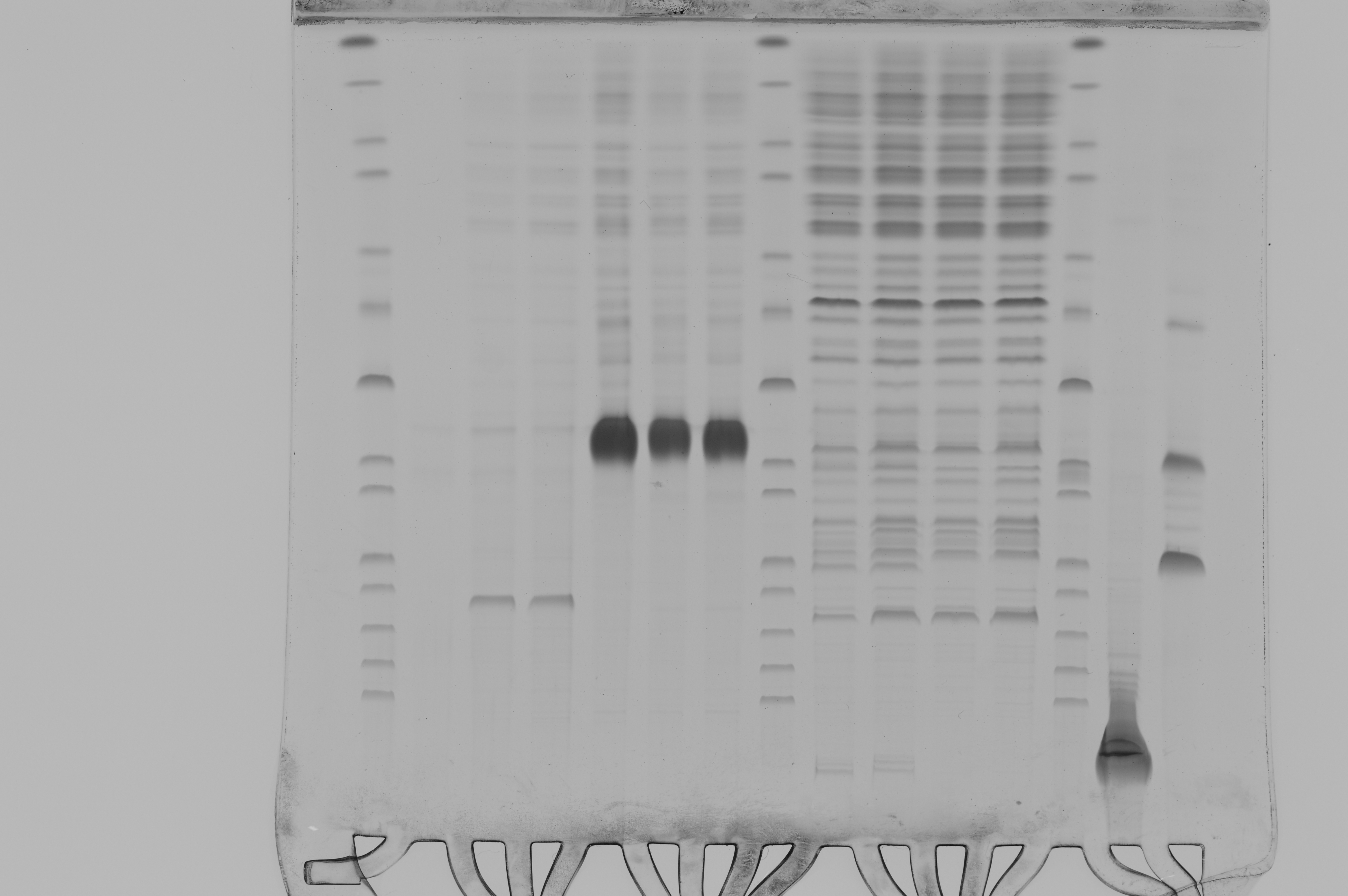

Supplement: Figure 4—figure supplement 1—source data 1. — Dashed boxes in the PDF indicate the respective areas shown in the figure. [file elife-84877-fig4-figsupp1-data1.zip › Figure4_Figure_Supplement1_Source_data1/Figure4_Figure_Supplement1C_Coomassie.JPG]

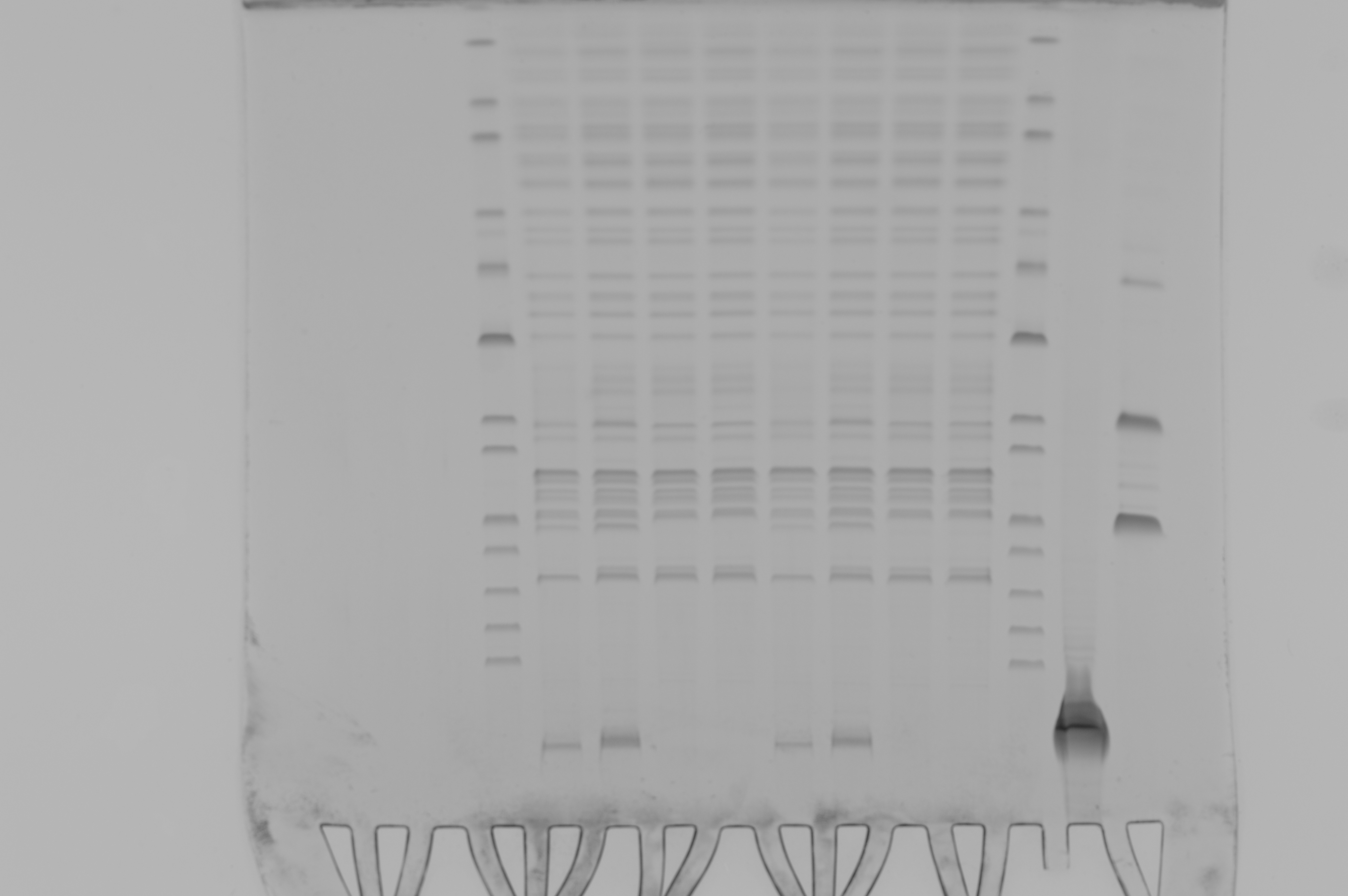

Supplement: Figure 4—figure supplement 2—source data 1. — Dashed boxes in the PDF indicate the respective areas shown in the figure. [file elife-84877-fig4-figsupp2-data1.zip › Figure4_Figure_Supplement2_Source_data1/Figure4_Figure_Supplement2B_left_panel_Coomassie.JPG]

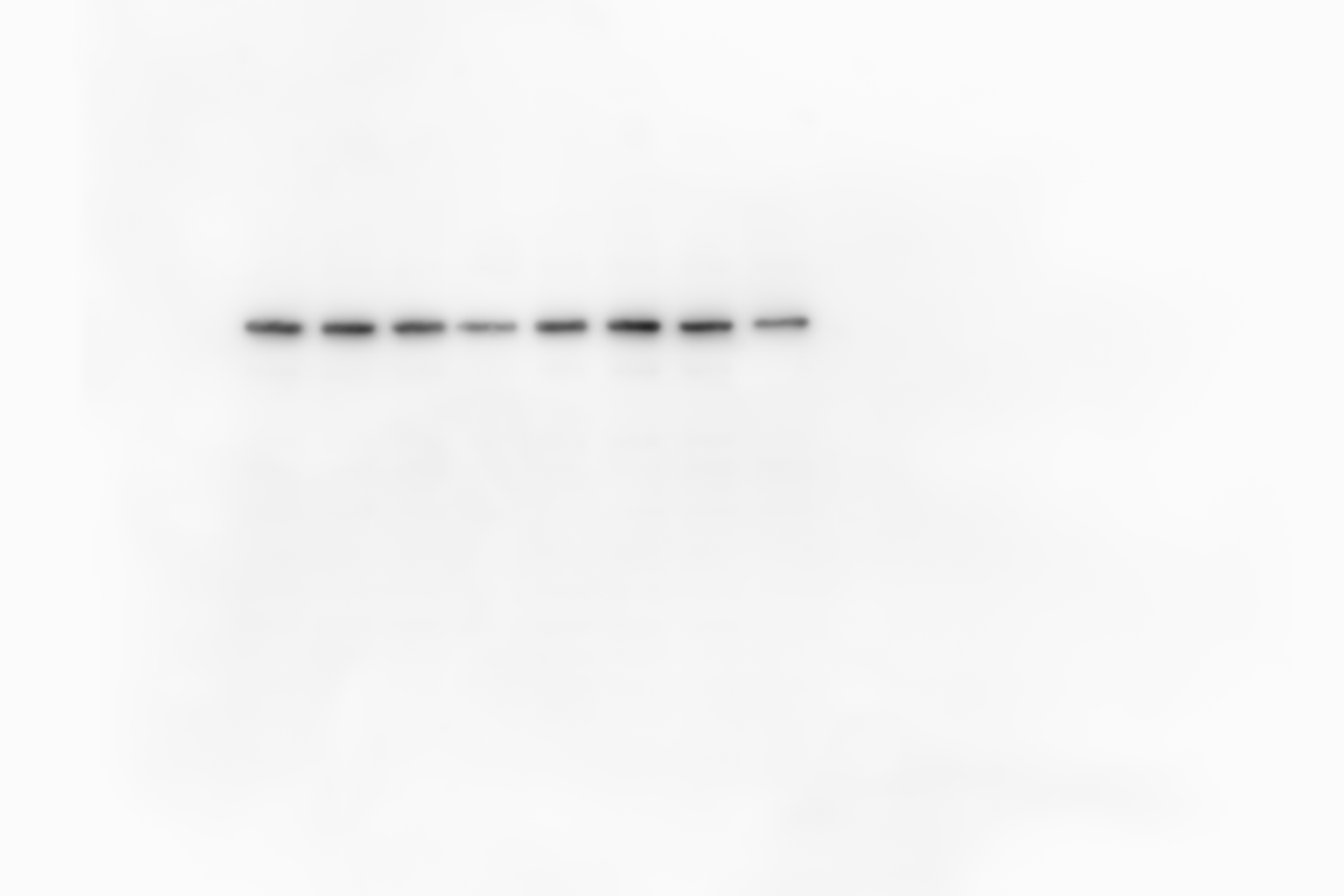

Supplement: Figure 4—figure supplement 2—source data 1. — Dashed boxes in the PDF indicate the respective areas shown in the figure. [file elife-84877-fig4-figsupp2-data1.zip › Figure4_Figure_Supplement2_Source_data1/Figure4_Figure_Supplement2B_left_panel_Ebp2.tif]

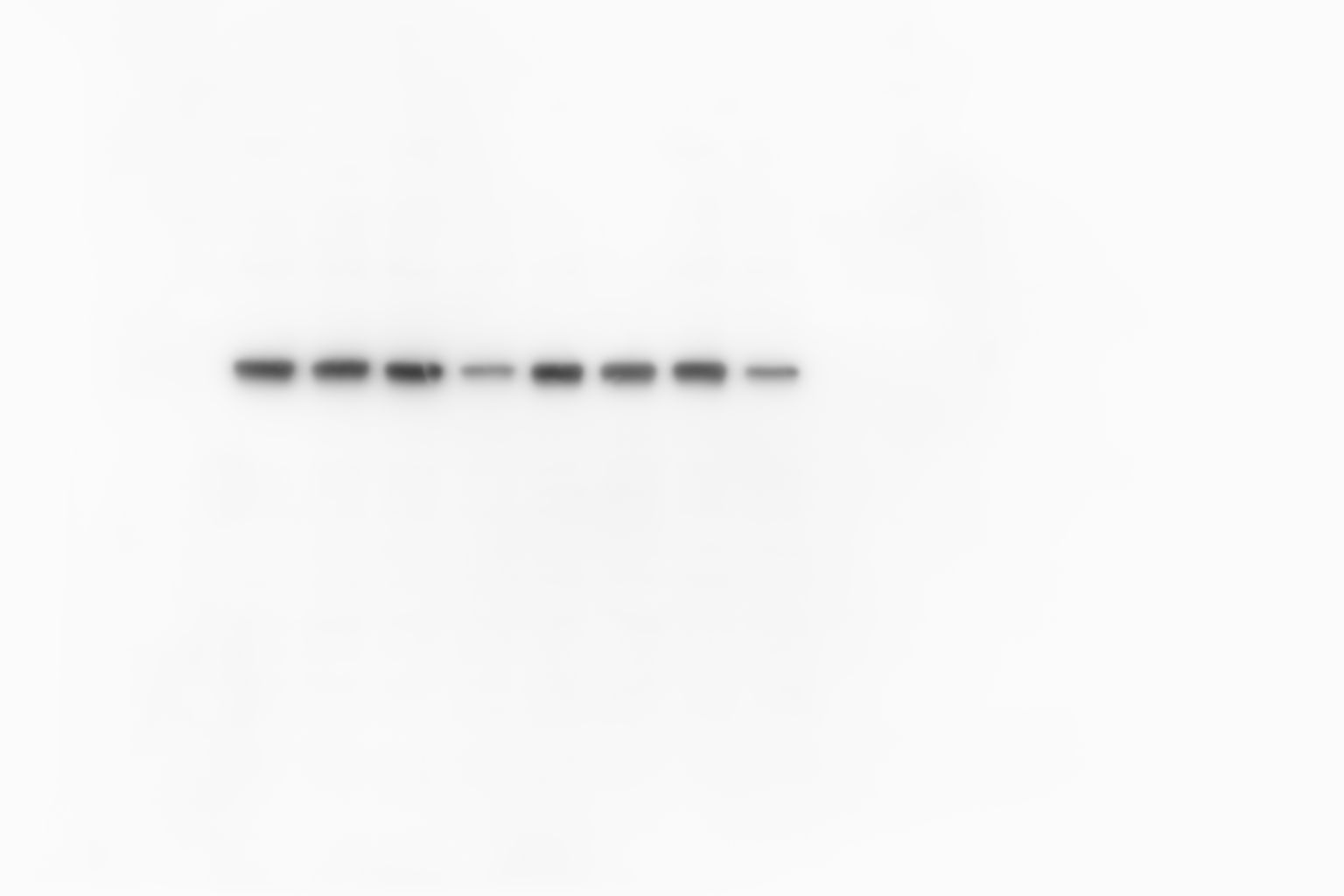

Supplement: Figure 4—figure supplement 2—source data 1. — Dashed boxes in the PDF indicate the respective areas shown in the figure. [file elife-84877-fig4-figsupp2-data1.zip › Figure4_Figure_Supplement2_Source_data1/Figure4_Figure_Supplement2B_left_panel_Has1.tif]

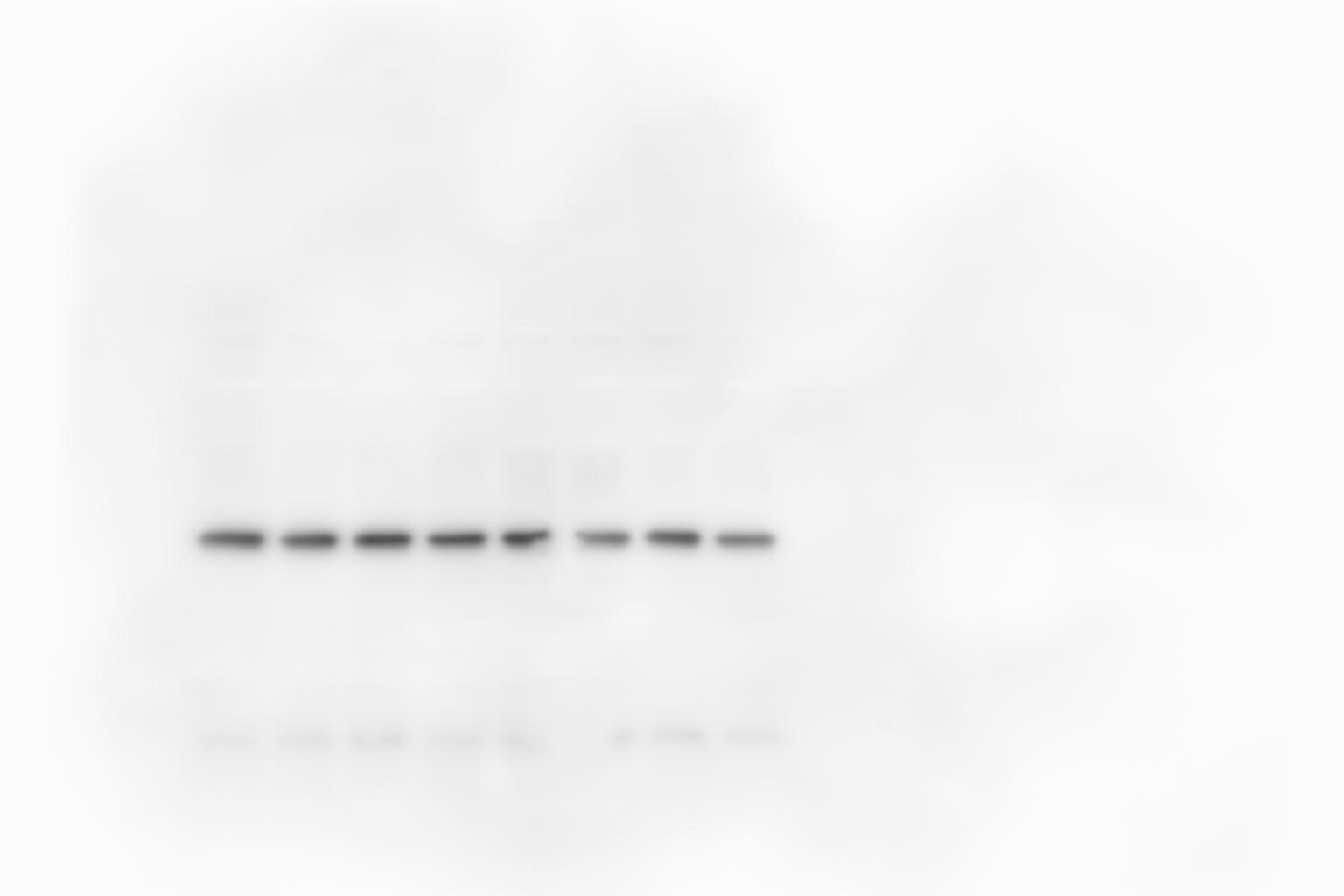

Supplement: Figure 4—figure supplement 2—source data 1. — Dashed boxes in the PDF indicate the respective areas shown in the figure. [file elife-84877-fig4-figsupp2-data1.zip › Figure4_Figure_Supplement2_Source_data1/Figure4_Figure_Supplement2B_left_panel_L3.tif]

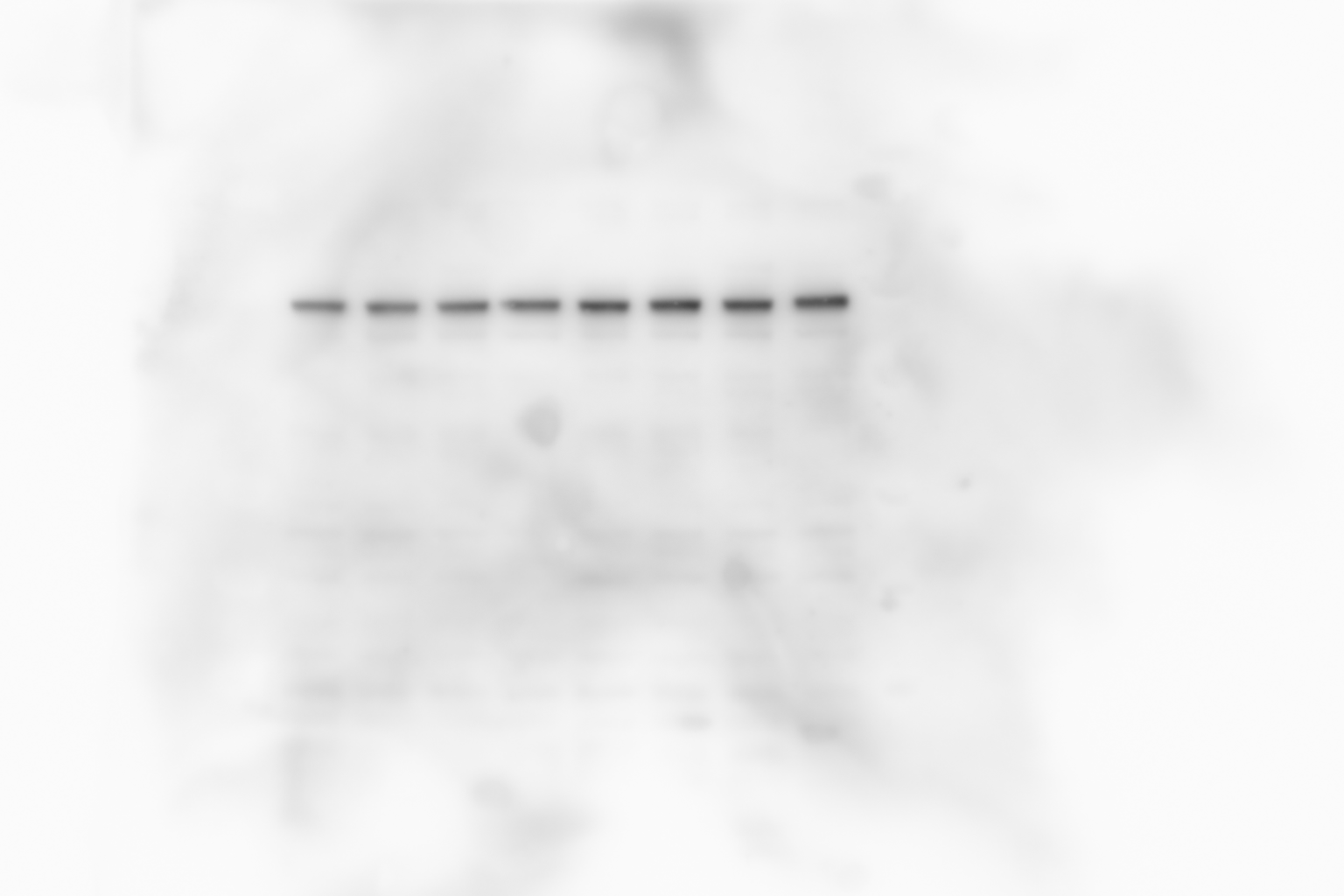

Supplement: Figure 4—figure supplement 2—source data 1. — Dashed boxes in the PDF indicate the respective areas shown in the figure. [file elife-84877-fig4-figsupp2-data1.zip › Figure4_Figure_Supplement2_Source_data1/Figure4_Figure_Supplement2B_left_panel_Noc3.tif]

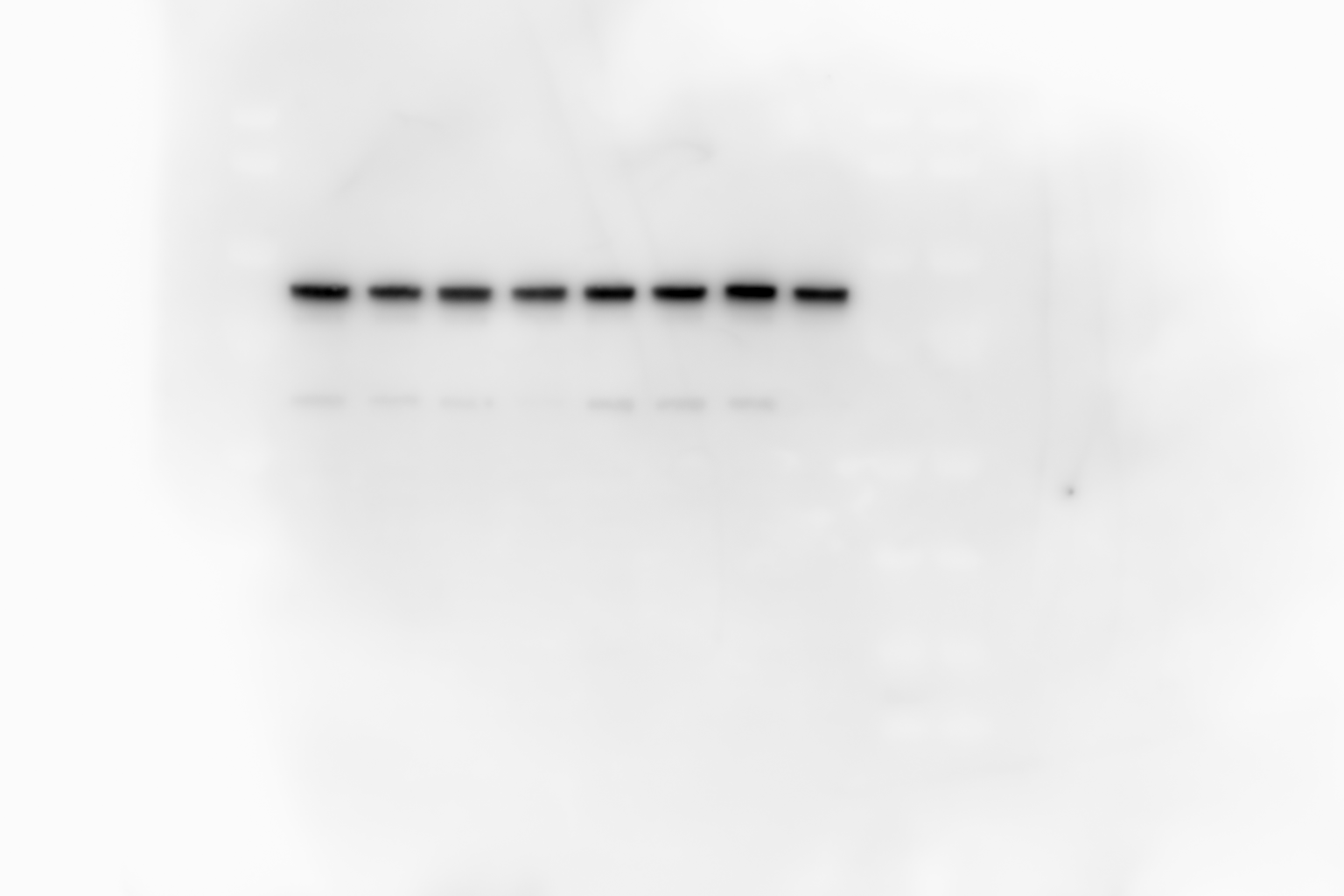

Supplement: Figure 4—figure supplement 2—source data 1. — Dashed boxes in the PDF indicate the respective areas shown in the figure. [file elife-84877-fig4-figsupp2-data1.zip › Figure4_Figure_Supplement2_Source_data1/Figure4_Figure_Supplement2B_left_panel_Nog1.tif]

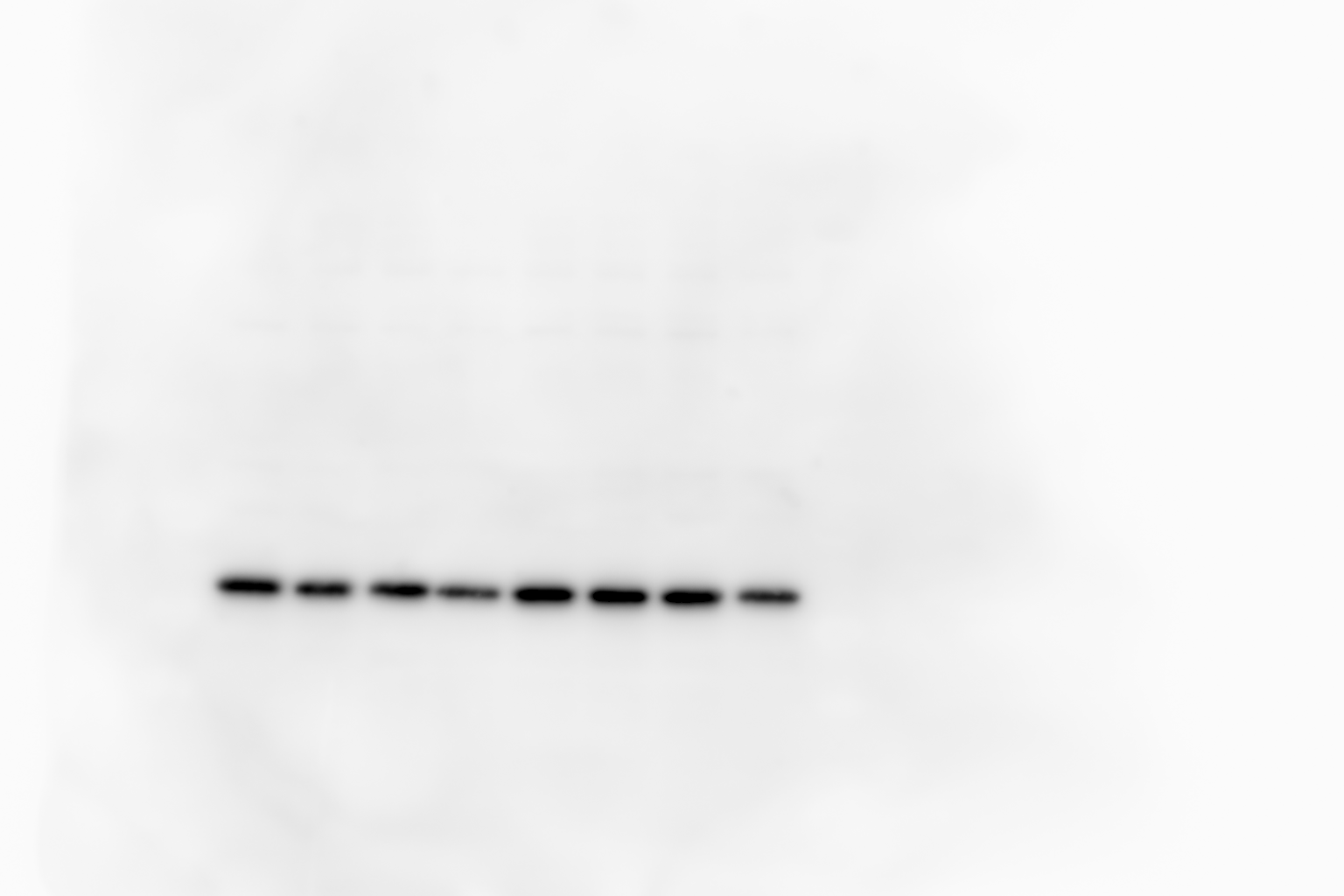

Supplement: Figure 4—figure supplement 2—source data 1. — Dashed boxes in the PDF indicate the respective areas shown in the figure. [file elife-84877-fig4-figsupp2-data1.zip › Figure4_Figure_Supplement2_Source_data1/Figure4_Figure_Supplement2B_left_panel_Nsa2.tif]

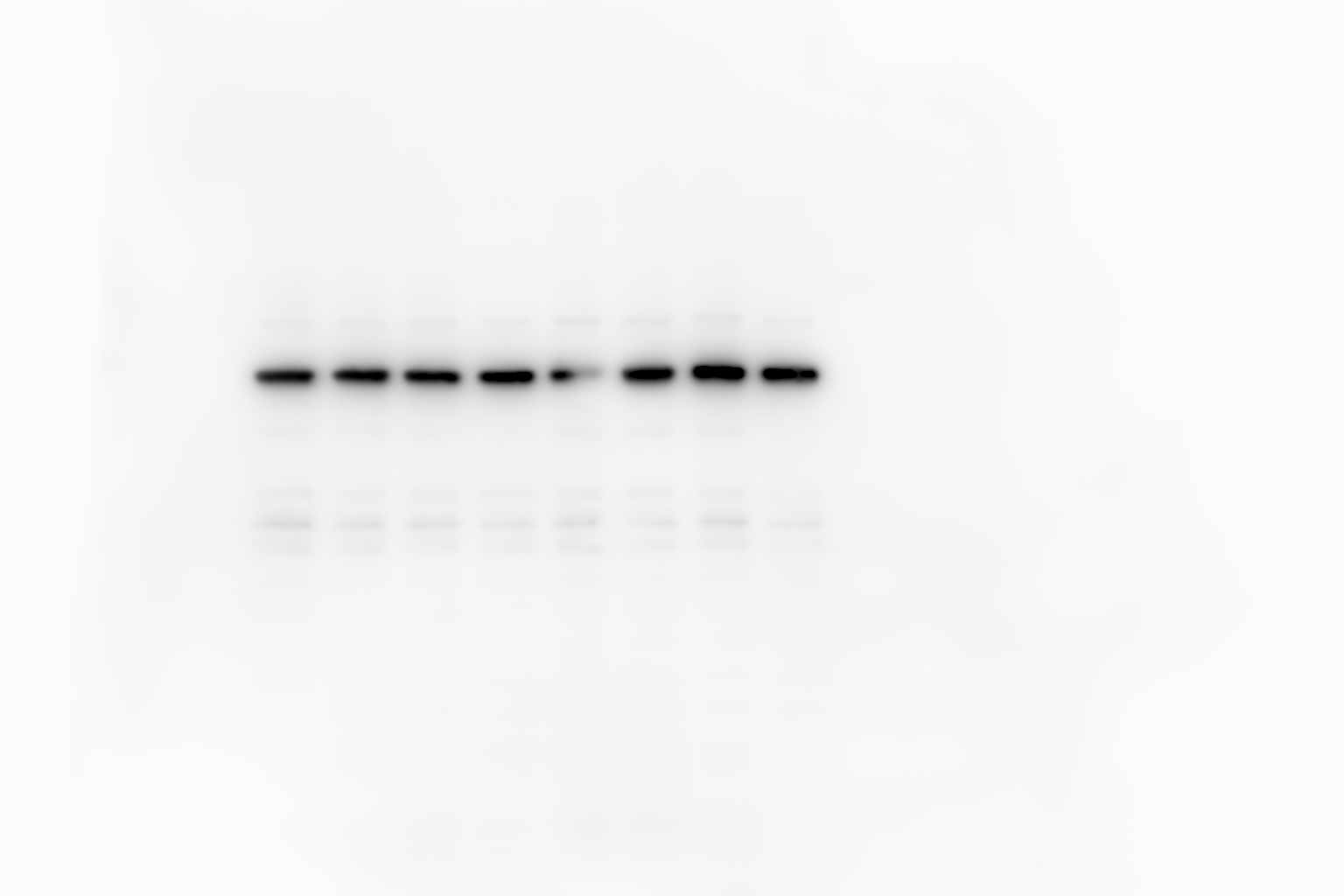

Supplement: Figure 4—figure supplement 2—source data 1. — Dashed boxes in the PDF indicate the respective areas shown in the figure. [file elife-84877-fig4-figsupp2-data1.zip › Figure4_Figure_Supplement2_Source_data1/Figure4_Figure_Supplement2B_left_panel_Nug1.tif]

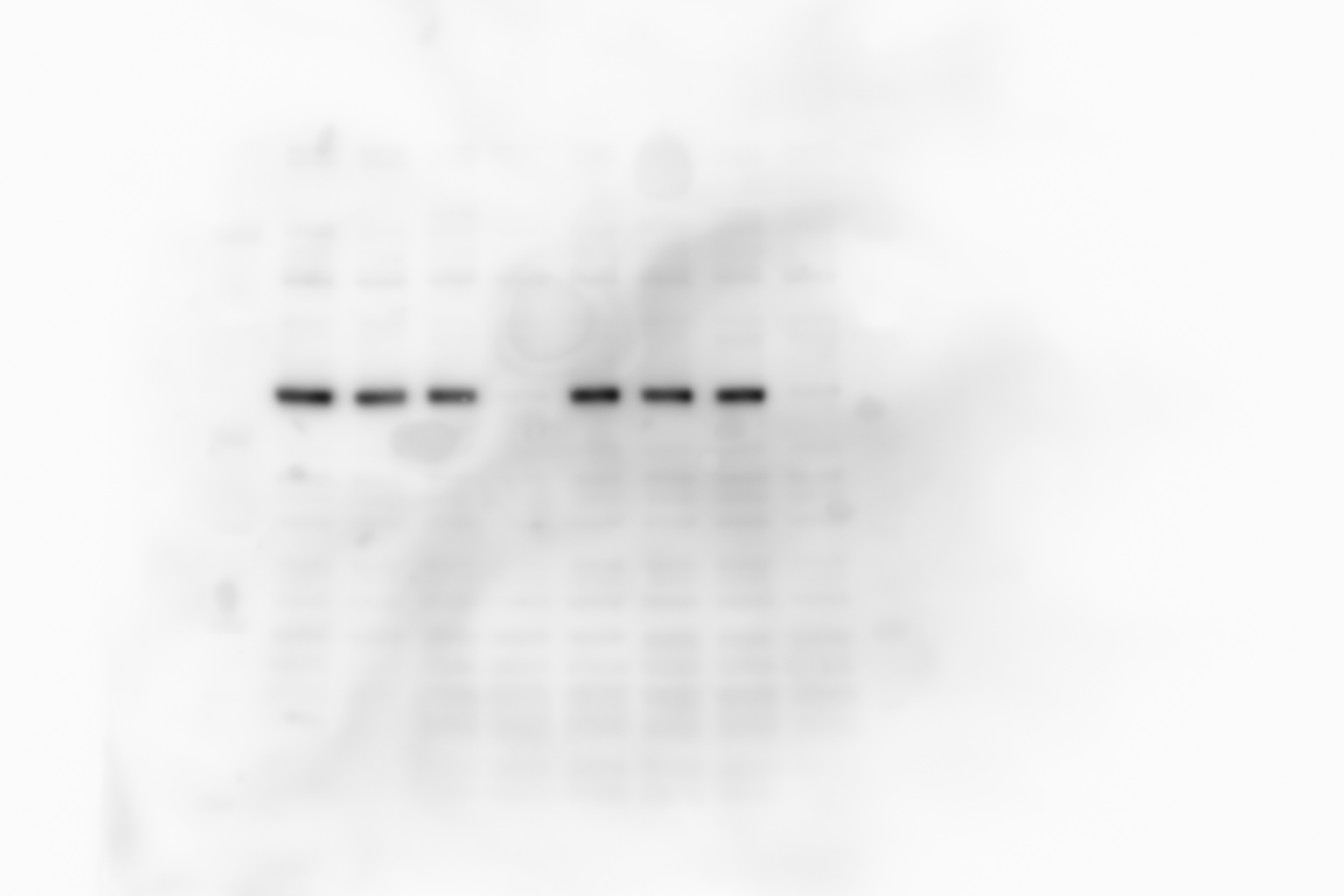

Supplement: Figure 4—figure supplement 2—source data 1. — Dashed boxes in the PDF indicate the respective areas shown in the figure. [file elife-84877-fig4-figsupp2-data1.zip › Figure4_Figure_Supplement2_Source_data1/Figure4_Figure_Supplement2B_left_panel_Ytm1.tif]

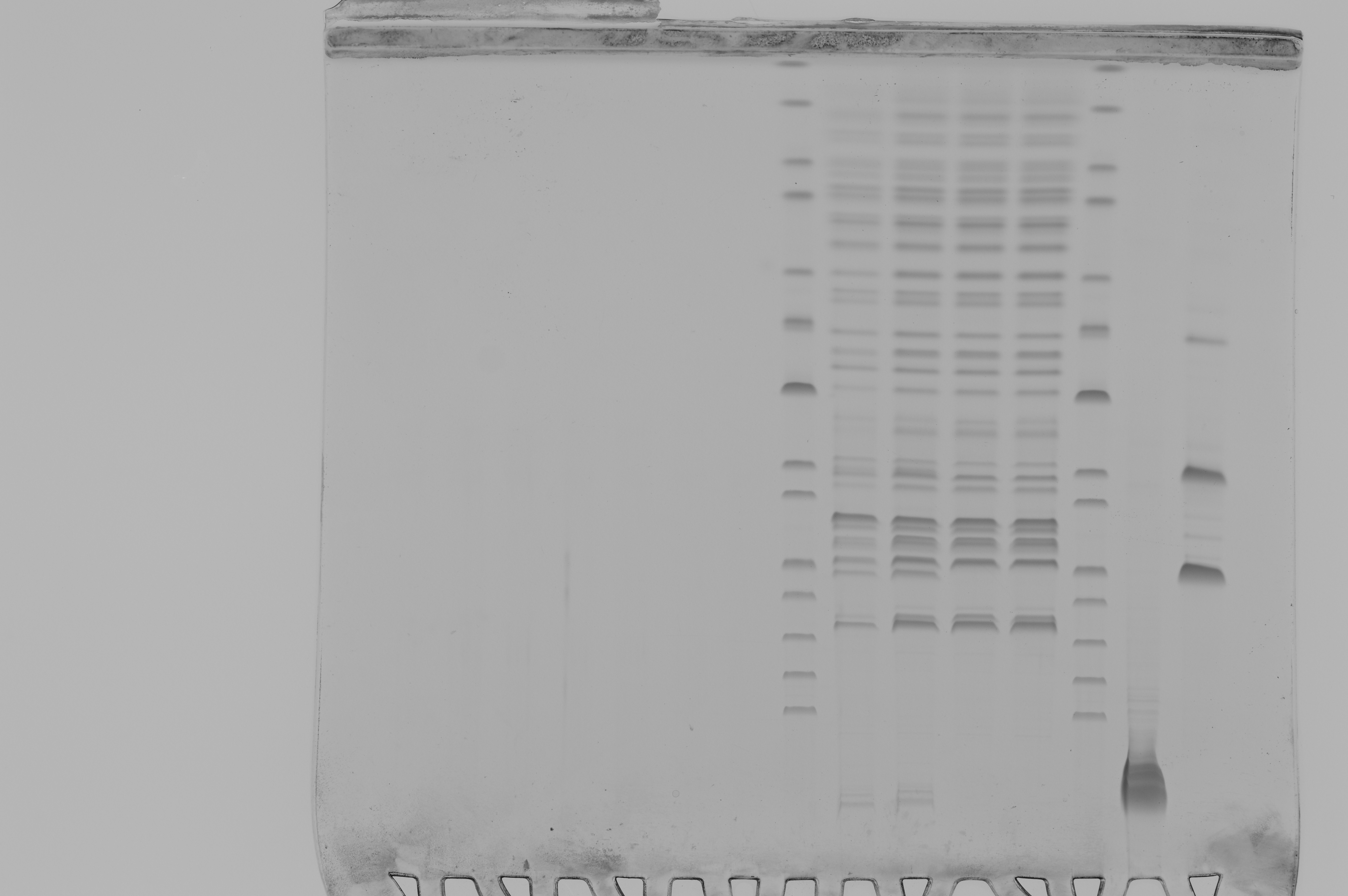

Supplement: Figure 4—figure supplement 2—source data 1. — Dashed boxes in the PDF indicate the respective areas shown in the figure. [file elife-84877-fig4-figsupp2-data1.zip › Figure4_Figure_Supplement2_Source_data1/Figure4_Figure_Supplement2B_right_panel_Coomassie.JPG]

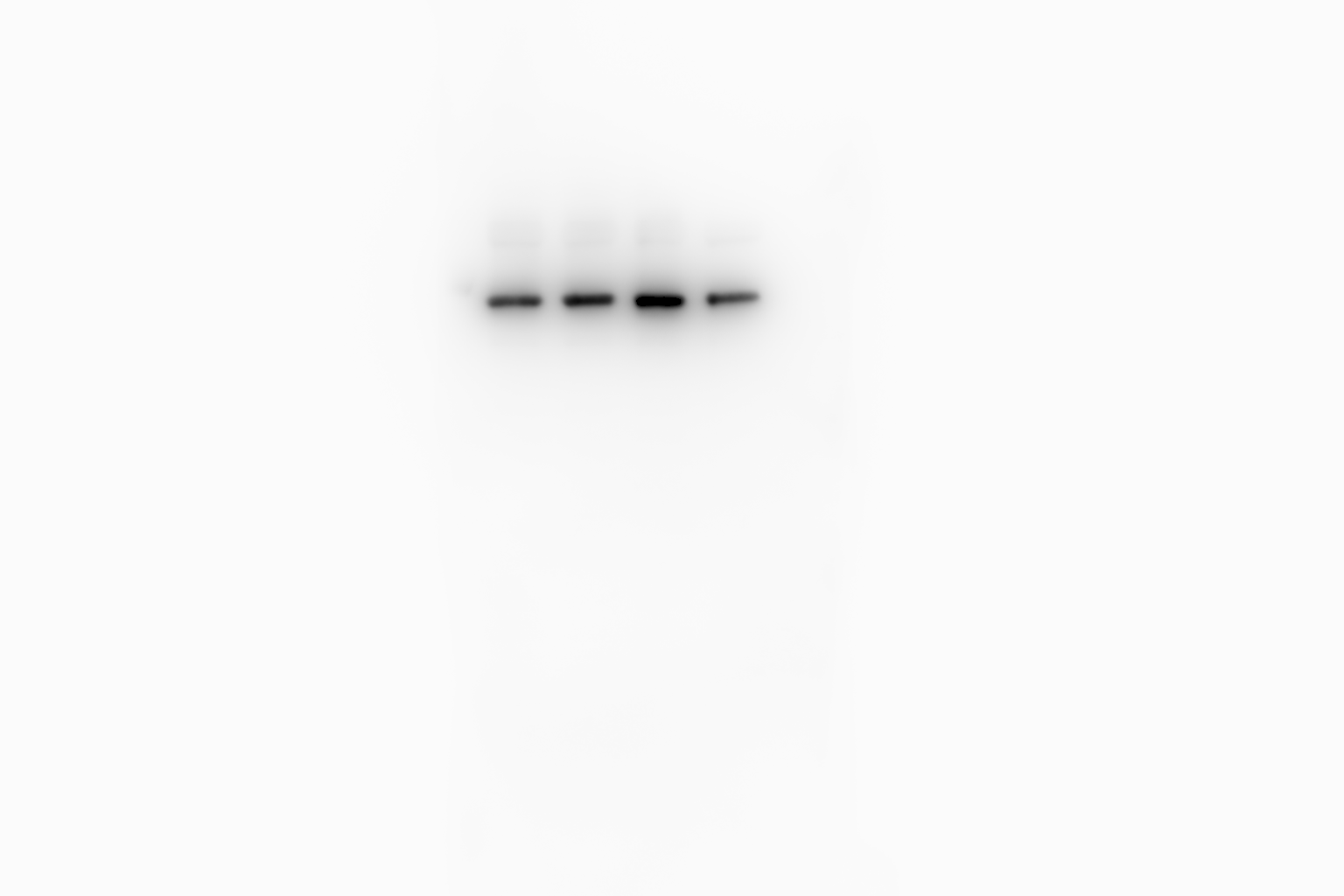

Supplement: Figure 4—figure supplement 2—source data 1. — Dashed boxes in the PDF indicate the respective areas shown in the figure. [file elife-84877-fig4-figsupp2-data1.zip › Figure4_Figure_Supplement2_Source_data1/Figure4_Figure_Supplement2B_right_panel_Ebp2.tif]

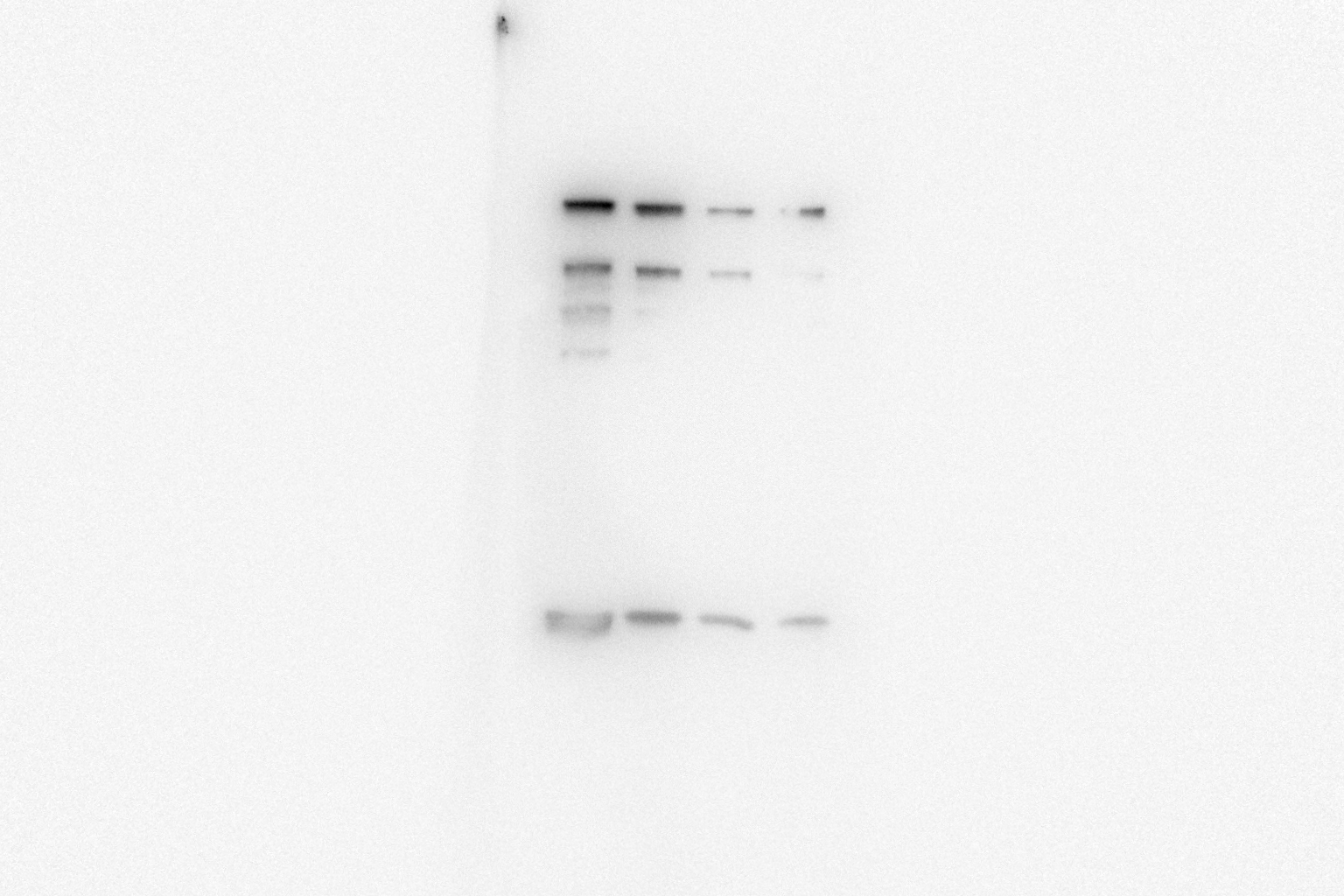

Supplement: Figure 4—figure supplement 2—source data 1. — Dashed boxes in the PDF indicate the respective areas shown in the figure. [file elife-84877-fig4-figsupp2-data1.zip › Figure4_Figure_Supplement2_Source_data1/Figure4_Figure_Supplement2B_right_panel_Has1.tif]

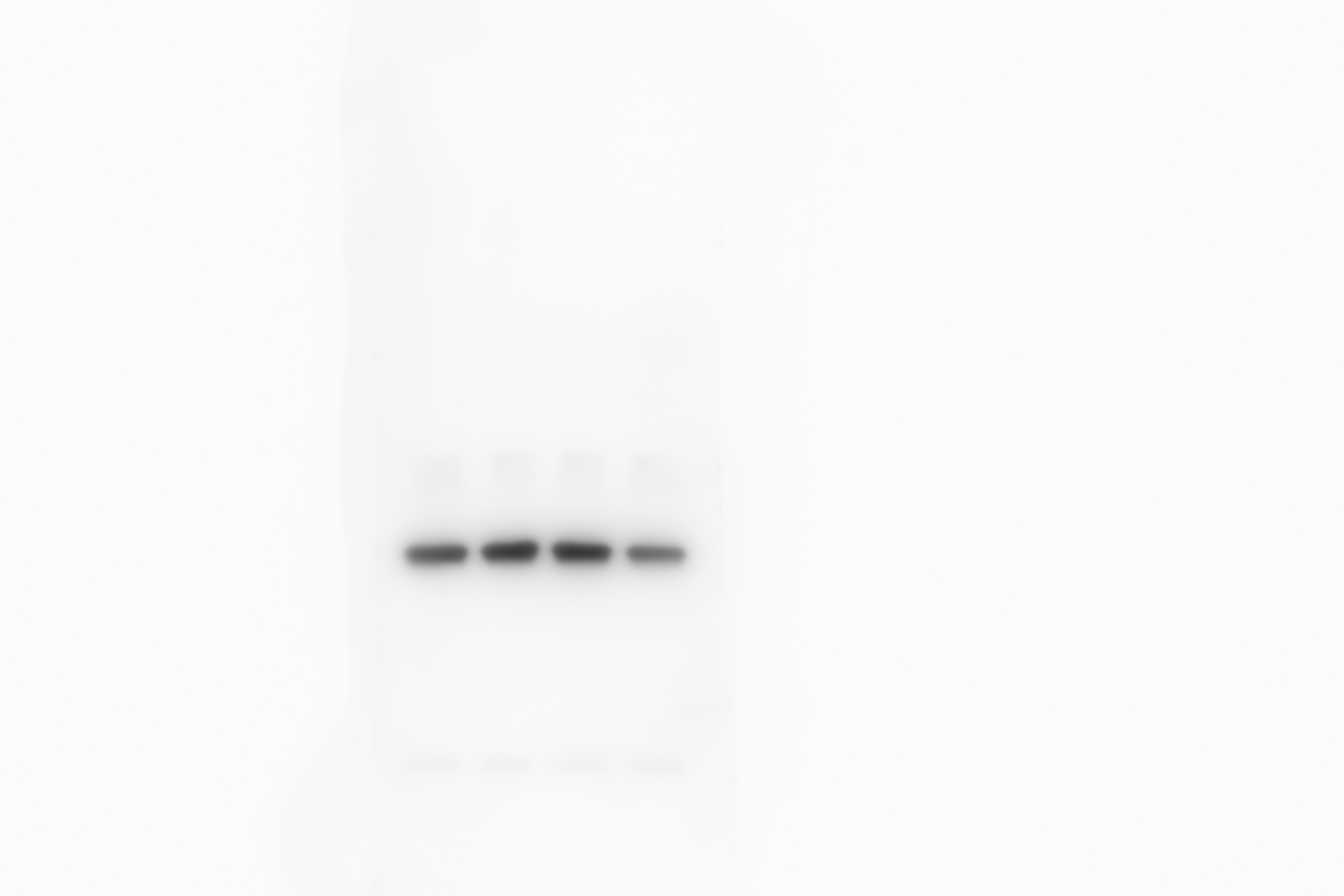

Supplement: Figure 4—figure supplement 2—source data 1. — Dashed boxes in the PDF indicate the respective areas shown in the figure. [file elife-84877-fig4-figsupp2-data1.zip › Figure4_Figure_Supplement2_Source_data1/Figure4_Figure_Supplement2B_right_panel_L3.tif]

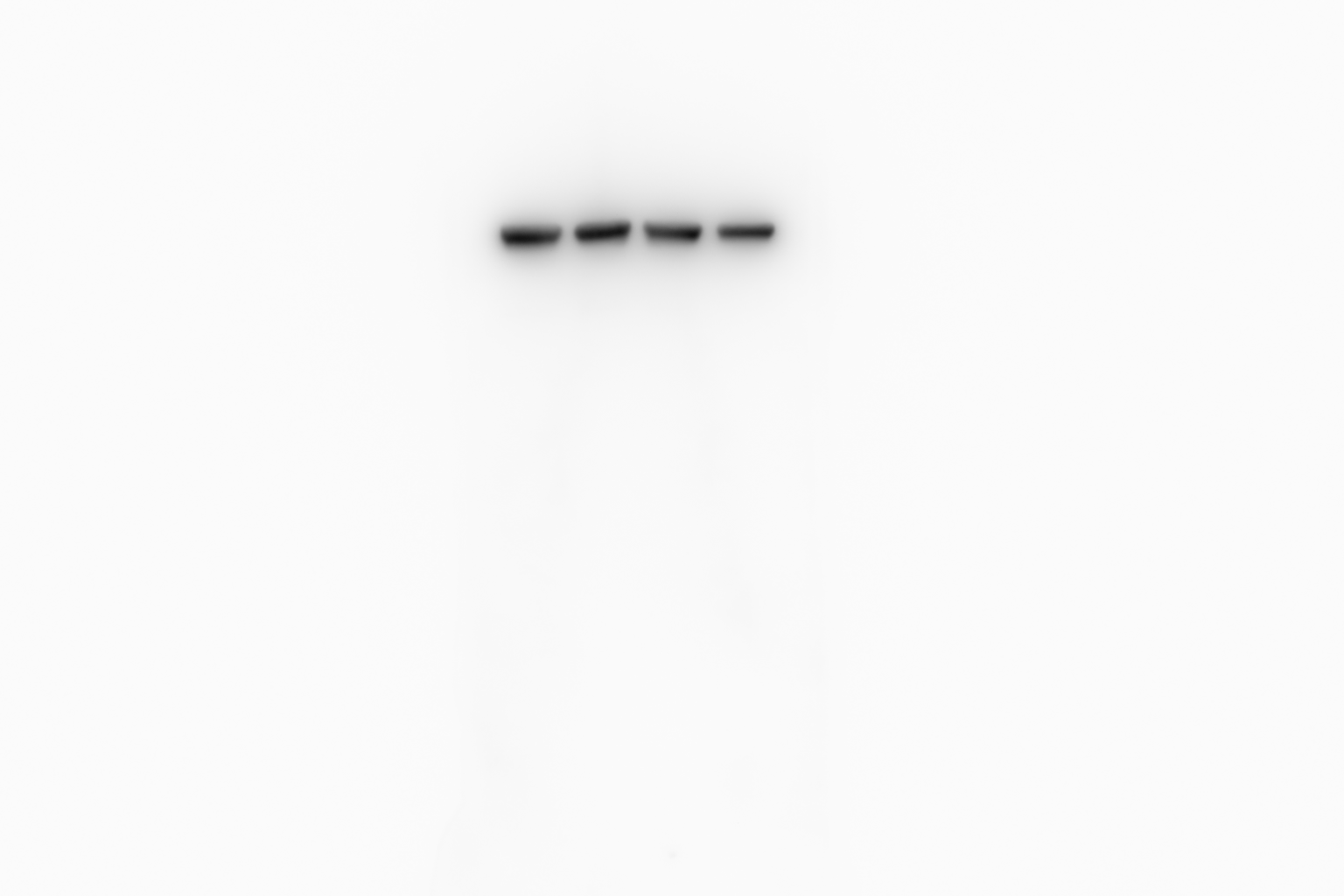

Supplement: Figure 4—figure supplement 2—source data 1. — Dashed boxes in the PDF indicate the respective areas shown in the figure. [file elife-84877-fig4-figsupp2-data1.zip › Figure4_Figure_Supplement2_Source_data1/Figure4_Figure_Supplement2B_right_panel_Noc3.tif]

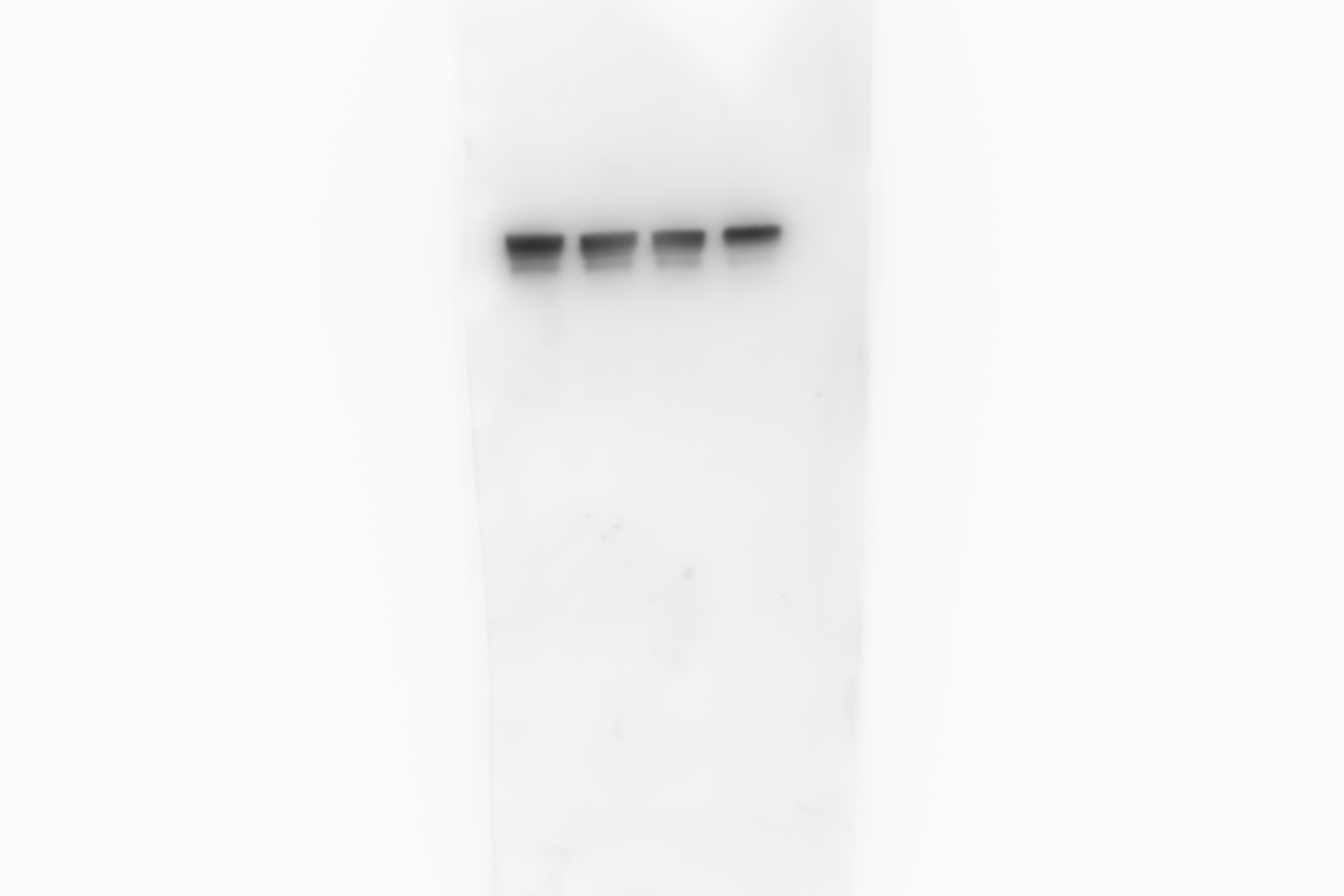

Supplement: Figure 4—figure supplement 2—source data 1. — Dashed boxes in the PDF indicate the respective areas shown in the figure. [file elife-84877-fig4-figsupp2-data1.zip › Figure4_Figure_Supplement2_Source_data1/Figure4_Figure_Supplement2B_right_panel_Nog1.tif]

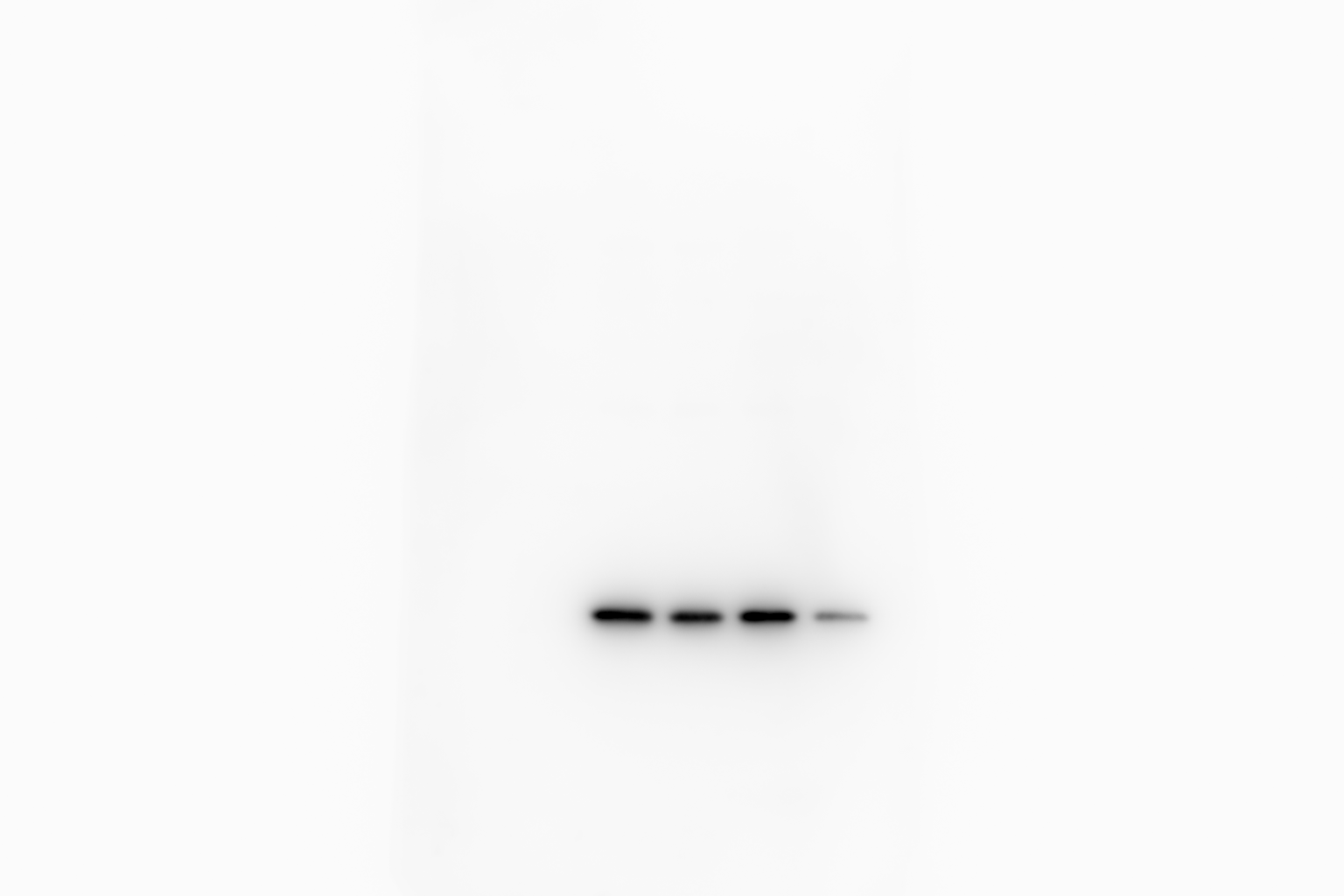

Supplement: Figure 4—figure supplement 2—source data 1. — Dashed boxes in the PDF indicate the respective areas shown in the figure. [file elife-84877-fig4-figsupp2-data1.zip › Figure4_Figure_Supplement2_Source_data1/Figure4_Figure_Supplement2B_right_panel_Nsa2.tif]

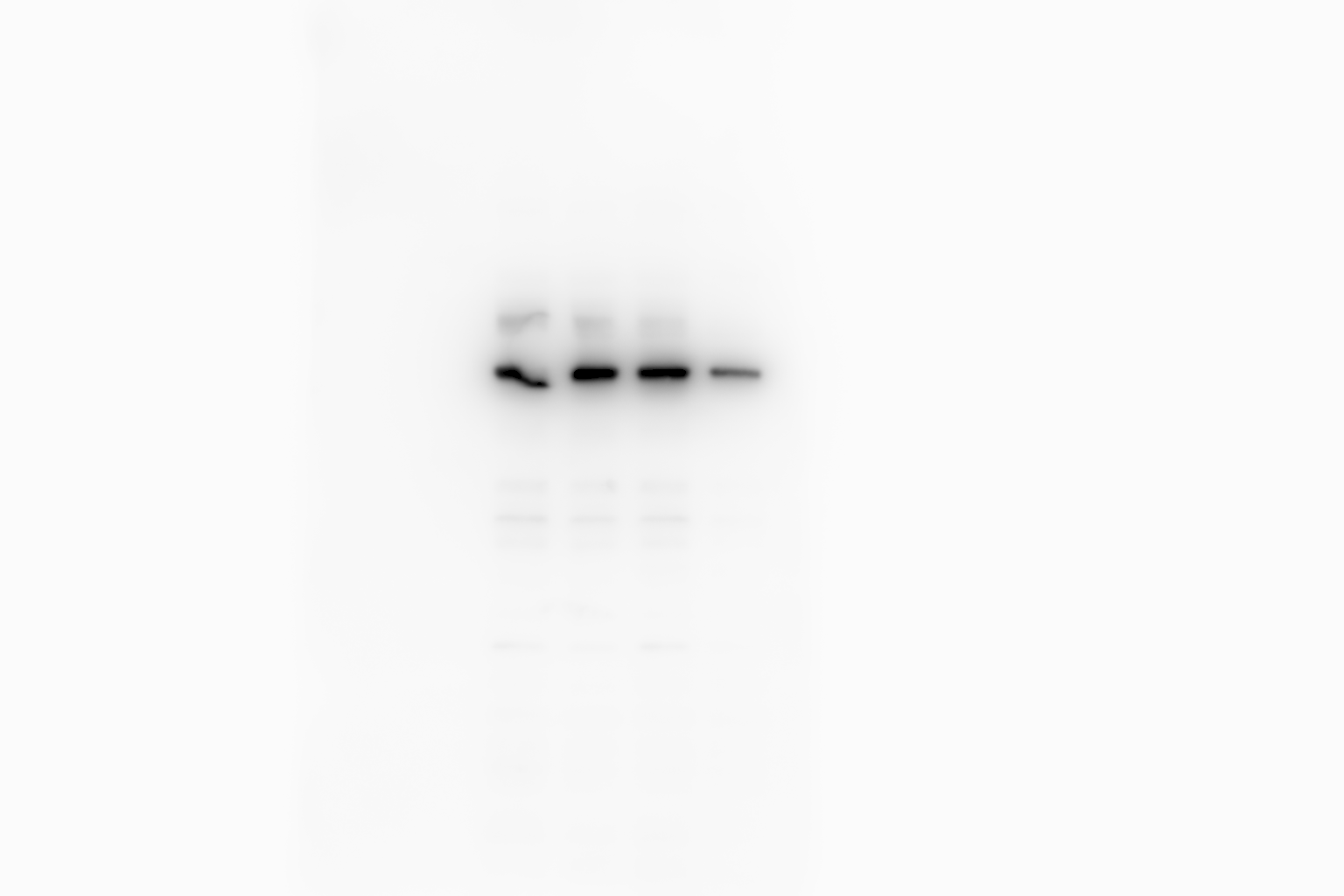

Supplement: Figure 4—figure supplement 2—source data 1. — Dashed boxes in the PDF indicate the respective areas shown in the figure. [file elife-84877-fig4-figsupp2-data1.zip › Figure4_Figure_Supplement2_Source_data1/Figure4_Figure_Supplement2B_right_panel_Nug1.tif]

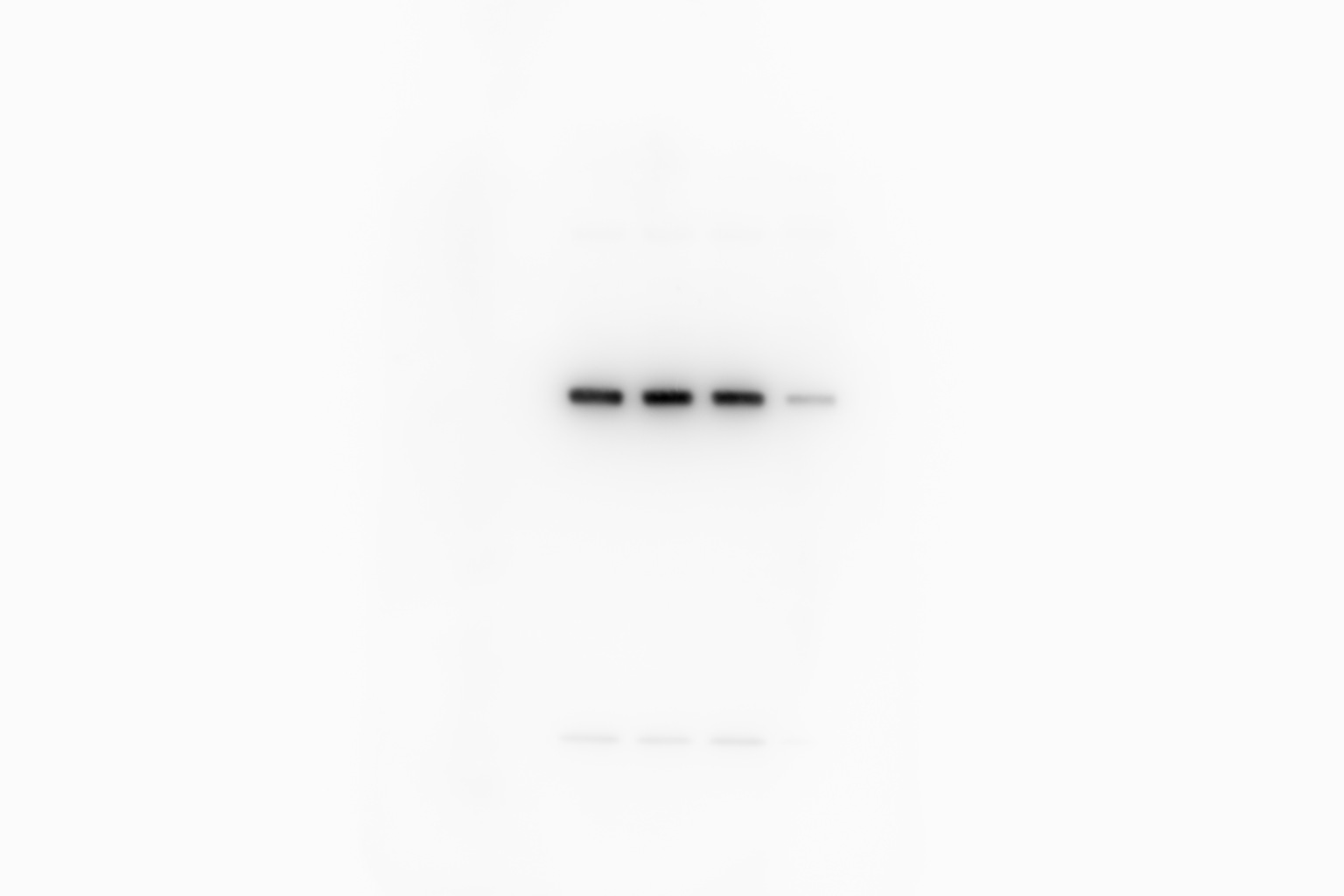

Supplement: Figure 4—figure supplement 2—source data 1. — Dashed boxes in the PDF indicate the respective areas shown in the figure. [file elife-84877-fig4-figsupp2-data1.zip › Figure4_Figure_Supplement2_Source_data1/Figure4_Figure_Supplement2B_right_panel_Ytm1.tif]
