# Supplementary material for: Concurrent remodelling of nucleolar 60S subunit precursors by the Rea1 ATPase and Spb4 RNA helicase
Source: eLife. 2023 Mar 17;12:e84877. doi: 10.7554/eLife.84877 (PMC10154028; doi:10.7554/eLife.84877)

Figure 1–Figure Supplement 1A, upper panel, anti-HA

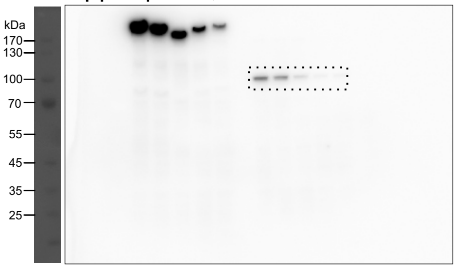

Figure 1–Figure Supplement 1A, upper panel, anti-Arc1

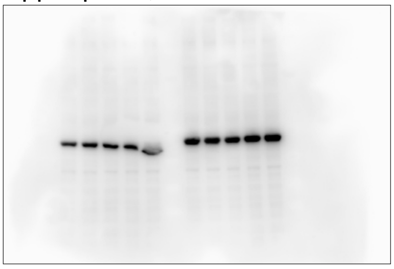

Figure 1–Figure Supplement 1A, lower panel, anti-HA

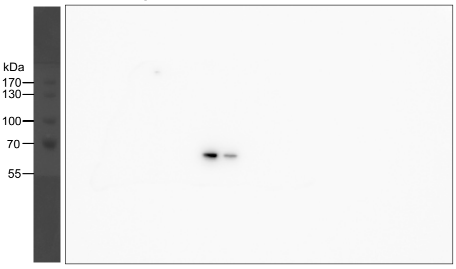

Figure 1–Figure Supplement 1A, lower panel, anti-Arc1

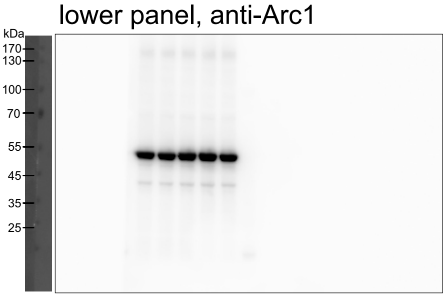

Figure 1–Figure Supplement 1C, left panel

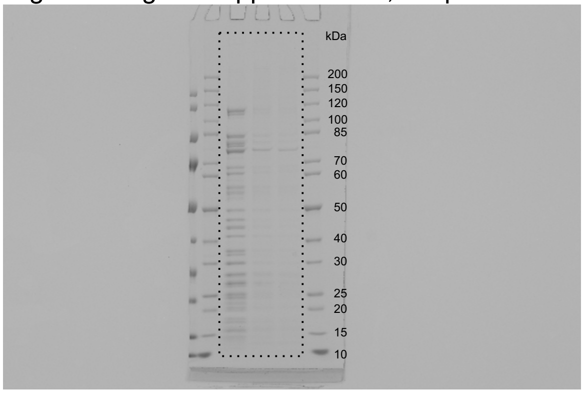

Figure 1–Figure Supplement 1C, right panel

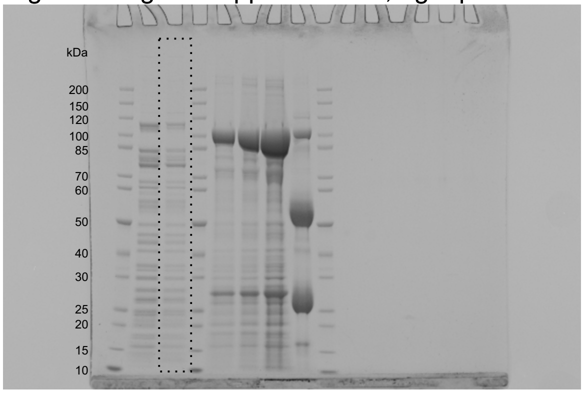

Figure 1–Figure Supplement 1D

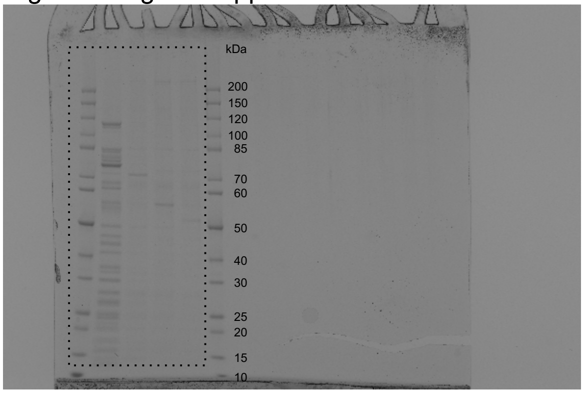

Supplement: Figure 1—figure supplement 1—source data 1. — Dashed boxes in the PDF indicate the respective areas shown in the figure. [file elife-84877-fig1-figsupp1-data1.zip › Figure1_Figure_Supplement1_Sourca_data1/Figure1_Figure_Supplement1_Source_data.pdf]
