# Supplementary material for: Concurrent remodelling of nucleolar 60S subunit precursors by the Rea1 ATPase and Spb4 RNA helicase
Source: eLife. 2023 Mar 17;12:e84877. doi: 10.7554/eLife.84877 (PMC10154028; doi:10.7554/eLife.84877)

Figure 2C, left panel

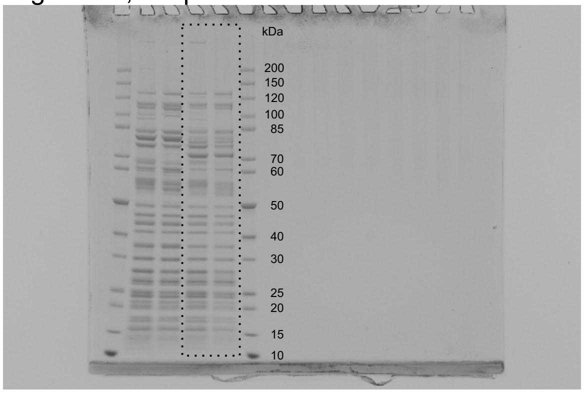

Figure 2C left panel, anti-Flag

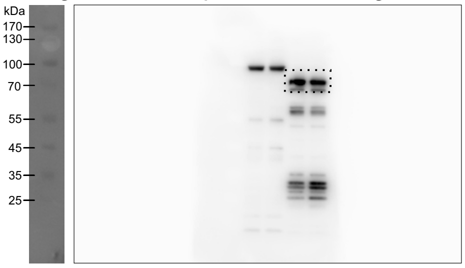

Figure 2C, left panel, anti-HA

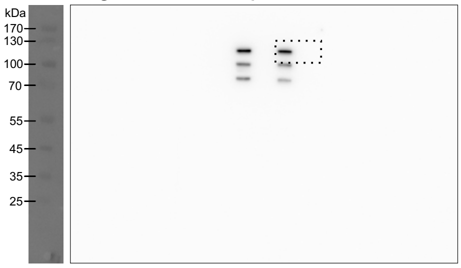

Figure 2C, left panel, anti-Has1

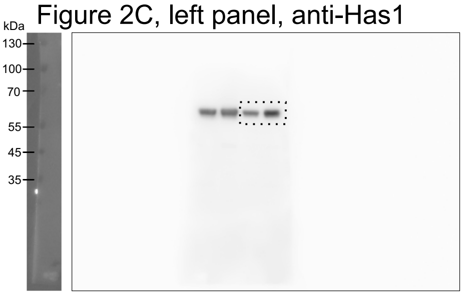

Figure 2C, left panel, anti-Ebp2

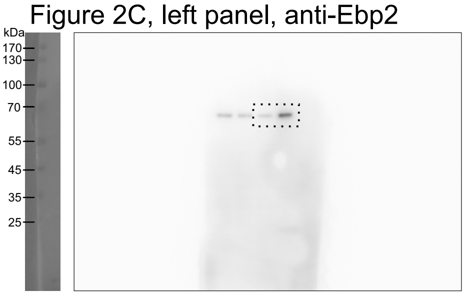

Figure 2C, left panel, anti-Rlp24

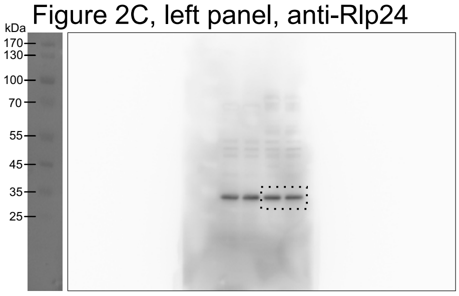

Figure 2C, left panel, anti-Nsa2

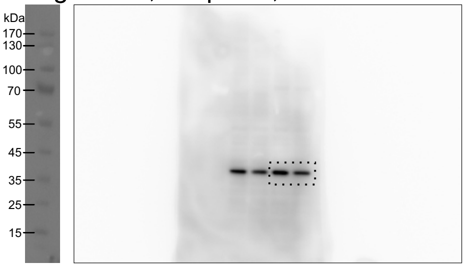

Figure 2C, left panel, anti-Nog2

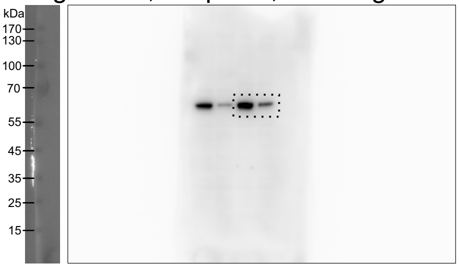

Figure 2C, left panel, anti-Bud20

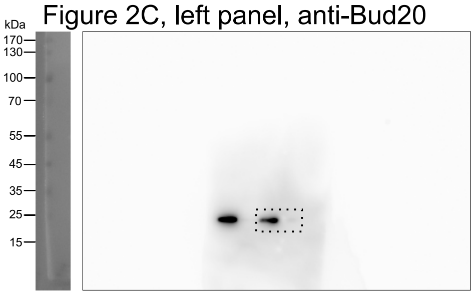

Figure 2C, left panel, anti-Arx1

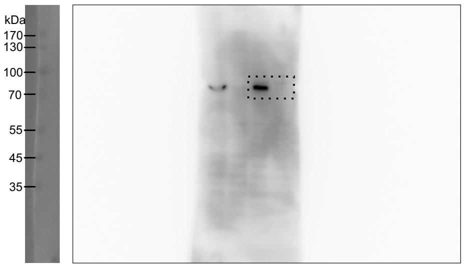

Figure 2C, left panel, anti-L3

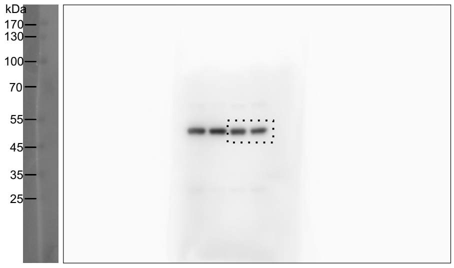

Figure 2C, right panel

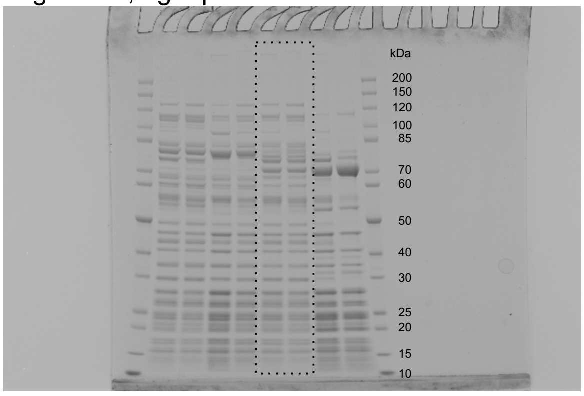

Figure 2C right panel, anti-Flag

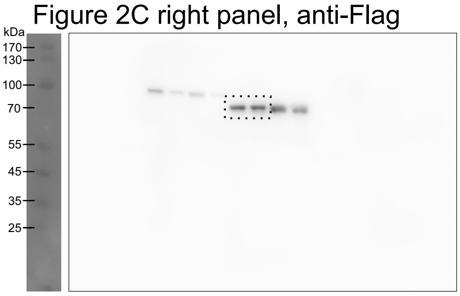

Figure 2C, right panel, anti-HA

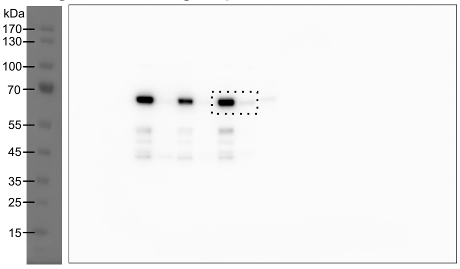

Figure 2C, right panel, anti-Has1

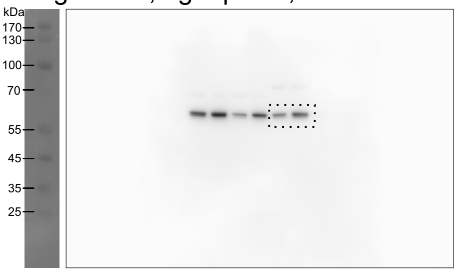

Figure 2C, right panel, anti-Ebp2

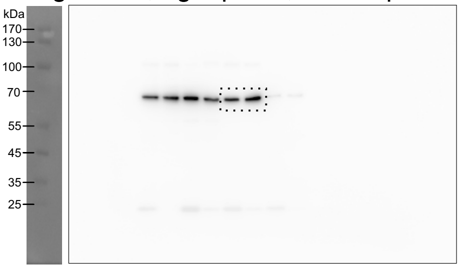

Figure 2C, right panel, anti-Rlp24

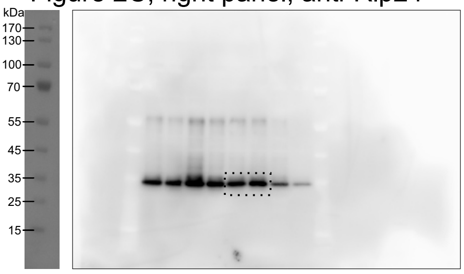

Figure 2C, right panel, anti-Nsa2

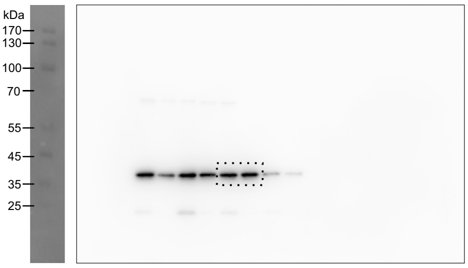

Figure 2C, right panel, anti-Nog2

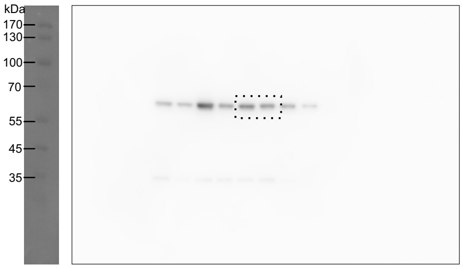

Figure 2C, right panel, anti-Bud20

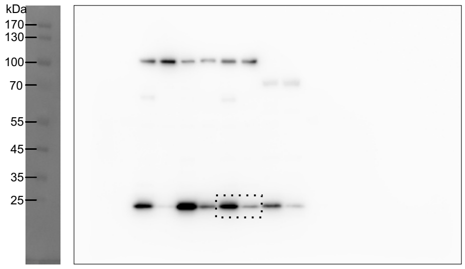

Figure 2C, right panel, anti-Arx1

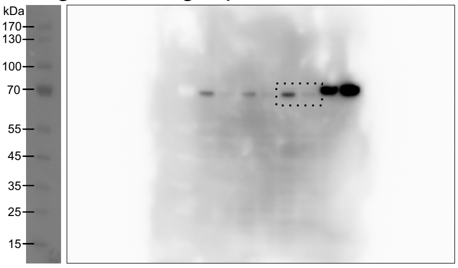

Figure 2C, right panel, anti-L3

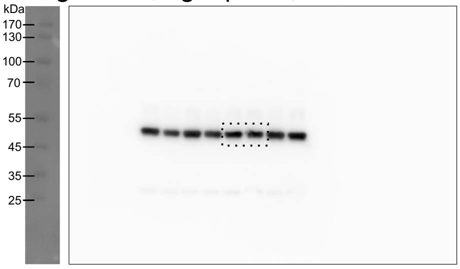

Supplement: Figure 2—source data 2. — Dashed boxes in the PDF indicate the respective areas shown in the figure. [file elife-84877-fig2-data2.zip › Figure2_Source_data_2/Figure2C_Source_data.pdf]
