# Supplementary material for: Concurrent remodelling of nucleolar 60S subunit precursors by the Rea1 ATPase and Spb4 RNA helicase
Source: eLife. 2023 Mar 17;12:e84877. doi: 10.7554/eLife.84877 (PMC10154028; doi:10.7554/eLife.84877)

Figure 4A

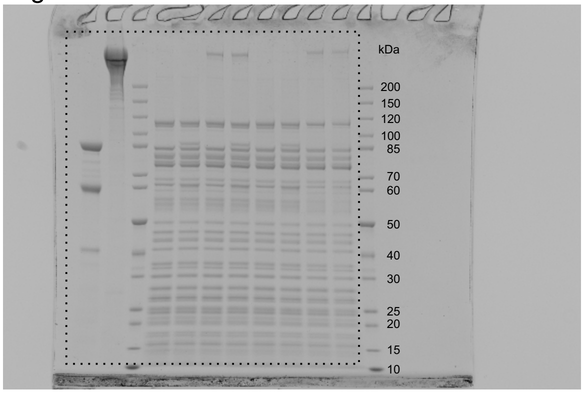

Figure 4A, anti-Ytm1

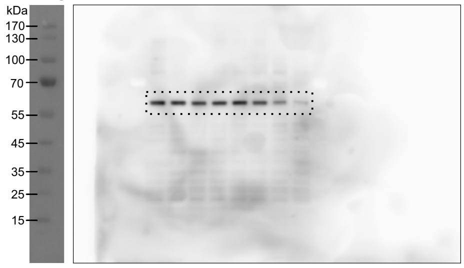

Figure 4A, anti-Has1

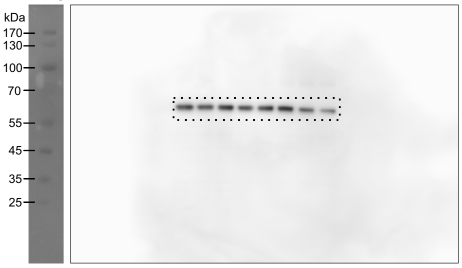

Figure 4A, anti-Noc3

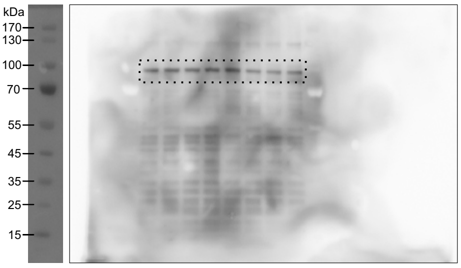

Figure 4A, anti-Nog1

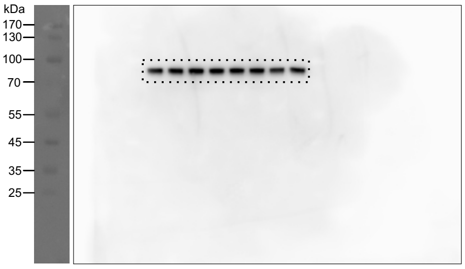

Figure 4A, anti-Nug1

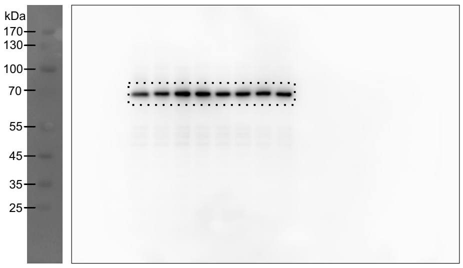

Figure 4A, anti-Nsa2

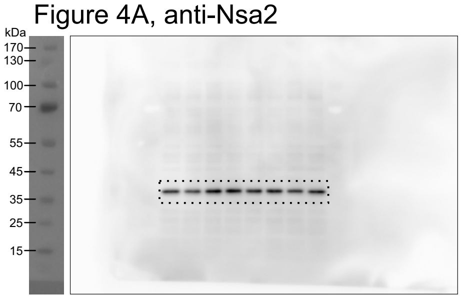

Figure 4A, anti-L3

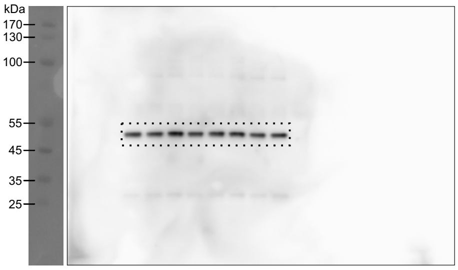

Supplement: Figure 4—source data 1. — Dashed boxes in the PDF indicate the respective areas shown in the figure. [file elife-84877-fig4-data1.zip › Figure4_Source_data/Figure4_Source_data_1.pdf]
