# Supplementary material for: Concurrent remodelling of nucleolar 60S subunit precursors by the Rea1 ATPase and Spb4 RNA helicase
Source: eLife. 2023 Mar 17;12:e84877. doi: 10.7554/eLife.84877 (PMC10154028; doi:10.7554/eLife.84877)

Figure 4–Figure Supplement 2B, left panel

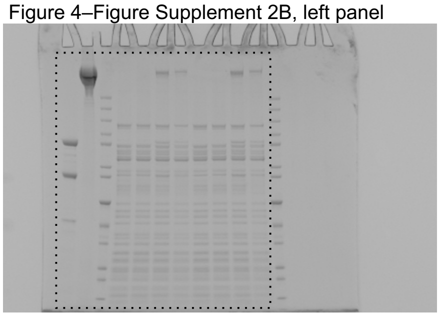

Figure 4–Figure Supplement 2B, right panel

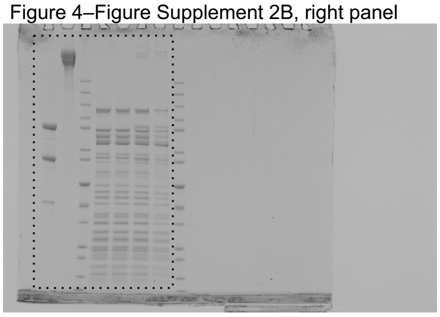

Figure 4–Figure Supplement 2B, left panel, anti-Ytm1

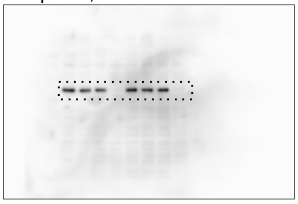

Figure 4–Figure Supplement 2B, left panel, anti-Has1

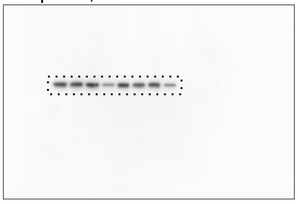

Figure 4–Figure Supplement 2B, left panel, anti-Ebp2

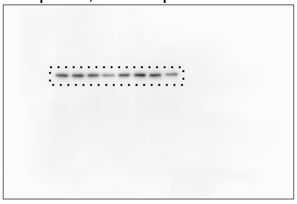

Figure 4–Figure Supplement 2B, left panel, anti-Noc3

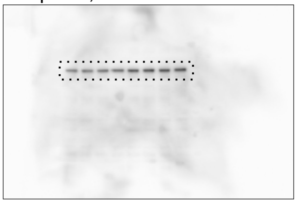

Figure 4–Figure Supplement 2B, left panel, anti-Nog1

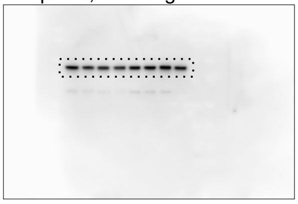

Figure 4–Figure Supplement 2B, left panel, anti-Nug1

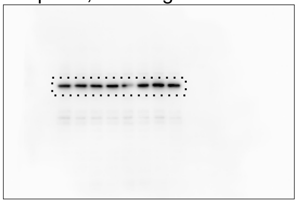

Figure 4–Figure Supplement 2B, left panel, anti-Nsa2

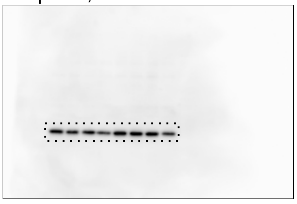

Figure 4–Figure Supplement 2B, left panel, anti-L3

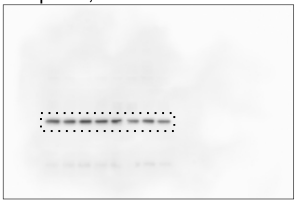

Figure 4–Figure Supplement 2B, right panel, anti-Ytm1

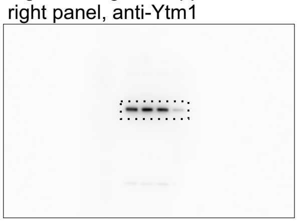

Figure 4–Figure Supplement 2B, right panel, anti-Has1

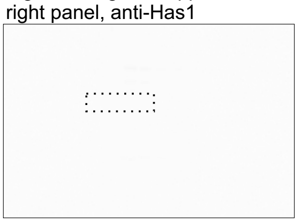

Figure 4–Figure Supplement 2B, right panel, anti-Ebp2

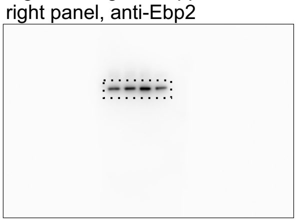

Figure 4–Figure Supplement 2B, right panel, anti-Noc3

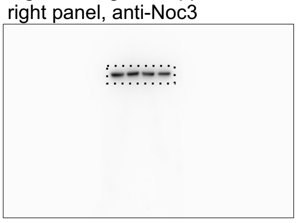

Figure 4–Figure Supplement 2B, right panel, anti-Nog1

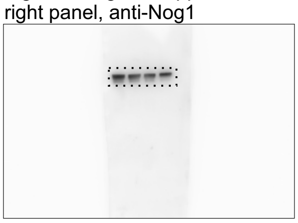

Figure 4–Figure Supplement 2B, right panel, anti-Nug1

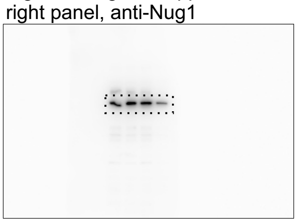

Figure 4–Figure Supplement 2B, right panel, anti-Nsa2

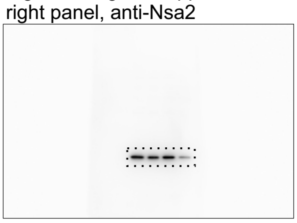

Figure 4–Figure Supplement 2B, right panel, anti-L3

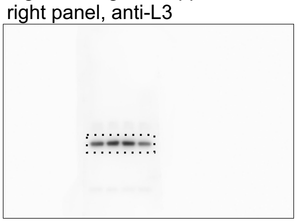

Supplement: Figure 4—figure supplement 2—source data 1. — Dashed boxes in the PDF indicate the respective areas shown in the figure. [file elife-84877-fig4-figsupp2-data1.zip › Figure4_Figure_Supplement2_Source_data1/Figure4_Figure_Supplement2_Source_data.pdf]
