# Supplementary material for: Concurrent remodelling of nucleolar 60S subunit precursors by the Rea1 ATPase and Spb4 RNA helicase
Source: eLife. 2023 Mar 17;12:e84877. doi: 10.7554/eLife.84877 (PMC10154028; doi:10.7554/eLife.84877)
Supplement: Supplementary file 1. [file elife-84877-supp1.docx]

**Supplementary File 1. Yeast strains used in this study**

| **Name** | **Genotype** | **Source** |
| --- | --- | --- |
| W303 | *ade2-1, his3-11,15, leu2-3,112, trp1-1, ura3-1, can1-100* | Thomas and Rothstein,1989 |
| *SPB4*-FTpA | W303 *MAT***a** *SPB4*-FTpA::nat*NT2* | this study |
| *RRP17*-FTpA | W303 *MAT***a** *RRP17*-FTpA::nat*NT2* | this study |
| *SPB4*-HA-AID | W303 *MAT*α *SPB4*-HA-AID:: HIS3MX6 P.*ADH1*-Os*TIR1*-9xmyc::*TRP1* | this study |
| *RRP17*-HA-AID | W303 *MAT*α *RRP17*-HA-AID:: HIS3MX6 P.*ADH1*-Os*TIR1*-9xmyc::*TRP1* | this study |
| *SPB4*-HA-AID *NOP7*-FTpA | W303 *MAT*α *SPB4*-HA-AID:: HIS3MX6 P.*ADH1*-Os*TIR1*-9xmyc::*TRP1 NOP7*-FTpA::natNT2 | this study |
| *SPB4*-HA-AID *NUG1*-FTpA | W303 *MAT*α *SPB4*-HA-AID:: HIS3MX6 P.*ADH1*-Os*TIR1*-9xmyc::*TRP1 NUG1*-FTpA::natNT2 | this study |
| *SPB4*-HA-AID *ARX1*-FTpA | W303 *MAT*α *SPB4*-HA-AID:: HIS3MX6 P.*ADH1*-Os*TIR1*-9xmyc::*TRP1 ARX1*-FTpA::natNT2 | this study |
| *RRP17*-HA-AID *NOP7*-FTpA | W303 *MAT*α *RRP17*-HA-AID:: HIS3MX6 P.*ADH1*-Os*TIR1*-9xmyc::*TRP1 NOP7*-FTpA::natNT2 | this study |
| *RRP17*-HA-AID *NUG1*-FTpA | W303 *MAT*α *RRP17*-HA-AID:: HIS3MX6 P.*ADH1*-Os*TIR1*-9xmyc::*TRP1 NUG1*-FTpA::natNT2 | this study |
| *RRP17*-HA-AID *ARX1*-FTpA | W303 *MAT*α *RRP17*-HA-AID:: HIS3MX6 P.*ADH1*-Os*TIR1*-9xmyc::*TRP1 ARX1*-FTpA::natNT2 | this study |
| TAP-HA-*YTM1* *RPL3*-Flag | W303 *MAT*α TAP-HA-*YTM1*::natNT2 *RPL3*-Flag::HIS3MX6 | this study |
| *HAS1*-HA-AID | W303 *MAT*α *HAS1*-HA-AID:: HIS3MX6 P.*ADH1*-Os*TIR1*-9xmyc::*TRP1* | this study |
| *HAS1*-HA-AID *SPB4*-FTpA | W303 *MAT*α *HAS1*-HA-AID:: HIS3MX6 P.*ADH1*-Os*TIR1*-9xmyc::*TRP1* *SPB4*-FTpA::natNT2 | this study |
| *SPB4* Shuffle | W303 *MAT***a** *spb4*::kanMX [Ycplac33-*SPB4*] | this study |
| PJ69-4A | *trp1-901, leu2-3,112, ura3-52, his3-200, gal4Δ, gal80Δ, LYS2::GAL1- HIS3, GAL2-ADE2, met2::GAL7-lacZ* | James *et al*. 1996 |
