# Supplementary material for: Concurrent remodelling of nucleolar 60S subunit precursors by the Rea1 ATPase and Spb4 RNA helicase
Source: eLife. 2023 Mar 17;12:e84877. doi: 10.7554/eLife.84877 (PMC10154028; doi:10.7554/eLife.84877)
Supplement: Supplementary file 2. [file elife-84877-supp2.docx]

**Supplementary File 2. Plasmids used in this study**

| **Name** | **Relevant Information** | **Source** |
| --- | --- | --- |
| YCplac111-TAP-Flag-*YTM1* | CEN*, LEU2,* P*YTM1, YTM1*, N-terminal TAP-Flag tag | Kater *et al*., 2017 |
| YCplac111-TAP-Flag-*ytm1*E80A | CEN*, LEU2,* P*YTM1, ytm1*E80A, N-terminal TAP-Flag tag | Kater *et al*., 2017 |
| YCplac111-*SPB4* | CEN*, LEU2,* P*SPB4, SPB4* | this study |
| YCplac111-*spb4*K57R | CEN*, LEU2,* P*SPB4, spb4*K57R | this study |
| YCplac111-*spb4*E173A | CEN*, LEU2,* P*SPB4, spb4*E173A | this study |
| YCplac111-*spb4*R360A | CEN*, LEU2,* P*SPB4, spb4*R360A | this study |
| YCplac111-*spb4*N563 | CEN*, LEU2,* P*SPB4, spb4*N563 (aa 1-563) | this study |
| YCplac111-*spb4*N531 | CEN*, LEU2,* P*SPB4, spb4*N531 (aa 1-531) | this study |
| YCplac111-*spb4*N470 | CEN*, LEU2,* P*SPB4, spb4*N470 (aa 1-470) | this study |
| YCplac111-*spb4*N405 | CEN*, LEU2,* P*SPB4, spb4*N405 (aa 1-405) | this study |
| YCplac111-*SPB4*-FTpA | CEN*, LEU2,* P*SPB4, SPB4*, C-terminal FTpA tag | this study |
| YCplac111-*spb4*K57R-FTpA | CEN*, LEU2,* P*SPB4, spb4*K57R, C-terminal FTpA tag | this study |
| YCplac111-*spb4*E173A-FTpA | CEN*, LEU2,* P*SPB4, spb4*E173A, C-terminal FTpA tag | this study |
| YCplac111-*spb4*R360A-FTpA | CEN*, LEU2,* P*SPB4, spb4*R360A, C-terminal FTpA tag | this study |
| YCplac111-*spb4*N563-FTpA | CEN*, LEU2,* P*SPB4, spb4*N563, C-terminal FTpA tag | this study |
| YCplac111-*spb4*N470-FTpA | CEN*, LEU2,* P*SPB4, spb4*N470, C-terminal FTpA tag | this study |
| YCplac111-*spb4*N405-FTpA | CEN*, LEU2,* P*SPB4, spb4*N405, C-terminal FTpA tag | this study |
| YCplac111-*RRP17* | CEN*, LEU2,* P*SPB4, RRP17* | this study |
| YCplac111-*rrp17*ΔN10 | CEN*, LEU2,* P*SPB4, rrp17*ΔN10 (aa 11-235) | this study |
| YCplac111-*rrp17*ΔN20 | CEN*, LEU2,* P*SPB4, rrp17*ΔN20 (aa 21-235) | this study |
| YCplac111-*rrp17*ΔN41 | CEN*, LEU2,* P*SPB4, rrp17*ΔN41 (aa 42-235) | this study |
| YCplac111-*rrp17*N216 | CEN*, LEU2,* P*SPB4, rrp17*N216 (aa 1-216) | this study |
| YCplac111-*rrp17*N200 | CEN*, LEU2,* P*SPB4, rrp17*N200 (aa 1-200) | this study |
| YCplac111-*rrp17*N176 | CEN*, LEU2,* P*SPB4, rrp17*N176 (aa 1-176) | this study |
| YCplac111-*rrp17*T154-L160>A | CEN*, LEU2,* P*SPB4, rrp17*T154-L160>7xA | this study |
| YCplac111-*rrp17* T154-L160>R | CEN*, LEU2,* P*SPB4, rrp17*T154-L160>7xR | this study |
| YCplac111-*HAS1* | CEN*, LEU2,* P*HAS1, HAS1* | this study |
| YCplac111-*has1*K92A | CEN*, LEU2,* P*HAS1, has1*K92A | this study |
| YCplac111-P*GAL1-10*-TAP-HA-*REA1* | CEN, *LEU2*, P*GAL1-10*, N-terminal TAP-HA tag | this study |
| YEplac112-P*GAL1-10*-*RIX1*-TEV-pA | 2μ, *TRP1*, P*GAL1-10*, C-terminal TEV-pA tag | Barrio-Garcia *et al*., 2016 |
| YEplac181-P2-*IPI1*-Cbp-P*GAL1-10*-P1-*IPI3* | 2μ, *LEU2*, P*GAL1-10*, C-terminal Ipi1 Cbp tag | this study |
| pG4BDC22-*SPB4* | CEN, *TRP1*, P*ADH1*, C-terminal Gal4-BD | this study |
| pG4ADN111-*RRP17* | CEN, *LEU2*, P*ADH1*, N-terminal Gal4-AD | this study |
| pG4ADN111-*rrp17* (1-176) | CEN, *LEU2*, P*ADH1*, N-terminal Gal4-AD | this study |
| pG4ADN111-*rrp17* (106-235) | CEN, *LEU2*, P*ADH1*, N-terminal Gal4-AD | this study |
| pG4ADN111-*rrp17* (177-235) | CEN, *LEU2*, P*ADH1*, N-terminal Gal4-AD | this study |
