# Supplementary material for: Concurrent remodelling of nucleolar 60S subunit precursors by the Rea1 ATPase and Spb4 RNA helicase
Source: eLife. 2023 Mar 17;12:e84877. doi: 10.7554/eLife.84877 (PMC10154028; doi:10.7554/eLife.84877)
Supplement: Supplementary file 3. [file elife-84877-supp3.docx]

**Supplementary File 3. Data collection, refinement and model statistics**

|  | **State E**  **(EMDB-**  **16267)**  **(PDB-**  **8BVN)** | **State D**  **(EMDB-**  **16272)**  **(PDB-**  **8BVU)** | **State E_withoutRrp17_**  **(EMDB-**  **16273)**  **(PDB-8BVV)** | **Release State**  **(EMDB-16275)**  **(PDB-**  **8BVY)** | **Release State ES27up**  **(EMDB-16276)** | **Release State with Spb4**  **(EMDB-**  **16277)** | **Release State with Nop53**  **(EMDB-16278)** |
| --- | --- | --- | --- | --- | --- | --- | --- |
| **Data collection & processing** |  |  |  |  |  |  |  |
| Camera | Gatan K2  Summit | Gatan K2  Summit | Gatan K2  Summit | Gatan K2  Summit | Gatan K2  Summit | Gatan K2  Summit | Gatan K2  Summit |
| Magnification | 130,000 | 130,000 | 130,000 | 130,000 | 130,000 | 130,000 | 130,000 |
| Voltage (kV) | 300 | 300 | 300 | 300 | 300 | 300 | 300 |
| Electron exposure (e^-^/Å²) | 42.4 | 42.4 | 42.4 | 46.8 | 46.8 | 46.8 | 46.8 |
| Defocus range (μm) | 0.4 - 4.0 | 0.4 - 4.0 | 0.4 - 4.0 | 0.5 - 3.5 | 0.5 - 3.5 | 0.5 - 3.5 | 0.5 - 3.5 |
| Pixel size (Å) | 1.059 | 1.059 | 1.059 | 1.059 | 1.059 | 1.059 | 1.059 |
| Symmetry imposed | C1 | C1 | C1 | C1 | C1 | C1 | C1 |
| Micrographs collected (no.) |  |  |  |  |  |  |  |
| Initial particle images (no.) | 586,609 | 1,046,219 | 459,610 | 490,891 | 950,501 | 490,891 | 490,891 |
| Final particle images (no.) | 239,642 | 35,948 | 29,081 | 67,484 | 29,118 | 18,326 | 14,472 |
| Map resolution (Å) | 2.7 | 3.0 | 3.1 | 3.0 | 3.0 | 3.7 | 3.7 |
| FSC threshold | 0.143 | 0.143 | 0.143 | 0.143 | 0.143 | 0.143 | 0.143 |
| **Refinement** |  |  |  |  |  |  |  |
| Model resolution (Å) | 2.7 | 3.0 | 3.1 | 3.0 |  |  |  |
| FSC threshold | 0.5 | 0.5 | 0.5 | 0.5 |  |  |  |
| Map sharpening B factor (Å^2^) | -80 | -60 | -55 | -65 |  |  |  |
| Model composition |  |  |  |  |  |  |  |
| Non-hydrogen atoms | 158,020 | 167,110 | 151,602 | 114,696 |  |  |  |
| Protein residues | 12,283 | 13,359 | 11,702 | 7,602 |  |  |  |
| Nucleotide residues | 2,857 | 2,876 | 2,791 | 2,577 |  |  |  |
| Ligands | 6 | 6 | 5 | 4 |  |  |  |
| R.m.s deviations |  |  |  |  |  |  |  |
| Bond lengths (Å) | 0.007 | 0.003 | 0.004 | 0.003 |  |  |  |
| Bond angles (°) | 1.027 | 0.769 | 0.763 | 0.733 |  |  |  |
| Validation |  |  |  |  |  |  |  |
| Molprobity score | 1.17 | 1.19 | 1.14 | 1.22 |  |  |  |
| Clash score | 3.03 | 3.76 | 3.48 | 3.79 |  |  |  |
| Poor rotamers (%) | 0.03 | 0.01 | 0.01 | 0.02 |  |  |  |
| Ramachandran plot |  |  |  |  |  |  |  |
| Favored (%) | 97.62 | 97.90 | 98.00 | 97.78 |  |  |  |
| Allowed (%) | 2.35 | 2.09 | 1.99 | 2.21 |  |  |  |
| Disallowed (%) | 0.02 | 0.01 | 0.01 | 0.01 |  |  |  |
| Map vs. Model CC (mask) | 0.86 | 0.85 | 0.86 | 0.84 |  |  |  |
